# Causal associations between Sarcopenia-related traits and obstructive sleep apnea: a mendelian randomization study

**DOI:** 10.1007/s40520-025-02963-3

**Published:** 2025-03-08

**Authors:** Huixian Sun, Xin Zeng, Wei Gao, Xiang Lu

**Affiliations:** 1 Department of Geriatrics, Sir Run Run Hospital, Nanjing Medical University, No.109 Longmian Avenue, Nanjing, Jiangsu 211166 China; 2 Department of Geriatrics, School of Medicine, Zhongda Hospital, Southeast University, No.87 Dingjiaqiao, Nanjing, Jiangsu 210009 China

**Keywords:** Sarcopenia-related traits, Obstructive sleep apnea, Mendelian randomization, Genetic analyses

## Abstract

**Background:**

Evidence for a causal relationship between sarcopenia and obstructive sleep apnea (OSA) is scarce. This study aimed to investigate the causal association between sarcopenia-related traits and OSA utilizing Mendelian randomization (MR) analyses.

**Methods:**

MR analyses were conducted using genetic instruments for sarcopenia-related traits, including hand grip strength, muscle mass, fat mass, water mass, and physical performance. Data from large-scale genome-wide association studies (GWAS) were utilized to identify genetic variants associated with these traits. Causal associations with OSA were assessed using various MR methods, including the inverse variance-weighted (IVW) method, MR-Egger, and weighted median approaches. Pleiotropy and heterogeneity were evaluated through MR-PRESSO and other sensitivity analyses.

**Results:**

Low hand grip strength in individuals aged 60 years and older exhibited a positive correlation with the risk of OSA (IVW, OR = 1.190, 95% CI = 1.003–1.413, *p* = 0.047), while no significant causal effects were observed for grip strength in the left and right hands. Muscle mass, fat mass, and water mass were significantly associated with OSA, even after adjusting for multiple testing. Notably, higher levels of body fat percentage, trunk fat percentage, and limb fat percentage were strongly correlated with increased risk of OSA. Physical performance indicators such as walking pace demonstrated an inverse association with OSA, while a higher risk of OSA was observed with increased log odds of falling risk and greater frequency of falls in the last year. Additionally, a causal effect was found between long-standing illness, disability, or infirmity and OSA.

**Conclusions:**

This comprehensive MR analysis provides evidence of a significant causal relationship between characteristics associated with sarcopenia, including low hand grip strength, muscle mass, fat mass, and physical performance, and the risk of OSA. These findings underscore the importance of addressing sarcopenia-related factors in the management and prevention of OSA.

**Supplementary Information:**

The online version contains supplementary material available at 10.1007/s40520-025-02963-3.

## Introduction

Sarcopenia is a skeletal muscle-related disorder. Studies have indicated that people with sarcopenia are at a higher risk of falls, cardiovascular disease, and type 2 diabetes [1]. Muscle strength, muscle mass, and physical performance are the three main indicators for the diagnosis of sarcopenia. At least two of these parameters are included in the diagnostic process, but different definitions of sarcopenia lead to varying cut-off points and standards. Hand grip strength is an efficient and valid measure of muscle strength. Low grip strength suggests further muscle mass assessment and predicts a series of adverse outcomes [2]. Appendicular lean mass (ALM) refers to the total skeletal muscle mass of the four limbs and approximately reflects overall body muscle content [3]. In clinical practice, physical performance is used to grade the severity of sarcopenia after a positive diagnosis, with walking pace, an objective measure, being the most widely applied indicator [4]. Sarcopenia is both an independent condition and a systemic disease. Consequently, an increasing number of studies have investigated sarcopenia-related adverse outcomes, including falls, frailty, reduced quality of life, and mortality.

Obstructive sleep apnea (OSA) is a prevalent and hazardous syndrome. Statistically, nearly 1 billion adults aged 30–69 years worldwide may suffer from OSA [5]. The main characteristic of OSA is chronic intermittent hypoxia, which increases the risk of systemic diseases such as diabetes [6, 7]. However, most patients are unaware of their affected breathing and the importance of seeking medical attention, leading to low diagnosis rates and increased healthcare costs [8].

Observational studies have suggested a detrimental relationship between sarcopenia and OSA, but the causality remains unclear [9]. Obesity and sarcopenic traits may also act synergistically in the development of OSA. For instance, pharyngeal and laryngeal muscle atrophy, combined with fat accumulation around the airway, could contribute to OSA [10]. Additionally, pro-inflammatory cytokines like interleukin-6 (IL-6) and tumor necrosis factor-α (TNF-α) are elevated in both sarcopenia and OSA [11]. Higher IL-6 levels can drive muscle atrophy, while TNF-α promotes muscle protein breakdown and inhibits muscle protein synthesis through specific signaling pathways. These observations indicate that exploring genetic variations could further clarify the causal link between sarcopenia and OSA.

Although randomized controlled trials (RCTs) are the gold standard in clinical research, they can sometimes lead to unreliable outcomes due to confounding variables and reverse causation. Additionally, RCTs are often time-consuming, costly, and come with ethical concerns. Mendelian randomization (MR) offers a powerful epidemiological tool that leverages genetic variants as instrumental variables (IVs) to account for confounders. MR is particularly valued for its ability to minimize the risk of reverse causality, thereby improving the robustness of causal inferences between exposures and clinical outcomes [12]. As such, MR analysis is an effective approach for exploring the causal relationship between sarcopenia and OSA.

In summary, the association between sarcopenia and OSA is frequently mediated through biological pathways and metabolism, including muscle atrophy, altered fat distribution, and inflammatory factors. Given the independence of genetic variation from lifestyle and environmental factors, SNPs identified through GWASs offer valuable insights into assessing causal relationships between exposures and outcomes. Leveraging large-scale GWAS data, this study employed two-sample MR analysis to investigate the causal relationship between sarcopenia and OSA, contributing to the development of more comprehensive treatment strategies for OSA patients and ultimately enhancing their quality of life.

## Design and methods

### Study design

**Fig. 1:**
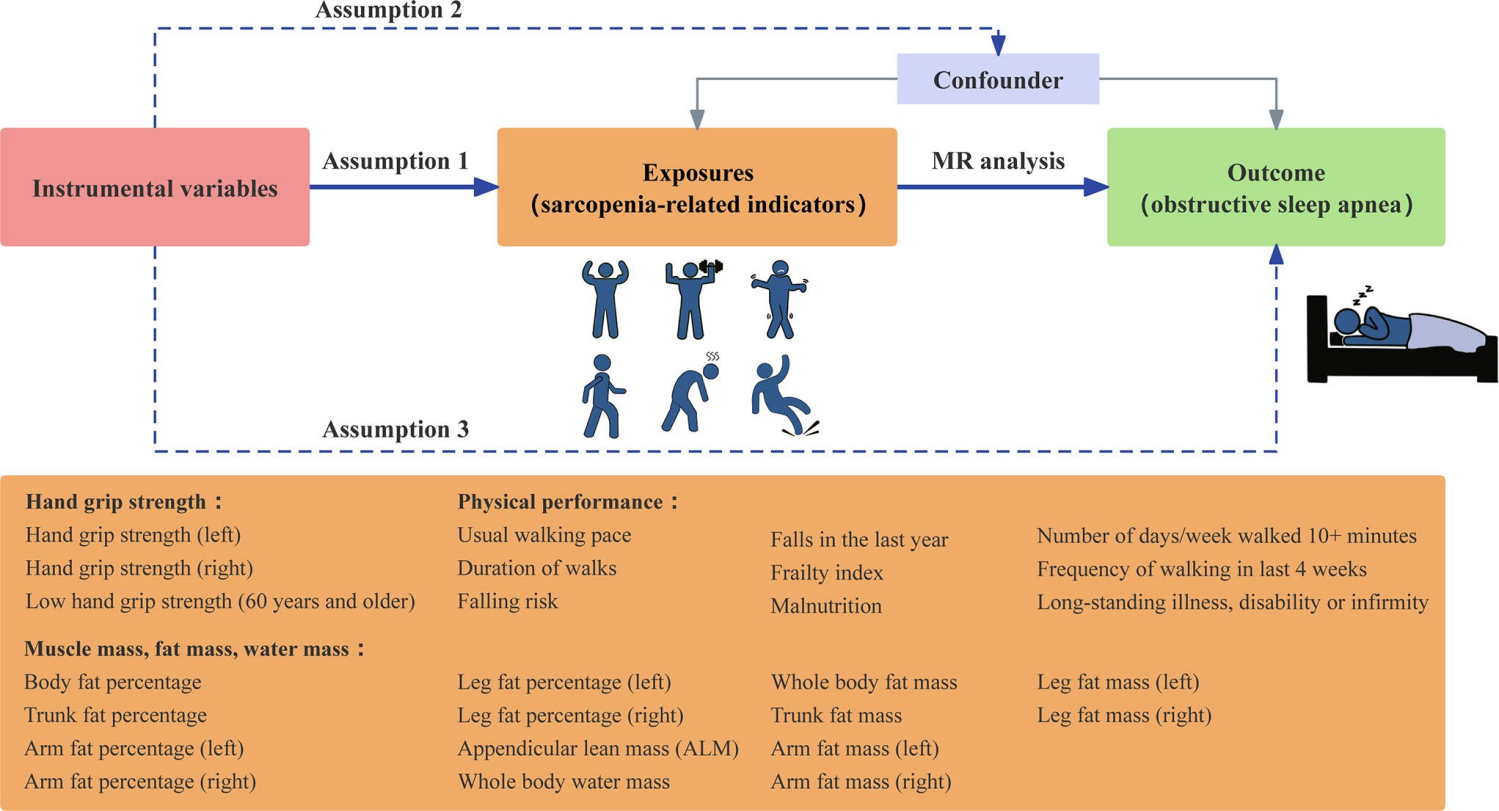
Overview of the design

Our study used a two-sample MR approach to investigate the causal association between sarcopenia-related traits and OSA. An overview of the design presented in Fig. 1. The genetic variants serving as instrumental variables (IVs), known as single-nucleotide polymorphisms (SNPs), must meet three essential criteria [13]: (1) they should strongly predict the exposures, (2) they should be exclusively associated with the outcome through the exposures, and (3) they should not be associated with any confounders that could influence the exposure-outcome relationship.

### Data sources

Based on a comprehensive analysis of the global epidemiological characteristics and impact of sarcopenia, the diagnostic methods, risk factors and clinical outcomes were considered as relevant key aspects [14]. The study underscored the detrimental effects of sarcopenia on health, emphasizing the need to standardize diagnostic criteria and improve detection in patients. Therefore, we included 26 sarcopenia-related traits across three domain: (1) muscular strength(hand grip strength (left), hand grip strength (right), low hand grip strength (60 years and older) (EWGSOP)), (2) body composition(body fat percentage, trunk fat percentage, arm fat percentage (left), arm fat percentage (right), leg fat percentage (left), leg fat percentage (right), appendicular lean mass (ALM), whole body water mass, whole body fat mass, trunk fat mass, arm fat mass (left), arm fat mass (right), leg fat mass (left), leg fat mass (right)), and (3) physical performance (usual walking pace, number of days/week walked 10 + minutes, frequency of walking for pleasure in last 4 weeks, duration of walks, falling risk, falls in the last year, frailty index, malnutrition, long-standing illness, disability or infirmity).

In terms of muscular strength, our study focused on hand grip strength, a crucial indicator that encompasses measurements for both the left and right hands as well as the criteria for low hand grip strength set forth by the European Working Group on Sarcopenia in Older People (EWGSOP), classifying individuals aged 60 and older with values below 30 kg for males and 20 kg for females.

In terms of body composition, sarcopenia is characterized by the loss of appendicular lean mass (ALM). However, ALM is not the sole determinant; the whole-body fat mass and water mass also exert significant influence on this condition. In light of this, our study selected whole-body mass, trunk mass, and extremities mass as key parameters. Additionally, we considered similar indicators such as limb, trunk, and whole-body fat percentages.

Physical performance is an essential measure that reflects the integrated functionality of the entire body, rather than the function of an isolated organ. The usual walking pace stands out as a frequently utilized metric in this context. Our analysis also encompassed other relevant aspects such as the duration and frequency of walking activities, including the number of days walked for 10 + minutes and the frequency of walking for leisure in the past four weeks. Furthermore, we examined fall-related factors, encompassing both the risk of falls and the actual number of falls within the preceding year. We also took into account indicators of malnutrition and frailty, including the frailty index and the presence of chronic illnesses, disabilities, or infirmities.

The above exposure datasets were obtained from the UK Biobank through the IEU Open GWAS project (https://gwas.mrcieu.ac.uk/). We also utilized GWAS data for OSA from the IEU Open GWAS Project, which contained 13,818 cases and 463,035 controls, for the outcome dataset. Data sources, participant demographics, ethnic backgrounds, and the studies included in our analysis are concisely outlined in Supplementary Table 1.

### Selection of instrumental variables

In line with the three core assumptions of MR analysis, we selected independent SNPs strongly associated with the exposures (*p* < 5 × 10^^−8^) as IVs for most indices. Due to the limited number of SNPs associated with indices such as falling risk, falls in the past year, and malnutrition, a more lenient threshold (*p* < 5 × 10^^−6^) was adopted to identify additional SNPs related to these phenotypes. This approach helps mitigate the risk of false negatives caused by Bonferroni correction and facilitates the identification of more candidate loci for further validation. To ensure that SNPs were not associated with any potential confounders and were independent of each other, we applied clumping to mitigate linkage disequilibrium (LD) among the IVs. Specifically, SNPs were clumped using a threshold of *r*^2^ < 0.001 and a physical distance of 10,000 kb [15]. Subsequently, palindromic SNPs were excluded from the harmonization process to prevent biased results stemming from the inability to determine the orientation of effector alleles in the analyses. MR Pleiotropy Residual Sum and Outlier (MR-PRESSO) was then utilized to identify and remove outlier SNPs, thereby preventing horizontal pleiotropy. The IVs that were eliminated due to being outliers are presented in Supplementary Table 2. The comprehensive steps of the screening process are outlined in Supplementary Table 3. To evaluate the relationship between IVs and exposure, the strength of selected IVs was estimated using F statistics. F statistics were calculated with the formula: F = beta.exposure^2^/ se.exposure^2^. IVs with F value > 10 was considered of a sufficient strength. All IVs with F value > 10 were considered of a sufficient strength of instrument [16]. The detailed information on all IVs ultimately used in our study is summarized in Supplementary Tables 5–30.

### Statistical analysis

The causal relationship between sarcopenia-related indicators and OSA was investigated using a range of MR methods, primarily implemented through the ‘TwoSampleMR’ package (version 0.5.6). These methods included inverse variance weighting (IVW), MR Egger, simple mode, weighted median, and weighted mode [17, 18]. Due to its superior estimation accuracy and test power, the IVW method was employed as the primary analytical approach. This method combines Wald estimates for each SNP using a meta-analysis approach to derive an overall estimate. In addition, we utilized the weighted median and the MR-Egger methods as supplementary analytical tools. The weighted median approach assumes that at least 50% of genetic variants are valid, making it suitable for situations where most IVs do not exhibit horizontal pleiotropy. Meanwhile, the MR-Egger method, which performs weighted linear regression of genetic outcome coefficients, was also employed, although it is generally less efficient than IVW. Heterogeneity among the selected IVs was assessed using Cochran’s Q statistic and its corresponding *P*-value. In the absence of significant heterogeneity, the estimates of random and fixed effects were consistent. If the *P*-value of the Cochran’s Q statistic is < 0.05, indicating significant heterogeneity, the random effects IVW model must be used instead of the fixed effects IVW model [19]. To address the potential impact of horizontal pleiotropy, we used the MR-PRESSO method for detection and adjustment [20]. We assessed the presence of overall horizontal pleiotropy by comparing the observed distances of all genetic variants from the regression line (derived from the IVW method) with the expected distances under the assumption of no horizontal pleiotropy. In MR-Egger regression, the significance of the intercept term serves as the indicator of horizontal pleiotropy. Additionally, the method identifies and excludes outliers that could bias the estimates, allowing for a retest to compare results before and after the exclusion. This approach enhances the accuracy and reliability of the MR analysis. Meanwhile, sensitivity analyses were conducted using the Leave-one-out sensitivity test, where each SNP was sequentially removed to assess its impact on the overall results. If the exclusion of any SNP did not significantly alter the findings, it indicated that the MR results were robust and not driven by any single genetic variant. The study results can also be effectively illustrated using visualization tools such as scatter plots and funnel plots. Scatter plots help visualize the relationship between genetic variation, exposure and outcome. A linear relationship between SNPs and the effect size of the exposure suggests a causal link. Funnel plots, on the other hand, are used to detect heterogeneity among genetic variants. Asymmetry in the funnel plot may indicate the presence of heterogeneity or other biases, which could affect the study’s conclusions.

## Results

### The causal association between Sarcopenia-related traits and OSA

The *P*-values from MR analyses and those adjusted by MR-PRESSO are presented in Fig. 2. The detailed results of the MR analyses, including the values for heterogeneity and pleiotropy tests, are shown in Supplementary Table 4. Figure 3 provides detailed information on the MR analyses, illustrating the causal associations between sarcopenia-related features and OSA. Scatter plots depicting the causal relationships between sarcopenia-related traits and OSA are visualized in Supplementary Figures A1-26. Forest plots showing the causal associations between sarcopenia-related traits and OSA are presented in Supplementary Figures B1-26. The leave-one-out analysis results for the causality of sarcopenia-related traits with OSA are visualized in Supplementary Figures C1-26. Funnel plots illustrating the causal associations between sarcopenia-related traits and OSA are shown in Supplementary Figures D1-26.

**Fig. 2:**
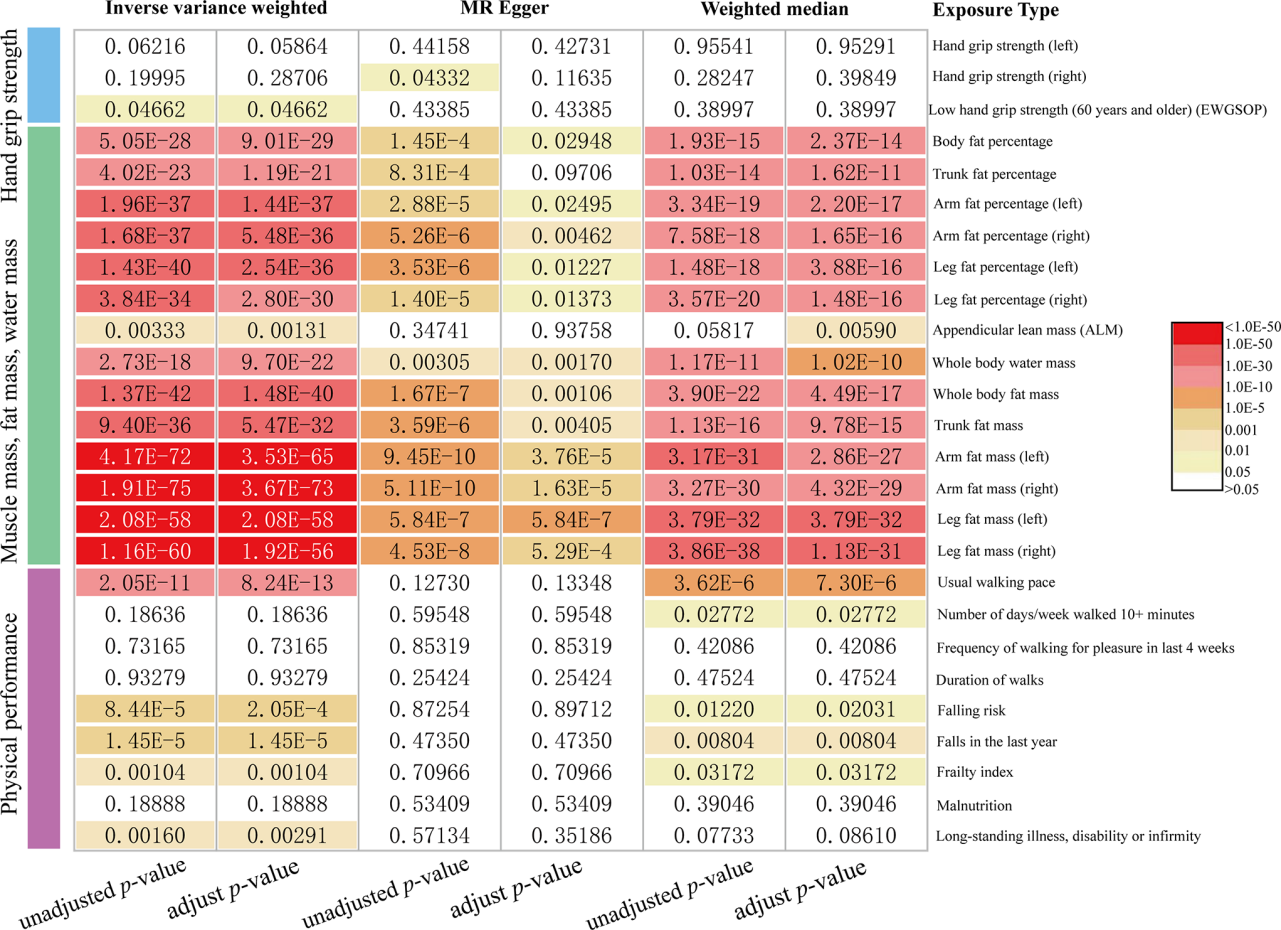
Results of main MR analyses with unadjusted *p*-values and adjusted *p*-values

### Hand grip strength and OSA

**Fig. 3:**
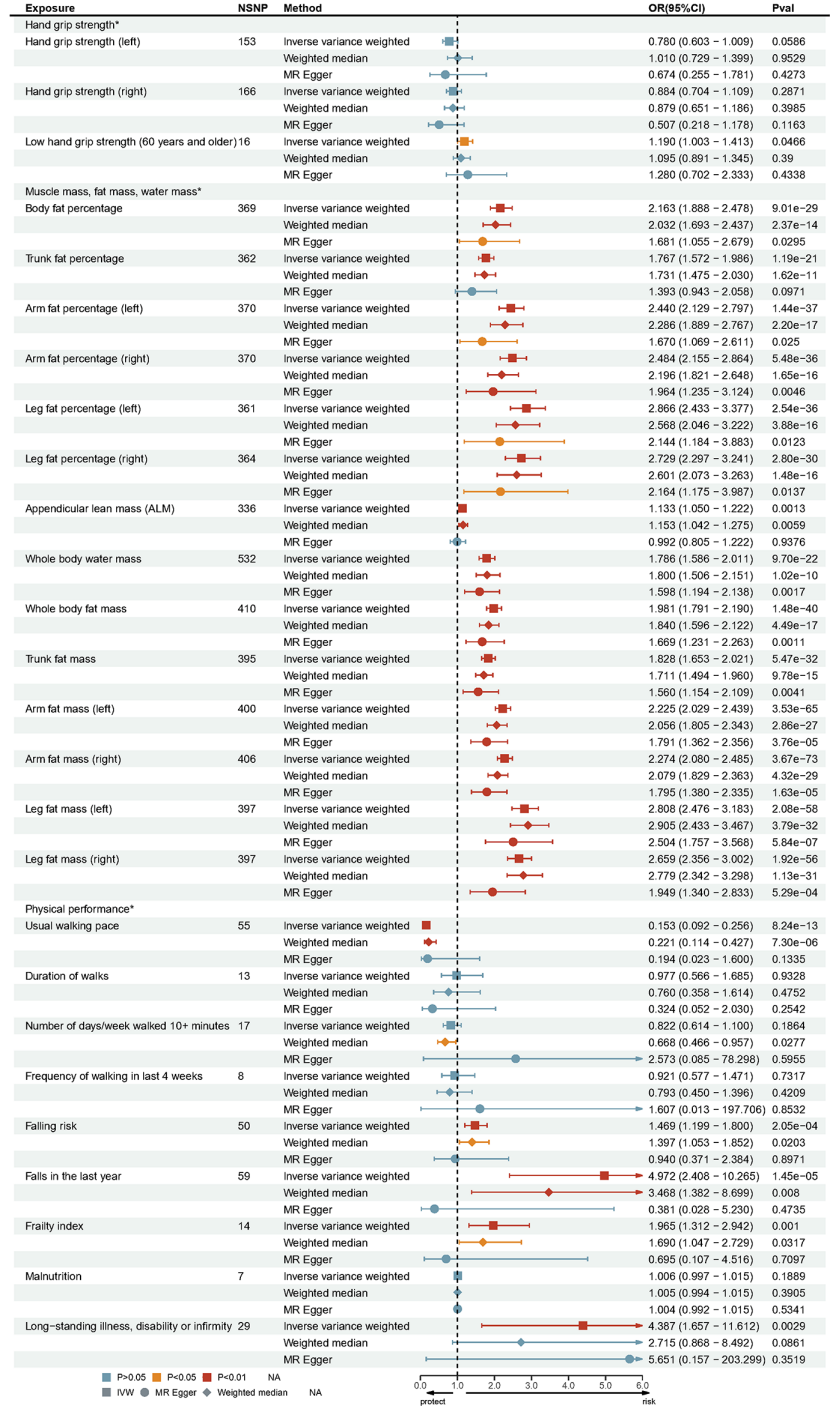
Detailed information about the causal relationships between sarcopenia-related traits and OSA. NSNP, number of instrumental SNPs used to process MR analyses; OR, odds ratio; Pval, *P*-value; IVW, Inverse variance weighted

Among the three grip strength targets, low hand grip strength in individuals aged 60 years and older was positively correlated with the risk of OSA. The odds ratio (OR) was 1.190 [95% confidence interval (95% CI), 1.003–1.413; *p* = 0.047] using the IVW method after excluding outliers identified by the MR-PRESSO test (Fig. 2). The *P*-value for the Egger-intercept test was 0.807, indicating no pleiotropic bias in assessing the effect of low grip strength in older adults with the IVW method. Leave-one-out analysis confirmed that the causal relationship was not driven by any single SNP. No significant causal effects were observed between hand grip strength (left and right) and OSA (Fig. 3).

### Muscle mass, fat mass, water mass, and OSA

Associations between appendicular lean mass (ALM), whole-body mass, trunk mass, extremities mass, and various fat percentages (limbs, trunk, and whole-body) with OSA were observed before adjusting the *p*-values. These associations remained significant after adjustment: OSA and ALM (IVW, OR = 1.133, 95% CI = 1.050–1.222, *p* = 0.001) Whole-body water mass (IVW, OR = 1.786, 95% CI = 1.586–2.011, *p* = 9.70E-22) Whole-body fat mass (IVW, OR = 1.981, 95% CI = 1.791–2.190, *p* = 1.48E-40) Trunk fat mass (IVW, OR = 1.828, 95% CI = 1.653–2.021, *p* = 5.47E-32) Left arm fat mass (IVW, OR = 2.225, 95% CI = 2.029–2.439, *p* = 3.53E-65) Right arm fat mass (IVW, OR = 2.274, 95% CI = 2.080–2.485, *p* = 3.67E-73) Left leg fat mass (IVW, OR = 2.808, 95% CI = 2.476–3.183, *p* = 2.08E-58) Right leg fat mass (IVW, OR = 2.659, 95% CI = 2.356–3.002, *p* = 1.92E-56) Body fat percentage (IVW, OR = 2.163, 95% CI = 1.888–2.478, *p* = 9.01E-29) Trunk fat percentage (IVW, OR = 1.767, 95% CI = 1.572–1.986, *p* = 1.19E-21) Left arm fat percentage (IVW, OR = 2.440, 95% CI = 2.129–2.797, *p* = 1.44E-37) Right arm fat percentage (IVW, OR = 2.484, 95% CI = 2.155–2.864, *p* = 5.48E-36) Left leg fat percentage (IVW, OR = 2.866, 95% CI = 2.433–3.377, *p* = 2.54E-36) Right leg fat percentage (IVW, OR = 2.729, 95% CI = 2.297–3.241, *p* = 2.80E-30) The results from MR Egger and the Weighted Median method were largely consistent. Additionally, no significant horizontal pleiotropy was detected by the MR-Egger intercept test (Supplementary Table 4).

### Physical performance and OSA

Usual walking pace was inversely associated with OSA using the IVW method, with an OR value of 0.153 (95% CI, 0.092–0.256; *p* = 8.24 × 10⁻¹³). The MR-Egger test indicated no significant pleiotropy in these results (*p* = 0.825). No causal associations were found between the duration of walks, the frequency of walking for pleasure in the last four weeks, the number of days per week walked for 10 + minutes, and OSA. A one-unit increase in the log odds of falling risk was associated with a 47% higher risk of OSA (OR, 1.469, 95% CI: 1.199-1.800, *p* < 0.001). More falls in the past year were related to a higher risk of OSA (OR, 4.972, 95% CI: 2.408–10.265, *p* < 0.001). A similar result was found between the frailty index and OSA (OR, 1.965, 95% CI: 1.312–2.942, *p* = 0.001). We found strong evidence supporting the causality of long-standing illness, disability, or infirmity on OSA (OR, 4.387, 95% CI: 1.657–11.612, *p* = 0.003). Additionally, the IVW, MR Egger, and Weighted Median results all suggested no causal effect of malnutrition on OSA. The results of sensitivity analyses are shown in Supplementary Table 4.

## Discussion

To the best of our knowledge, this is the first mendelian randomization study to systematically evaluate the causal relationships between sarcopenia-related traits and OSA in European population. Existing clinical studies have indicated that early-onset sarcopenia is more likely to occur in younger patients with OSA [21]. The sarcopenia index was negatively correlated with the odds ratio of sleep disorders. Maintaining optimal muscle mass may have a beneficial effect on OSA [22]. Our findings reveal that genetically determined low hand grip strength, falls, frailty, and disability are significantly associated with increased risks of OSA. Genetically predicted usual walking pace was negatively correlated with OSA, indicating that a lower walking speed increases the probability of suffering from sleep apnea. Additionally, muscle mass, fat mass, and water mass were positively related to OSA. However, we did not find a direct causal relationship between (left & right) hand grip strength, malnutrition, the duration and frequency of walking and OSA. Overall, the current MR results provide valuable insights into the impact of sarcopenia on OSA, highlighting the importance of considering skeletal muscle health in preventive and therapeutic strategies.

Observational investigations have demonstrated a transdiagnostic relationship between sleep duration and hand grip strength as well as the potential use of hand grip strength as a marker in OSA [23]. In the MR analysis, we strongly supported the effect of low grip strength on OSA, indirectly suggesting a causal relationship between sarcopenia and OSA. A cross-sectional study of Korean adults aged 40 to 80 years showed that low OSA risk was found to be associated with high grip strength after adjusting for other sleep parameters and confounders [24]. Another cross-sectional study involving Chinese individuals also reported that self-reported excessive daytime sleepiness accompanied by snoring or apnea was associated with the lowest grip strength [25]. The underlying physiological mechanism may involve obesity, insulin resistance, and sarcopenia converging on a shared pro-inflammatory pathway [26], triggering an inflammatory cascade that impairs muscle metabolism and function. This process may result in weakened muscle strength, such as reduced hand grip strength, ultimately increasing the risk of OSA. Furthermore, a HypnoLaus cohort study based on a Swiss population found that severe OSA measured using polysomnography was associated with decreased muscle strength. In adults over 60 years of age, low muscle strength directly affected OSA [27]. This finding aligned with our MR results that the more severe the low grip strength condition in individuals over 60 years of age, the higher the risk of OSA. However, no association was found between either left hand or right hand grip strength and OSA. Therefore, further research is required to explore the relationship between grip strength and OSA for different hand grip strength levels.

Previous studies have found that sleep apnea is associated with an increased risk of falls in older men [28, 29], independent of confounders. Sleep deprivation is a major cause of unintentional injuries from falls, but no studies have investigated whether the frequency of falls and the risk of falling increase the risk of OSA. Patients with sarcopenia experience decreased muscle strength, particularly in the lower limbs, leading to longer sitting or lying times [30]. Some studies have shown that a 20% reduction in muscle mass is associated with a reduced ability to perform activities of daily living and an increased risk of falls. The risk of death significantly increases when muscle mass loss reaches 40%. Our results suggest that fall is a risk factor for OSA, offering a new perspective for preventing OSA. Aging, inflammation, oxidative stress, and other factors may partially contribute to the development of sarcopenia and OSA [31, 32].

Treatment of frailty is an important approach to treating OSA, and sarcopenia is a major component of frailty [33]. Karla et al. found that a gender-stratified association between OSA risk and frailty in women but not in men [34]. Nevertheless, cross-sectional analyses from a prospective cohort study focusing on older men in the United States suggested that OSA was independently associated with greater evidence of frailty [35]. In addition, Omachi et al. reported that patients with OSA are at increased risk of recent work disability relative to patients without OSA [36]. In our study, a strong causal relationship was found between frailty, disability and OSA. This findings also strongly validate the above studies and provide a broader range of therapeutic strategies for OSA. In contrast, genome-wide association studies have not found a causal relationship between malnutrition and OSA. A retrospective intra-laboratory review of PSG data similarly confirmed that sleep-disordered breathing is common among the patients with muscular dystrophy (MD). Not all types of MD had the same degree of OSA or the same clinical manifestations. After adjusting for age, gender, body mass index and type, MD was marginally associated with OSA [37]. Although some reports have claimed that deficient vitamin D leads to a statistically higher risk of OSA, especially in children and adolescents [38]. However, evidence supporting the role of vitamin D and other nutritional indicators in adult OSA remains limited. Further research is needed to determine whether interventions for malnutrition can prevent the development of OSA.

Our MR study on the relationship of usual walking pace with OSA indicated that usual walking pace was a protective factor for OSA. A possible causal pathway for this finding involves the Rostral Fluid Shift hypothesis [39]. According to this hypothesis, during the day fluid accumulates in the intravascular and interstitial spaces of the legs due to gravity, and upon lying down at night redistributes rostrally, again owing to gravity. This fluid is displaced to the rostral side (towards the head), increasing the tendency to narrow the upper airway, thus predisposing to OSA [40]. Physical activity, such as walking, reduces fluid accumulation in the lower extremities, while increasing upper airway dilator muscle strength, reducing nasal resistance and improving sleep architecture [41]. A large population-based study also verified that a slower walking speed is associated with a greater prevalence of OSA [42]. Certain inflammatory pathways involve substances like IL-6 and TNF are implicated in both sarcopenia and OSA. Elevated levels of IL-6 and TNF, alongside decreased levels of IGF-1, are linked to reductions in muscle mass, strength, and function [26]. Evidence suggests that aerobic exercise, such as walking, can effectively downregulate these circulating inflammatory biomarkers. This indicates that regular physical activity plays a crucial role in mitigating age-related muscle changes. Consequently, this highlights the importance of focusing on interventions that promote walking pace as a means to prevent OSA. Nevertheless, a large number of observational and cohort studies are needed to demonstrate the effect of walking duration and frequency on OSA.

Different patterns of body fat distribution are genetic factors contributing to OSA. In our study, excess fat-whether located in the limbs or trunk was found to increase the risk of OSA. Existing research confirms that body fat tends to increase with age, peaking around 70 years old before gradually declining, while muscle mass begins to decrease after reaching a peak around age 40 [10]. Ongoing fat accumulation triggers pro-inflammatory responses, creating a negative feedback cycle that promotes the onset of sarcopenic obesity [43]. Specifically, fat buildup near the neck, often associated with central obesity, may contribute mechanically to upper airway obstruction during sleep, reduce lung volumes, and alter airflow dynamics, all of which increase airway collapsibility [11]. Additionally, inflammatory lipids release paracrine hormones and cytokines, leading to lipotoxicity, which impairs muscle fiber contractility and disrupts muscle protein synthesis. This exacerbates sarcopenia, leading to respiratory overload, increased oxygen consumption, and greater CO^2^ production [44]. However, resistance training has been shown to increase the number and size of muscle fibers (type IIA and IIX). This, in turn, improves glucose metabolism, enhances muscle protein synthesis, and reduces inflammatory cytokines levels [45], and ultimately mitigates sarcopenia while lowering the risk of OSA.

ALM is a sarcopenia-related trait mainly affected by skeletal muscle and is more heritable than whole-body lean mass [46, 47]. Contrary to the study by Liu et al. [48], we found a genetic causal relationship between ALM and OSA. Additionally, our results indicated that higher limb, trunk, and whole-body fat mass increases the risk of OSA, similar to a study showing a positive correlation between OSA severity and the muscle skeletal index reported by Takeshi et al. [49]. More observational studies are needed to investigate the relationship between skeletal muscle and OSA. Interestingly, we also found a strong link between whole-body water mass and OSA. This observation aligns with the findings of Hsu et al. [50], which suggested that increased truncal adiposity and body water mass are associated with a higher risk of low arousal threshold obstructive sleep apnea.

This study has several advantages. Firstly, our research is the first to use MR analysis to evaluate the effect of sarcopenia on OSA and innovatively propose that the diagnostic modality, risk factors and clinical outcomes of sarcopenia are all relevant features of sarcopenia. Secondly, the method minimizes bias due to confounding factors and reverse causality, while the reliability of the results was enhanced by the combined use of multiple MR analysis methods and sensitivity analysis methods. Lastly, unlike previous observational studies, MR analyses utilize genetic variations as IVs that are randomly assigned at conception. These genetic variations are independent of behavioral, psychological, socio-economic, and environmental exposures, enabling MR to reflect the lifelong impact of risk or protective factors on outcomes. This approach provides more robust evidence for the causal relationship between sarcopenia and OSA.

We must acknowledge several unavoidable limitations in this study. Although heterogeneity may exist in the MR analyses, we used the random-effects IVW model, which yields more conservative results. Even in the presence of heterogeneity, the findings remain relatively stable. Meanwhile, our application of MR Methods (IVW, MR-Egger, MR-PRESSO) was very robust, which ensured the credibility of the research results. To address the lack of SNPs at the genome-wide significance level for outcomes such as falling risk, falls in the past year, and malnutrition, a less stringent threshold (*p* < 5 × 10^^−6^) was applied to include more SNPs. However, this approach may increase the potential for confounding in the selected instrumental variables. Given that the findings are based solely on populations of European origin, the distinct characteristics of the European genome may impact the validity of the IVs and limit the generalizability of the results in MR analyses. Therefore, it is crucial to validate these findings in other genetic lineages. Future research should include cross-sectional and cohort studies using large databases of diverse ethnic group. The inability to stratify GWAS statistics by gender or age presents significant challenges, as confounding factors associated with these variables may influence the results. However, stratified analyses of other databases by gender and age have revealed that early-onset sarcopenia (characterized by low hand grip strength and slow walking pace) shows a significant association with OSA in individuals aged 18–39. In contrast, this association was not statistically significant in those aged 40–59 [21]. Notably, gender was found to have a significant impact on the relationship between sarcopenia and OSA. Therefore, while the potential effect of age as a confounding factor appears to be minimal, the influence of gender cannot be overlooked. To further enhance the reliability of MR results, future approaches such as Polygenic Risk Scores could be used to validate the connections between genetic and disease. Lifestyle profiling based on genetic exposure could also be used to assess genetic susceptibility to various diseases or traits. In addition, this study primarily relied on data from the MRC-IEU and the UK Biobank, which have inherent limitations, including possible measurement errors and sample selection bias. Addressing these limitations will allow for a more comprehensive exploration of the relationship between sarcopenia and OSA, thereby providing stronger support for the MR findings.

## Conclusion

In summary, our study identified a significant association between sarcopenia and OSA. We found that genetically determined factors, such as low hand grip strength, falls, frailty, and disability, were significantly linked to an increased risk of OSA. Genetically predicted usual walking pace was negatively correlated with OSA. Additionally, a genetically predicted slower walking pace was negatively correlated with OSA, while muscle mass, fat mass, and water mass showed positive relationships with OSA. However, we did not observe a direct causal relationship between hand grip strength (left & right), malnutrition, the duration and frequency of walking, and OSA. Furthermore, due to the lack of adjustment for confounding factors such as age, gender, and ethnicity, future prospective studies will be essential to explore the underlying mechanistic pathways and clarify the effects of sarcopenia on OSA.

## Electronic supplementary material

Below is the link to the electronic supplementary material.

Supplementary Material 1

Supplementary Material 2

## Data Availability

The GWAS summary data could be found at https://gwas.mrcieu.ac.uk/. The database ID of each GWAS and the data generated in our study could be found in Supplementary Material. The datasets generated during and/or analyzed during the current study are available from the corresponding author on reasonable request.
