# Supplementary material for: Causal associations between Sarcopenia-related traits and obstructive sleep apnea: a mendelian randomization study
Source: Aging Clin Exp Res. 2025 Mar 8;37(1):68. doi: 10.1007/s40520-025-02963-3 (PMC11889072; doi:10.1007/s40520-025-02963-3)
Supplement: Supplementary file 1 — Supplementary Material 1 [file 40520_2025_2963_MOESM1_ESM.docx]

| **STROBE-MR checklist** | | | |
| --- | --- | --- | --- |
| **No.** | **Section** | **Checklist item** | **Position** |
| 1 | Title and abstract | Indicate MR as the study’s design in the title and the abstract as a main purpose of the study | Title, Abstract |
| 2 | Background | Explain the scientific background and rationale for the reported study. Explain the exposure and a plausible potential causal relationship between exposure and outcome. Justify why MR is a helpful method to address the study question. | Introduction |
| 3 | Objectives | State specific objectives clearly, including prespecified causal hypotheses. State that MR is a method that intends to estimate causal effects. | Introduction |
| 4a | Study design and data sourses | Setting: Describe the study design (two-sample MR) and the underlying population . Describe the setting, locations, and relevant dates, including periods of recruitment, exposure, follow-up, and data collection. | Method: Study design |
| 4b |  | Participants: Report the eligibility criteria and the sources and methods of selection of participants. Report the sample size and whether any power or sample size calculations were carried out prior to the main analysis. | Method: Data sources, Table S1 |
| 4c |  | Describe measurement, quality control, and selection of genetic variants. | Method |
| 4d |  | For each exposure, outcome, and other relevant variables, describe methods of assessment and diagnostic criteria for diseases. | Methods: Data sources |
| 4e |  | Provide details of ethics committee approval and participant informed consent, if relevant. | Methods: Ehical approval and consent to participate |
| 5 | Assumptions | Explicitly state the 3 core instrumental variable (IV) assumptions for the main analysis (relevance, independence, and exclusion restriction), as well  assumptions for any additional or sensitivity analysis. | Methods: Study design and  Sensitivity analyses |
| 6a | Statistical methods: | Describe how quantitative variables were handled in the analyses. | Methods: Genetic analyses to elucidate causality |
| 6b |  | Describe how genetic variants were handled in the analyses and, if applicable, how their weights were selected |  |
| 6c |  | Describe the MR estimator and related statistics. Detail the included covariates  and, in case of 2-sample MR, whether the same covariate set was used for adjustment in the 2 samples. |  |
| 6d |  | Explain how missing data were addressed. | Method |
| 6e |  | Indicate how multiple testing was addressed (false discovery rate method) | Methods: Sensitivity analyses |
| 7 | Assessment of  assumptions | Describe any methods or prior knowledge used to assess the assumptions or justify their validity | N/A |
| 8 | Sensitivity analyses and additional analyses | Describe any sensitivity analyses or additional analyses performed (eg, comparison of effect estimates from different approaches, independent replication, bias analytic techniques, validation of instruments, simulations). | Method: Sensitivity analyses |
| 9a | Software and  preregistration | Name statistical software and package(s), including version and settings used. | Methods: Sensitivity analyses |
| 9b |  | State whether the study protocol and details were preregistered (as well as when and where). | N/A |
| 10a | Descriptive data | Report the numbers of individuals at each stage of included studies and reasons for exclusion. Use of a flow diagram. | Methods: Study design and Data sources |
| 10b |  | Report summary statistics for phenotypic exposure , outcomes, and other relevant variables (eg, means, SDs, proportions). | Results: Genetic instruments for exposures, Table S1 |
| 10c |  | If the data sources include meta-analyses of previous studies, provide the assessments of heterogeneity across these studies. | N/A |
| 10d |  | For 2-sample MR:  i. Provide justification of the similarity of the genetic variant–exposure associations between the exposure and outcome samples.  ii. Provide information on the number of individuals who overlap between the exposure and outcome studies. | N/A |
| 11a | Main results | Report the associations between genetic variant and exposure and between genetic variant and outcome, preferably on an interpretable scale. | Results: Genetic instruments for exposures, Tables S2-S4 |
| 11b |  | Report MR estimates of the relationship between exposure and outcome and the measures of uncertainty from the MR analysis, on an interpretable scale, such as odds ratio or relative risk per SD difference. | Results,  Tables S2-S4 |
| 11c |  | If relevant, consider translating estimates of relative risk into absolute risk for a meaningful time period. | N/A |
| 11d |  | Consider plots to visualize results (eg, forest plot, scatterplot of associations between genetic variants and outcome vs between genetic variants and exposure). | Supplementary Figures 1-5 |
| 12a | Assessment of  assumptions | Report the assessment of the validity of the assumptions by removing confounders-related SNPs. | Results, Tables 2-4 |
| 12b |  | Report any additional statistics (eg, assessments of heterogeneity across genetic variants, such as I2, Q statistic). |  |
| 13a | Sensitivity analyses and additional analyses | Report any sensitivity analyses to assess the robustness of the main results to violations of the assumptions. | Results , Tables 5, and S6 |
| 13b |  | Report results from other sensitivity analyses or additional analyses |  |
| 13c |  | Report any assessment of the direction of the causal relationship. |  |
| 13d |  | When relevant, report and compare with estimates from other RCTs and meta- analyses. | N/A |
| 13e |  | Consider additional plots to visualize results. | N/A |
| 14 | Key results | Summarize key results with reference to study objectives. | Discussion |
| 15 | Limitations | Discuss limitations of the study, taking into account the validity of the IV assumptions, other sources of potential bias, and imprecision. Discuss both direction and magnitude of any potential bias and any efforts to address them. | Discussion |
| 16a | Interpretation | Meaning: Give a cautious overall interpretation of results in the context of their limitations and in comparison with other studies. | Discussion |
| 16b |  | Mechanism: Discuss underlying biological mechanisms that could drive a potential causal relationship between the investigated exposure and the outcome, and whether the gene-environment equivalence assumption is reasonable. Use causal language carefully, clarifying that IV estimates may provide causal effects only under certain assumptions. |  |
| 16c |  | Clinical relevance: Discuss whether the results have clinical or public policy relevance, and to what extent they inform effect sizes of possible interventions. |  |
| 17 |  | Discuss the generalizability of the study results (a) to other populations, (b) across other exposure periods/timings, and (c) across other levels of exposure. |  |
| 18 | Funding | Describe sources of funding and the role offunders in the present study. | Funding statement |
| 19 | Data and data sharing | Provide the data used to perform all analyses or report where and how the data can be accessed, and reference these sources in the article. | Methods: Data sources |
| 20 | Conflicts of interest | All authors should declare all potential conflicts of interest. | Conflicts of interest |

**Three fundamental assumptions**

a. The instrument variables (IVs) must be associated with exposures;

b. The IVs must not be associated with any confounders, such as age, sex, lifestyle;

c. The IVs must influence the outcomes only through exposures and not through any direct or alternative pathways.
