# Supplementary material for: Causal associations between Sarcopenia-related traits and obstructive sleep apnea: a mendelian randomization study
Source: Aging Clin Exp Res. 2025 Mar 8;37(1):68. doi: 10.1007/s40520-025-02963-3 (PMC11889072; doi:10.1007/s40520-025-02963-3)
Supplement: Supplementary file 2 — Supplementary Material 2 [file 40520_2025_2963_MOESM2_ESM.docx]

**Supplementary Information:**

**Supplementary Figures:**

Supplementary Figure A1: Scatter plot of causal association of hand grip strength (left) with OSA;

Supplementary Figure A2: Scatter plot of causal association of hand grip strength (right) with OSA;

Supplementary Figure A3: Scatter plot of causal association of low hand grip strength (60 years and older) (EWGSOP) with OSA;

Supplementary Figure A4: Scatter plot of causal association of body fat percentage with OSA;

Supplementary Figure A5: Scatter plot of causal association of trunk fat percentage with OSA;

Supplementary Figure A6: Scatter plot of causal association of arm fat percentage (left) with OSA;

Supplementary Figure A7: Scatter plot of causal association of arm fat percentage (right) with OSA;

Supplementary Figure A8: Scatter plot of causal association of leg fat percentage (left) with OSA;

Supplementary Figure A9: Scatter plot of causal association of leg fat percentage (right) with OSA;

Supplementary Figure A10: Scatter plot of causal association of appendicular lean mass (ALM) with OSA;

Supplementary Figure A11: Scatter plot of causal association of whole body water mass with OSA;

Supplementary Figure A12: Scatter plot of causal association of whole body fat mass with OSA;

Supplementary Figure A13: Scatter plot of causal association of trunk fat mass with OSA;

Supplementary Figure A14: Scatter plot of causal association of arm fat mass (left) with OSA;

Supplementary Figure A15: Scatter plot of causal association of arm fat mass (right) with OSA;

Supplementary Figure A16: Scatter plot of causal association of leg fat mass (left) with OSA;

Supplementary Figure A17: Scatter plot of causal association of leg fat mass (right) with OSA;

Supplementary Figure A18: Scatter plot of causal association of usual walking pace with OSA;

Supplementary Figure A19: Scatter plot of causal association of duration of walks with OSA;

Supplementary Figure A20: Scatter plot of causal association of number of days/week walked 10+ minutes with OSA;

Supplementary Figure A21: Scatter plot of causal association of frequency of walking for pleasure in last 4 weeks with OSA;

Supplementary Figure A22: Scatter plot of causal association of falling risk with OSA;

Supplementary Figure A23: Scatter plot of causal association of falls in the last year with OSA;

Supplementary Figure A24: Scatter plot of causal association of frailty index with OSA;

Supplementary Figure A25: Scatter plot of causal association of Malnutrition with OSA;

Supplementary Figure A26: Scatter plot of causal association of long-standing illness, disability or infirmity with OSA;

Supplementary Figure B1: Forest plot of causal association of hand grip strength (left) with OSA;

Supplementary Figure B2: Forest plot of causal association of hand grip strength (right) with OSA;

Supplementary Figure B3: Forest plot of causal association of body fat percentage with OSA;

Supplementary Figure B4: Forest plot of causal association of trunk fat percentage with OSA;

Supplementary Figure B5: Forest plot of causal association of arm fat percentage (left) with OSA;

Supplementary Figure B6: Forest plot of causal association of arm fat percentage (right) with OSA;

Supplementary Figure B7: Forest plot of causal association of leg fat percentage (left) with OSA;

Supplementary Figure B8: Forest plot of causal association of leg fat percentage (right) with OSA;

Supplementary Figure B9: Forest plot of causal association of appendicular lean mass (ALM) with OSA;

Supplementary Figure B10: Forest plot of causal association of whole body water mass with OSA;

Supplementary Figure B11: Forest plot of causal association of whole body fat mass with OSA;

Supplementary Figure B12: Forest plot of causal association of trunk fat mass with OSA;

Supplementary Figure B13: Forest plot of causal association of arm fat mass (left) with OSA;

Supplementary Figure B14: Forest plot of causal association of arm fat mass (right) with OSA;

Supplementary Figure B15: Forest plot of causal association of leg fat mass (left) with OSA;

Supplementary Figure B16: Forest plot of causal association of leg fat mass (right) with OSA;

Supplementary Figure B17: Forest plot of causal association of low hand grip strength (60 years and older) (EWGSOP) with OSA;

Supplementary Figure B18: Forest plot of causal association of usual walking pace with OSA;

Supplementary Figure B19: Forest plot of causal association of duration of walks with OSA;

Supplementary Figure B20: Forest plot of causal association of number of days/week walked 10+ minutes with OSA;

Supplementary Figure B21: Forest plot of causal association of frequency of walking for pleasure in last 4 weeks with OSA;

Supplementary Figure B22: Forest plot of causal association of falling risk with OSA;

Supplementary Figure B23: Forest plot of causal association of falls in the last year with OSA;

Supplementary Figure B24: Forest plot of causal association of frailty index with OSA;

Supplementary Figure B25: Forest plot of causal association of Malnutrition with OSA;

Supplementary Figure B26: Forest plot of causal association of long-standing illness, disability or infirmity with OSA;

Supplementary Figure C1: Leave-one-out analysis for causal association of hand grip strength (left) with OSA;

Supplementary Figure C2: Leave-one-out analysis for causal association of hand grip strength (right) with OSA;

Supplementary Figure C3: Leave-one-out analysis for causal association of body fat percentage with OSA;

Supplementary Figure C4: Leave-one-out analysis for causal association of trunk fat percentage with OSA;

Supplementary Figure C5: Leave-one-out analysis for causal association of arm fat percentage (left) with OSA;

Supplementary Figure C6: Leave-one-out analysis for causal association of arm fat percentage (right) with OSA;

Supplementary Figure C7: Leave-one-out analysis for causal association of leg fat percentage (left) with OSA;

Supplementary Figure C8: Leave-one-out analysis for causal association of leg fat percentage (right) with OSA;

Supplementary Figure C9: Leave-one-out analysis for causal association of appendicular lean mass (ALM) with OSA;

Supplementary Figure C10: Leave-one-out analysis for causal association of whole body water mass with OSA;

Supplementary Figure C11: Leave-one-out analysis for causal association of whole body fat mass with OSA;

Supplementary Figure C12: Leave-one-out analysis for causal association of trunk fat mass with OSA;

Supplementary Figure C13: Leave-one-out analysis for causal association of arm fat mass (left) with OSA;

Supplementary Figure C14: Leave-one-out analysis for causal association of arm fat mass (right) with OSA;

Supplementary Figure C15: Leave-one-out analysis for causal association of leg fat mass (left) with OSA;

Supplementary Figure C16: Leave-one-out analysis for causal association of leg fat mass (right) with OSA;

Supplementary Figure C17: Leave-one-out analysis for causal association of low hand grip strength (60 years and older) (EWGSOP) with OSA;

Supplementary Figure C18: Leave-one-out analysis for causal association of usual walking pace with OSA;

Supplementary Figure C19: Leave-one-out analysis for causal association of duration of walks with OSA;

Supplementary Figure C20: Leave-one-out analysis for causal association of number of days/week walked 10+ minutes with OSA;

Supplementary Figure C21: Leave-one-out analysis for causal association of frequency of walking for pleasure in last 4 weeks with OSA;

Supplementary Figure C22: Leave-one-out analysis for causal association of falling risk with OSA;

Supplementary Figure C23: Leave-one-out analysis for causal association of falls in the last year with OSA;

Supplementary Figure C24: Leave-one-out analysis for causal association of frailty index with OSA;

Supplementary Figure C25: Leave-one-out analysis for causal association of Malnutrition with OSA;

Supplementary Figure C26: Leave-one-out analysis for causal association of long-standing illness, disability or infirmity with OSA;

Supplementary Figure D1: Funnel plot of causal association of hand grip strength (left) with OSA;

Supplementary Figure D2: Funnel plot of causal association of hand grip strength (right) with OSA;

Supplementary Figure D3: Funnel plot of causal association of low hand grip strength (60 years and older) (EWGSOP) with OSA;

Supplementary Figure D4: Funnel plot of causal association of body fat percentage with OSA;

Supplementary Figure D5: Funnel plot of causal association of trunk fat percentage with OSA;

Supplementary Figure D6: Funnel plot of causal association of arm fat percentage (left) with OSA;

Supplementary Figure D7: Funnel plot of causal association of arm fat percentage (right) with OSA;

Supplementary Figure D8: Funnel plot of causal association of leg fat percentage (left) with OSA;

Supplementary Figure D9: Funnel plot of causal association of leg fat percentage (right) with OSA;

Supplementary Figure D10: Funnel plot of causal association of appendicular lean mass (ALM) with OSA;

Supplementary Figure D11: Funnel plot of causal association of whole body water mass with OSA;

Supplementary Figure D12: Funnel plot of causal association of whole body fat mass with OSA;

Supplementary Figure D13: Funnel plot of causal association of trunk fat mass with OSA;

Supplementary Figure D14: Funnel plot of causal association of arm fat mass (left) with OSA;

Supplementary Figure D15: Funnel plot of causal association of arm fat mass (right) with OSA;

Supplementary Figure D16: Funnel plot of causal association of leg fat mass (left) with OSA;

Supplementary Figure D17: Funnel plot of causal association of leg fat mass (right) with OSA;

Supplementary Figure D18: Funnel plot of causal association of usual walking pace with OSA;

Supplementary Figure D19: Funnel plot of causal association of duration of walks with OSA;

Supplementary Figure D20: Funnel plot of causal association of number of days/week walked 10+ minutes with OSA;

Supplementary Figure D21: Funnel plot of causal association of frequency of walking for pleasure in last 4 weeks with OSA;

Supplementary Figure D22: Funnel plot of causal association of falling risk with OSA;

Supplementary Figure D23: Funnel plot of causal association of falls in the last year with OSA;

Supplementary Figure D24: Funnel plot of causal association of frailty index with OSA;

Supplementary Figure D25: Funnel plot of causal association of Malnutrition with OSA;

Supplementary Figure D26: Funnel plot of causal association of long-standing illness, disability or infirmity with OSA.


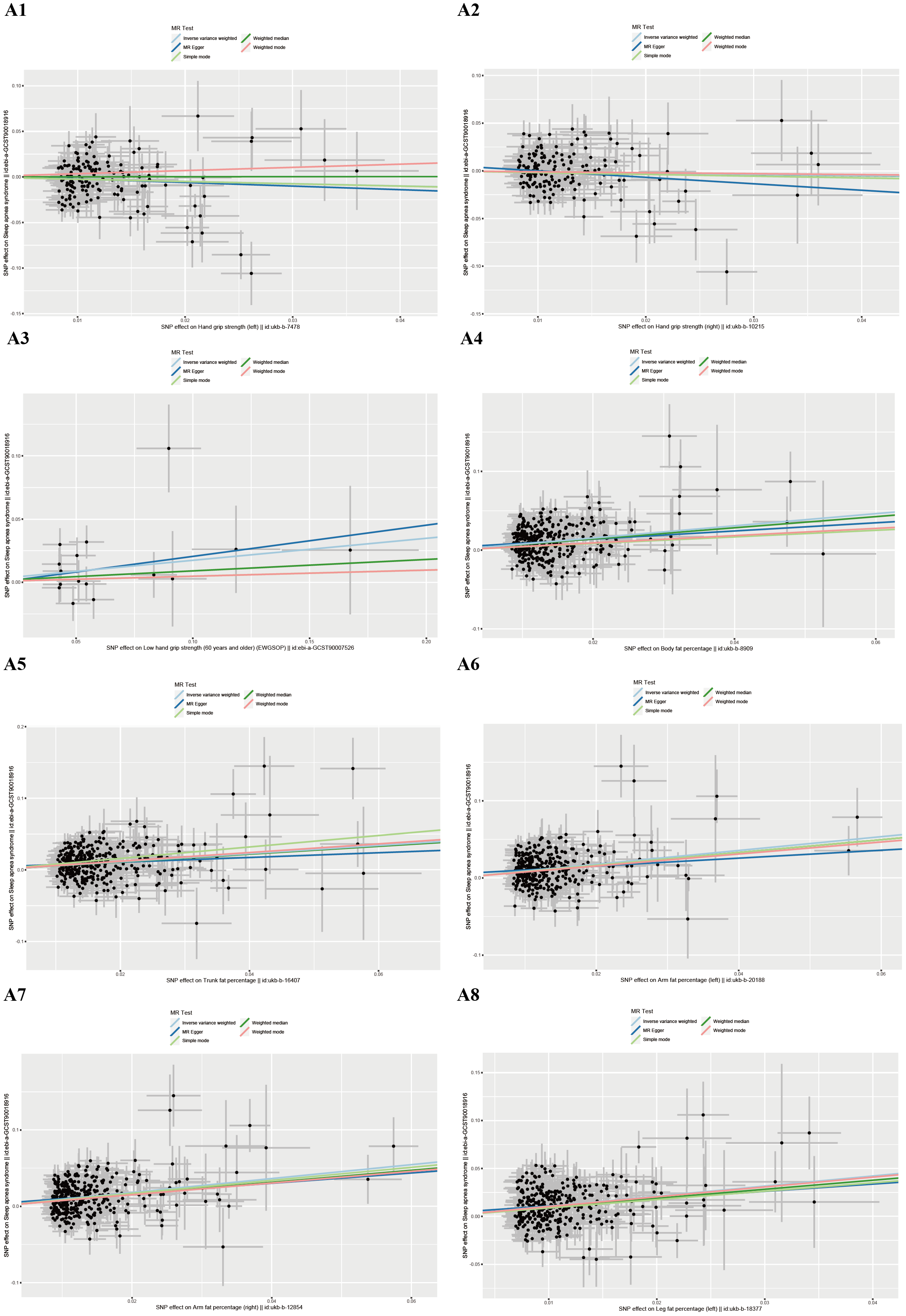


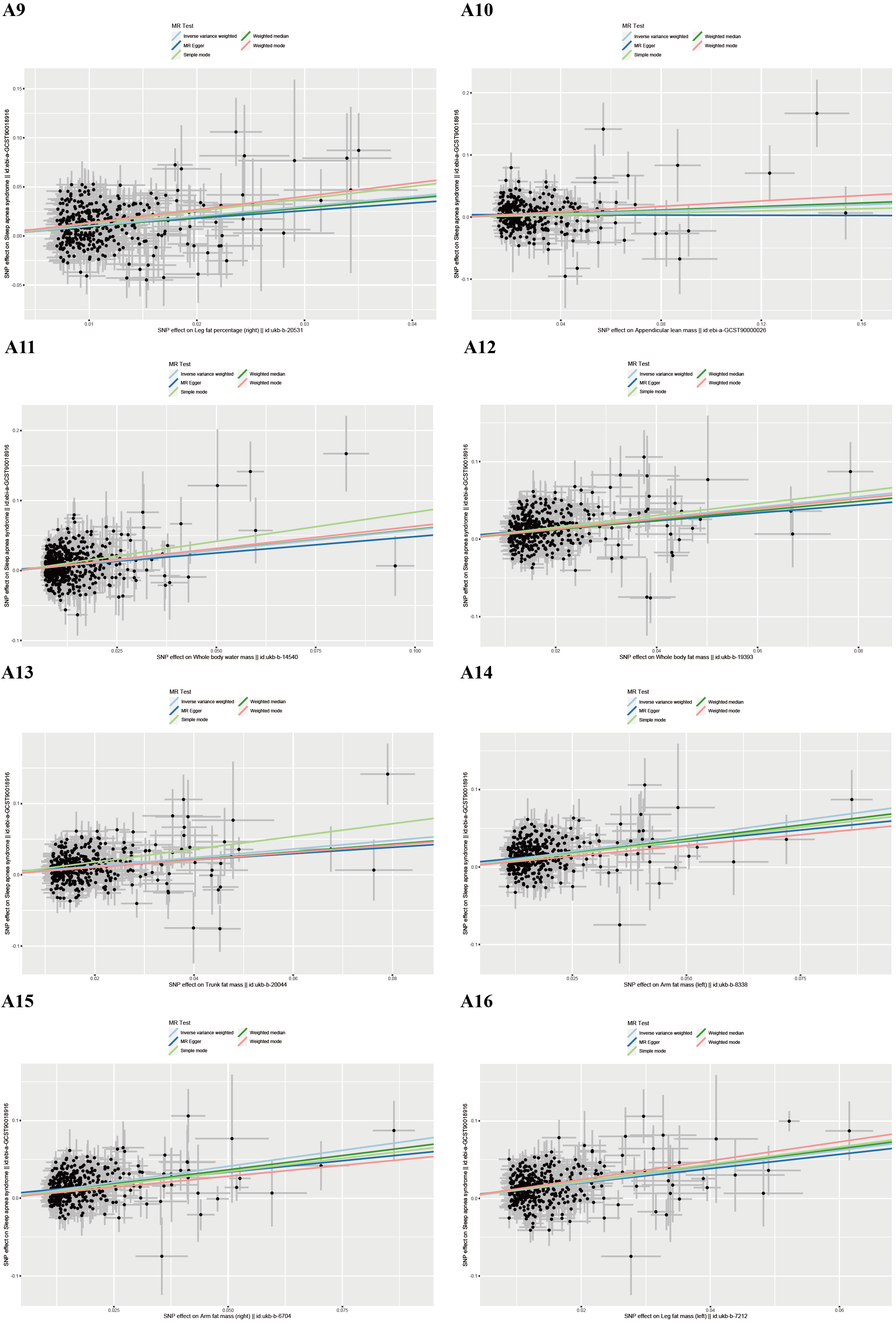


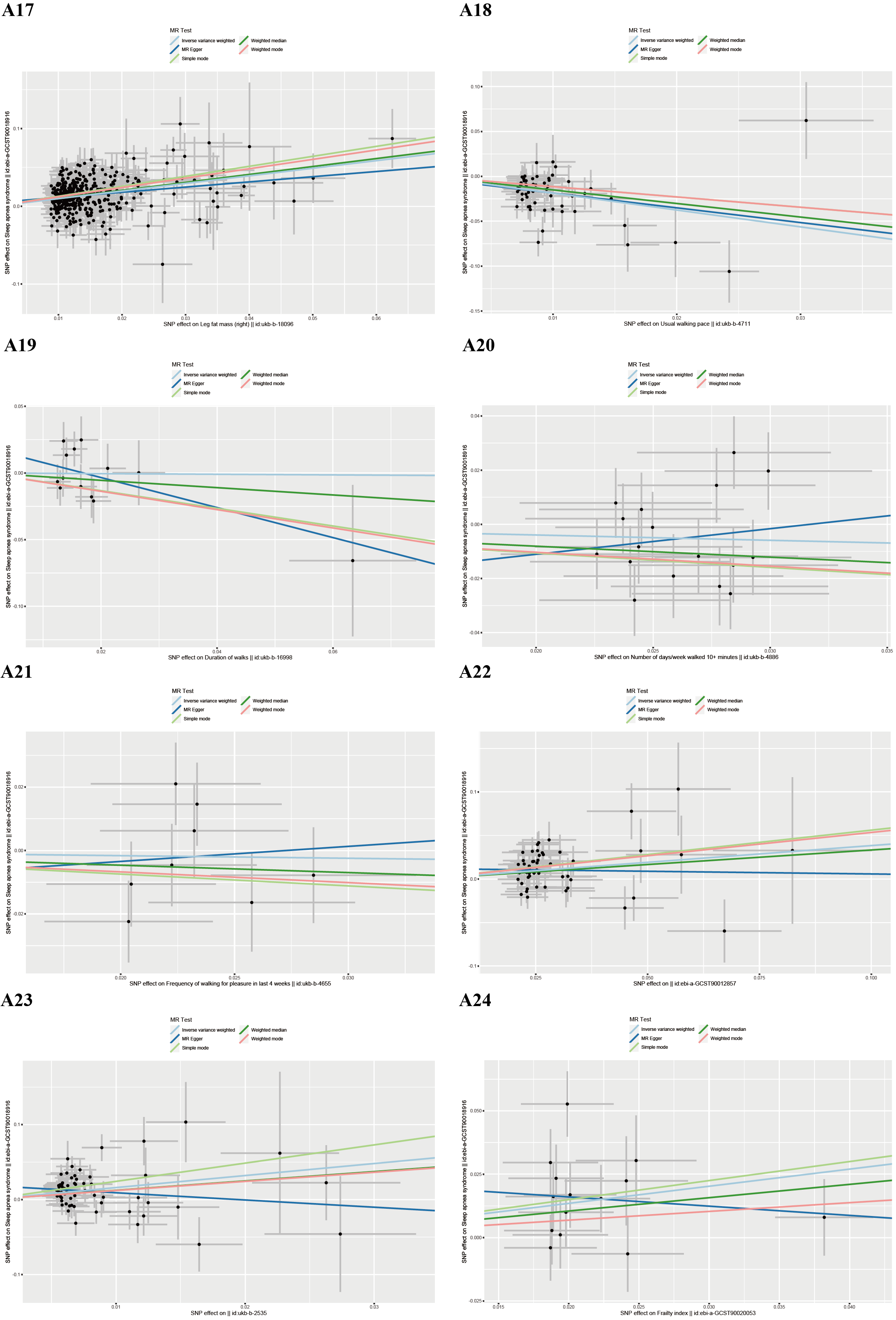


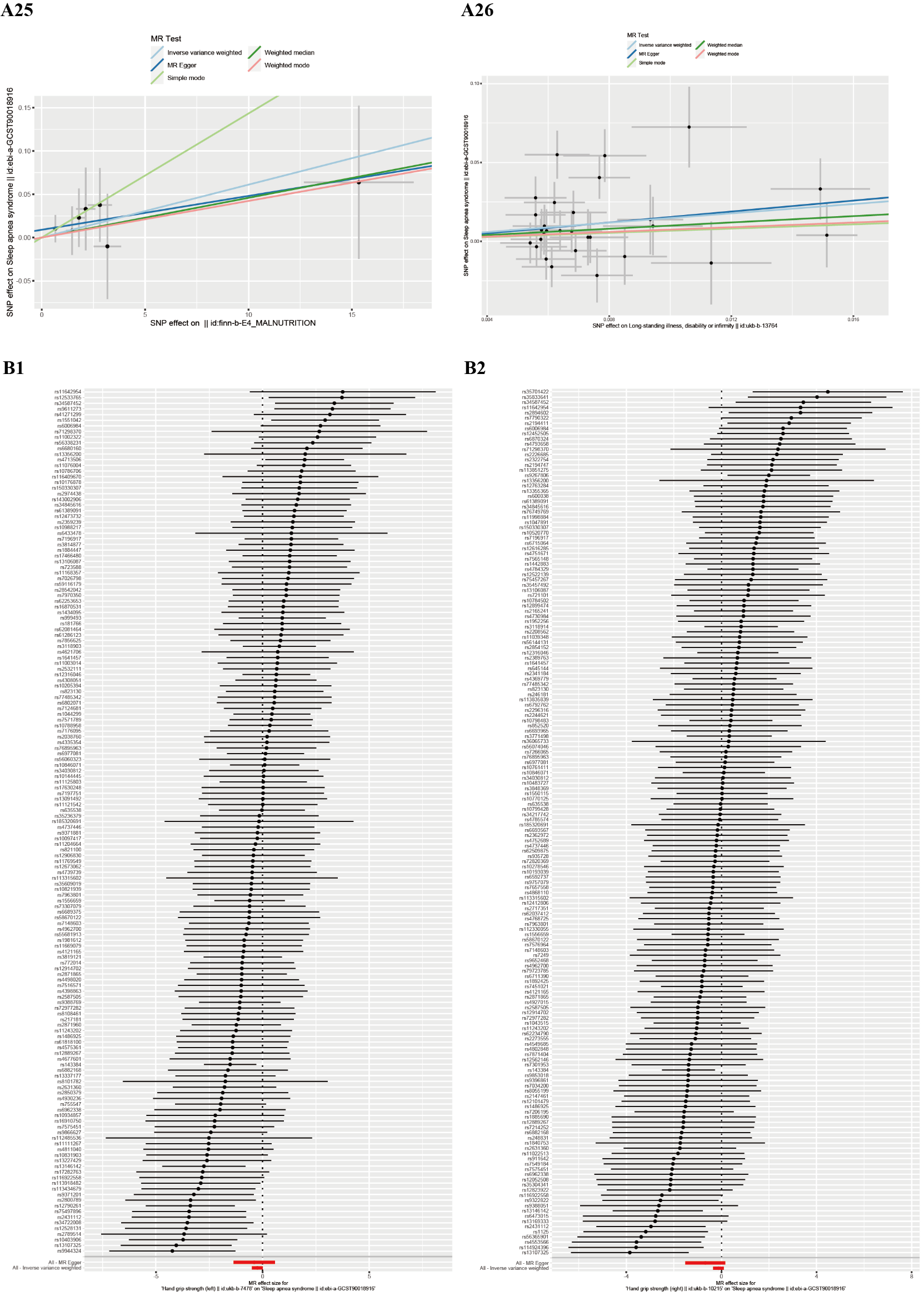


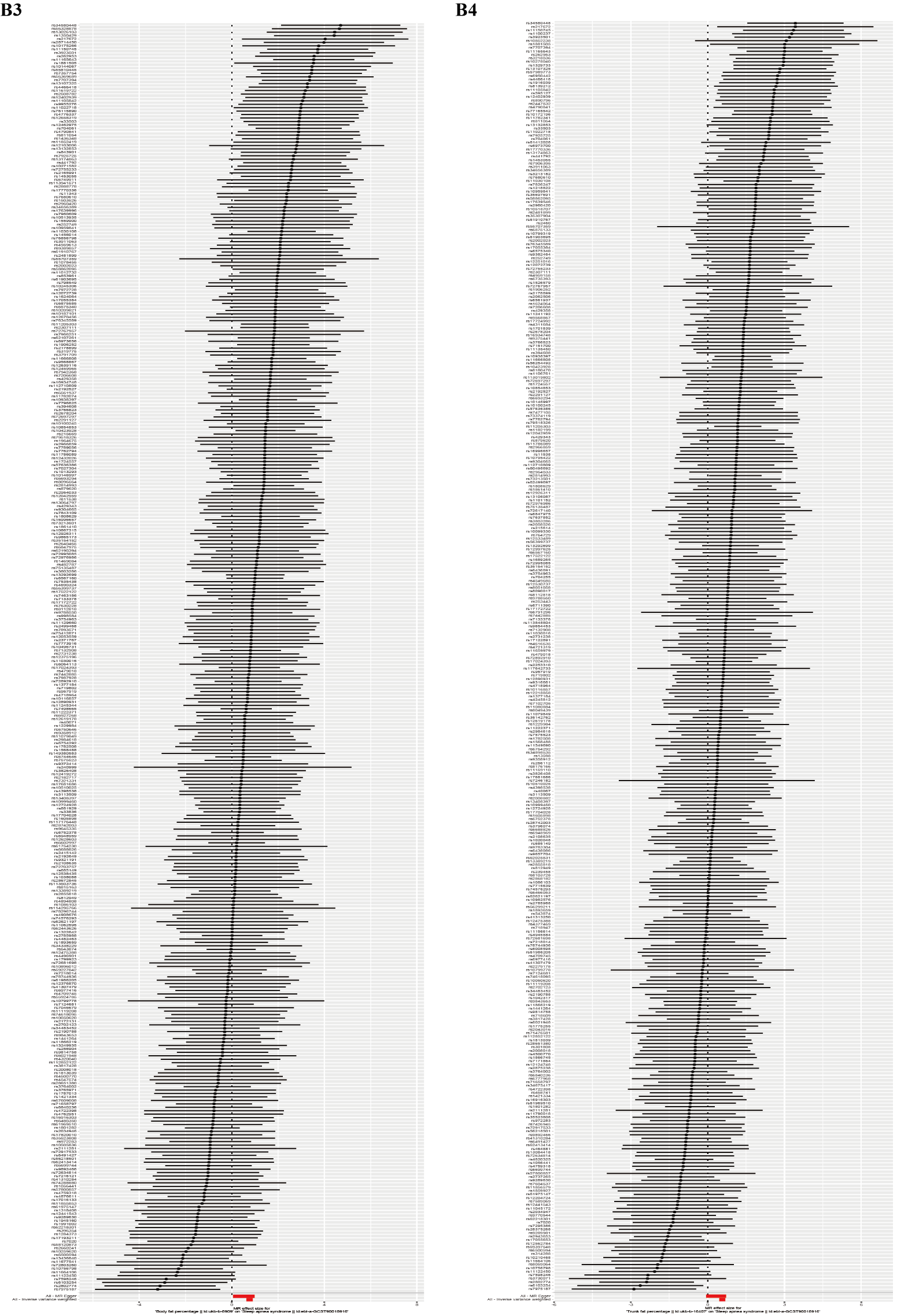


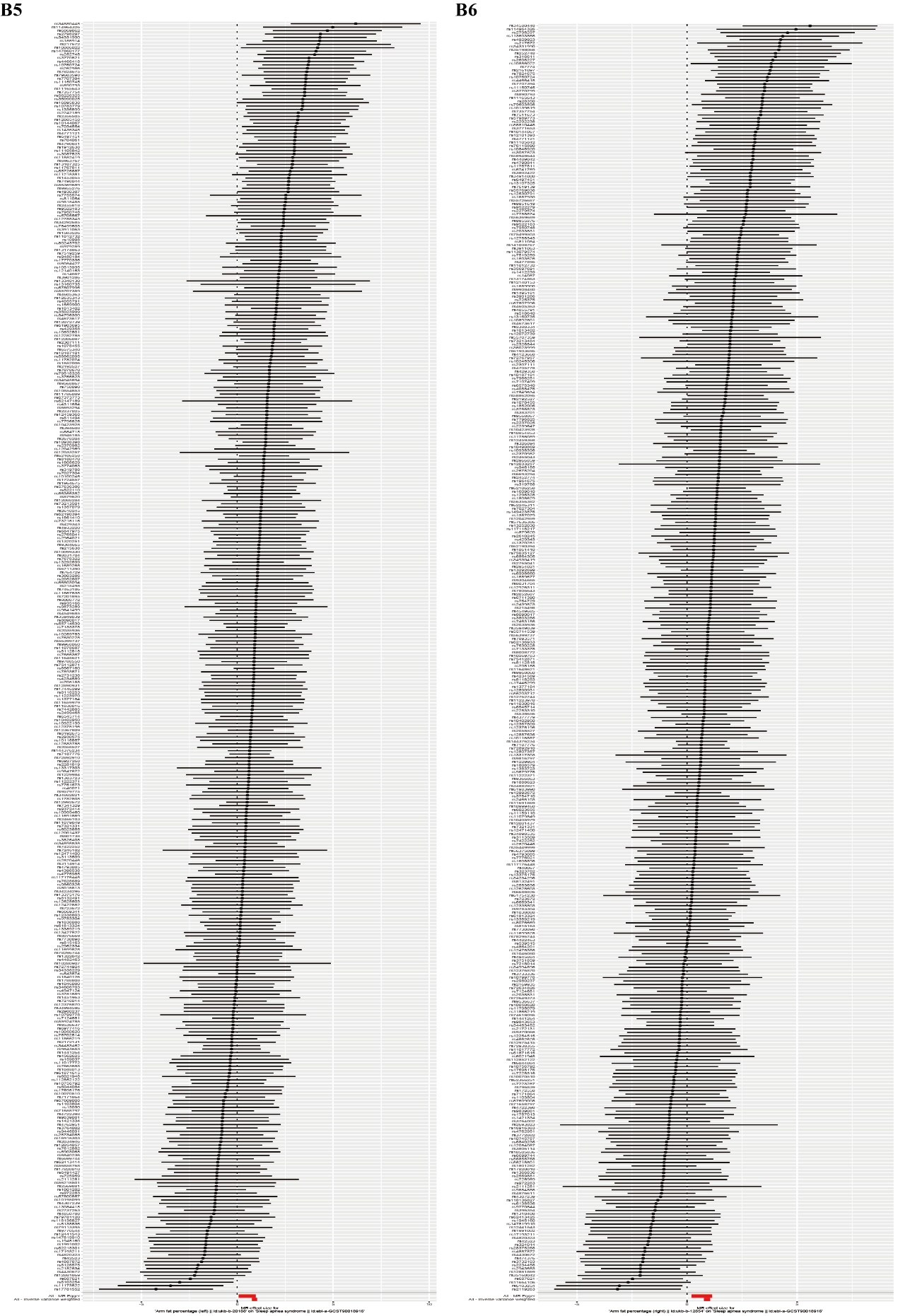


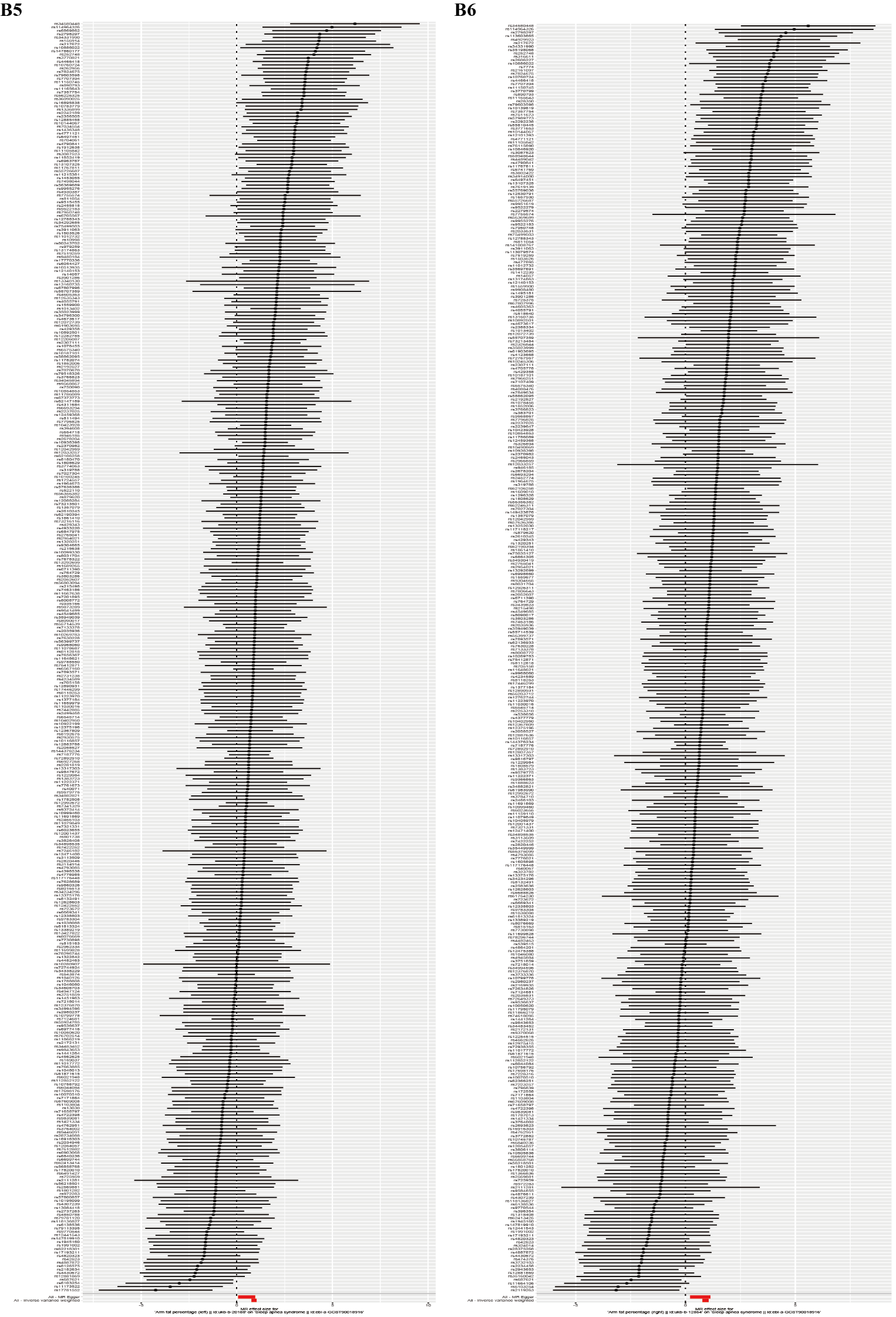


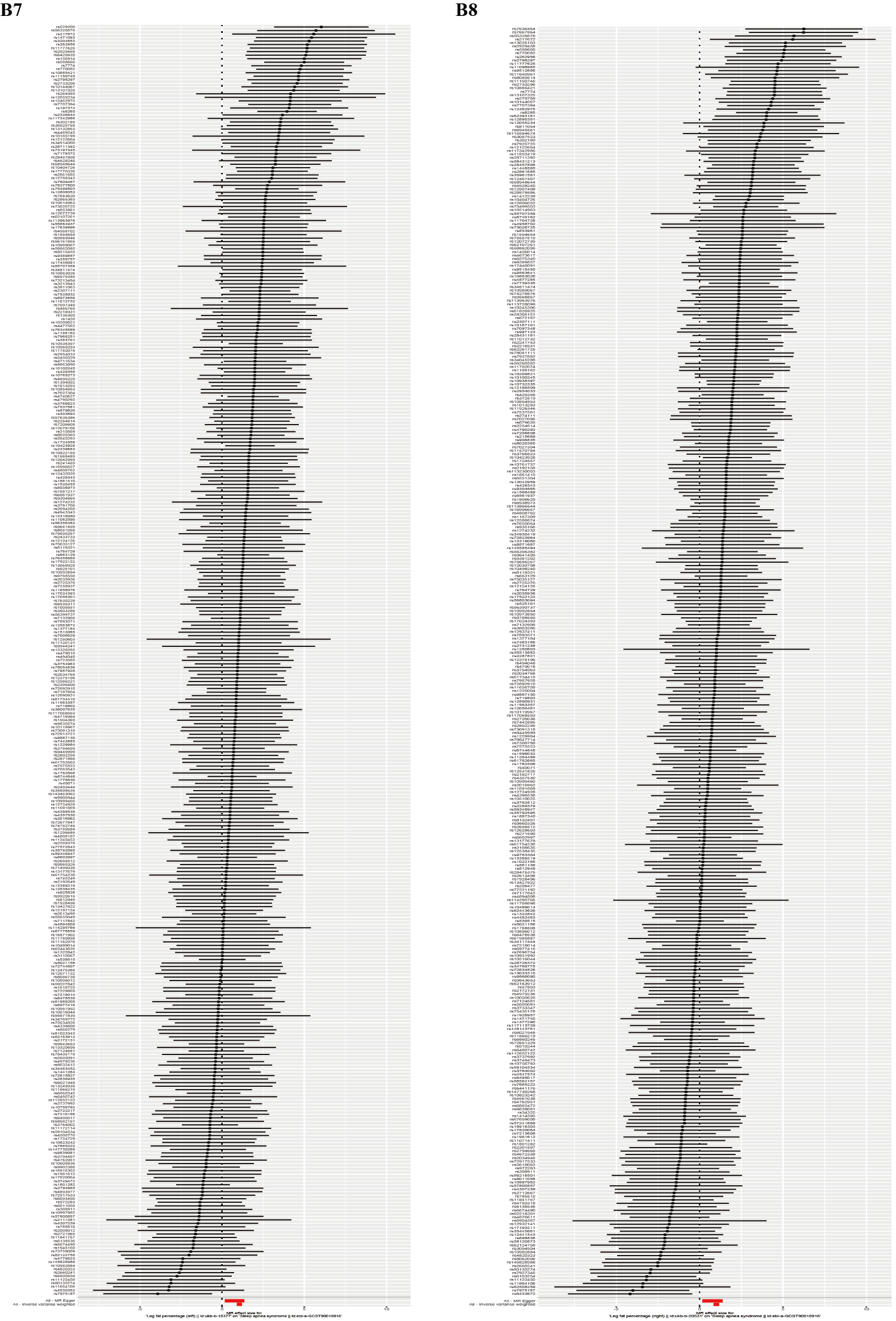


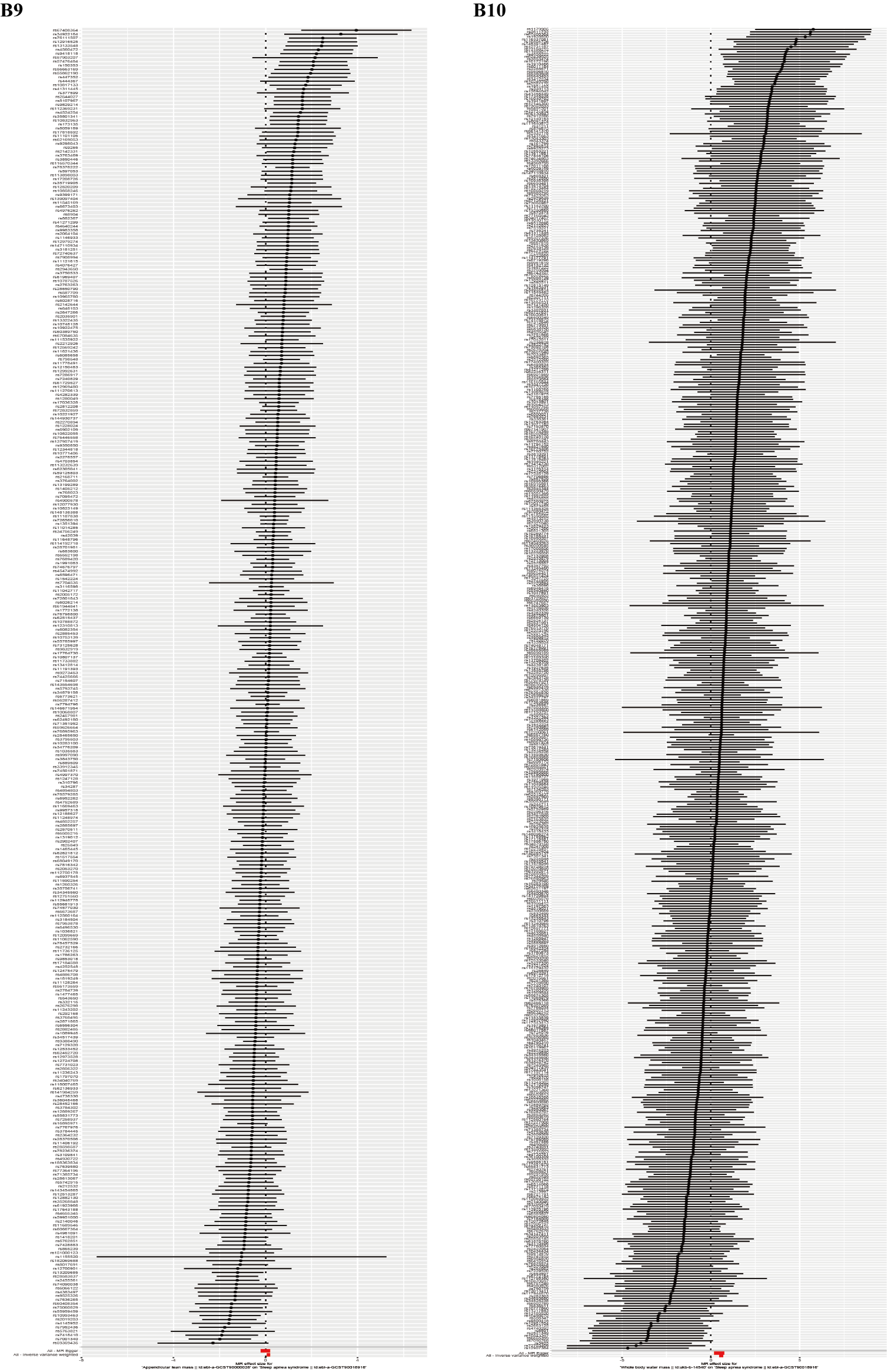


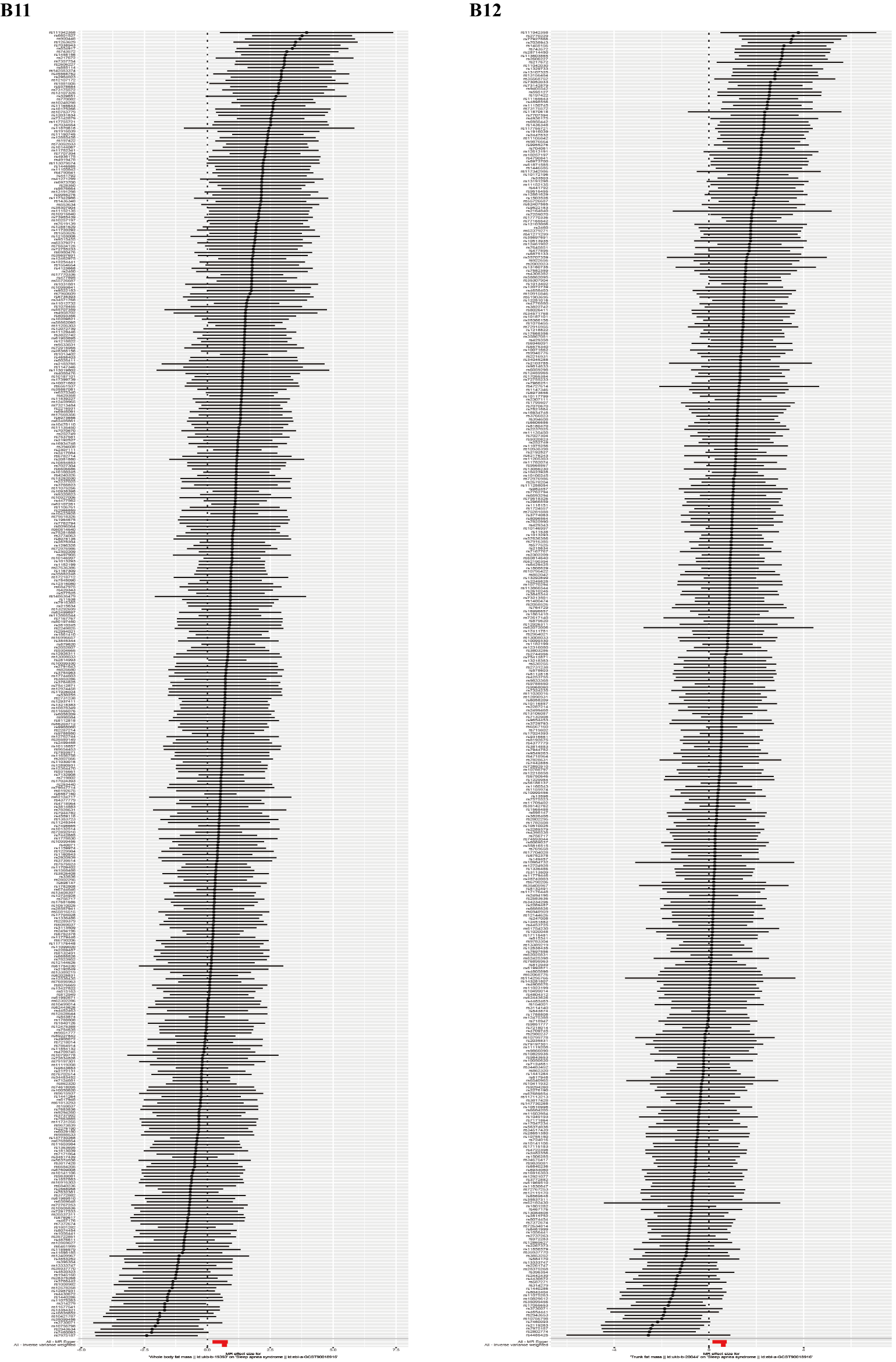


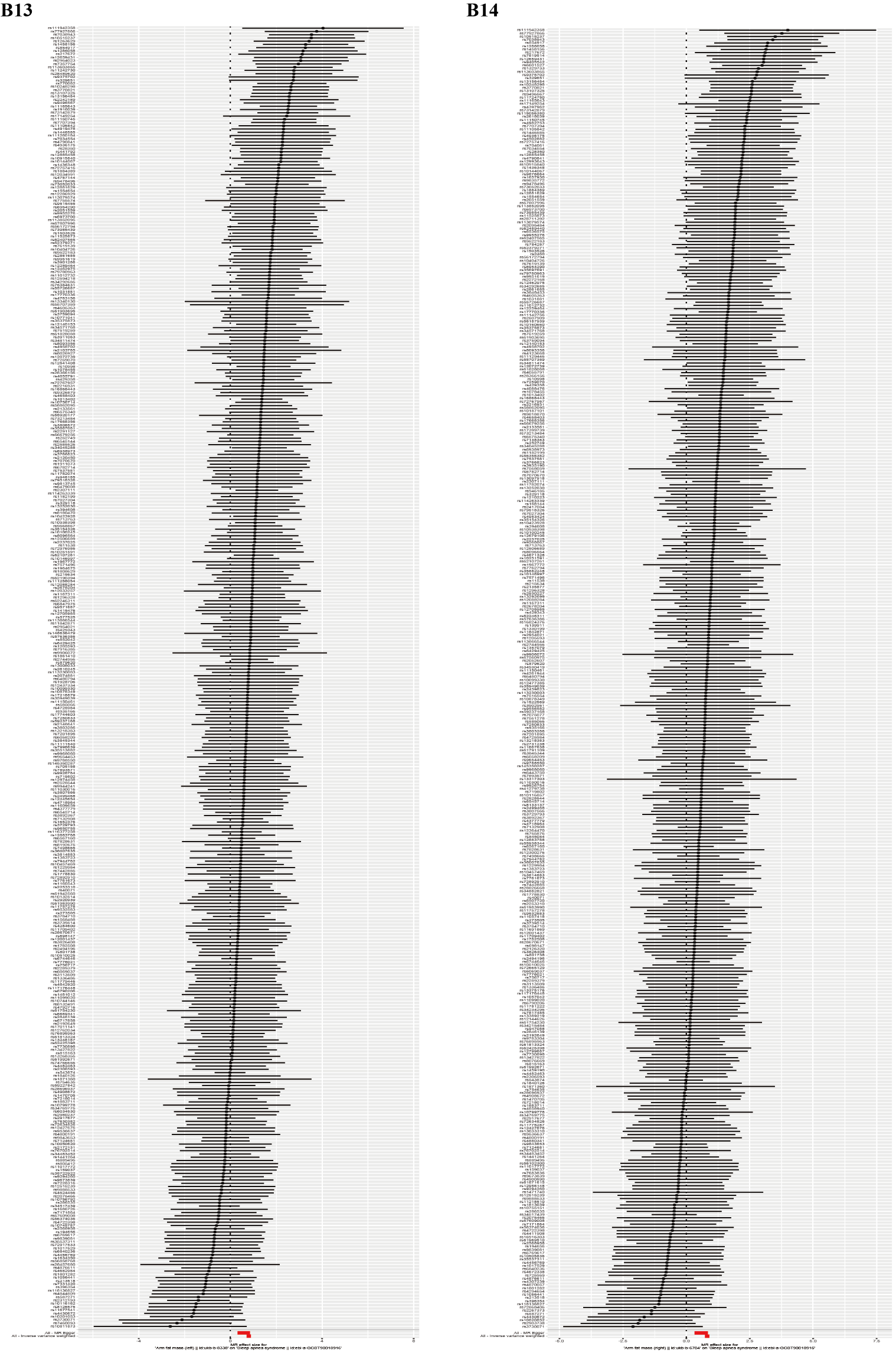


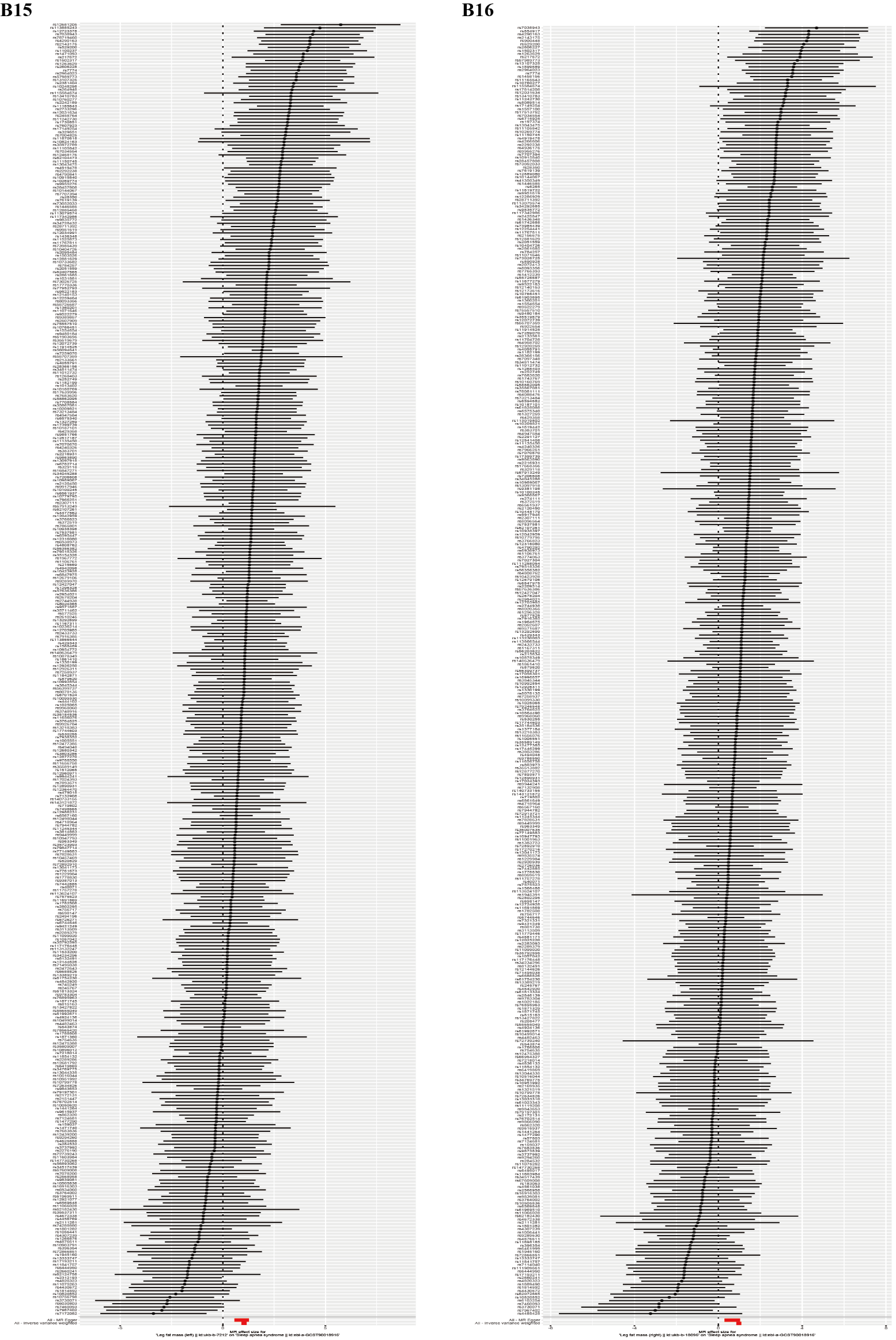


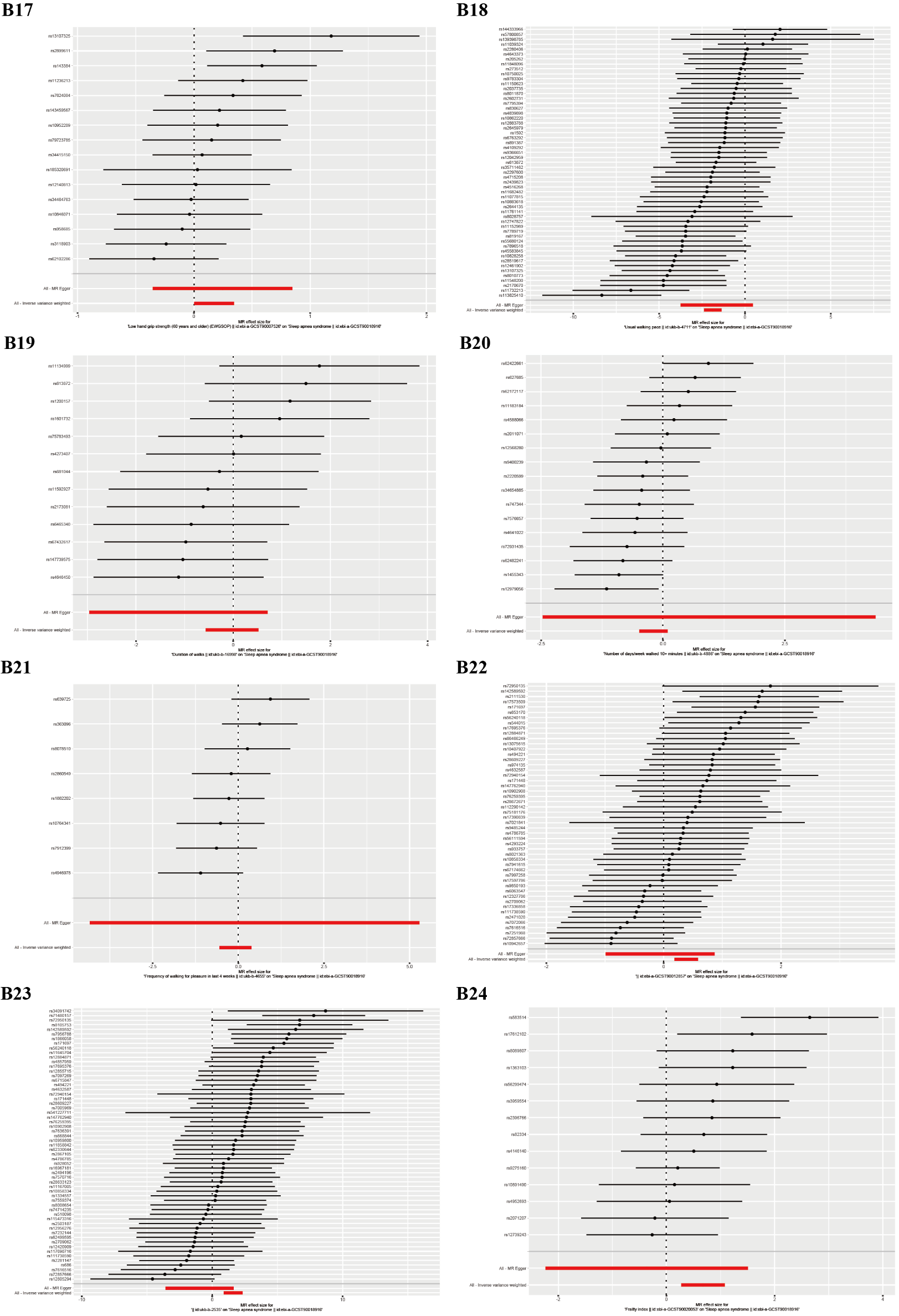


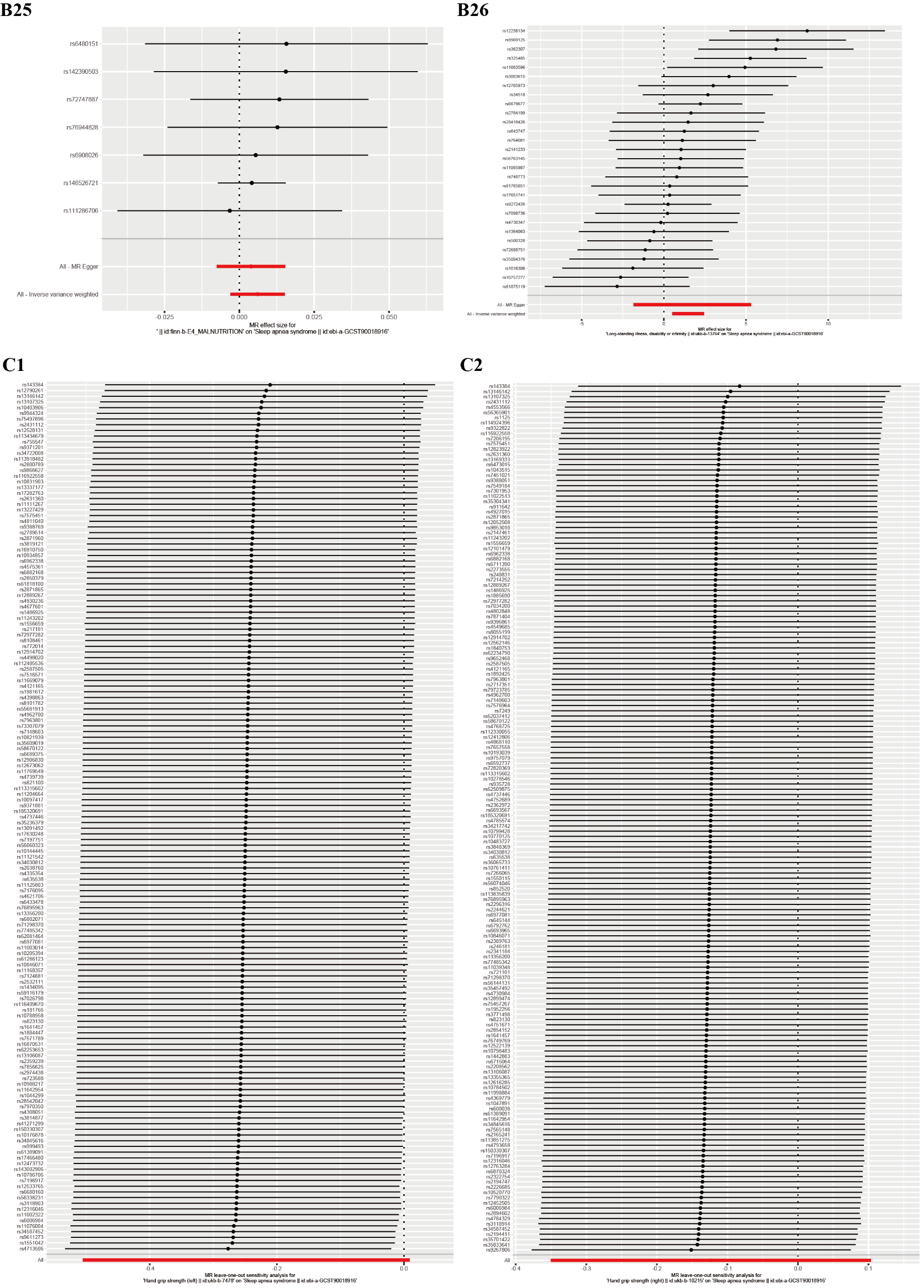


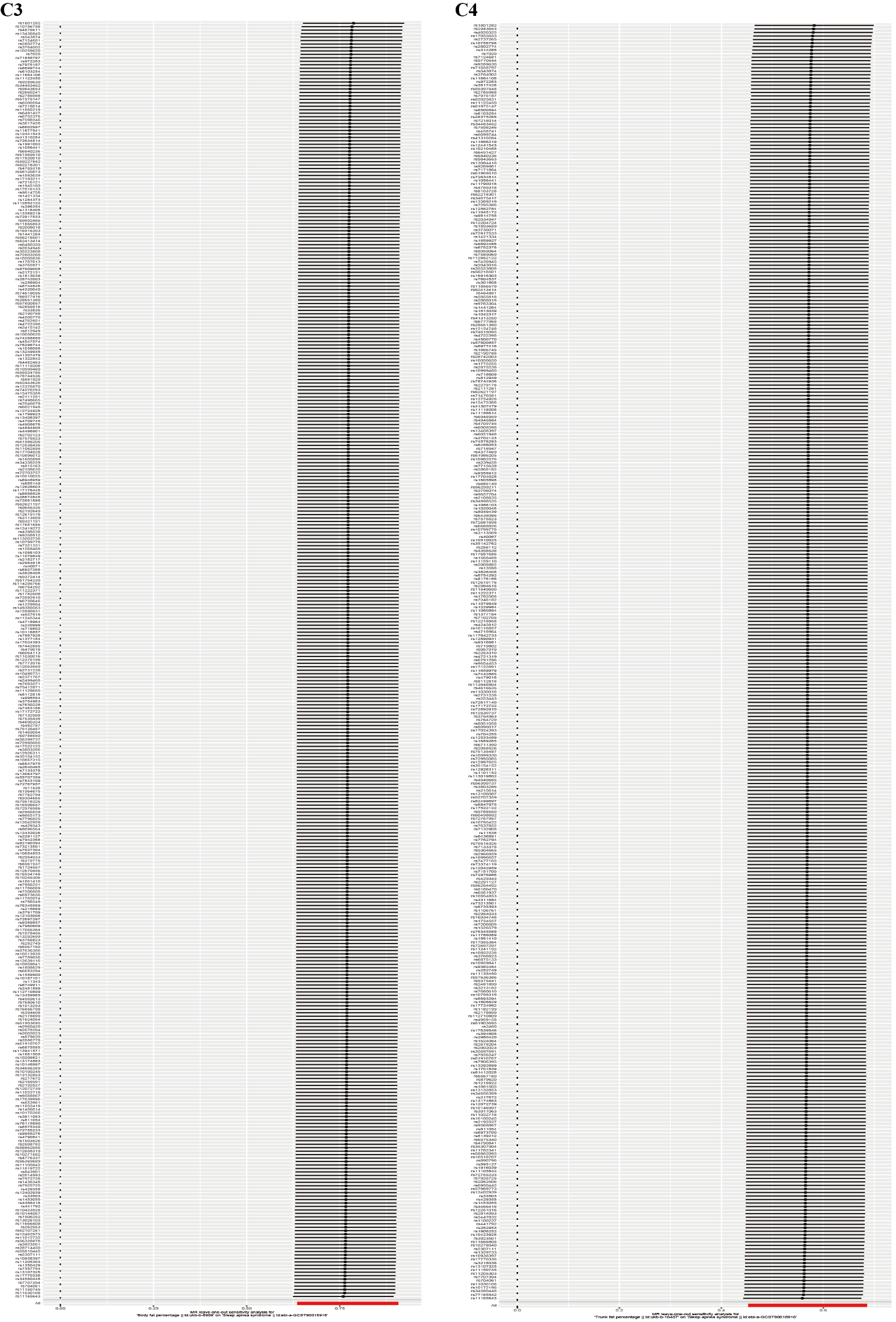


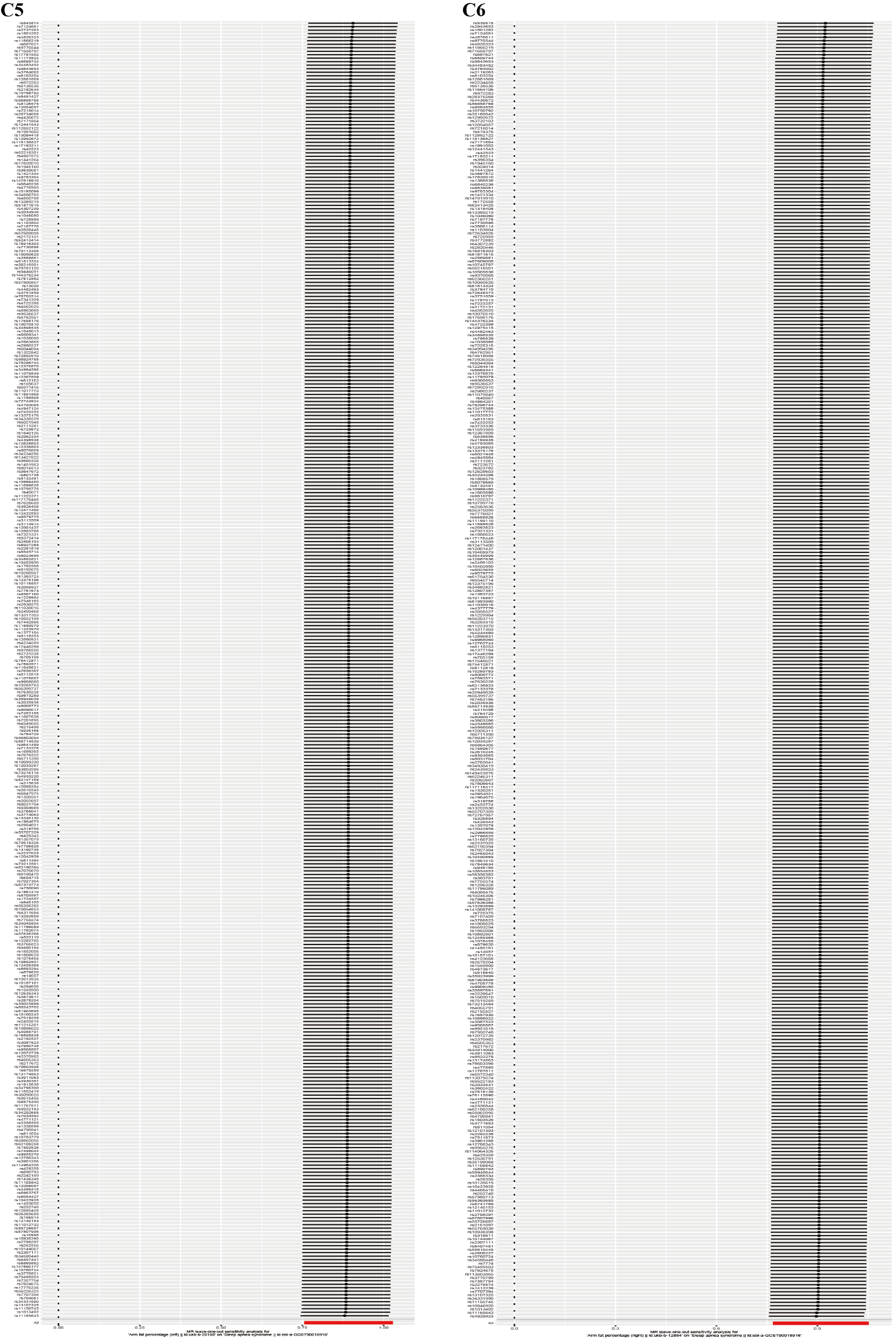


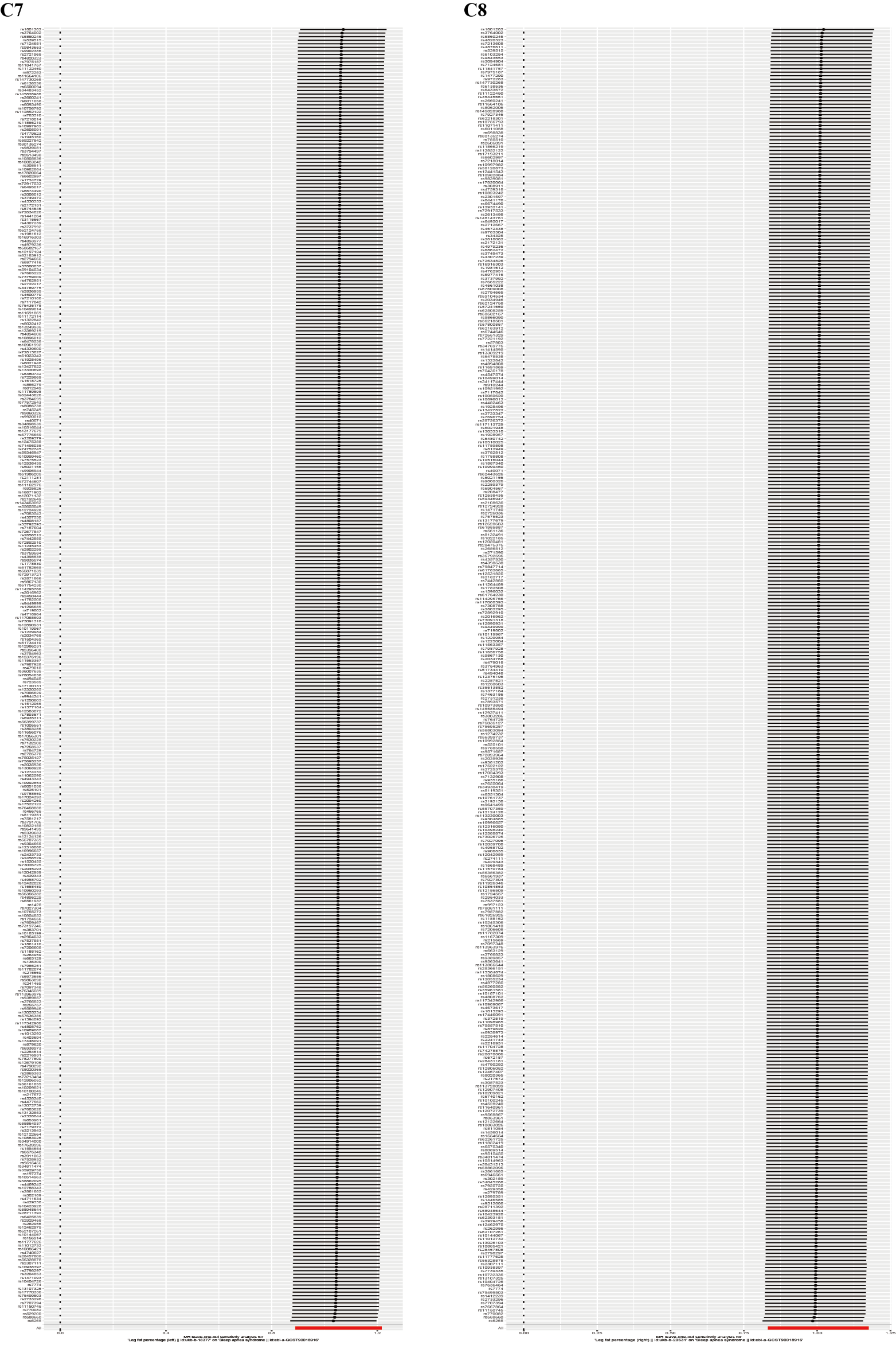


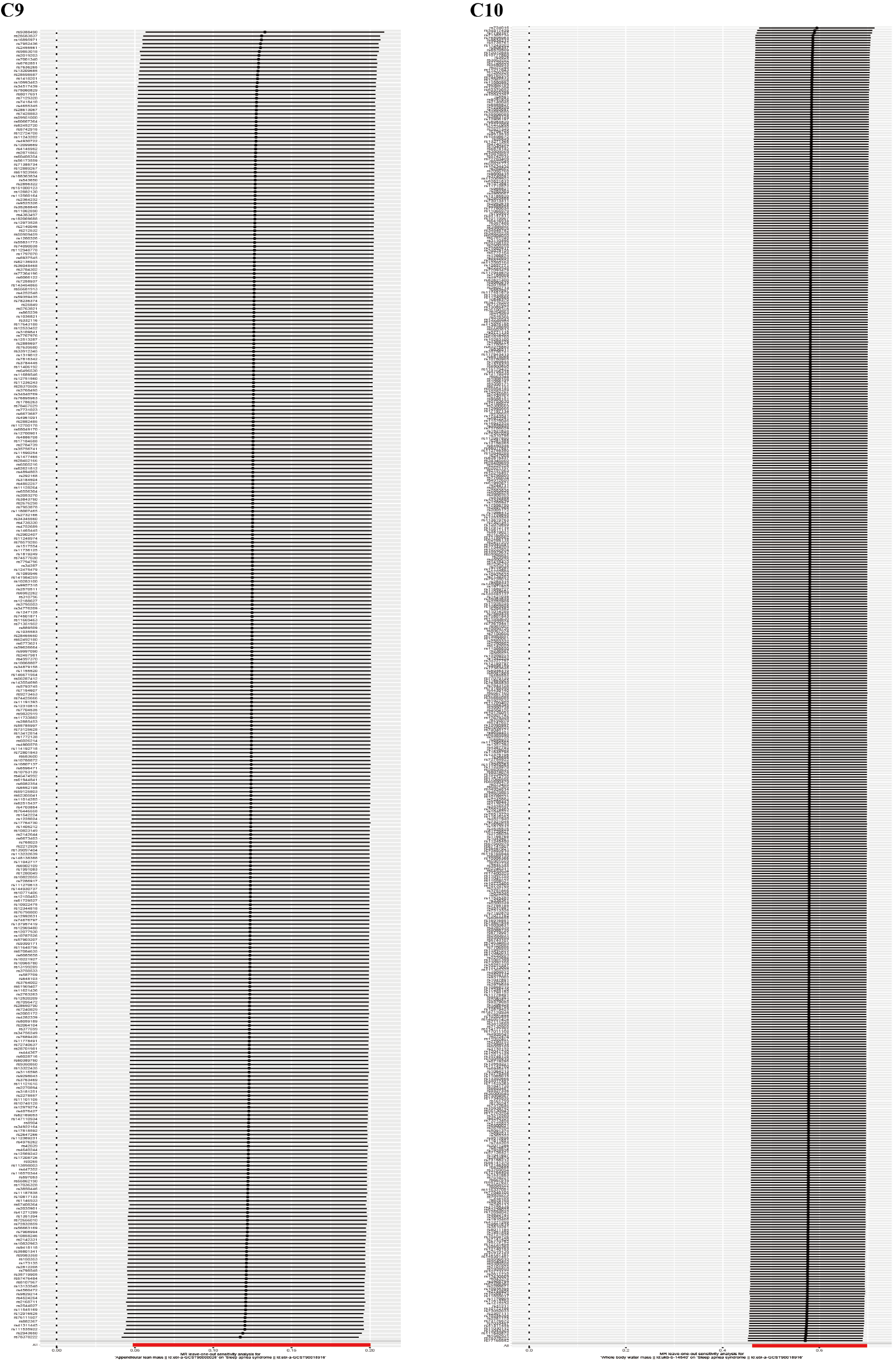


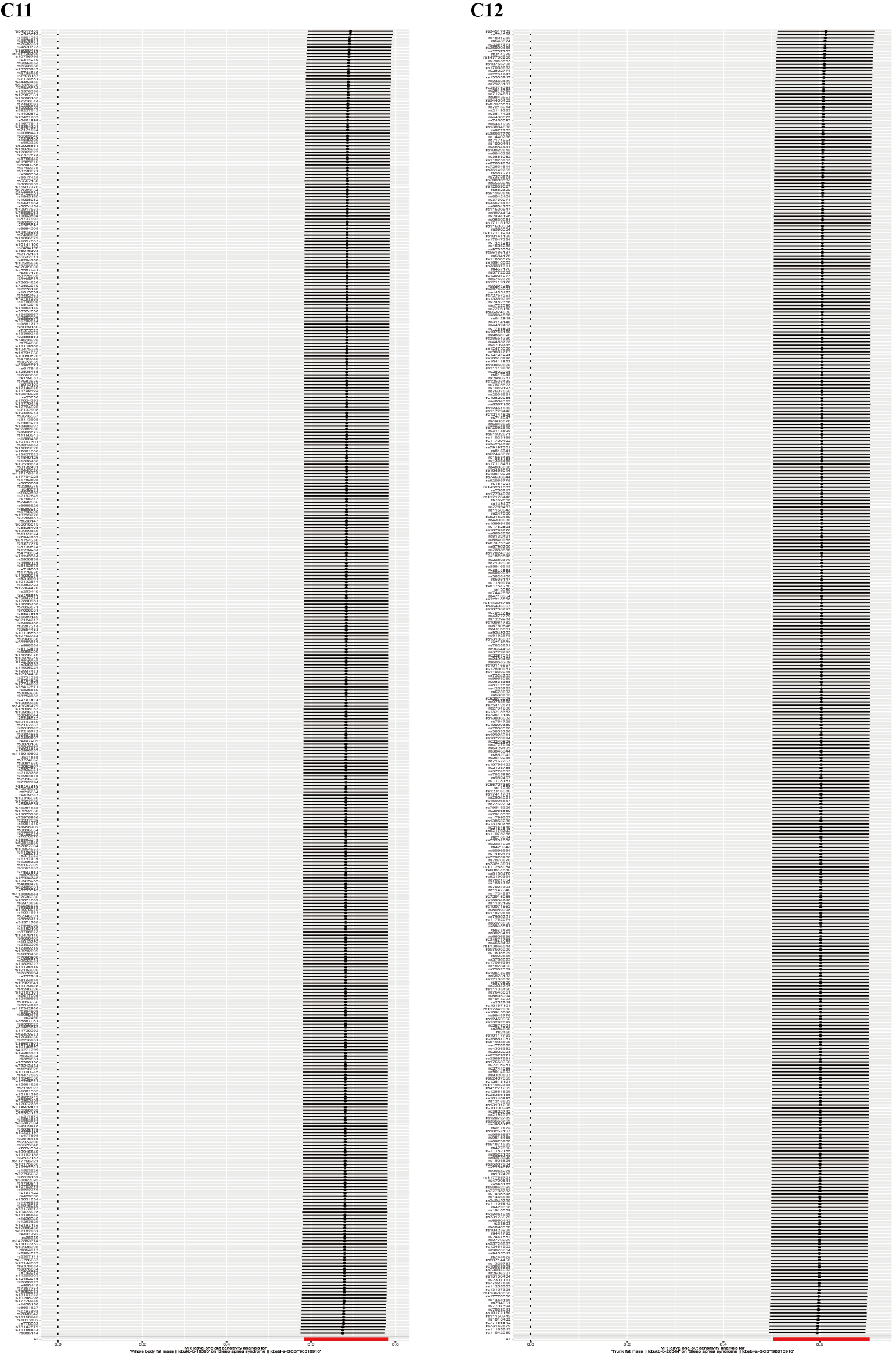


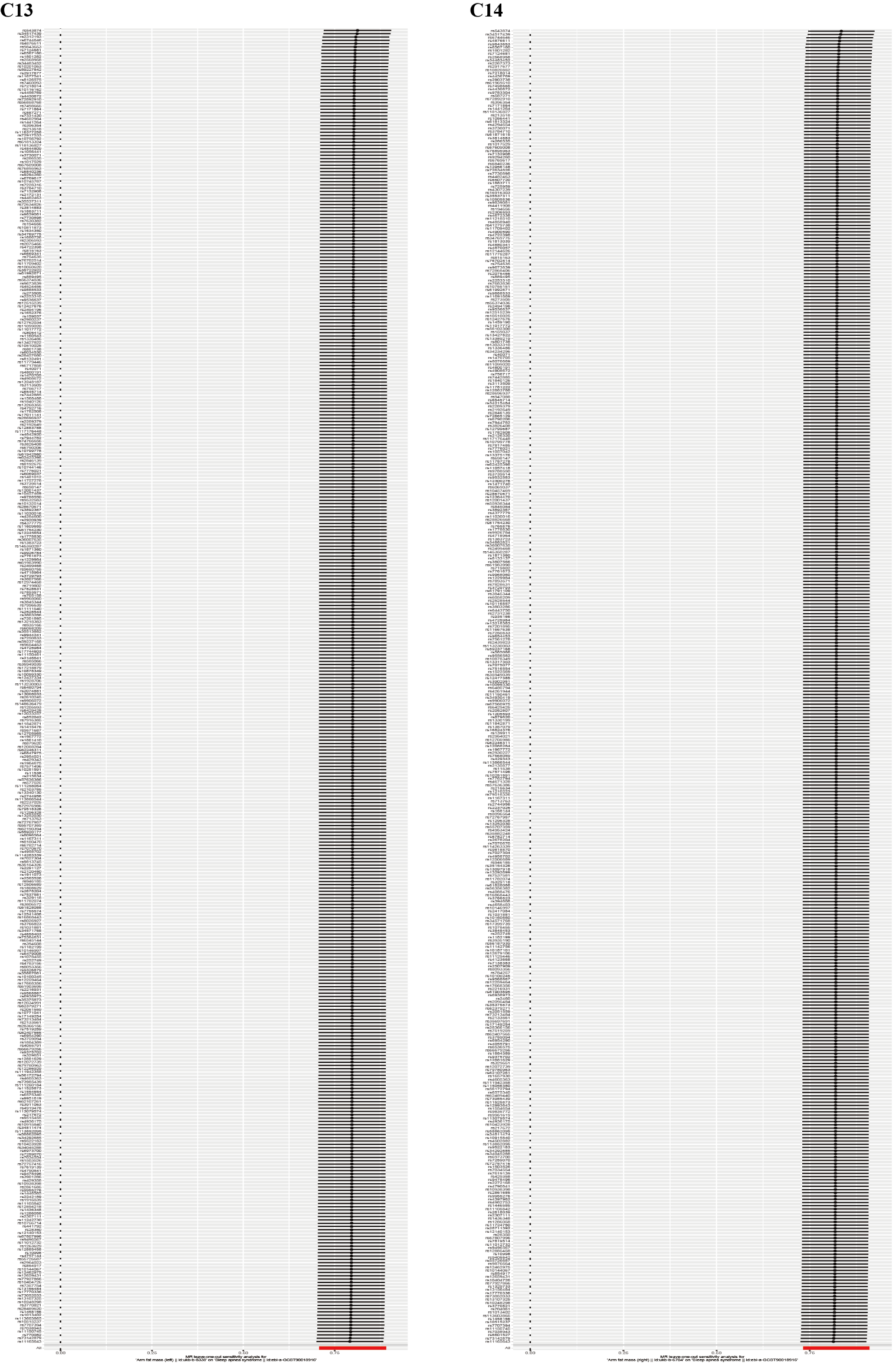


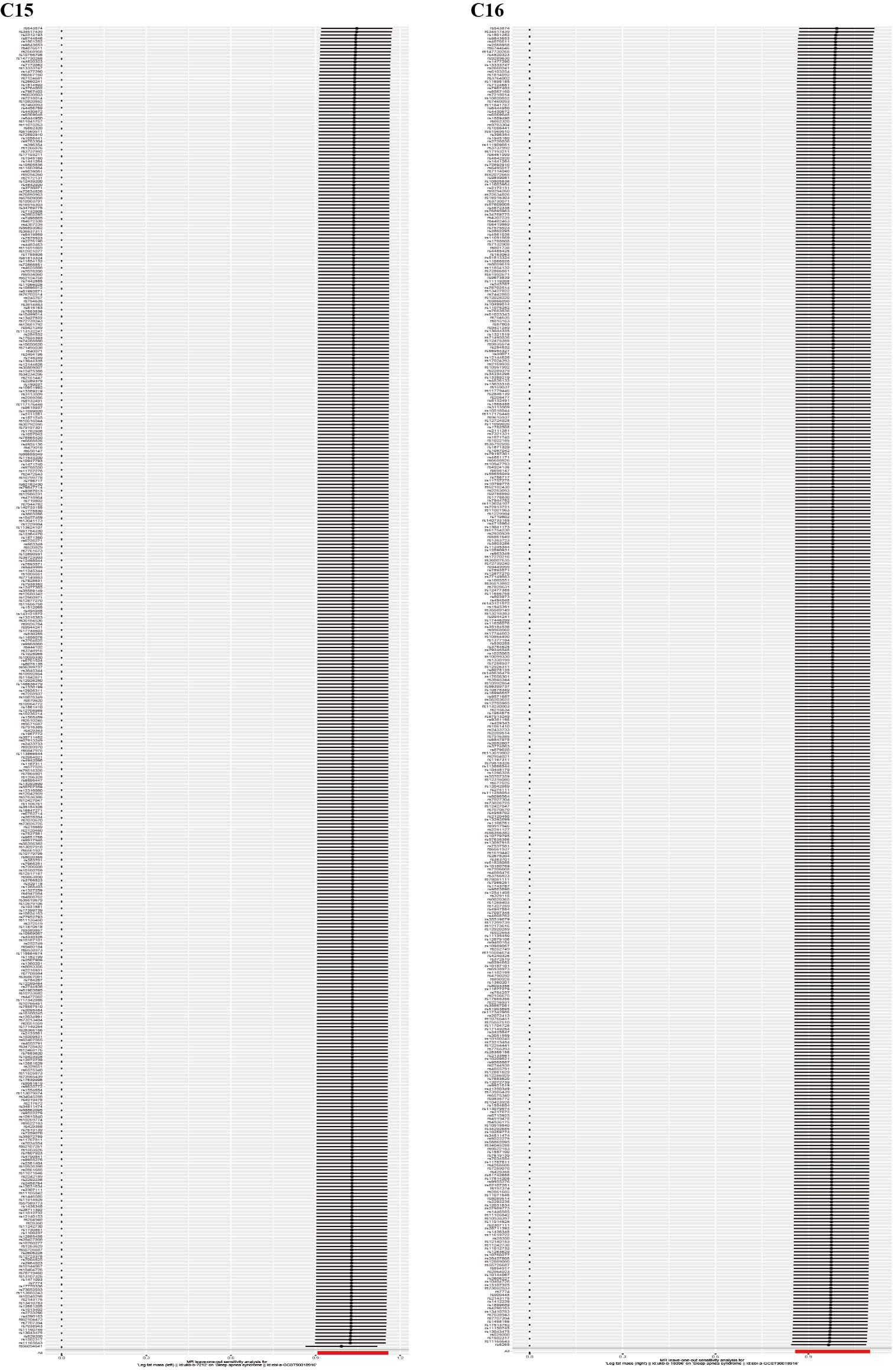


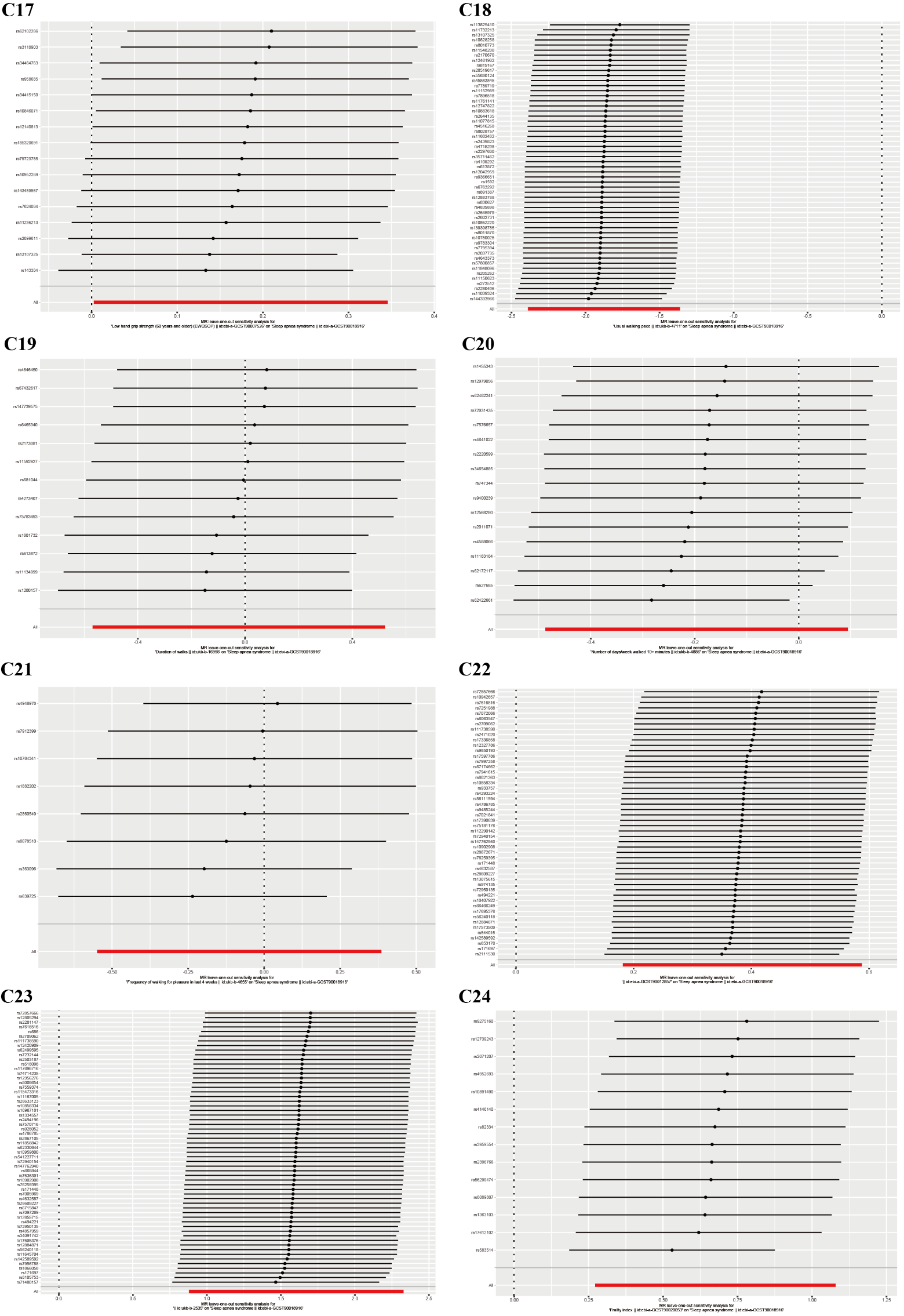


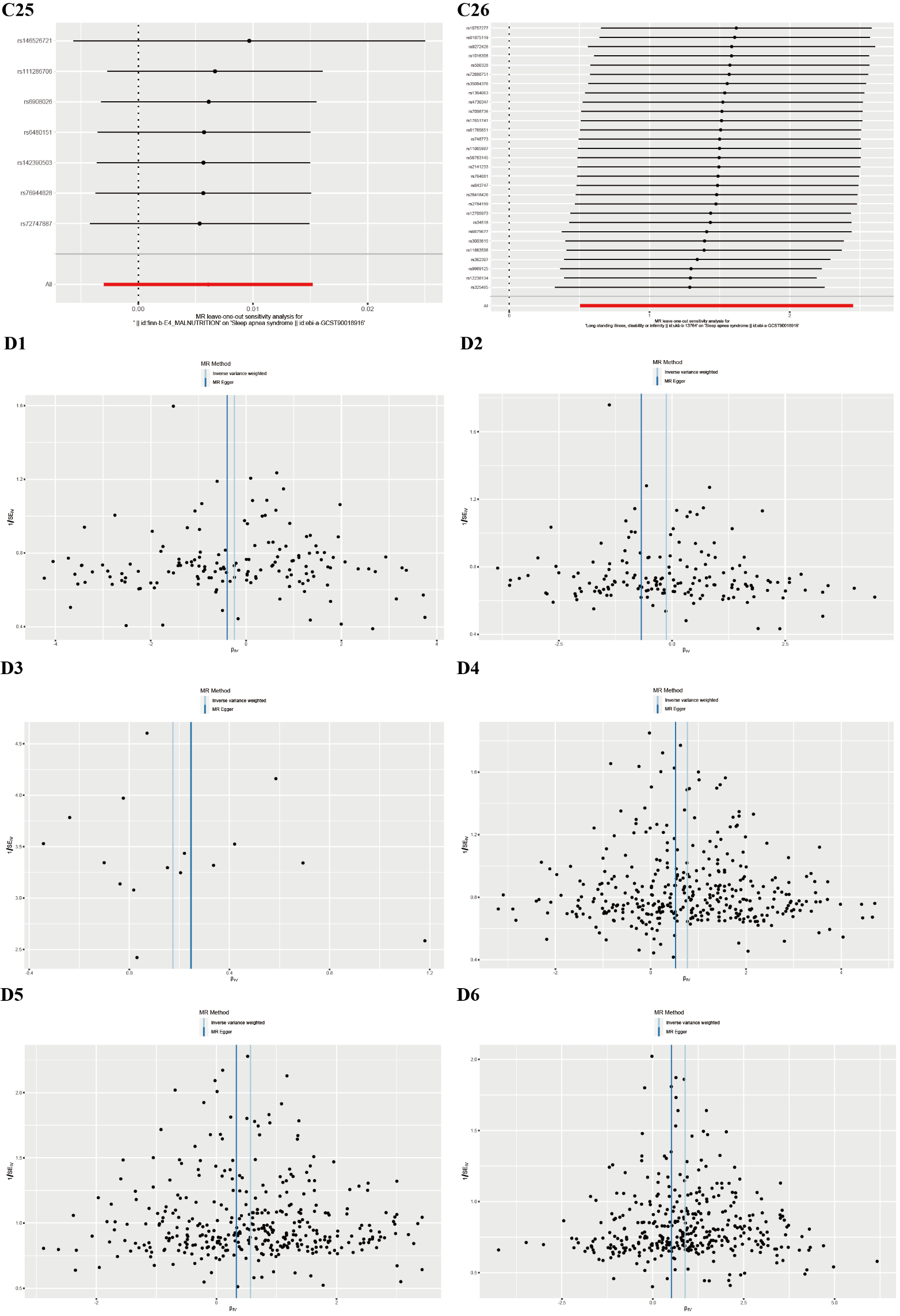


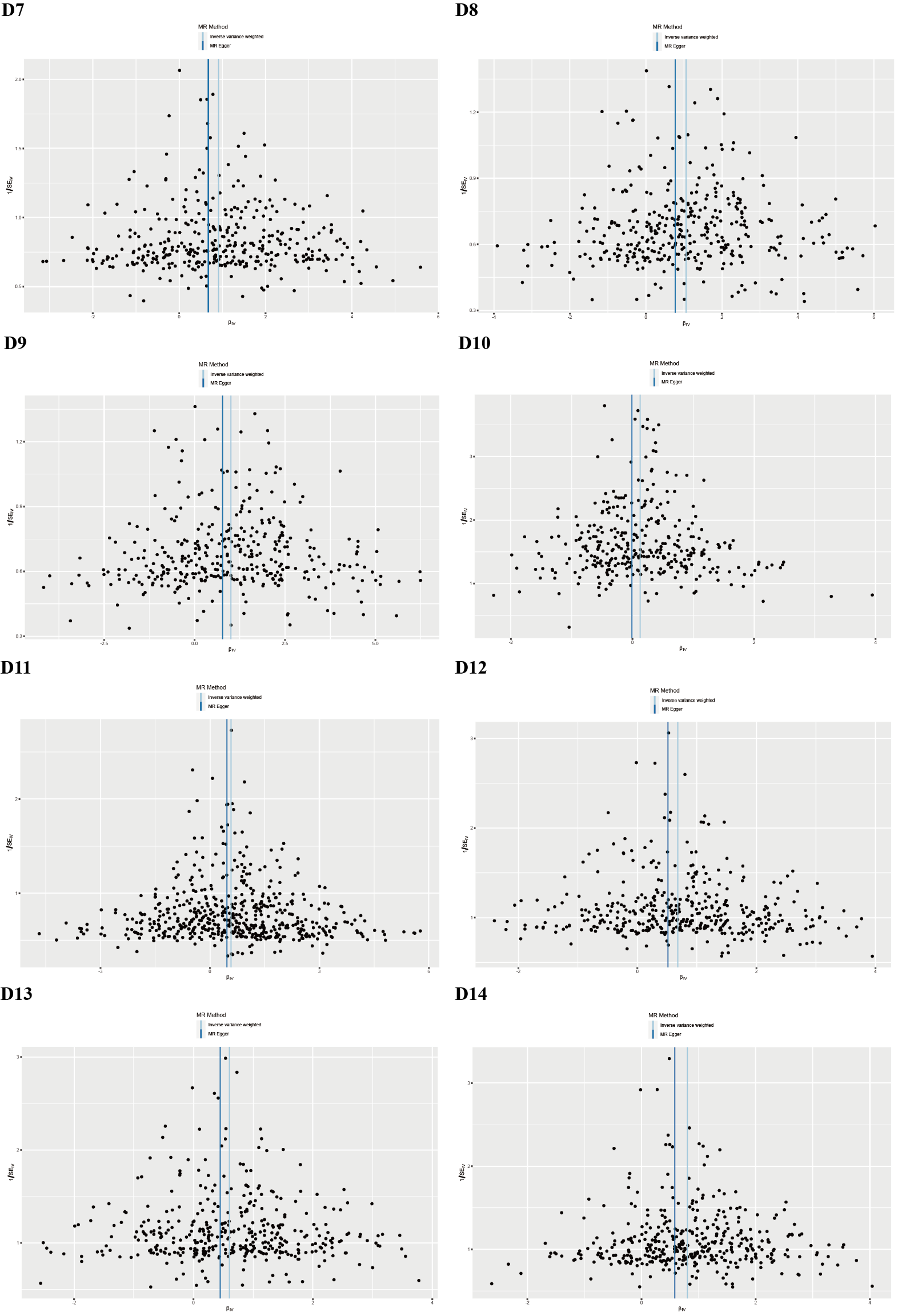


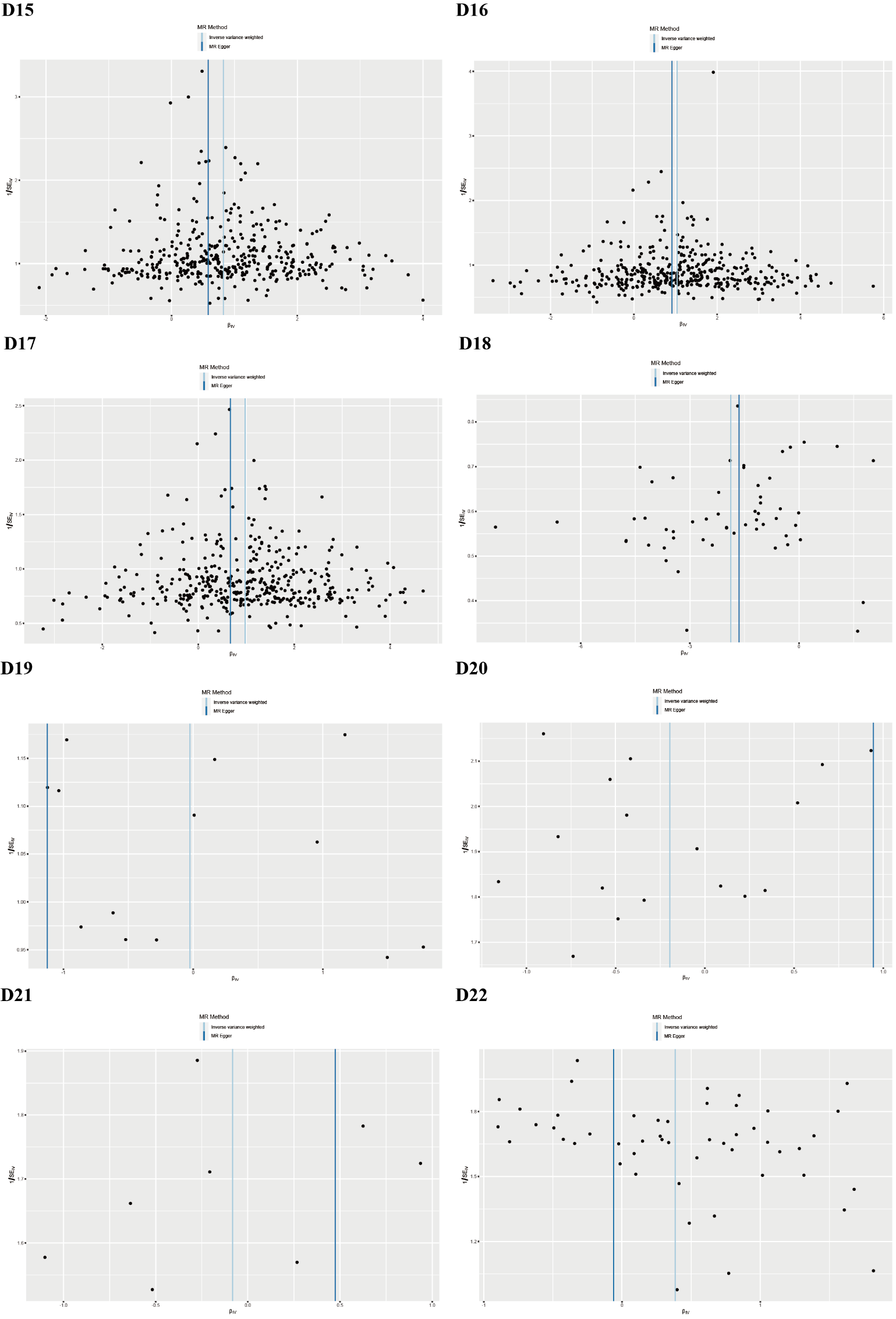


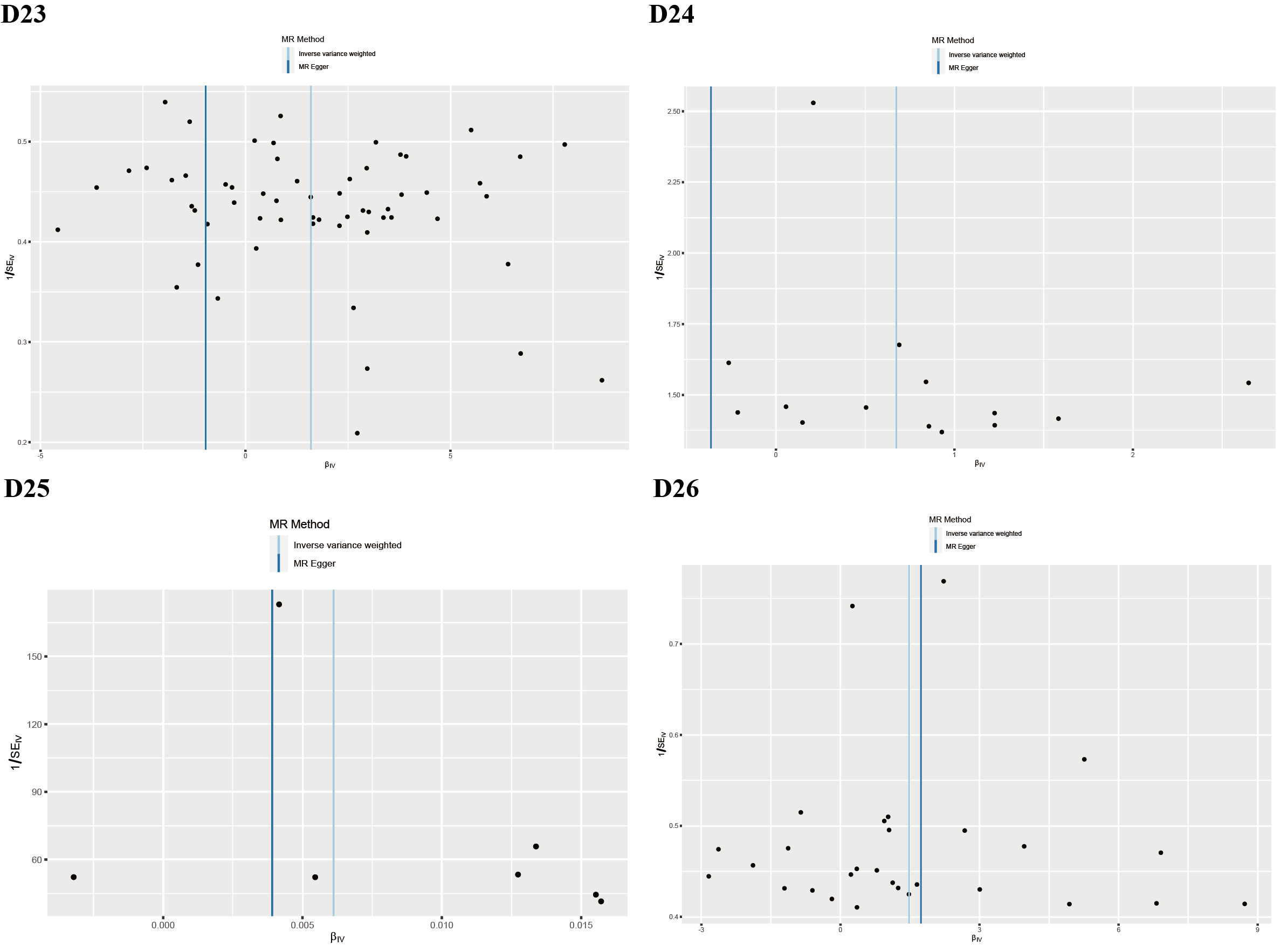


**Supplementary Tables:**

Supplementary Table 1: Details of the subjects included in our study

| **Exposure or Outcome** | | **GWAS ID** | **SNPs** | **Consortium** | **Total Population（Cases/Controls）** | **Ethnicity** | **Year of publication** | **PubMed ID** |
| --- | --- | --- | --- | --- | --- | --- | --- | --- |
| Hand grip strength | Hand grip strength (left) | ukb-b-7478 | 9851867 | MRC-IEU | 461026 | European | 2018 | - |
|  | Hand grip strength (right) | ukb-b-10215 | 9851867 | MRC-IEU | 461089 | European | 2018 | - |
|  | Low hand grip strength (60 years and older) (EWGSOP) | ebi-a-GCST90007526 | 9336415 | NA | 256523(48596/207927) | European | 2021 | 33510174 |
| Muscle mass, fat mass, water mass | Body fat percentage | ukb-b-8909 | 9851867 | MRC-IEU | 454633 | European | 2018 | - |
|  | Trunk fat percentage | ukb-b-16407 | 9851867 | MRC-IEU | 454613 | European | 2018 | - |
|  | Arm fat percentage (left) | ukb-b-20188 | 9851867 | MRC-IEU | 454724 | European | 2018 | - |
|  | Arm fat percentage (right) | ukb-b-12854 | 9851867 | MRC-IEU | 454789 | European | 2018 | - |
|  | Leg fat percentage (left) | ukb-b-18377 | 9851867 | MRC-IEU | 454826 | European | 2018 | - |
|  | Leg fat percentage (right) | ukb-b-20531 | 9851867 | MRC-IEU | 454854 | European | 2018 | - |
|  | Appendicular lean mass (ALM) | ebi-a-GCST90000026 | 18164071 | NA | 205513 | European | 2020 | 33097823 |
|  | Whole body water mass | ukb-b-14540 | 9851867 | MRC-IEU | 454888 | European | 2018 | - |
|  | Whole body fat mass | ukb-b-19393 | 9851867 | MRC-IEU | 454137 | European | 2018 | - |
|  | Trunk fat mass | ukb-b-20044 | 9851867 | MRC-IEU | 454588 | European | 2018 | - |
|  | Arm fat mass (left) | ukb-b-8338 | 9851867 | MRC-IEU | 454684 | European | 2018 | - |
|  | Arm fat mass (right) | ukb-b-6704 | 9851867 | MRC-IEU | 454757 | European | 2018 | - |
|  | Leg fat mass (left) | ukb-b-7212 | 9851867 | MRC-IEU | 454823 | European | 2018 | - |
|  | Leg fat mass (right) | ukb-b-18096 | 9851867 | MRC-IEU | 454846 | European | 2018 | - |
| Physical performance | Usual walking pace | ukb-b-4711 | 9851867 | MRC-IEU | 459915 | European | 2018 | - |
|  | Number of days/week walked 10+ minutes | ukb-b-4886 | 9851867 | MRC-IEU | 454783 | European | 2018 | - |
|  | Frequency of walking for pleasure in last 4 weeks | ukb-b-4655 | 9851867 | MRC-IEU | 328320 | European | 2018 | - |
|  | Duration of walks | ukb-b-16998 | 9851867 | MRC-IEU | 395831 | European | 2018 | - |
|  | Falling risk | ebi-a-GCST90012857 | 7720247 | NA | 451179(89076/362103) | European | 2020 | 32999390 |
|  | Falls in the last year | ukb-b-2535 | 9851867 | MRC-IEU | 461725 | European | 2018 | - |
|  | Frailty index | ebi-a-GCST90020053 | 7589717 | NA | 175226 | European | 2021 | 34431594 |
|  | Malnutrition | finn-b-E4_MALNUTRITION | 16380466 | NA | 218792（180/218612） | European | 2021 | - |
|  | Long-standing illness, disability or infirmity | ukb-b-13764 | 9851867 | MRC-IEU | 451893(147104/304789) | European | 2018 | - |
| obstructive sleep apnea（OSA） | | ebi-a-GCST90018916 | 24183940 | NA | 476853(13818/463035) | European | 2021 | 34594039 |

Supplementary Table 2: Detailed information on SNPs filtrations in each step

| **SNPs** | **trait** |
| --- | --- |
| rs35175534 | Hand grip strength (left)/Hand grip strength (right) |
| rs12790261 | Hand grip strength (right) |
| rs11619393 | Body fat percentage/Trunk fat percentage/Leg fat percentage (left)/Leg fat percentage (right)/Trunk fat mass |
| rs2276936 | Body fat percentage |
| rs2943653 | Body fat percentage/Arm fat percentage (left) |
| rs2957678 | Body fat percentage |
| rs4820323 | Body fat percentage |
| rs529200 | Body fat percentage/Trunk fat percentage/Arm fat percentage (right)/Long-standing illness, disability or infirmity |
| rs56094641 | Body fat percentage/Trunk fat percentage/Arm fat percentage (left)/Arm fat percentage (right)/Whole body water mass/Whole body fat mass/Trunk fat mass/Arm fat mass (left)/Arm fat mass (right)/Leg fat mass (right) |
| rs885114 | Body fat percentage/Trunk fat percentage |
| rs2923115 | Trunk fat percentage |
| rs9859077 | Trunk fat percentage |
| rs1023229 | Arm fat percentage (left)/Arm fat percentage (right) |
| rs17055847 | Arm fat percentage (left)/Arm fat percentage (right) |
| rs67257872 | Arm fat percentage (left)/Leg fat percentage (left) |
| rs7697886 | Arm fat percentage (left) |
| rs57400569 | Arm fat percentage (right) |
| rs28594633 | Arm fat percentage (right) |
| rs62048402 | Leg fat percentage (left)/Usual walking pace |
| rs12635614 | Leg fat percentage (right) |
| rs1421085 | Leg fat percentage (right)/Appendicular lean mass (ALM) |
| rs115912456 | Appendicular lean mass (ALM) |
| rs12419948 | Appendicular lean mass (ALM) |
| rs143384 | Appendicular lean mass (ALM) /Whole body water mass |
| rs112544217 | Whole body water mass |
| rs118173451 | Whole body water mass |
| rs12820906 | Whole body water mass |
| rs61939893 | Whole body water mass |
| rs6235 | Whole body water mass |
| rs7671110 | Whole body water mass |
| rs7952436 | Whole body water mass |
| rs8002779 | Whole body water mass |
| rs1350506 | Whole body fat mass |
| rs17781552 | Whole body fat mass |
| rs1874832 | Whole body fat mass |
| rs61779305 | Whole body fat mass |
| rs34580448 | Trunk fat mass |
| rs4929923 | Arm fat mass (left) |
| rs6601451 | Arm fat mass (left) |
| rs10756798 | Arm fat mass (right)/Leg fat mass (right) |
| rs11042030 | Arm fat mass (right) |
| rs2312193 | Arm fat mass (right) |
| rs11250094 | Leg fat mass (right) |
| rs13099083 | Leg fat mass (right) |
| rs11881338 | Usual walking pace |
| rs11030084 | Falling risk |

Supplementary Table 3: Outliers eliminated instrumental variables (IVs)

| **Exposures** | ***p*-values** | **SNPs** | **Step 1** | **Step 2** | **Step 3** |
| --- | --- | --- | --- | --- | --- |
| **Hand grip strength** |  | | | | |
| Hand grip strength (left) | 5.00E-08 | 157 | 157 | 154 | 153 |
| Hand grip strength (right) | 5.00E-08 | 176 | 176 | 168 | 166 |
| Low hand grip strength (60 years and older) (EWGSOP) | 5.00E-08 | 17 | 17 | 16 | 16 |
| **Muscle mass, fat mass, water mass** |  | | | | |
| Body fat percentage | 5.00E-08 | 395 | 398 | 377 | 369 |
| Trunk fat percentage | 5.00E-08 | 389 | 391 | 368 | 362 |
| Arm fat percentage (left) | 5.00E-08 | 394 | 396 | 376 | 370 |
| Arm fat percentage (right) | 5.00E-08 | 394 | 395 | 376 | 370 |
| Leg fat percentage (left) | 5.00E-08 | 383 | 384 | 364 | 361 |
| Leg fat percentage (right) | 5.00E-08 | 385 | 387 | 367 | 364 |
| Appendicular lean mass (ALM) | 5.00E-08 | 365 | 352 | 340 | 336 |
| Whole body water mass | 5.00E-08 | 565 | 566 | 542 | 532 |
| Whole body fat mass | 5.00E-08 | 435 | 438 | 415 | 410 |
| Trunk fat mass | 5.00E-08 | 422 | 424 | 398 | 395 |
| Arm fat mass (left) | 5.00E-08 | 421 | 421 | 402 | 400 |
| Arm fat mass (right) | 5.00E-08 | 428 | 429 | 410 | 406 |
| Leg fat mass (left) | 5.00E-08 | 424 | 425 | 397 | 397 |
| Leg fat mass (right) | 5.00E-08 | 427 | 429 | 401 | 397 |
| **Physical performance** |  | | | | |
| Usual walking pace | 5.00E-08 | 57 | 57 | 56 | 55 |
| Duration of walks | 5.00E-08 | 15 | 16 | 13 | 13 |
| Number of days/week walked 10+ minutes | 5.00E-08 | 17 | 17 | 17 | 17 |
| Frequency of walking for pleasure in last 4 weeks | 5.00E-08 | 8 | 8 | 8 | 8 |
| Falling risk | 5.00E-06 | 53 | 53 | 51 | 50 |
| Falls in the last year | 5.00E-06 | 59 | 59 | 59 | 59 |
| Frailty index | 5.00E-08 | 15 | 14 | 14 | 14 |
| Malnutrition | 5.00E-06 | 7 | 7 | 7 | 7 |
| Long-standing illness, disability or infirmity | 5.00E-08 | 30 | 30 | 30 | 29 |
| *p*-values: the *p* values set for genetic variants closely associated with exposures | | | | | |
| Step 1: clumping the SNPs (linkage disequilibrium (LD) r^2^ = 0.001, kb = 10000) and excluding the SNPs associated with outcomes (*p* < 5E-8) or (*p* < 5E-6) | | | | | |
| Step 2: excluding the SNPs in the harmonization procedures | | | | | |
| Step 3: excluding the SNPs with potential pleiotropy by MR-PRESSO | | | | | |

Supplementary Table 4: Results of risk & protective factor MR analysis

| **Exposures** | **Inverse variance weighted** | | | **MR-Egger** | | | **Weighted median** | | | **Directional pleiotropy** | | **Cochran's Q** | |
| --- | --- | --- | --- | --- | --- | --- | --- | --- | --- | --- | --- | --- | --- |
|  | *p*-value | adjusted *p*-value | OR (95%CI) | *p*-value | adjusted *p*-value | OR (95%CI) | *p*-value | adjusted *p*-value | OR (95%CI) | MR-Egger intercept | *p*-value | Q statistic | *p*-value |
| Hand grip strength (left) | 0.06215694 | 0.05864197 | 0.780（0.603-1.009） | 0.44158446 | 0.42730632 | 0.674（0.255-1.781） | 0.95540691 | 0.95290642 | 1.010（0.729-1.399） | 0.00178790 | 0.75963135 | 233.20602563 | 2.51E-05 |
| Hand grip strength (right) | 0.19995032 | 0.28706128 | 0.884（0.704-1.109） | 0.04332022 | 0.11634740 | 0.507（0.218-1.178） | 0.28247402 | 0.39849049 | 0.879（0.651-1.186） | 0.00681472 | 0.18172420 | 218.69028468 | 0.00325849 |
| Low hand grip strength (60 years and older) (EWGSOP) | 0.04662149 | **0.04662149** | 1.190（1.003-1.413） | 0.43384822 | 0.43384822 | 1.280（0.702-2.333） | 0.38996761 | 0.38996761 | 1.095（0.891-1.345） | -0.00416726 | 0.80737571 | 22.03617115 | 0.10685897 |
| Body fat percentage | 5.05E-28 | **9.01E-29** | 2.163（1.888-2.478） | 0.00014504 | **0.02948360** | 1.681（1.055-2.679） | 1.93E-15 | **2.37E-14** | 2.032（1.693-2.437） | 0.00351821 | 0.26868677 | 508.94091837 | 1.48E-06 |
| Trunk fat percentage | 4.02E-23 | **1.19E-21** | 1.767（1.572-1.986） | 0.00083143 | 0.09705998 | 1.393（0.943-2.058） | 1.03E-14 | **1.62E-11** | 1.731（1.475-2.030） | 0.00403202 | 0.21140341 | 520.24840991 | 7.54E-08 |
| Arm fat percentage (left) | 1.96E-37 | **1.44E-37** | 2.440（2.129-2.797） | 2.88E-05 | **0.02495332** | 1.670（1.069-2.611） | 3.34E-19 | **2.20E-17** | 2.286（1.889-2.767） | 0.00520027 | 0.08148958 | 502.69575506 | 4.26E-06 |
| Arm fat percentage (right) | 1.68E-37 | **5.48E-36** | 2.484（2.155-2.864） | 5.26E-06 | **0.00461628** | 1.964（1.235-3.124） | 7.58E-18 | **1.65E-16** | 2.196（1.821-2.648） | 0.00323604 | 0.29777680 | 552.12304158 | 1.92E-09 |
| Leg fat percentage (left) | 1.43E-40 | **2.54E-36** | 2.866（2.433-3.377） | 3.53E-06 | **0.01227282** | 2.144（1.184-3.883） | 1.48E-18 | **3.88E-16** | 2.568（2.046-3.222） | 0.00319756 | 0.31971590 | 444.26062486 | 0.00159696 |
| Leg fat percentage (right) | 3.84E-34 | **2.80E-30** | 2.729（2.297-3.241） | 1.40E-05 | **0.01372566** | 2.164（1.175-3.987） | 3.57E-20 | **1.48E-16** | 2.601（2.073-3.263） | 0.00259684 | 0.43883259 | 510.33314522 | 5.05E-07 |
| Appendicular lean mass (ALM) | 0.00333375 | **0.00130730** | 1.133（1.050-1.222） | 0.34740765 | 0.93758192 | 0.992（0.805-1.222） | 0.05817321 | **0.00590298** | 1.153（1.042-1.275） | 0.00389560 | 0.18131590 | 505.84540071 | 4.43E-09 |
| Whole body water mass | 2.73E-18 | **9.70E-22** | 1.786（1.586-2.011） | 0.00304845 | **0.00170346** | 1.598（1.194-2.138） | 1.17E-11 | **1.02E-10** | 1.800（1.506-2.151） | 0.00159439 | 0.41223294 | 742.56953686 | 3.17E-09 |
| Whole body fat mass | 1.37E-42 | **1.48E-40** | 1.981（1.791-2.190） | 1.67E-07 | **0.00106469** | 1.669（1.231-2.263） | 3.90E-22 | **4.49E-17** | 1.840（1.596-2.122） | 0.00313891 | 0.24421703 | 586.70746792 | 1.77E-08 |
| Trunk fat mass | 9.40E-36 | **5.47E-32** | 1.828（1.653-2.021） | 3.59E-06 | **0.00405375** | 1.560（1.154-2.109） | 1.13E-16 | **9.78E-15** | 1.711（1.494-1.960） | 0.00303622 | 0.27621473 | 587.02848706 | 8.87E-10 |
| Arm fat mass (left) | 4.17E-72 | **3.53E-65** | 2.225（2.029-2.439） | 9.45E-10 | **3.76E-05** | 1.791（1.362-2.356） | 3.17E-31 | **2.86E-27** | 2.056（1.805-2.343） | 0.00402503 | 0.10071905 | 474.72303882 | 0.00537306 |
| Arm fat mass (right) | 1.91E-75 | **3.67E-73** | 2.274（2.080-2.485） | 5.11E-10 | **1.63E-05** | 1.795（1.380-2.335） | 3.27E-30 | **4.32E-29** | 2.079（1.829-2.363） | 0.00434120 | 0.06209076 | 453.43778287 | 0.04829100 |
| Leg fat mass (left) | 2.08E-58 | **2.08E-58** | 2.808（2.476-3.183） | 5.84E-07 | **5.84E-07** | 2.504（1.757-3.568） | 3.79E-32 | **3.79E-32** | 2.905（2.433-3.467） | 0.00175487 | 0.49828408 | 579.59094568 | 4.58E-09 |
| Leg fat mass (right) | 1.16E-60 | **1.92E-56** | 2.659（2.356-3.002） | 4.53E-08 | **5.29E-04** | 1.949（1.340-2.833） | 3.86E-38 | **1.13E-31** | 2.779（2.342-3.298） | 0.00464670 | 0.08610485 | 525.80031102 | 1.28E-05 |
| Usual walking pace | 2.05E-11 | **8.24E-13** | 0.153（0.092-0.256） | 0.12730487 | 0.13348380 | 0.194（0.023-1.600） | 3.62E-06 | **7.30E-06** | 0.221（0.114-0.427） | -0.00219895 | 0.82468851 | 72.77751492 | 0.04510438 |
| Number of days/week walked 10+ minutes | 0.18635625 | 0.18635625 | 0.822（0.614-1.100） | 0.59548120 | 0.59548120 | 2.573（0.085-78.298） | 0.02771715 | **0.02771715** | 0.668（0.466-0.957） | -0.02995783 | 0.52075232 | 22.31306577 | 0.13336767 |
| Frequency of walking for pleasure in last 4 weeks | 0.73165349 | 0.73165349 | 0.921（0.577-1.471） | 0.85318775 | 0.85318775 | 1.607（0.013-197.706） | 0.42086472 | 0.42086472 | 0.793（0.450-1.396） | -0.01293942 | 0.82741328 | 9.02790525 | 0.25065877 |
| Duration of walks | 0.93278824 | 0.93278824 | 0.977（0.566-1.685） | 0.25423751 | 0.25423751 | 0.324（0.052-2.030） | 0.47523686 | 0.47523686 | 0.760（0.358-1.614） | 0.01909076 | 0.24391462 | 13.41390347 | 0.33968755 |
| Falling risk | 8.44E-05 | **2.05E-04** | 1.469（1.199-1.800） | 0.87254334 | 0.89711681 | 0.940（0.371-2.384） | 0.01219612 | **0.02030762** | 1.397（1.053-1.852） | 0.01204112 | 0.33996300 | 72.13339063 | 0.01739138 |
| Falls in the last year | 1.45E-05 | **1.45E-05** | 4.972（2.408-10.265） | 0.47350388 | 0.47350388 | 0.381（0.028-5.230） | 0.00804406 | **0.00804406** | 3.468（1.382-8.699） | 0.01877769 | 0.05069456 | 89.49807314 | 0.00497863 |
| Frailty index | 0.00103883 | **0.00103883** | 1.965（1.312-2.942） | 0.70966105 | 0.70966105 | 0.695（0.107-4.516） | 0.03172043 | **0.03172043** | 1.690（1.047-2.729） | 0.02333386 | 0.28697949 | 19.12387681 | 0.11931265 |
| Malnutrition | 0.18888416 | 0.18888416 | 1.006（0.997-1.015） | 0.53409358 | 0.53409358 | 1.004（0.992-1.015） | 0.39045848 | 0.39045848 | 1.005（0.994-1.015） | 0.00897718 | 0.56305059 | 1.04016339 | 0.98404384 |
| Long-standing illness, disability or infirmity | 0.00159539 | **0.00290938** | 4.387（1.657-11.612） | 0.57134116 | 0.35185833 | 5.651（0.157-203.299） | 0.07733289 | 0.08610056 | 2.715（0.868-8.492） | -0.00189444 | 0.88647044 | 47.07589902 | 0.01344832 |

Supplementary Table 5: Information on all instrumental variables (IVs) ultimately used for Hand grip strength (left) in our study

| SNPs | effect_allele.exposure | other_allele.exposure | eaf.exposure | beta.exposure | se.exposure | pval.exposure | R^2^ | F | trait | id.exposure |
| --- | --- | --- | --- | --- | --- | --- | --- | --- | --- | --- |
| rs10097417 | G | A | 0.170904 | -0.0132302 | 0.00197055 | 1.90E-11 | 0.224178962 | 45.077300599 | Hand grip strength (left) | ukb-b-7478 |
| rs10144445 | G | C | 0.350118 | -0.00936876 | 0.00155918 | 1.90E-09 | 0.187945628 | 36.105363092 | Hand grip strength (left) | ukb-b-7478 |
| rs10176878 | C | T | 0.190861 | -0.0129478 | 0.0018962 | 8.60E-12 | 0.230106846 | 46.625519131 | Hand grip strength (left) | ukb-b-7478 |
| rs10205394 | C | G | 0.200728 | -0.0113217 | 0.00185667 | 1.10E-09 | 0.192478899 | 37.183806418 | Hand grip strength (left) | ukb-b-7478 |
| rs10403906 | A | G | 0.476375 | -0.0100323 | 0.00148665 | 1.50E-11 | 0.225956291 | 45.539006409 | Hand grip strength (left) | ukb-b-7478 |
| rs1044299 | T | C | 0.546046 | 0.01401 | 0.00149362 | 6.60E-21 | 0.360609737 | 87.982445661 | Hand grip strength (left) | ukb-b-7478 |
| rs10786706 | T | C | 0.465745 | 0.0100072 | 0.00148741 | 1.70E-11 | 0.224902990 | 45.265129281 | Hand grip strength (left) | ukb-b-7478 |
| rs10788958 | G | C | 0.644753 | 0.0141976 | 0.00156276 | 1.00E-19 | 0.346011646 | 82.536357638 | Hand grip strength (left) | ukb-b-7478 |
| rs10821939 | A | G | 0.573342 | -0.00935422 | 0.00150339 | 4.90E-10 | 0.198826337 | 38.714338700 | Hand grip strength (left) | ukb-b-7478 |
| rs10831903 | T | C | 0.423383 | 0.00928662 | 0.00151154 | 8.10E-10 | 0.194823938 | 37.746445479 | Hand grip strength (left) | ukb-b-7478 |
| rs10846071 | T | C | 0.393672 | -0.016641 | 0.00151685 | 5.30E-28 | 0.435514015 | 120.357613947 | Hand grip strength (left) | ukb-b-7478 |
| rs10934857 | A | G | 0.258913 | 0.00928675 | 0.00170182 | 4.80E-08 | 0.160289554 | 29.778324793 | Hand grip strength (left) | ukb-b-7478 |
| rs10988217 | G | A | 0.603742 | -0.00920657 | 0.00152911 | 1.70E-09 | 0.188560179 | 36.250855667 | Hand grip strength (left) | ukb-b-7478 |
| rs11002322 | T | G | 0.340459 | -0.00998924 | 0.00157255 | 2.10E-10 | 0.205505055 | 40.351154893 | Hand grip strength (left) | ukb-b-7478 |
| rs11003014 | G | A | 0.161184 | 0.011433 | 0.00202143 | 0.000000016 | 0.170164969 | 31.989171587 | Hand grip strength (left) | ukb-b-7478 |
| rs11076004 | A | G | 0.418177 | -0.0115356 | 0.00150909 | 2.10E-14 | 0.272496342 | 58.431911562 | Hand grip strength (left) | ukb-b-7478 |
| rs11111267 | G | A | 0.18106 | 0.0108359 | 0.00192886 | 1.90E-08 | 0.168263438 | 31.559387292 | Hand grip strength (left) | ukb-b-7478 |
| rs11121542 | A | G | 0.122677 | -0.0157601 | 0.00225916 | 3.00E-12 | 0.237781827 | 48.665810169 | Hand grip strength (left) | ukb-b-7478 |
| rs11125803 | T | C | 0.740516 | 0.0142851 | 0.00169673 | 3.80E-17 | 0.312420446 | 70.882837180 | Hand grip strength (left) | ukb-b-7478 |
| rs11168357 | A | G | 0.246008 | -0.00962864 | 0.00172661 | 0.000000025 | 0.166215211 | 31.098639863 | Hand grip strength (left) | ukb-b-7478 |
| rs11204664 | C | T | 0.578725 | -0.00865184 | 0.00150032 | 8.10E-09 | 0.175712706 | 33.254403446 | Hand grip strength (left) | ukb-b-7478 |
| rs11243202 | C | T | 0.486019 | 0.00972237 | 0.0014909 | 7.00E-11 | 0.214205899 | 42.525287667 | Hand grip strength (left) | ukb-b-7478 |
| rs112485536 | T | C | 0.075507 | 0.0161779 | 0.00281803 | 9.40E-09 | 0.174417133 | 32.957409548 | Hand grip strength (left) | ukb-b-7478 |
| rs113315602 | C | A | 0.095848 | -0.0181886 | 0.00266345 | 8.60E-12 | 0.230141834 | 46.634727951 | Hand grip strength (left) | ukb-b-7478 |
| rs113434679 | A | C | 0.199577 | -0.0149156 | 0.00187618 | 1.90E-15 | 0.288328432 | 63.202237286 | Hand grip strength (left) | ukb-b-7478 |
| rs113918482 | G | A | 0.222507 | -0.0101011 | 0.00178515 | 0.000000015 | 0.170290160 | 32.017536457 | Hand grip strength (left) | ukb-b-7478 |
| rs116409670 | T | C | 0.079819 | -0.0152418 | 0.00273867 | 2.60E-08 | 0.165658064 | 30.973701473 | Hand grip strength (left) | ukb-b-7478 |
| rs11642954 | A | G | 0.195019 | -0.0117025 | 0.00187683 | 4.50E-10 | 0.199500465 | 38.878314274 | Hand grip strength (left) | ukb-b-7478 |
| rs11669079 | T | A | 0.705026 | 0.0109542 | 0.00163169 | 1.90E-11 | 0.224150181 | 45.069841218 | Hand grip strength (left) | ukb-b-7478 |
| rs116922558 | G | A | 0.039916 | -0.0215842 | 0.00385425 | 0.000000021 | 0.167383475 | 31.361162364 | Hand grip strength (left) | ukb-b-7478 |
| rs11769549 | A | T | 0.062372 | 0.0204956 | 0.00311228 | 4.50E-11 | 0.217525253 | 43.367456292 | Hand grip strength (left) | ukb-b-7478 |
| rs12316046 | G | A | 0.37795 | -0.0174165 | 0.00152991 | 5.00E-30 | 0.453772952 | 129.595523862 | Hand grip strength (left) | ukb-b-7478 |
| rs12473732 | T | C | 0.486507 | 0.0109869 | 0.00148601 | 1.40E-13 | 0.259486718 | 54.664688719 | Hand grip strength (left) | ukb-b-7478 |
| rs12528131 | G | A | 0.488343 | -0.00882788 | 0.00148565 | 2.80E-09 | 0.184563347 | 35.308545491 | Hand grip strength (left) | ukb-b-7478 |
| rs12533765 | G | A | 0.280054 | -0.0092 | 0.00165199 | 2.60E-08 | 0.165838821 | 31.014217379 | Hand grip strength (left) | ukb-b-7478 |
| rs12673062 | A | G | 0.215756 | -0.0107691 | 0.00180808 | 2.60E-09 | 0.185272622 | 35.475092371 | Hand grip strength (left) | ukb-b-7478 |
| rs12790261 | A | C | 0.082383 | -0.0251882 | 0.00270305 | 1.20E-20 | 0.357583884 | 86.833260312 | Hand grip strength (left) | ukb-b-7478 |
| rs12889267 | G | A | 0.167084 | -0.0137394 | 0.00198726 | 4.70E-12 | 0.234542949 | 47.799808077 | Hand grip strength (left) | ukb-b-7478 |
| rs12906830 | C | T | 0.601065 | 0.0108401 | 0.00151797 | 9.30E-13 | 0.246363996 | 50.996479976 | Hand grip strength (left) | ukb-b-7478 |
| rs12914702 | A | G | 0.300395 | 0.0109514 | 0.00169071 | 9.30E-11 | 0.211948767 | 41.956672801 | Hand grip strength (left) | ukb-b-7478 |
| rs13091492 | G | A | 0.372833 | -0.00847903 | 0.00153407 | 3.30E-08 | 0.163760157 | 30.549351090 | Hand grip strength (left) | ukb-b-7478 |
| rs13106087 | C | T | 0.829801 | 0.0116202 | 0.00197399 | 3.90E-09 | 0.181758323 | 34.652718400 | Hand grip strength (left) | ukb-b-7478 |
| rs13107325 | T | C | 0.074908 | -0.0261553 | 0.0028208 | 1.80E-20 | 0.355306691 | 85.975522045 | Hand grip strength (left) | ukb-b-7478 |
| rs13146142 | C | T | 0.158623 | -0.0202031 | 0.00202888 | 2.30E-23 | 0.388611677 | 99.156983150 | Hand grip strength (left) | ukb-b-7478 |
| rs13227429 | C | T | 0.561343 | -0.00858767 | 0.0014977 | 9.80E-09 | 0.174068593 | 32.877670236 | Hand grip strength (left) | ukb-b-7478 |
| rs13337177 | T | G | 0.180836 | -0.0142799 | 0.00193621 | 1.60E-13 | 0.258531525 | 54.393300847 | Hand grip strength (left) | ukb-b-7478 |
| rs13356200 | G | T | 0.393874 | -0.00876765 | 0.0015286 | 9.70E-09 | 0.174160529 | 32.898697108 | Hand grip strength (left) | ukb-b-7478 |
| rs143002906 | T | C | 0.027829 | 0.0262281 | 0.00457421 | 9.80E-09 | 0.174068734 | 32.877702676 | Hand grip strength (left) | ukb-b-7478 |
| rs143384 | G | A | 0.404388 | 0.0209202 | 0.00151159 | 1.50E-43 | 0.551133175 | 191.541835103 | Hand grip strength (left) | ukb-b-7478 |
| rs1434095 | C | T | 0.875121 | 0.0140301 | 0.00225906 | 5.30E-10 | 0.198237987 | 38.571453191 | Hand grip strength (left) | ukb-b-7478 |
| rs1486925 | C | T | 0.314604 | -0.0104734 | 0.00160211 | 6.30E-11 | 0.215037822 | 42.735689812 | Hand grip strength (left) | ukb-b-7478 |
| rs150330307 | C | T | 0.031896 | -0.0307753 | 0.0042167 | 2.90E-13 | 0.254541262 | 53.267115840 | Hand grip strength (left) | ukb-b-7478 |
| rs1551042 | C | A | 0.647025 | -0.0111279 | 0.00155167 | 7.40E-13 | 0.247943863 | 51.431323626 | Hand grip strength (left) | ukb-b-7478 |
| rs1556659 | T | C | 0.38153 | 0.0162896 | 0.00153452 | 2.50E-26 | 0.419399855 | 112.687497371 | Hand grip strength (left) | ukb-b-7478 |
| rs1641457 | G | T | 0.223305 | 0.0120456 | 0.0017829 | 1.40E-11 | 0.226367072 | 45.646018804 | Hand grip strength (left) | ukb-b-7478 |
| rs16870531 | T | C | 0.238492 | 0.011198 | 0.00174241 | 1.30E-10 | 0.209337396 | 41.302868678 | Hand grip strength (left) | ukb-b-7478 |
| rs16910750 | C | G | 0.159639 | 0.0112498 | 0.00204139 | 3.60E-08 | 0.162953164 | 30.369499677 | Hand grip strength (left) | ukb-b-7478 |
| rs17282763 | C | T | 0.296281 | 0.00893956 | 0.00163298 | 4.40E-08 | 0.161149986 | 29.968882966 | Hand grip strength (left) | ukb-b-7478 |
| rs17466480 | G | A | 0.386968 | -0.0118235 | 0.00152732 | 9.80E-15 | 0.277537995 | 59.928310440 | Hand grip strength (left) | ukb-b-7478 |
| rs17630248 | C | T | 0.347808 | -0.009184 | 0.00156162 | 4.10E-09 | 0.181476221 | 34.587010455 | Hand grip strength (left) | ukb-b-7478 |
| rs181766 | C | T | 0.321793 | 0.00963247 | 0.00160372 | 1.90E-09 | 0.187821439 | 36.075988477 | Hand grip strength (left) | ukb-b-7478 |
| rs185320691 | C | G | 0.104199 | -0.0166866 | 0.00268915 | 5.50E-10 | 0.197959868 | 38.503982871 | Hand grip strength (left) | ukb-b-7478 |
| rs1884447 | A | G | 0.400566 | 0.00846069 | 0.00151391 | 2.30E-08 | 0.166812822 | 31.232838115 | Hand grip strength (left) | ukb-b-7478 |
| rs1981612 | A | C | 0.45601 | 0.00922755 | 0.00151053 | 0.000000001 | 0.193037921 | 37.317634277 | Hand grip strength (left) | ukb-b-7478 |
| rs2038760 | T | C | 0.170908 | -0.0115262 | 0.00198662 | 6.60E-09 | 0.177485090 | 33.662215463 | Hand grip strength (left) | ukb-b-7478 |
| rs217181 | T | C | 0.193087 | 0.0119554 | 0.00188145 | 2.10E-10 | 0.205612932 | 40.377819261 | Hand grip strength (left) | ukb-b-7478 |
| rs2359239 | T | C | 0.391621 | -0.00881461 | 0.00152153 | 6.90E-09 | 0.177049357 | 33.561793747 | Hand grip strength (left) | ukb-b-7478 |
| rs2431112 | A | G | 0.440641 | -0.00958496 | 0.00149514 | 1.40E-10 | 0.208514117 | 41.097640484 | Hand grip strength (left) | ukb-b-7478 |
| rs2532111 | G | A | 0.639542 | 0.0102852 | 0.00155794 | 4.10E-11 | 0.218373016 | 43.583692965 | Hand grip strength (left) | ukb-b-7478 |
| rs2587505 | C | T | 0.419871 | -0.00900424 | 0.00150694 | 2.30E-09 | 0.186240342 | 35.702793882 | Hand grip strength (left) | ukb-b-7478 |
| rs2631360 | A | G | 0.519209 | -0.0109177 | 0.00148434 | 1.90E-13 | 0.257495667 | 54.099783977 | Hand grip strength (left) | ukb-b-7478 |
| rs2789514 | A | G | 0.867941 | 0.0120546 | 0.00220932 | 4.90E-08 | 0.160254876 | 29.770652949 | Hand grip strength (left) | ukb-b-7478 |
| rs2800789 | C | A | 0.480339 | 0.00826025 | 0.00149261 | 3.10E-08 | 0.164104686 | 30.626240557 | Hand grip strength (left) | ukb-b-7478 |
| rs2850379 | A | C | 0.432305 | -0.00828995 | 0.00150007 | 3.30E-08 | 0.163721938 | 30.540825458 | Hand grip strength (left) | ukb-b-7478 |
| rs28542042 | T | C | 0.308877 | 0.0110101 | 0.00162277 | 1.20E-11 | 0.227848635 | 46.032926572 | Hand grip strength (left) | ukb-b-7478 |
| rs2871865 | G | C | 0.116247 | -0.0217979 | 0.00231659 | 5.00E-21 | 0.362063003 | 88.538254786 | Hand grip strength (left) | ukb-b-7478 |
| rs2871960 | C | A | 0.444681 | 0.0120879 | 0.00149222 | 5.50E-16 | 0.296092304 | 65.619966469 | Hand grip strength (left) | ukb-b-7478 |
| rs2974438 | A | G | 0.211026 | -0.0100735 | 0.00182387 | 3.30E-08 | 0.163561926 | 30.505139951 | Hand grip strength (left) | ukb-b-7478 |
| rs3118903 | A | G | 0.219552 | -0.0174477 | 0.00179692 | 2.70E-22 | 0.376697726 | 94.279850585 | Hand grip strength (left) | ukb-b-7478 |
| rs34030812 | C | T | 0.367365 | -0.0101679 | 0.00154042 | 4.10E-11 | 0.218318080 | 43.569666471 | Hand grip strength (left) | ukb-b-7478 |
| rs34587452 | C | G | 0.21517 | -0.0113642 | 0.00180741 | 3.20E-10 | 0.202182416 | 39.533418966 | Hand grip strength (left) | ukb-b-7478 |
| rs34722008 | A | G | 0.353332 | 0.00858569 | 0.00155452 | 3.30E-08 | 0.163557125 | 30.504069322 | Hand grip strength (left) | ukb-b-7478 |
| rs34845616 | A | G | 0.245653 | 0.0108395 | 0.00173373 | 4.00E-10 | 0.200365335 | 39.089090988 | Hand grip strength (left) | ukb-b-7478 |
| rs35054365 | A | T | 0.437643 | 0.0128072 | 0.00149718 | 1.20E-17 | 0.319296288 | 73.174598681 | Hand grip strength (left) | ukb-b-7478 |
| rs35236379 | T | G | 0.142324 | 0.0123565 | 0.00212204 | 5.80E-09 | 0.178543413 | 33.906566595 | Hand grip strength (left) | ukb-b-7478 |
| rs35609019 | C | G | 0.397748 | 0.00947689 | 0.00154061 | 7.70E-10 | 0.195210784 | 37.839575391 | Hand grip strength (left) | ukb-b-7478 |
| rs3814877 | T | G | 0.401594 | 0.0105385 | 0.00151385 | 3.40E-12 | 0.237018114 | 48.460948419 | Hand grip strength (left) | ukb-b-7478 |
| rs3819121 | C | T | 0.368945 | 0.0140963 | 0.00152872 | 2.90E-20 | 0.352768301 | 85.026513833 | Hand grip strength (left) | ukb-b-7478 |
| rs3959716 | G | C | 0.56703 | -0.00832523 | 0.0015043 | 3.10E-08 | 0.164114124 | 30.628347799 | Hand grip strength (left) | ukb-b-7478 |
| rs4121165 | A | G | 0.211447 | -0.0114169 | 0.00181868 | 3.40E-10 | 0.201670135 | 39.407947183 | Hand grip strength (left) | ukb-b-7478 |
| rs41271299 | T | C | 0.051241 | 0.0211891 | 0.00336402 | 3.00E-10 | 0.202756309 | 39.674173016 | Hand grip strength (left) | ukb-b-7478 |
| rs4308051 | G | T | 0.789105 | 0.0159929 | 0.00181863 | 1.40E-18 | 0.331428067 | 77.333157304 | Hand grip strength (left) | ukb-b-7478 |
| rs4335354 | A | C | 0.315536 | -0.00939409 | 0.00160262 | 4.60E-09 | 0.180498457 | 34.359617203 | Hand grip strength (left) | ukb-b-7478 |
| rs4398863 | C | G | 0.7368 | -0.00945015 | 0.00168454 | 0.00000002 | 0.167872650 | 31.471304733 | Hand grip strength (left) | ukb-b-7478 |
| rs4498020 | A | C | 0.723767 | -0.0104238 | 0.00166995 | 4.30E-10 | 0.199845531 | 38.962355429 | Hand grip strength (left) | ukb-b-7478 |
| rs4575361 | T | A | 0.31205 | -0.0108028 | 0.00160223 | 1.60E-11 | 0.225650129 | 45.459321896 | Hand grip strength (left) | ukb-b-7478 |
| rs4621706 | T | C | 0.544473 | -0.0117068 | 0.00150003 | 6.00E-15 | 0.280802086 | 60.908304195 | Hand grip strength (left) | ukb-b-7478 |
| rs4677601 | G | A | 0.510324 | 0.00905884 | 0.001485 | 1.10E-09 | 0.192600048 | 37.212793318 | Hand grip strength (left) | ukb-b-7478 |
| rs4713506 | A | G | 0.255607 | -0.0157268 | 0.00169811 | 2.00E-20 | 0.354765865 | 85.772701464 | Hand grip strength (left) | ukb-b-7478 |
| rs4737446 | T | G | 0.694626 | 0.0104178 | 0.00161882 | 1.20E-10 | 0.209785438 | 41.414737082 | Hand grip strength (left) | ukb-b-7478 |
| rs4739739 | G | A | 0.415057 | -0.00853387 | 0.00150466 | 1.40E-08 | 0.170950755 | 32.167350703 | Hand grip strength (left) | ukb-b-7478 |
| rs4811040 | G | C | 0.276636 | -0.00924973 | 0.00166896 | 3.00E-08 | 0.164507029 | 30.716112926 | Hand grip strength (left) | ukb-b-7478 |
| rs4930236 | A | C | 0.835892 | 0.0119032 | 0.00202069 | 3.80E-09 | 0.181960717 | 34.699888322 | Hand grip strength (left) | ukb-b-7478 |
| rs4962700 | G | C | 0.301977 | 0.00904307 | 0.00163488 | 0.000000032 | 0.163967756 | 30.595673887 | Hand grip strength (left) | ukb-b-7478 |
| rs55681913 | C | T | 0.106095 | 0.0137738 | 0.00243658 | 0.000000016 | 0.170016495 | 31.955542512 | Hand grip strength (left) | ukb-b-7478 |
| rs56060323 | T | C | 0.314543 | 0.00906905 | 0.00160238 | 0.000000015 | 0.170356750 | 32.032627331 | Hand grip strength (left) | ukb-b-7478 |
| rs56338231 | G | A | 0.258266 | -0.010847 | 0.00169739 | 1.70E-10 | 0.207466898 | 40.837204209 | Hand grip strength (left) | ukb-b-7478 |
| rs58670122 | G | A | 0.143159 | -0.0118148 | 0.00214462 | 3.60E-08 | 0.162863488 | 30.349535260 | Hand grip strength (left) | ukb-b-7478 |
| rs59116179 | T | C | 0.617442 | 0.00857385 | 0.00153635 | 2.40E-08 | 0.166416360 | 31.143787895 | Hand grip strength (left) | ukb-b-7478 |
| rs6006984 | C | T | 0.277764 | 0.00984917 | 0.00165413 | 2.60E-09 | 0.185181040 | 35.453571436 | Hand grip strength (left) | ukb-b-7478 |
| rs61286123 | C | T | 0.227916 | -0.0101041 | 0.00177111 | 0.000000012 | 0.172617865 | 32.546493191 | Hand grip strength (left) | ukb-b-7478 |
| rs61389091 | T | C | 0.04174 | 0.0261627 | 0.00373295 | 2.40E-12 | 0.239470593 | 49.120273556 | Hand grip strength (left) | ukb-b-7478 |
| rs61818100 | C | T | 0.116595 | 0.0134422 | 0.00231335 | 6.20E-09 | 0.177927655 | 33.764320670 | Hand grip strength (left) | ukb-b-7478 |
| rs62081464 | T | C | 0.227338 | -0.0098827 | 0.00177979 | 2.80E-08 | 0.165029071 | 30.832852093 | Hand grip strength (left) | ukb-b-7478 |
| rs62253653 | G | A | 0.295379 | 0.0106985 | 0.00163146 | 5.50E-11 | 0.216089945 | 43.002422468 | Hand grip strength (left) | ukb-b-7478 |
| rs635538 | A | G | 0.913853 | -0.0216569 | 0.00265852 | 3.80E-16 | 0.298438027 | 66.360968961 | Hand grip strength (left) | ukb-b-7478 |
| rs6433478 | C | T | 0.544228 | 0.00905674 | 0.00149951 | 1.50E-09 | 0.189522732 | 36.479180028 | Hand grip strength (left) | ukb-b-7478 |
| rs6680160 | G | A | 0.628066 | 0.0100686 | 0.00153878 | 6.00E-11 | 0.215346737 | 42.813931466 | Hand grip strength (left) | ukb-b-7478 |
| rs6689375 | T | A | 0.185489 | -0.0160169 | 0.00191158 | 5.30E-17 | 0.310362008 | 70.205635048 | Hand grip strength (left) | ukb-b-7478 |
| rs6802071 | T | C | 0.434928 | -0.00940386 | 0.00150182 | 3.80E-10 | 0.200853109 | 39.208167304 | Hand grip strength (left) | ukb-b-7478 |
| rs6882168 | T | C | 0.337417 | -0.00935387 | 0.00157364 | 2.80E-09 | 0.184664578 | 35.332298188 | Hand grip strength (left) | ukb-b-7478 |
| rs6962338 | G | A | 0.043993 | -0.0214095 | 0.0036171 | 3.20E-09 | 0.183392229 | 35.034184958 | Hand grip strength (left) | ukb-b-7478 |
| rs6977081 | T | G | 0.333835 | 0.0147525 | 0.00158641 | 1.40E-20 | 0.356639881 | 86.476951028 | Hand grip strength (left) | ukb-b-7478 |
| rs7026798 | C | T | 0.43232 | 0.00823598 | 0.00150641 | 4.60E-08 | 0.160799695 | 29.891257557 | Hand grip strength (left) | ukb-b-7478 |
| rs7124681 | A | C | 0.408355 | -0.0116557 | 0.00150615 | 1.00E-14 | 0.277403310 | 59.888063380 | Hand grip strength (left) | ukb-b-7478 |
| rs71298370 | A | G | 0.086241 | 0.0148764 | 0.00270414 | 3.80E-08 | 0.162482515 | 30.264768049 | Hand grip strength (left) | ukb-b-7478 |
| rs7148603 | A | G | 0.359427 | 0.00957206 | 0.00158612 | 1.60E-09 | 0.189273040 | 36.419899391 | Hand grip strength (left) | ukb-b-7478 |
| rs7176095 | G | A | 0.128192 | -0.013375 | 0.00222025 | 1.70E-09 | 0.188724254 | 36.289737262 | Hand grip strength (left) | ukb-b-7478 |
| rs7196917 | G | A | 0.429948 | -0.0117302 | 0.00150067 | 5.40E-15 | 0.281436764 | 61.099890592 | Hand grip strength (left) | ukb-b-7478 |
| rs7197751 | T | G | 0.363019 | -0.00946321 | 0.00156268 | 1.40E-09 | 0.190334631 | 36.672190140 | Hand grip strength (left) | ukb-b-7478 |
| rs723588 | C | T | 0.142598 | 0.0127904 | 0.00211948 | 1.60E-09 | 0.189262747 | 36.417456283 | Hand grip strength (left) | ukb-b-7478 |
| rs72977282 | A | T | 0.413856 | -0.0155569 | 0.00151173 | 7.80E-25 | 0.404353747 | 105.900413736 | Hand grip strength (left) | ukb-b-7478 |
| rs73307079 | C | T | 0.211119 | 0.0111025 | 0.00183407 | 1.40E-09 | 0.190218588 | 36.644580084 | Hand grip strength (left) | ukb-b-7478 |
| rs7516571 | G | A | 0.259017 | 0.00937798 | 0.00169361 | 3.10E-08 | 0.164262073 | 30.661386224 | Hand grip strength (left) | ukb-b-7478 |
| rs75497896 | C | T | 0.051006 | -0.0206727 | 0.00337524 | 9.10E-10 | 0.193853793 | 37.513284092 | Hand grip strength (left) | ukb-b-7478 |
| rs755547 | A | G | 0.188906 | 0.0165156 | 0.00189766 | 3.20E-18 | 0.326845272 | 75.744639875 | Hand grip strength (left) | ukb-b-7478 |
| rs7571789 | C | T | 0.522782 | 0.0129539 | 0.001487 | 3.00E-18 | 0.327264496 | 75.889054708 | Hand grip strength (left) | ukb-b-7478 |
| rs7575451 | G | C | 0.649752 | -0.00973095 | 0.00155349 | 3.80E-10 | 0.200970300 | 39.236797951 | Hand grip strength (left) | ukb-b-7478 |
| rs76895963 | G | T | 0.020694 | 0.0359634 | 0.00574862 | 3.90E-10 | 0.200564149 | 39.137608435 | Hand grip strength (left) | ukb-b-7478 |
| rs772014 | G | A | 0.392538 | -0.0106178 | 0.00151891 | 2.70E-12 | 0.238525897 | 48.865798343 | Hand grip strength (left) | ukb-b-7478 |
| rs77485342 | T | C | 0.018003 | 0.0329675 | 0.00558187 | 3.50E-09 | 0.182745027 | 34.882900914 | Hand grip strength (left) | ukb-b-7478 |
| rs7856625 | T | C | 0.609861 | -0.0111297 | 0.0015209 | 2.50E-13 | 0.255550283 | 53.550754782 | Hand grip strength (left) | ukb-b-7478 |
| rs7963801 | C | T | 0.571927 | -0.0104348 | 0.00150824 | 4.60E-12 | 0.234791569 | 47.866023533 | Hand grip strength (left) | ukb-b-7478 |
| rs7970350 | T | C | 0.494068 | -0.0101345 | 0.00148418 | 8.60E-12 | 0.230110038 | 46.626359094 | Hand grip strength (left) | ukb-b-7478 |
| rs8101782 | C | A | 0.702702 | 0.00955082 | 0.00172167 | 2.90E-08 | 0.164765197 | 30.773826180 | Hand grip strength (left) | ukb-b-7478 |
| rs8108461 | C | T | 0.572775 | 0.00952393 | 0.0015066 | 2.60E-10 | 0.203923273 | 39.961010637 | Hand grip strength (left) | ukb-b-7478 |
| rs821100 | G | A | 0.265512 | -0.0101977 | 0.00168615 | 1.50E-09 | 0.189935821 | 36.577334117 | Hand grip strength (left) | ukb-b-7478 |
| rs823130 | T | C | 0.432903 | -0.0113317 | 0.00150098 | 4.40E-14 | 0.267590051 | 56.995468211 | Hand grip strength (left) | ukb-b-7478 |
| rs9371201 | T | C | 0.335102 | -0.00933847 | 0.0015738 | 3.00E-09 | 0.184138366 | 35.208893265 | Hand grip strength (left) | ukb-b-7478 |
| rs9371881 | A | G | 0.359174 | 0.00947946 | 0.00154996 | 9.60E-10 | 0.193401209 | 37.404703440 | Hand grip strength (left) | ukb-b-7478 |
| rs9388769 | A | G | 0.67318 | -0.0140821 | 0.00158065 | 5.10E-19 | 0.337217370 | 79.371285953 | Hand grip strength (left) | ukb-b-7478 |
| rs9611273 | T | C | 0.25267 | 0.0107888 | 0.00173048 | 4.50E-10 | 0.199465787 | 38.869872498 | Hand grip strength (left) | ukb-b-7478 |
| rs9866627 | A | C | 0.084376 | -0.0155989 | 0.00267761 | 5.70E-09 | 0.178681744 | 33.938551706 | Hand grip strength (left) | ukb-b-7478 |
| rs9944324 | G | A | 0.456723 | -0.00855272 | 0.00150169 | 0.000000012 | 0.172139486 | 32.437541519 | Hand grip strength (left) | ukb-b-7478 |
| rs997850 | C | G | 0.60474 | -0.00887326 | 0.0015279 | 6.30E-09 | 0.177765557 | 33.726909913 | Hand grip strength (left) | ukb-b-7478 |
| rs999493 | A | G | 0.621921 | 0.0128713 | 0.00153936 | 6.20E-17 | 0.309471884 | 69.914045210 | Hand grip strength (left) | ukb-b-7478 |

Supplementary Table 6: Information on all instrumental variables (IVs) ultimately used for Hand grip strength (right) in our study

| SNPs | effect_allele.exposure | other_allele.exposure | eaf.exposure | beta.exposure | se.exposure | pval.exposure | R^2^ | F | trait | id.exposure |
| --- | --- | --- | --- | --- | --- | --- | --- | --- | --- | --- |
| rs10193039 | T | A | 0.28057 | -0.0103284 | 0.00165217 | 4.10E-10 | 0.183406011 | 39.080187076 | Hand grip strength (right) | ukb-b-10215 |
| rs10278546 | C | A | 0.194705 | 0.0107539 | 0.00188373 | 1.10E-08 | 0.157755226 | 32.590774300 | Hand grip strength (right) | ukb-b-10215 |
| rs1043515 | G | A | 0.566183 | 0.0138281 | 0.00149848 | 2.80E-20 | 0.328593727 | 85.157542781 | Hand grip strength (right) | ukb-b-10215 |
| rs1047891 | A | C | 0.315772 | 0.0096867 | 0.0015965 | 1.30E-09 | 0.174628152 | 36.814071688 | Hand grip strength (right) | ukb-b-10215 |
| rs10483727 | C | T | 0.610138 | -0.00901803 | 0.00152456 | 3.30E-09 | 0.167421187 | 34.989223906 | Hand grip strength (right) | ukb-b-10215 |
| rs10520770 | C | T | 0.44929 | 0.011742 | 0.00149773 | 4.50E-15 | 0.261031879 | 61.463472682 | Hand grip strength (right) | ukb-b-10215 |
| rs10761411 | T | C | 0.812419 | -0.0114975 | 0.00202082 | 1.30E-08 | 0.156856896 | 32.370661444 | Hand grip strength (right) | ukb-b-10215 |
| rs10770125 | G | A | 0.477072 | 0.00844132 | 0.00148775 | 1.40E-08 | 0.156130228 | 32.192952621 | Hand grip strength (right) | ukb-b-10215 |
| rs10784502 | T | C | 0.512218 | -0.0111247 | 0.00148608 | 7.10E-14 | 0.243607308 | 56.039240104 | Hand grip strength (right) | ukb-b-10215 |
| rs10798483 | A | G | 0.54729 | 0.0145083 | 0.00149469 | 2.80E-22 | 0.351272345 | 94.217330849 | Hand grip strength (right) | ukb-b-10215 |
| rs10798876 | G | C | 0.55229 | 0.00879466 | 0.00149794 | 4.30E-09 | 0.165350071 | 34.470634113 | Hand grip strength (right) | ukb-b-10215 |
| rs10799428 | T | C | 0.18721 | -0.0143588 | 0.00190367 | 4.60E-14 | 0.246401667 | 56.892230503 | Hand grip strength (right) | ukb-b-10215 |
| rs10846071 | T | C | 0.393685 | -0.0156575 | 0.00151902 | 6.50E-25 | 0.379119774 | 106.247288839 | Hand grip strength (right) | ukb-b-10215 |
| rs11022513 | T | C | 0.569312 | -0.00921054 | 0.00151016 | 1.10E-09 | 0.176130122 | 37.198400014 | Hand grip strength (right) | ukb-b-10215 |
| rs11039348 | A | G | 0.348363 | -0.00976855 | 0.00156104 | 3.90E-10 | 0.183708127 | 39.159049747 | Hand grip strength (right) | ukb-b-10215 |
| rs112330055 | A | G | 0.062972 | 0.0179824 | 0.00319007 | 0.000000017 | 0.154419034 | 31.775682097 | Hand grip strength (right) | ukb-b-10215 |
| rs11243202 | C | T | 0.486018 | 0.0116378 | 0.00149304 | 6.50E-15 | 0.258809180 | 60.757359701 | Hand grip strength (right) | ukb-b-10215 |
| rs1125 | A | G | 0.336554 | -0.0100304 | 0.00157472 | 1.90E-10 | 0.189084586 | 40.572317768 | Hand grip strength (right) | ukb-b-10215 |
| rs113315602 | C | A | 0.095826 | -0.0212911 | 0.00266761 | 1.40E-15 | 0.267990318 | 63.701773850 | Hand grip strength (right) | ukb-b-10215 |
| rs113835839 | T | C | 0.247871 | -0.00986353 | 0.00172486 | 1.10E-08 | 0.158203138 | 32.700699133 | Hand grip strength (right) | ukb-b-10215 |
| rs113851275 | A | G | 0.107596 | 0.0131425 | 0.00239826 | 4.30E-08 | 0.147186582 | 30.030560864 | Hand grip strength (right) | ukb-b-10215 |
| rs114924396 | G | A | 0.05363 | -0.0191251 | 0.00331785 | 8.20E-09 | 0.160341944 | 33.227214412 | Hand grip strength (right) | ukb-b-10215 |
| rs11642954 | A | G | 0.195039 | -0.0131918 | 0.00187938 | 2.20E-12 | 0.220673075 | 49.269586227 | Hand grip strength (right) | ukb-b-10215 |
| rs116922558 | G | A | 0.039907 | -0.0246271 | 0.00386049 | 1.80E-10 | 0.189548187 | 40.695059154 | Hand grip strength (right) | ukb-b-10215 |
| rs11998884 | T | C | 0.061825 | 0.0173433 | 0.0031243 | 2.80E-08 | 0.150451624 | 30.814705062 | Hand grip strength (right) | ukb-b-10215 |
| rs12052508 | T | C | 0.879044 | -0.0133517 | 0.00227992 | 4.70E-09 | 0.164647279 | 34.295245379 | Hand grip strength (right) | ukb-b-10215 |
| rs12101479 | C | G | 0.237244 | -0.0106133 | 0.0017554 | 1.50E-09 | 0.173613224 | 36.555160298 | Hand grip strength (right) | ukb-b-10215 |
| rs12316046 | G | A | 0.377959 | -0.0162046 | 0.00153212 | 3.80E-26 | 0.391319345 | 111.864186018 | Hand grip strength (right) | ukb-b-10215 |
| rs12412806 | A | G | 0.294214 | -0.00909582 | 0.00164139 | 3.00E-08 | 0.150011323 | 30.708609254 | Hand grip strength (right) | ukb-b-10215 |
| rs12452505 | G | C | 0.142315 | -0.0144313 | 0.00213545 | 1.40E-11 | 0.207903174 | 45.670113985 | Hand grip strength (right) | ukb-b-10215 |
| rs12522139 | G | T | 0.171144 | -0.0114381 | 0.00197332 | 6.80E-09 | 0.161841423 | 33.597947191 | Hand grip strength (right) | ukb-b-10215 |
| rs12562146 | A | T | 0.145156 | 0.0118706 | 0.00211554 | 0.00000002 | 0.153222613 | 31.484939229 | Hand grip strength (right) | ukb-b-10215 |
| rs12616285 | G | T | 0.148607 | 0.0122195 | 0.00209396 | 5.40E-09 | 0.163679284 | 34.054155035 | Hand grip strength (right) | ukb-b-10215 |
| rs12763284 | G | A | 0.466048 | 0.00959741 | 0.00148928 | 1.20E-10 | 0.192685447 | 41.529373723 | Hand grip strength (right) | ukb-b-10215 |
| rs12823922 | G | A | 0.222184 | -0.0114203 | 0.00178649 | 1.60E-10 | 0.190190019 | 40.865220441 | Hand grip strength (right) | ukb-b-10215 |
| rs12889267 | G | A | 0.167048 | -0.0122874 | 0.0019904 | 6.70E-10 | 0.179671033 | 38.110027903 | Hand grip strength (right) | ukb-b-10215 |
| rs12899474 | T | C | 0.108065 | -0.0148939 | 0.00239936 | 5.40E-10 | 0.181301291 | 38.532398082 | Hand grip strength (right) | ukb-b-10215 |
| rs12914702 | A | G | 0.300438 | 0.0107788 | 0.00169282 | 1.90E-10 | 0.188974966 | 40.543315834 | Hand grip strength (right) | ukb-b-10215 |
| rs13106087 | C | T | 0.829827 | 0.0129259 | 0.001977 | 6.20E-11 | 0.197221672 | 42.747256272 | Hand grip strength (right) | ukb-b-10215 |
| rs13107325 | T | C | 0.074904 | -0.0275 | 0.00282478 | 2.10E-22 | 0.352619589 | 94.775509609 | Hand grip strength (right) | ukb-b-10215 |
| rs13146142 | C | T | 0.158627 | -0.0207968 | 0.00203165 | 1.40E-24 | 0.375861029 | 104.784065740 | Hand grip strength (right) | ukb-b-10215 |
| rs13169333 | C | T | 0.260299 | 0.00929083 | 0.00169626 | 4.30E-08 | 0.147059670 | 30.000202433 | Hand grip strength (right) | ukb-b-10215 |
| rs13355365 | T | C | 0.379543 | -0.00846604 | 0.00153398 | 3.40E-08 | 0.148975267 | 30.459392517 | Hand grip strength (right) | ukb-b-10215 |
| rs13356200 | G | T | 0.393811 | -0.00920983 | 0.00153092 | 1.80E-09 | 0.172180721 | 36.190804240 | Hand grip strength (right) | ukb-b-10215 |
| rs143384 | G | A | 0.404392 | 0.0230423 | 0.00151366 | 2.50E-52 | 0.571150547 | 231.736788721 | Hand grip strength (right) | ukb-b-10215 |
| rs1440152 | G | C | 0.444561 | 0.00827068 | 0.00149983 | 3.50E-08 | 0.148764365 | 30.408735662 | Hand grip strength (right) | ukb-b-10215 |
| rs1442883 | A | C | 0.253067 | -0.0106189 | 0.00171426 | 5.80E-10 | 0.180679968 | 38.371226290 | Hand grip strength (right) | ukb-b-10215 |
| rs1486925 | C | T | 0.314649 | -0.0095288 | 0.00160467 | 2.90E-09 | 0.168505825 | 35.261838866 | Hand grip strength (right) | ukb-b-10215 |
| rs150330307 | C | T | 0.031875 | -0.0325831 | 0.00422401 | 1.20E-14 | 0.254825841 | 59.502460845 | Hand grip strength (right) | ukb-b-10215 |
| rs1550115 | T | C | 0.748561 | 0.0152836 | 0.00171276 | 4.50E-19 | 0.313952150 | 79.626623839 | Hand grip strength (right) | ukb-b-10215 |
| rs1556659 | T | C | 0.381531 | 0.0175345 | 0.00153689 | 3.80E-30 | 0.427946006 | 130.167092175 | Hand grip strength (right) | ukb-b-10215 |
| rs1635527 | C | G | 0.547486 | 0.00975325 | 0.001495 | 6.80E-11 | 0.196532869 | 42.561441399 | Hand grip strength (right) | ukb-b-10215 |
| rs1641457 | G | T | 0.223327 | 0.0130726 | 0.00178536 | 2.40E-13 | 0.235545469 | 53.613275908 | Hand grip strength (right) | ukb-b-10215 |
| rs1840753 | T | C | 0.062797 | 0.0176942 | 0.00312088 | 1.40E-08 | 0.155932201 | 32.144577626 | Hand grip strength (right) | ukb-b-10215 |
| rs185320691 | C | G | 0.104173 | -0.020249 | 0.00269349 | 5.60E-14 | 0.245173836 | 56.516651879 | Hand grip strength (right) | ukb-b-10215 |
| rs1885690 | A | C | 0.410365 | -0.00839964 | 0.00151212 | 2.80E-08 | 0.150625588 | 30.856654009 | Hand grip strength (right) | ukb-b-10215 |
| rs1892425 | A | G | 0.238631 | 0.0113357 | 0.00175726 | 1.10E-10 | 0.192996994 | 41.612579768 | Hand grip strength (right) | ukb-b-10215 |
| rs1952256 | G | A | 0.345023 | 0.00976791 | 0.00156323 | 4.10E-10 | 0.183268420 | 39.044290631 | Hand grip strength (right) | ukb-b-10215 |
| rs2147461 | C | T | 0.117274 | 0.0134299 | 0.00231042 | 6.10E-09 | 0.162608420 | 33.788093483 | Hand grip strength (right) | ukb-b-10215 |
| rs2165241 | C | T | 0.508842 | 0.0122486 | 0.00148844 | 1.90E-16 | 0.280155745 | 67.718953665 | Hand grip strength (right) | ukb-b-10215 |
| rs2194411 | A | G | 0.128444 | 0.0142831 | 0.00224795 | 2.10E-10 | 0.188323805 | 40.371200053 | Hand grip strength (right) | ukb-b-10215 |
| rs2194747 | G | A | 0.707014 | 0.00985126 | 0.00163925 | 1.90E-09 | 0.171883886 | 36.115462266 | Hand grip strength (right) | ukb-b-10215 |
| rs2208562 | T | C | 0.609627 | -0.0115616 | 0.00152246 | 3.10E-14 | 0.248929150 | 57.669222669 | Hand grip strength (right) | ukb-b-10215 |
| rs2226685 | C | T | 0.758696 | 0.0103333 | 0.00174324 | 3.10E-09 | 0.168009175 | 35.136921726 | Hand grip strength (right) | ukb-b-10215 |
| rs2244621 | T | C | 0.144157 | 0.0115957 | 0.00211936 | 4.50E-08 | 0.146788470 | 29.935359296 | Hand grip strength (right) | ukb-b-10215 |
| rs2273555 | A | G | 0.606106 | 0.0110316 | 0.00152155 | 4.20E-13 | 0.232011710 | 52.565954593 | Hand grip strength (right) | ukb-b-10215 |
| rs2296316 | C | T | 0.464404 | -0.00821333 | 0.00150213 | 4.60E-08 | 0.146626771 | 29.896717345 | Hand grip strength (right) | ukb-b-10215 |
| rs2322754 | A | G | 0.831641 | -0.0117022 | 0.00198538 | 3.80E-09 | 0.166432863 | 34.741434536 | Hand grip strength (right) | ukb-b-10215 |
| rs2341184 | C | T | 0.271748 | 0.0100606 | 0.00167125 | 1.70E-09 | 0.172366787 | 36.238058677 | Hand grip strength (right) | ukb-b-10215 |
| rs2362972 | A | C | 0.577904 | -0.00856859 | 0.00150767 | 1.30E-08 | 0.156569204 | 32.300268982 | Hand grip strength (right) | ukb-b-10215 |
| rs2389763 | C | T | 0.591848 | -0.00832615 | 0.00151353 | 3.80E-08 | 0.148155410 | 30.262610909 | Hand grip strength (right) | ukb-b-10215 |
| rs2431112 | A | G | 0.440644 | -0.011172 | 0.00149737 | 8.60E-14 | 0.242383805 | 55.667741080 | Hand grip strength (right) | ukb-b-10215 |
| rs246181 | T | C | 0.373429 | 0.00970432 | 0.00154856 | 3.70E-10 | 0.184137370 | 39.271197442 | Hand grip strength (right) | ukb-b-10215 |
| rs248831 | A | G | 0.265259 | 0.00988989 | 0.00171686 | 8.40E-09 | 0.160161984 | 33.182809789 | Hand grip strength (right) | ukb-b-10215 |
| rs2587505 | C | T | 0.419822 | -0.0092717 | 0.0015091 | 8.10E-10 | 0.178264716 | 37.747022816 | Hand grip strength (right) | ukb-b-10215 |
| rs2631360 | A | G | 0.519189 | -0.0111803 | 0.00148648 | 5.40E-14 | 0.245349590 | 56.570337993 | Hand grip strength (right) | ukb-b-10215 |
| rs2717351 | G | A | 0.211783 | 0.0127695 | 0.00183473 | 3.40E-12 | 0.217766324 | 48.439924816 | Hand grip strength (right) | ukb-b-10215 |
| rs2854152 | G | A | 0.678266 | 0.010991 | 0.00159765 | 6.00E-12 | 0.213833759 | 47.327234547 | Hand grip strength (right) | ukb-b-10215 |
| rs2871865 | G | C | 0.116217 | -0.0236863 | 0.00232022 | 1.80E-24 | 0.374587579 | 104.216412480 | Hand grip strength (right) | ukb-b-10215 |
| rs2894602 | G | A | 0.766101 | 0.0101332 | 0.00176778 | 9.90E-09 | 0.158841933 | 32.857672502 | Hand grip strength (right) | ukb-b-10215 |
| rs3118914 | T | G | 0.215004 | -0.0194408 | 0.00180843 | 5.90E-27 | 0.399097839 | 115.564610265 | Hand grip strength (right) | ukb-b-10215 |
| rs34030812 | C | T | 0.36737 | -0.00912858 | 0.00154266 | 3.30E-09 | 0.167527678 | 35.015958136 | Hand grip strength (right) | ukb-b-10215 |
| rs34217742 | A | T | 0.124284 | 0.0146245 | 0.00227778 | 1.40E-10 | 0.191535831 | 41.222896413 | Hand grip strength (right) | ukb-b-10215 |
| rs34587452 | C | G | 0.215181 | -0.0110867 | 0.00181019 | 9.10E-10 | 0.177346953 | 37.510795034 | Hand grip strength (right) | ukb-b-10215 |
| rs34845616 | A | G | 0.245647 | 0.00979506 | 0.00173626 | 0.000000017 | 0.154626541 | 31.826192195 | Hand grip strength (right) | ukb-b-10215 |
| rs35304341 | A | G | 0.089496 | -0.0142862 | 0.00260278 | 0.00000004 | 0.147590256 | 30.127183290 | Hand grip strength (right) | ukb-b-10215 |
| rs35457492 | C | A | 0.495364 | 0.00833889 | 0.00149104 | 2.20E-08 | 0.152368663 | 31.277922645 | Hand grip strength (right) | ukb-b-10215 |
| rs35701422 | C | T | 0.625757 | -0.00869121 | 0.00153481 | 0.000000015 | 0.155612293 | 32.066476878 | Hand grip strength (right) | ukb-b-10215 |
| rs35833641 | G | A | 0.312389 | 0.00922306 | 0.00160181 | 8.50E-09 | 0.160042749 | 33.153399477 | Hand grip strength (right) | ukb-b-10215 |
| rs36065733 | G | T | 0.472588 | 0.00971991 | 0.00149596 | 8.20E-11 | 0.195251888 | 42.216723579 | Hand grip strength (right) | ukb-b-10215 |
| rs3771498 | T | C | 0.514178 | 0.0141605 | 0.00148856 | 1.90E-21 | 0.342142523 | 90.494979801 | Hand grip strength (right) | ukb-b-10215 |
| rs3848369 | T | C | 0.38657 | -0.00953776 | 0.00153126 | 4.70E-10 | 0.182318187 | 38.796710507 | Hand grip strength (right) | ukb-b-10215 |
| rs4121165 | A | G | 0.211455 | -0.0119792 | 0.00182142 | 4.80E-11 | 0.199097509 | 43.254911675 | Hand grip strength (right) | ukb-b-10215 |
| rs4369779 | C | T | 0.789015 | 0.017203 | 0.00182068 | 3.40E-21 | 0.339099922 | 89.277318108 | Hand grip strength (right) | ukb-b-10215 |
| rs4549685 | T | C | 0.329917 | 0.00971494 | 0.00158124 | 8.10E-10 | 0.178265376 | 37.747192983 | Hand grip strength (right) | ukb-b-10215 |
| rs4553566 | C | T | 0.453271 | -0.00930181 | 0.00149229 | 4.60E-10 | 0.182535770 | 38.853350071 | Hand grip strength (right) | ukb-b-10215 |
| rs4730984 | T | G | 0.239566 | 0.0104789 | 0.00174245 | 1.80E-09 | 0.172086387 | 36.166854774 | Hand grip strength (right) | ukb-b-10215 |
| rs4737446 | T | G | 0.694661 | 0.0102668 | 0.00162131 | 2.40E-10 | 0.187293451 | 40.099419087 | Hand grip strength (right) | ukb-b-10215 |
| rs4751671 | A | G | 0.530724 | 0.0082332 | 0.00150019 | 4.10E-08 | 0.147557313 | 30.119294735 | Hand grip strength (right) | ukb-b-10215 |
| rs4752689 | A | G | 0.584246 | 0.0087531 | 0.00150761 | 6.40E-09 | 0.162289516 | 33.708991632 | Hand grip strength (right) | ukb-b-10215 |
| rs4768725 | C | T | 0.699597 | 0.00905894 | 0.00162081 | 2.30E-08 | 0.152205961 | 31.238527297 | Hand grip strength (right) | ukb-b-10215 |
| rs4784329 | C | A | 0.426476 | -0.0133357 | 0.00150734 | 9.00E-19 | 0.310269639 | 78.272496506 | Hand grip strength (right) | ukb-b-10215 |
| rs4785574 | G | A | 0.555329 | -0.0103769 | 0.00149954 | 4.50E-12 | 0.215817653 | 47.887167897 | Hand grip strength (right) | ukb-b-10215 |
| rs4793658 | C | A | 0.110375 | -0.0136707 | 0.00248614 | 3.80E-08 | 0.148046164 | 30.236418219 | Hand grip strength (right) | ukb-b-10215 |
| rs4802848 | C | G | 0.730261 | 0.0110767 | 0.00167496 | 3.80E-11 | 0.200857069 | 43.733265521 | Hand grip strength (right) | ukb-b-10215 |
| rs4868110 | T | A | 0.323165 | -0.00971069 | 0.00158981 | 0.000000001 | 0.176560041 | 37.308666914 | Hand grip strength (right) | ukb-b-10215 |
| rs4927015 | A | G | 0.582858 | 0.013059 | 0.00150895 | 5.00E-18 | 0.300918412 | 74.897986905 | Hand grip strength (right) | ukb-b-10215 |
| rs4962700 | G | C | 0.302017 | 0.00932733 | 0.00163735 | 0.000000012 | 0.157186081 | 32.451265407 | Hand grip strength (right) | ukb-b-10215 |
| rs56074046 | A | G | 0.371748 | -0.00903502 | 0.00153954 | 4.40E-09 | 0.165231577 | 34.441041989 | Hand grip strength (right) | ukb-b-10215 |
| rs56144131 | C | T | 0.149344 | -0.01307 | 0.00208666 | 3.80E-10 | 0.183989933 | 39.232663492 | Hand grip strength (right) | ukb-b-10215 |
| rs56365901 | G | A | 0.222684 | -0.0142618 | 0.00179293 | 1.80E-15 | 0.266669126 | 63.273522909 | Hand grip strength (right) | ukb-b-10215 |
| rs58670122 | G | A | 0.14316 | -0.0132234 | 0.00214787 | 7.40E-10 | 0.178868461 | 37.902712069 | Hand grip strength (right) | ukb-b-10215 |
| rs600038 | C | T | 0.20706 | -0.0103761 | 0.00183358 | 0.000000015 | 0.155436084 | 32.023483378 | Hand grip strength (right) | ukb-b-10215 |
| rs6006984 | C | T | 0.277766 | 0.0102987 | 0.00165665 | 5.10E-10 | 0.181738231 | 38.645887483 | Hand grip strength (right) | ukb-b-10215 |
| rs6063504 | G | C | 0.492707 | 0.00862345 | 0.0014938 | 7.80E-09 | 0.160740156 | 33.325539513 | Hand grip strength (right) | ukb-b-10215 |
| rs61389091 | T | C | 0.041752 | 0.0220553 | 0.00373782 | 3.60E-09 | 0.166733813 | 34.816825596 | Hand grip strength (right) | ukb-b-10215 |
| rs62037412 | A | G | 0.357285 | 0.00932966 | 0.0015542 | 1.90E-09 | 0.171564450 | 36.034443957 | Hand grip strength (right) | ukb-b-10215 |
| rs62234790 | A | C | 0.245816 | 0.0105846 | 0.0017265 | 8.80E-10 | 0.177635863 | 37.585102185 | Hand grip strength (right) | ukb-b-10215 |
| rs62509875 | G | A | 0.170951 | -0.0131798 | 0.00197325 | 2.40E-11 | 0.204069955 | 44.612177046 | Hand grip strength (right) | ukb-b-10215 |
| rs635538 | A | G | 0.913868 | -0.0219943 | 0.00266274 | 1.50E-16 | 0.281668568 | 68.228019400 | Hand grip strength (right) | ukb-b-10215 |
| rs645144 | C | T | 0.329996 | -0.00868793 | 0.00158627 | 4.30E-08 | 0.147046448 | 29.997040161 | Hand grip strength (right) | ukb-b-10215 |
| rs6473015 | C | A | 0.285659 | 0.00959961 | 0.00164629 | 5.50E-09 | 0.163466445 | 34.001219868 | Hand grip strength (right) | ukb-b-10215 |
| rs6592737 | T | A | 0.37154 | -0.00924791 | 0.00154126 | 0.000000002 | 0.171439323 | 36.002725095 | Hand grip strength (right) | ukb-b-10215 |
| rs6693567 | T | C | 0.733032 | -0.00974656 | 0.00167765 | 6.30E-09 | 0.162463089 | 33.752037815 | Hand grip strength (right) | ukb-b-10215 |
| rs6693965 | T | G | 0.12803 | -0.016251 | 0.00223033 | 3.20E-13 | 0.233787758 | 53.091125038 | Hand grip strength (right) | ukb-b-10215 |
| rs6711390 | T | C | 0.37107 | 0.0132663 | 0.00152876 | 4.00E-18 | 0.302058364 | 75.304513370 | Hand grip strength (right) | ukb-b-10215 |
| rs6715064 | T | C | 0.310405 | -0.00921128 | 0.00160563 | 9.60E-09 | 0.159061172 | 32.911601806 | Hand grip strength (right) | ukb-b-10215 |
| rs6792762 | A | G | 0.419501 | -0.00911683 | 0.0015155 | 1.80E-09 | 0.172173369 | 36.188937451 | Hand grip strength (right) | ukb-b-10215 |
| rs6870324 | G | C | 0.269673 | -0.00998568 | 0.00168316 | 3.00E-09 | 0.168247736 | 35.196905801 | Hand grip strength (right) | ukb-b-10215 |
| rs6882168 | T | C | 0.337386 | -0.00909312 | 0.00157605 | 7.90E-09 | 0.160587596 | 33.287858923 | Hand grip strength (right) | ukb-b-10215 |
| rs6962338 | G | A | 0.043971 | -0.0203488 | 0.00362338 | 0.00000002 | 0.153445906 | 31.539139566 | Hand grip strength (right) | ukb-b-10215 |
| rs6977081 | T | G | 0.333818 | 0.01286 | 0.00158867 | 5.70E-16 | 0.273565699 | 65.526134379 | Hand grip strength (right) | ukb-b-10215 |
| rs7034200 | A | C | 0.480149 | 0.00868874 | 0.00148875 | 5.30E-09 | 0.163710789 | 34.061992923 | Hand grip strength (right) | ukb-b-10215 |
| rs71298370 | A | G | 0.086217 | 0.0165994 | 0.00270846 | 8.90E-10 | 0.177543110 | 37.561240648 | Hand grip strength (right) | ukb-b-10215 |
| rs7148603 | A | G | 0.359456 | 0.00930613 | 0.00158837 | 4.70E-09 | 0.164774301 | 34.326923202 | Hand grip strength (right) | ukb-b-10215 |
| rs7196917 | G | A | 0.429932 | -0.010582 | 0.00150287 | 1.90E-12 | 0.221749577 | 49.578420191 | Hand grip strength (right) | ukb-b-10215 |
| rs7206195 | T | C | 0.180373 | -0.0153211 | 0.00193924 | 2.80E-15 | 0.264018528 | 62.418995188 | Hand grip strength (right) | ukb-b-10215 |
| rs721101 | C | T | 0.271238 | 0.00946697 | 0.0016742 | 0.000000016 | 0.155236312 | 31.974762443 | Hand grip strength (right) | ukb-b-10215 |
| rs7214252 | A | G | 0.211101 | -0.0102442 | 0.00182532 | 0.00000002 | 0.153274965 | 31.497644186 | Hand grip strength (right) | ukb-b-10215 |
| rs7249 | T | C | 0.365956 | 0.00848186 | 0.00154442 | 0.00000004 | 0.147733079 | 30.161390919 | Hand grip strength (right) | ukb-b-10215 |
| rs7266065 | A | G | 0.322982 | 0.00993881 | 0.00159481 | 4.60E-10 | 0.182474759 | 38.837465120 | Hand grip strength (right) | ukb-b-10215 |
| rs72820369 | T | A | 0.117642 | 0.0161266 | 0.00232961 | 4.40E-12 | 0.215934596 | 47.920262034 | Hand grip strength (right) | ukb-b-10215 |
| rs72977282 | A | T | 0.413909 | -0.016811 | 0.00151401 | 1.20E-28 | 0.414713888 | 123.290498610 | Hand grip strength (right) | ukb-b-10215 |
| rs7301953 | A | G | 0.312268 | -0.0115397 | 0.00160449 | 6.40E-13 | 0.229156416 | 51.726727987 | Hand grip strength (right) | ukb-b-10215 |
| rs7451021 | C | T | 0.689474 | -0.0157967 | 0.00160573 | 7.70E-23 | 0.357413021 | 96.780463565 | Hand grip strength (right) | ukb-b-10215 |
| rs75457267 | T | C | 0.05122 | -0.0187295 | 0.00339456 | 3.40E-08 | 0.148906429 | 30.442855662 | Hand grip strength (right) | ukb-b-10215 |
| rs7549184 | A | G | 0.787331 | 0.01056 | 0.00181367 | 5.80E-09 | 0.163062808 | 33.900905437 | Hand grip strength (right) | ukb-b-10215 |
| rs7565148 | G | T | 0.500863 | -0.0103511 | 0.00148921 | 3.60E-12 | 0.217318595 | 48.312679054 | Hand grip strength (right) | ukb-b-10215 |
| rs7575451 | G | C | 0.649801 | -0.0106047 | 0.00155571 | 9.30E-12 | 0.210764266 | 46.466449313 | Hand grip strength (right) | ukb-b-10215 |
| rs7576964 | T | G | 0.342052 | 0.00974539 | 0.00156908 | 5.30E-10 | 0.181466165 | 38.575207782 | Hand grip strength (right) | ukb-b-10215 |
| rs7652177 | G | C | 0.504702 | 0.00858128 | 0.00148684 | 7.90E-09 | 0.160677570 | 33.310079806 | Hand grip strength (right) | ukb-b-10215 |
| rs7657558 | G | T | 0.719597 | 0.0107255 | 0.00166228 | 1.10E-10 | 0.193069481 | 41.631948264 | Hand grip strength (right) | ukb-b-10215 |
| rs76749769 | T | C | 0.091199 | 0.0144315 | 0.00257957 | 2.20E-08 | 0.152455047 | 31.298845072 | Hand grip strength (right) | ukb-b-10215 |
| rs76895963 | G | T | 0.020696 | 0.0359771 | 0.00575693 | 4.10E-10 | 0.183307325 | 39.054439479 | Hand grip strength (right) | ukb-b-10215 |
| rs77485342 | T | C | 0.018008 | 0.0353373 | 0.0055898 | 2.60E-10 | 0.186780916 | 39.964482009 | Hand grip strength (right) | ukb-b-10215 |
| rs7790322 | T | C | 0.415619 | -0.00860301 | 0.0015092 | 0.000000012 | 0.157361752 | 32.494305735 | Hand grip strength (right) | ukb-b-10215 |
| rs7871404 | G | A | 0.188654 | 0.0119345 | 0.00190074 | 3.40E-10 | 0.184722306 | 39.424212680 | Hand grip strength (right) | ukb-b-10215 |
| rs7953280 | C | G | 0.506945 | -0.00898631 | 0.00149436 | 1.80E-09 | 0.172067224 | 36.161990219 | Hand grip strength (right) | ukb-b-10215 |
| rs7963801 | C | T | 0.571983 | -0.0112734 | 0.00151048 | 8.40E-14 | 0.242500643 | 55.703165395 | Hand grip strength (right) | ukb-b-10215 |
| rs79723785 | C | T | 0.016495 | -0.0340504 | 0.00602877 | 0.000000016 | 0.154928469 | 31.899729823 | Hand grip strength (right) | ukb-b-10215 |
| rs8055199 | A | G | 0.660123 | -0.00896262 | 0.00157271 | 0.000000012 | 0.157290140 | 32.476758094 | Hand grip strength (right) | ukb-b-10215 |
| rs823130 | T | C | 0.432873 | -0.0123872 | 0.00150332 | 1.70E-16 | 0.280682254 | 67.895881106 | Hand grip strength (right) | ukb-b-10215 |
| rs852520 | A | C | 0.662481 | -0.00890306 | 0.00157301 | 0.000000015 | 0.155480542 | 32.034329129 | Hand grip strength (right) | ukb-b-10215 |
| rs911642 | T | C | 0.375746 | 0.00862996 | 0.00153783 | 0.00000002 | 0.153251907 | 31.492048222 | Hand grip strength (right) | ukb-b-10215 |
| rs9267806 | A | G | 0.255832 | -0.0167434 | 0.00170197 | 7.70E-23 | 0.357410803 | 96.779528956 | Hand grip strength (right) | ukb-b-10215 |
| rs9322822 | T | C | 0.320183 | 0.0110799 | 0.00159266 | 3.50E-12 | 0.217618127 | 48.397790568 | Hand grip strength (right) | ukb-b-10215 |
| rs935728 | T | C | 0.327604 | 0.00955337 | 0.0015872 | 1.80E-09 | 0.172329003 | 36.228461114 | Hand grip strength (right) | ukb-b-10215 |
| rs9388051 | A | G | 0.186051 | 0.0106262 | 0.0019116 | 2.70E-08 | 0.150806288 | 30.900245343 | Hand grip strength (right) | ukb-b-10215 |
| rs9396861 | A | C | 0.598901 | -0.00962972 | 0.00155347 | 5.70E-10 | 0.180889999 | 38.425681392 | Hand grip strength (right) | ukb-b-10215 |
| rs9639938 | G | C | 0.540731 | 0.00873031 | 0.0014948 | 5.20E-09 | 0.163907312 | 34.110897931 | Hand grip strength (right) | ukb-b-10215 |
| rs9652468 | A | G | 0.249359 | -0.0125308 | 0.00172118 | 3.30E-13 | 0.233492187 | 53.003556923 | Hand grip strength (right) | ukb-b-10215 |
| rs9757079 | T | C | 0.318301 | 0.00980223 | 0.00159699 | 8.40E-10 | 0.177982465 | 37.674316663 | Hand grip strength (right) | ukb-b-10215 |
| rs9853018 | T | C | 0.443249 | 0.0101977 | 0.00149426 | 8.80E-12 | 0.211152384 | 46.574920394 | Hand grip strength (right) | ukb-b-10215 |
| rs997850 | C | G | 0.60473 | -0.0090224 | 0.00153019 | 3.70E-09 | 0.166530536 | 34.765896712 | Hand grip strength (right) | ukb-b-10215 |

Supplementary Table 7: Information on all instrumental variables (IVs) ultimately used for Low hand grip strength (60 years and older) (EWGSOP) in our study

| SNPs | effect_allele.exposure | other_allele.exposure | eaf.exposure | beta.exposure | se.exposure | pval.exposure | R^2^ | F | trait | id.exposure |
| --- | --- | --- | --- | --- | --- | --- | --- | --- | --- | --- |
| rs10846071 | T | C | 0.3917 | 0.0433 | 0.0075 | 7.32E-09 | 0.662238533 | 33.33137778 | Low hand grip strength (60 years and older) (EWGSOP) | ebi-a-GCST90007526 |
| rs10952289 | C | T | 0.3436 | -0.0435 | 0.0078 | 2.10E-08 | 0.646584863 | 31.10207101 | Low hand grip strength (60 years and older) (EWGSOP) | ebi-a-GCST90007526 |
| rs11236213 | A | G | 0.3136 | -0.0504 | 0.008 | 3.01E-10 | 0.700123479 | 39.69 | Low hand grip strength (60 years and older) (EWGSOP) | ebi-a-GCST90007526 |
| rs12140813 | T | C | 0.1856 | 0.0511 | 0.0094 | 4.76E-08 | 0.63481656 | 29.55194658 | Low hand grip strength (60 years and older) (EWGSOP) | ebi-a-GCST90007526 |
| rs13107325 | T | C | 0.0739 | 0.0897 | 0.0138 | 7.42E-11 | 0.713080169 | 42.25 | Low hand grip strength (60 years and older) (EWGSOP) | ebi-a-GCST90007526 |
| rs143384 | G | A | 0.4088 | -0.0545 | 0.0075 | 4.47E-13 | 0.756462498 | 52.80444444 | Low hand grip strength (60 years and older) (EWGSOP) | ebi-a-GCST90007526 |
| rs143459567 | T | C | 0.0386 | 0.1185 | 0.0189 | 3.41E-10 | 0.698104681 | 39.31090955 | Low hand grip strength (60 years and older) (EWGSOP) | ebi-a-GCST90007526 |
| rs185320691 | C | G | 0.1047 | 0.0913 | 0.0146 | 3.84E-10 | 0.696998431 | 39.10531995 | Low hand grip strength (60 years and older) (EWGSOP) | ebi-a-GCST90007526 |
| rs2899611 | G | T | 0.5049 | 0.0431 | 0.0074 | 6.01E-09 | 0.666161024 | 33.92275383 | Low hand grip strength (60 years and older) (EWGSOP) | ebi-a-GCST90007526 |
| rs3118903 | A | G | 0.2185 | 0.0575 | 0.0088 | 6.71E-11 | 0.715215901 | 42.69434401 | Low hand grip strength (60 years and older) (EWGSOP) | ebi-a-GCST90007526 |
| rs34415150 | G | A | 0.178 | 0.0833 | 0.0099 | 4.42E-17 | 0.806373227 | 70.79777574 | Low hand grip strength (60 years and older) (EWGSOP) | ebi-a-GCST90007526 |
| rs34464763 | A | T | 0.3943 | 0.0544 | 0.0086 | 3.15E-10 | 0.701822287 | 40.01297999 | Low hand grip strength (60 years and older) (EWGSOP) | ebi-a-GCST90007526 |
| rs62102286 | G | T | 0.4359 | -0.0487 | 0.0074 | 5.49E-11 | 0.718125967 | 43.3106282 | Low hand grip strength (60 years and older) (EWGSOP) | ebi-a-GCST90007526 |
| rs7624084 | C | T | 0.4423 | -0.0428 | 0.0074 | 8.51E-09 | 0.663047098 | 33.45215486 | Low hand grip strength (60 years and older) (EWGSOP) | ebi-a-GCST90007526 |
| rs79723785 | C | T | 0.0165 | 0.1674 | 0.0293 | 1.16E-08 | 0.657547477 | 32.64191779 | Low hand grip strength (60 years and older) (EWGSOP) | ebi-a-GCST90007526 |
| rs8061064 | A | T | 0.4627 | 0.0407 | 0.0074 | 3.55E-08 | 0.64021164 | 30.25 | Low hand grip strength (60 years and older) (EWGSOP) | ebi-a-GCST90007526 |
| rs958685 | A | C | 0.5142 | -0.0428 | 0.0074 | 6.52E-09 | 0.663047098 | 33.45215486 | Low hand grip strength (60 years and older) (EWGSOP) | ebi-a-GCST90007526 |

Supplementary Table 8: Information on all instrumental variables (IVs) ultimately used for Body fat percentage in our study

| SNPs | effect_allele.exposure | other_allele.exposure | eaf.exposure | beta.exposure | se.exposure | pval.exposure | R^2^ | F | trait | id.exposure |
| --- | --- | --- | --- | --- | --- | --- | --- | --- | --- | --- |
| rs10050620 | T | C | 0.325382 | -0.0103073 | 0.00164616 | 3.80E-10 | 0.091344117 | 39.2053869 | Body fat percentage | ukb-b-8909 |
| rs10100245 | A | G | 0.564497 | 0.0152146 | 0.00155645 | 1.40E-22 | 0.196794442 | 95.55440893 | Body fat percentage | ukb-b-8909 |
| rs10116857 | A | C | 0.061826 | -0.0219296 | 0.00320576 | 7.90E-12 | 0.107132627 | 46.7949954 | Body fat percentage | ukb-b-8909 |
| rs1013293 | A | G | 0.430286 | -0.0139271 | 0.00155866 | 4.10E-19 | 0.169929565 | 79.83964688 | Body fat percentage | ukb-b-8909 |
| rs10144067 | T | C | 0.59128 | 0.0100925 | 0.00158231 | 1.80E-10 | 0.094461863 | 40.68313114 | Body fat percentage | ukb-b-8909 |
| rs10146997 | G | A | 0.221871 | 0.018731 | 0.00185697 | 6.30E-24 | 0.206905669 | 101.7447836 | Body fat percentage | ukb-b-8909 |
| rs10175266 | G | A | 0.368852 | 0.0125399 | 0.00159364 | 3.60E-15 | 0.137009072 | 61.9166859 | Body fat percentage | ukb-b-8909 |
| rs10187101 | T | C | 0.363611 | -0.010248 | 0.00159844 | 1.40E-10 | 0.095346194 | 41.104139 | Body fat percentage | ukb-b-8909 |
| rs10209821 | T | C | 0.342612 | 0.0123638 | 0.0016188 | 2.20E-14 | 0.130111721 | 58.33343501 | Body fat percentage | ukb-b-8909 |
| rs10245306 | C | G | 0.685981 | 0.00917484 | 0.00167492 | 4.30E-08 | 0.071441952 | 30.00605229 | Body fat percentage | ukb-b-8909 |
| rs10259620 | G | A | 0.787159 | -0.0139877 | 0.00189659 | 1.60E-13 | 0.122399084 | 54.3933373 | Body fat percentage | ukb-b-8909 |
| rs10271582 | G | A | 0.154048 | -0.0138378 | 0.00213519 | 9.10E-11 | 0.097224598 | 42.0011368 | Body fat percentage | ukb-b-8909 |
| rs1038088 | G | T | 0.519335 | 0.0125471 | 0.00154189 | 4.00E-16 | 0.14514663 | 66.21859091 | Body fat percentage | ukb-b-8909 |
| rs10423928 | A | T | 0.194436 | -0.0232502 | 0.00194747 | 7.40E-33 | 0.267649397 | 142.531821 | Body fat percentage | ukb-b-8909 |
| rs10496731 | G | T | 0.374905 | -0.0115311 | 0.00158686 | 3.70E-13 | 0.119248527 | 52.80368734 | Body fat percentage | ukb-b-8909 |
| rs10505836 | C | A | 0.860003 | 0.0128411 | 0.00223802 | 9.60E-09 | 0.077842541 | 32.92126613 | Body fat percentage | ukb-b-8909 |
| rs10510025 | T | C | 0.24708 | 0.0129238 | 0.00179232 | 5.60E-13 | 0.117634155 | 51.99353612 | Body fat percentage | ukb-b-8909 |
| rs10513935 | A | G | 0.301501 | -0.00942576 | 0.00168416 | 2.20E-08 | 0.074344816 | 31.3231953 | Body fat percentage | ukb-b-8909 |
| rs1056441 | C | T | 0.675301 | 0.0120757 | 0.0016495 | 2.50E-13 | 0.120818619 | 53.59447125 | Body fat percentage | ukb-b-8909 |
| rs10756798 | T | C | 0.642437 | -0.0140265 | 0.00160866 | 2.80E-18 | 0.163139324 | 76.02739429 | Body fat percentage | ukb-b-8909 |
| rs1078455 | C | T | 0.309511 | 0.00964481 | 0.00167852 | 9.10E-09 | 0.07805069 | 33.01674933 | Body fat percentage | ukb-b-8909 |
| rs10788497 | C | G | 0.499616 | 0.0103873 | 0.00154427 | 1.70E-11 | 0.103950434 | 45.24377984 | Body fat percentage | ukb-b-8909 |
| rs10799778 | G | T | 0.833671 | -0.0130789 | 0.00206635 | 2.50E-10 | 0.093154408 | 40.06218843 | Body fat percentage | ukb-b-8909 |
| rs10854853 | T | G | 0.456784 | 0.0104441 | 0.00155077 | 1.60E-11 | 0.104184095 | 45.35730698 | Body fat percentage | ukb-b-8909 |
| rs1086103 | A | C | 0.045775 | -0.021384 | 0.00368848 | 6.70E-09 | 0.079344289 | 33.61112319 | Body fat percentage | ukb-b-8909 |
| rs10867315 | A | G | 0.190594 | -0.0111522 | 0.00196791 | 0.000000015 | 0.076081601 | 32.11520018 | Body fat percentage | ukb-b-8909 |
| rs10896012 | C | T | 0.217394 | 0.0115223 | 0.00187372 | 7.80E-10 | 0.088391913 | 37.81542382 | Body fat percentage | ukb-b-8909 |
| rs10938397 | G | A | 0.4343 | 0.0201696 | 0.00155596 | 2.00E-38 | 0.301118119 | 168.0342124 | Body fat percentage | ukb-b-8909 |
| rs10959841 | C | T | 0.387368 | -0.00911255 | 0.00158643 | 9.20E-09 | 0.078001612 | 32.99423176 | Body fat percentage | ukb-b-8909 |
| rs10999460 | T | C | 0.265521 | 0.016885 | 0.00175383 | 6.10E-22 | 0.192025959 | 92.68877464 | Body fat percentage | ukb-b-8909 |
| rs11012732 | G | A | 0.331719 | 0.0166039 | 0.00163819 | 3.80E-24 | 0.208489334 | 102.7286728 | Body fat percentage | ukb-b-8909 |
| rs11022718 | T | C | 0.202968 | -0.0107504 | 0.00193009 | 0.000000025 | 0.073686435 | 31.02373816 | Body fat percentage | ukb-b-8909 |
| rs11030016 | T | C | 0.739777 | 0.0132564 | 0.001761 | 5.20E-14 | 0.126866901 | 56.66729578 | Body fat percentage | ukb-b-8909 |
| rs11030108 | G | A | 0.679962 | -0.0185028 | 0.00165098 | 3.80E-29 | 0.243600388 | 125.6004758 | Body fat percentage | ukb-b-8909 |
| rs11062595 | G | C | 0.09222 | -0.0153041 | 0.00266658 | 9.50E-09 | 0.077880537 | 32.93869241 | Body fat percentage | ukb-b-8909 |
| rs11079849 | T | C | 0.328562 | -0.0130416 | 0.00164632 | 2.30E-15 | 0.138602824 | 62.75281946 | Body fat percentage | ukb-b-8909 |
| rs11105842 | A | G | 0.367542 | -0.0101643 | 0.00160647 | 2.50E-10 | 0.093091218 | 40.03222326 | Body fat percentage | ukb-b-8909 |
| rs11119208 | G | A | 0.614902 | -0.00965759 | 0.00157945 | 9.70E-10 | 0.087479039 | 37.38744299 | Body fat percentage | ukb-b-8909 |
| rs11122450 | G | T | 0.611689 | -0.0101571 | 0.00157943 | 1.30E-10 | 0.095874438 | 41.35601563 | Body fat percentage | ukb-b-8909 |
| rs11129660 | T | C | 0.209783 | 0.0110275 | 0.00189369 | 5.80E-09 | 0.079994847 | 33.91066928 | Body fat percentage | ukb-b-8909 |
| rs11150745 | G | A | 0.317696 | -0.0126783 | 0.00166019 | 2.20E-14 | 0.130082773 | 58.31851556 | Body fat percentage | ukb-b-8909 |
| rs11165643 | T | C | 0.590144 | 0.0146762 | 0.00156269 | 5.90E-21 | 0.184446156 | 88.20263852 | Body fat percentage | ukb-b-8909 |
| rs11205303 | C | T | 0.406646 | 0.018328 | 0.00156672 | 1.30E-31 | 0.259752493 | 136.8508116 | Body fat percentage | ukb-b-8909 |
| rs11208779 | C | G | 0.528878 | 0.010317 | 0.00154445 | 2.40E-11 | 0.102670673 | 44.62304013 | Body fat percentage | ukb-b-8909 |
| rs11222371 | T | C | 0.409234 | 0.0118153 | 0.00156853 | 5.00E-14 | 0.127012705 | 56.74189652 | Body fat percentage | ukb-b-8909 |
| rs11245344 | T | C | 0.571137 | 0.00937385 | 0.00156054 | 1.90E-09 | 0.084682442 | 36.08163337 | Body fat percentage | ukb-b-8909 |
| rs112710809 | T | C | 0.063535 | 0.0210464 | 0.00319585 | 4.50E-11 | 0.100074788 | 43.3693453 | Body fat percentage | ukb-b-8909 |
| rs112852122 | A | G | 0.157829 | -0.0164403 | 0.00215064 | 2.10E-14 | 0.130311608 | 58.43647874 | Body fat percentage | ukb-b-8909 |
| rs11343 | G | T | 0.561331 | 0.00867343 | 0.00158474 | 4.40E-08 | 0.071328521 | 29.95475113 | Body fat percentage | ukb-b-8909 |
| rs113503736 | G | T | 0.223372 | -0.0102974 | 0.00185568 | 2.90E-08 | 0.073178029 | 30.79278659 | Body fat percentage | ukb-b-8909 |
| rs113941571 | T | C | 0.078721 | 0.0209909 | 0.00297573 | 1.70E-12 | 0.113151404 | 49.75939285 | Body fat percentage | ukb-b-8909 |
| rs114295766 | T | A | 0.069527 | -0.0195859 | 0.00321452 | 1.10E-09 | 0.086916218 | 37.12400318 | Body fat percentage | ukb-b-8909 |
| rs11538 | G | A | 0.171838 | 0.0121586 | 0.00204591 | 2.80E-09 | 0.083038691 | 35.31783652 | Body fat percentage | ukb-b-8909 |
| rs11619722 | C | T | 0.300933 | -0.0108221 | 0.00168444 | 1.30E-10 | 0.095709561 | 41.27736763 | Body fat percentage | ukb-b-8909 |
| rs11664106 | T | A | 0.373948 | 0.010994 | 0.00163476 | 1.80E-11 | 0.103917105 | 45.2275914 | Body fat percentage | ukb-b-8909 |
| rs11664848 | G | C | 0.659616 | 0.00965173 | 0.00163558 | 3.60E-09 | 0.081970707 | 34.82304516 | Body fat percentage | ukb-b-8909 |
| rs11666808 | C | T | 0.625293 | -0.0177091 | 0.00160075 | 1.90E-28 | 0.238861034 | 122.390007 | Body fat percentage | ukb-b-8909 |
| rs11677541 | A | G | 0.535271 | -0.00923656 | 0.00154968 | 2.50E-09 | 0.083485524 | 35.52519375 | Body fat percentage | ukb-b-8909 |
| rs117176448 | G | C | 0.096175 | 0.0172978 | 0.00261256 | 3.60E-11 | 0.101046613 | 43.83784496 | Body fat percentage | ukb-b-8909 |
| rs11782074 | T | G | 0.383624 | 0.0106691 | 0.00161125 | 3.60E-11 | 0.101063455 | 43.84597286 | Body fat percentage | ukb-b-8909 |
| rs11786089 | G | A | 0.459661 | 0.0112967 | 0.00154993 | 3.10E-13 | 0.119882325 | 53.12256326 | Body fat percentage | ukb-b-8909 |
| rs11852419 | T | A | 0.261669 | 0.0106498 | 0.00175622 | 1.30E-09 | 0.08616452 | 36.77266172 | Body fat percentage | ukb-b-8909 |
| rs11855853 | T | C | 0.269414 | -0.0103933 | 0.00174766 | 2.70E-09 | 0.083143762 | 35.36657747 | Body fat percentage | ukb-b-8909 |
| rs11866219 | C | A | 0.583787 | -0.0158787 | 0.00158501 | 1.30E-23 | 0.204667917 | 100.3612066 | Body fat percentage | ukb-b-8909 |
| rs12042959 | G | A | 0.143917 | -0.0145771 | 0.00220294 | 3.70E-11 | 0.100939519 | 43.78616696 | Body fat percentage | ukb-b-8909 |
| rs12053559 | G | T | 0.471201 | 0.00856437 | 0.00154317 | 2.90E-08 | 0.073195882 | 30.80089239 | Body fat percentage | ukb-b-8909 |
| rs12072739 | G | A | 0.224584 | 0.0134145 | 0.00184627 | 3.70E-13 | 0.119222968 | 52.79083773 | Body fat percentage | ukb-b-8909 |
| rs12103006 | G | A | 0.569085 | 0.0104802 | 0.00155885 | 1.80E-11 | 0.103858591 | 45.19917279 | Body fat percentage | ukb-b-8909 |
| rs1229984 | C | T | 0.972781 | 0.0310059 | 0.00467505 | 3.30E-11 | 0.101353855 | 43.98617158 | Body fat percentage | ukb-b-8909 |
| rs12375196 | A | C | 0.424277 | 0.0102996 | 0.00156914 | 5.20E-11 | 0.099482127 | 43.08413026 | Body fat percentage | ukb-b-8909 |
| rs12376870 | A | G | 0.237663 | -0.0110309 | 0.00181676 | 1.30E-09 | 0.086364477 | 36.86606431 | Body fat percentage | ukb-b-8909 |
| rs12402939 | C | A | 0.392363 | -0.0101759 | 0.00157772 | 1.10E-10 | 0.096384032 | 41.59927858 | Body fat percentage | ukb-b-8909 |
| rs12419272 | G | C | 0.056932 | 0.0206723 | 0.00333136 | 5.50E-10 | 0.089862193 | 38.5065371 | Body fat percentage | ukb-b-8909 |
| rs12432026 | G | T | 0.540113 | 0.00996462 | 0.00154621 | 1.20E-10 | 0.096243489 | 41.53216089 | Body fat percentage | ukb-b-8909 |
| rs12441543 | A | G | 0.287112 | 0.0114053 | 0.0017089 | 2.50E-11 | 0.102505531 | 44.54306803 | Body fat percentage | ukb-b-8909 |
| rs12459965 | T | C | 0.267624 | -0.0126806 | 0.00174938 | 4.20E-13 | 0.118728844 | 52.54256768 | Body fat percentage | ukb-b-8909 |
| rs12462975 | A | G | 0.32959 | 0.0123462 | 0.00165332 | 8.20E-14 | 0.125097298 | 55.76385345 | Body fat percentage | ukb-b-8909 |
| rs12475388 | A | G | 0.485685 | -0.00988584 | 0.00154662 | 1.60E-10 | 0.094825989 | 40.85638249 | Body fat percentage | ukb-b-8909 |
| rs12538435 | G | A | 0.261821 | -0.0109846 | 0.00175319 | 3.70E-10 | 0.091452112 | 39.25640487 | Body fat percentage | ukb-b-8909 |
| rs12619178 | T | C | 0.400829 | -0.0144544 | 0.0015682 | 3.00E-20 | 0.178872382 | 84.95662255 | Body fat percentage | ukb-b-8909 |
| rs12628603 | A | G | 0.617054 | -0.00959714 | 0.00159107 | 1.60E-09 | 0.085330569 | 36.38355123 | Body fat percentage | ukb-b-8909 |
| rs12639116 | T | C | 0.096558 | -0.0161252 | 0.00262251 | 7.80E-10 | 0.088374649 | 37.80732181 | Body fat percentage | ukb-b-8909 |
| rs12658319 | T | C | 0.299121 | 0.0107194 | 0.0016852 | 2.00E-10 | 0.093994861 | 40.46113427 | Body fat percentage | ukb-b-8909 |
| rs12670456 | G | A | 0.301754 | 0.00941089 | 0.00168003 | 0.000000021 | 0.074465534 | 31.37814897 | Body fat percentage | ukb-b-8909 |
| rs12724928 | C | T | 0.20486 | -0.0146759 | 0.00190484 | 1.30E-14 | 0.132098613 | 59.35980739 | Body fat percentage | ukb-b-8909 |
| rs1284373 | T | C | 0.199214 | -0.0108576 | 0.00192322 | 0.000000016 | 0.07554905 | 31.87203125 | Body fat percentage | ukb-b-8909 |
| rs12890931 | G | T | 0.362416 | 0.0120832 | 0.00161524 | 7.40E-14 | 0.125485163 | 55.96155915 | Body fat percentage | ukb-b-8909 |
| rs12926311 | C | G | 0.353546 | -0.00918915 | 0.00161862 | 1.40E-08 | 0.076332897 | 32.23004241 | Body fat percentage | ukb-b-8909 |
| rs12926311 | C | G | 0.353546 | -0.00918915 | 0.00161862 | 1.40E-08 | 0.076332897 | 32.23004241 | Body fat percentage | ukb-b-8909 |
| rs13026103 | A | G | 0.74098 | 0.00961952 | 0.00175567 | 4.30E-08 | 0.071474367 | 30.02071471 | Body fat percentage | ukb-b-8909 |
| rs13064797 | A | G | 0.205187 | -0.0108506 | 0.00190707 | 1.30E-08 | 0.076644105 | 32.37235087 | Body fat percentage | ukb-b-8909 |
| rs13107325 | T | C | 0.074871 | 0.0323745 | 0.00292921 | 2.10E-28 | 0.238509217 | 122.1532773 | Body fat percentage | ukb-b-8909 |
| rs13132853 | G | A | 0.360225 | -0.00936528 | 0.00161107 | 6.10E-09 | 0.079737029 | 33.79190778 | Body fat percentage | ukb-b-8909 |
| rs13174863 | G | A | 0.14814 | 0.0128832 | 0.00218539 | 3.70E-09 | 0.081818848 | 34.7527835 | Body fat percentage | ukb-b-8909 |
| rs1318408 | G | A | 0.119506 | 0.0157919 | 0.00237893 | 3.20E-11 | 0.101519505 | 44.06618407 | Body fat percentage | ukb-b-8909 |
| rs1322842 | G | A | 0.609473 | -0.0114888 | 0.00158746 | 4.60E-13 | 0.118399767 | 52.3773783 | Body fat percentage | ukb-b-8909 |
| rs13249935 | C | T | 0.366657 | 0.00954133 | 0.00161413 | 3.40E-09 | 0.082226486 | 34.94144127 | Body fat percentage | ukb-b-8909 |
| rs13292699 | C | A | 0.433725 | -0.0200044 | 0.0015583 | 1.00E-37 | 0.297039995 | 164.7968547 | Body fat percentage | ukb-b-8909 |
| rs13389219 | T | C | 0.392441 | 0.0170899 | 0.00157313 | 1.70E-27 | 0.232311309 | 118.0184255 | Body fat percentage | ukb-b-8909 |
| rs13408397 | T | C | 0.411055 | -0.0130723 | 0.0015614 | 5.70E-17 | 0.152345588 | 70.09316361 | Body fat percentage | ukb-b-8909 |
| rs13436840 | T | C | 0.265069 | 0.0136415 | 0.0017496 | 6.30E-15 | 0.134856063 | 60.79203963 | Body fat percentage | ukb-b-8909 |
| rs1350429 | G | A | 0.480306 | 0.00973719 | 0.00154554 | 3.00E-10 | 0.092373877 | 39.69234795 | Body fat percentage | ukb-b-8909 |
| rs1377184 | T | A | 0.748963 | 0.0127442 | 0.00178122 | 8.40E-13 | 0.116028266 | 51.19057775 | Body fat percentage | ukb-b-8909 |
| rs1421334 | C | A | 0.54884 | -0.0113003 | 0.00155717 | 4.00E-13 | 0.11896916 | 52.66327827 | Body fat percentage | ukb-b-8909 |
| rs1436348 | G | A | 0.582718 | 0.0107963 | 0.00156132 | 4.70E-12 | 0.109213217 | 47.81520719 | Body fat percentage | ukb-b-8909 |
| rs1441264 | A | G | 0.59371 | 0.0119959 | 0.00160493 | 7.80E-14 | 0.125299226 | 55.86675975 | Body fat percentage | ukb-b-8909 |
| rs1453055 | A | G | 0.273355 | 0.0126532 | 0.00173398 | 2.90E-13 | 0.120133652 | 53.24913747 | Body fat percentage | ukb-b-8909 |
| rs1454687 | G | C | 0.51535 | -0.0135392 | 0.00153973 | 1.50E-18 | 0.165455674 | 77.32089351 | Body fat percentage | ukb-b-8909 |
| rs1456014 | G | A | 0.205602 | -0.0150933 | 0.00190234 | 2.10E-15 | 0.138976819 | 62.94947741 | Body fat percentage | ukb-b-8909 |
| rs1469084 | G | A | 0.545742 | 0.00878996 | 0.00154865 | 1.40E-08 | 0.076301467 | 32.21567563 | Body fat percentage | ukb-b-8909 |
| rs1475860 | C | G | 0.505368 | 0.00875321 | 0.00155205 | 0.000000017 | 0.075406616 | 31.80704178 | Body fat percentage | ukb-b-8909 |
| rs149380583 | C | G | 0.128655 | -0.0127741 | 0.00231146 | 3.30E-08 | 0.072623835 | 30.54132388 | Body fat percentage | ukb-b-8909 |
| rs1503526 | C | T | 0.47999 | 0.0113767 | 0.00154191 | 1.60E-13 | 0.122490345 | 54.43955443 | Body fat percentage | ukb-b-8909 |
| rs1559900 | T | C | 0.286141 | 0.00986943 | 0.00170532 | 7.10E-09 | 0.079090566 | 33.49441301 | Body fat percentage | ukb-b-8909 |
| rs1568488 | C | G | 0.594863 | 0.0117289 | 0.00158106 | 1.20E-13 | 0.123659164 | 55.03232539 | Body fat percentage | ukb-b-8909 |
| rs1605898 | A | T | 0.837607 | 0.0129238 | 0.00209124 | 6.40E-10 | 0.089193689 | 38.19202647 | Body fat percentage | ukb-b-8909 |
| rs1624064 | C | T | 0.420353 | -0.0106391 | 0.00156343 | 1.00E-11 | 0.10613535 | 46.30766678 | Body fat percentage | ukb-b-8909 |
| rs16916303 | G | A | 0.11977 | -0.0143754 | 0.00240082 | 2.10E-09 | 0.084190167 | 35.8526016 | Body fat percentage | ukb-b-8909 |
| rs16934748 | C | T | 0.150341 | 0.0128932 | 0.00215901 | 2.30E-09 | 0.083781226 | 35.66252846 | Body fat percentage | ukb-b-8909 |
| rs16996657 | C | T | 0.127757 | 0.0140368 | 0.00231882 | 1.40E-09 | 0.085888807 | 36.64393878 | Body fat percentage | ukb-b-8909 |
| rs17016133 | C | T | 0.130053 | -0.0137367 | 0.00229121 | 0.000000002 | 0.084388215 | 35.94471422 | Body fat percentage | ukb-b-8909 |
| rs17024393 | C | T | 0.025909 | 0.0474389 | 0.00485686 | 1.60E-22 | 0.196542455 | 95.40212543 | Body fat percentage | ukb-b-8909 |
| rs17055384 | T | C | 0.182952 | -0.0123469 | 0.00200425 | 7.30E-10 | 0.088678639 | 37.95002592 | Body fat percentage | ukb-b-8909 |
| rs17172722 | T | C | 0.418942 | -0.010769 | 0.0015641 | 5.80E-12 | 0.108377279 | 47.40473486 | Body fat percentage | ukb-b-8909 |
| rs17193211 | T | C | 0.066803 | -0.0180452 | 0.00314532 | 9.60E-09 | 0.077828856 | 32.91498994 | Body fat percentage | ukb-b-8909 |
| rs1724557 | A | C | 0.586755 | -0.0110497 | 0.0015726 | 2.10E-12 | 0.112365732 | 49.37014826 | Body fat percentage | ukb-b-8909 |
| rs17522122 | T | G | 0.471197 | 0.0126085 | 0.00155155 | 4.40E-16 | 0.144808502 | 66.03821019 | Body fat percentage | ukb-b-8909 |
| rs17639996 | A | G | 0.150071 | -0.0143309 | 0.00216477 | 3.60E-11 | 0.101020307 | 43.82514984 | Body fat percentage | ukb-b-8909 |
| rs17681686 | C | G | 0.30483 | 0.0136226 | 0.00165383 | 1.80E-16 | 0.148189275 | 67.84819181 | Body fat percentage | ukb-b-8909 |
| rs17704028 | T | C | 0.147192 | -0.0146575 | 0.00217921 | 1.70E-11 | 0.103942447 | 45.23990033 | Body fat percentage | ukb-b-8909 |
| rs17770336 | T | C | 0.322448 | 0.0154638 | 0.00164622 | 5.80E-21 | 0.184506821 | 88.23821235 | Body fat percentage | ukb-b-8909 |
| rs17820010 | G | T | 0.295596 | 0.0124473 | 0.00168847 | 1.70E-13 | 0.12230461 | 54.34550345 | Body fat percentage | ukb-b-8909 |
| rs1782508 | G | C | 0.655489 | -0.00913519 | 0.00162015 | 0.000000017 | 0.075374746 | 31.79250284 | Body fat percentage | ukb-b-8909 |
| rs1787013 | C | T | 0.450716 | 0.00961178 | 0.00155008 | 5.60E-10 | 0.089742672 | 38.45027216 | Body fat percentage | ukb-b-8909 |
| rs1799923 | G | A | 0.886606 | 0.0140939 | 0.00242625 | 6.30E-09 | 0.079632094 | 33.74358916 | Body fat percentage | ukb-b-8909 |
| rs1801282 | G | C | 0.119535 | 0.0301151 | 0.0023675 | 4.60E-37 | 0.293227014 | 161.8037728 | Body fat percentage | ukb-b-8909 |
| rs1808629 | A | G | 0.685441 | -0.01632 | 0.00166697 | 1.20E-22 | 0.197280422 | 95.84837202 | Body fat percentage | ukb-b-8909 |
| rs1813039 | A | G | 0.709746 | 0.0103333 | 0.00170753 | 1.40E-09 | 0.085841678 | 36.62194373 | Body fat percentage | ukb-b-8909 |
| rs1861410 | T | C | 0.555344 | -0.0135852 | 0.0015523 | 2.10E-18 | 0.164151141 | 76.59153226 | Body fat percentage | ukb-b-8909 |
| rs1881505 | C | T | 0.943033 | -0.0191979 | 0.00335242 | 0.00000001 | 0.077564337 | 32.79371397 | Body fat percentage | ukb-b-8909 |
| rs1893659 | A | C | 0.460098 | -0.0163989 | 0.00155688 | 6.10E-26 | 0.221475954 | 110.9479177 | Body fat percentage | ukb-b-8909 |
| rs1893659 | A | C | 0.460098 | -0.0163989 | 0.00155688 | 6.10E-26 | 0.221475954 | 110.9479177 | Body fat percentage | ukb-b-8909 |
| rs1906252 | A | C | 0.484388 | -0.0161687 | 0.00154706 | 1.40E-25 | 0.218794569 | 109.2284803 | Body fat percentage | ukb-b-8909 |
| rs1945160 | A | G | 0.375871 | -0.00962166 | 0.0016001 | 1.80E-09 | 0.084846709 | 36.15811336 | Body fat percentage | ukb-b-8909 |
| rs1964675 | T | C | 0.711087 | 0.0096786 | 0.00170107 | 1.30E-08 | 0.07664516 | 32.3728334 | Body fat percentage | ukb-b-8909 |
| rs1991002 | G | T | 0.495176 | -0.0100728 | 0.00154583 | 7.20E-11 | 0.098181873 | 42.45970368 | Body fat percentage | ukb-b-8909 |
| rs2002023 | T | C | 0.409059 | 0.0102433 | 0.00157002 | 6.80E-11 | 0.098404824 | 42.56664436 | Body fat percentage | ukb-b-8909 |
| rs2008018 | A | G | 0.322077 | 0.0110009 | 0.00164659 | 2.40E-11 | 0.102697476 | 44.63602238 | Body fat percentage | ukb-b-8909 |
| rs2034946 | G | T | 0.208834 | 0.0119487 | 0.00190099 | 3.30E-10 | 0.091983664 | 39.50769115 | Body fat percentage | ukb-b-8909 |
| rs2108635 | G | A | 0.338522 | 0.00983786 | 0.00163453 | 1.80E-09 | 0.084991592 | 36.22559137 | Body fat percentage | ukb-b-8909 |
| rs2111281 | C | A | 0.366942 | 0.0115537 | 0.00160056 | 5.30E-13 | 0.117861131 | 52.10726216 | Body fat percentage | ukb-b-8909 |
| rs215669 | A | G | 0.611659 | -0.012096 | 0.00158845 | 2.60E-14 | 0.129440533 | 57.98777676 | Body fat percentage | ukb-b-8909 |
| rs2165991 | G | A | 0.253707 | 0.0117936 | 0.00178131 | 3.60E-11 | 0.101039205 | 43.83426974 | Body fat percentage | ukb-b-8909 |
| rs2172131 | C | T | 0.578543 | -0.0110832 | 0.00156255 | 1.30E-12 | 0.114262313 | 50.31094724 | Body fat percentage | ukb-b-8909 |
| rs217672 | C | A | 0.27173 | 0.0129295 | 0.00173907 | 1.00E-13 | 0.124136915 | 55.27507421 | Body fat percentage | ukb-b-8909 |
| rs2178899 | T | A | 0.128827 | -0.0191217 | 0.002297 | 8.50E-17 | 0.15088112 | 69.29964505 | Body fat percentage | ukb-b-8909 |
| rs2182717 | A | G | 0.691021 | 0.010121 | 0.00167361 | 1.50E-09 | 0.085732773 | 36.57112543 | Body fat percentage | ukb-b-8909 |
| rs2190788 | T | G | 0.319702 | 0.0110461 | 0.00166011 | 2.90E-11 | 0.101948501 | 44.27353606 | Body fat percentage | ukb-b-8909 |
| rs2192527 | G | A | 0.465502 | 0.0141172 | 0.00154567 | 6.60E-20 | 0.176204965 | 83.4187311 | Body fat percentage | ukb-b-8909 |
| rs2192649 | G | T | 0.500777 | 0.00852919 | 0.00155109 | 3.80E-08 | 0.071952699 | 30.23720068 | Body fat percentage | ukb-b-8909 |
| rs2243928 | G | C | 0.648983 | -0.0120426 | 0.00163641 | 1.90E-13 | 0.121932522 | 54.15720878 | Body fat percentage | ukb-b-8909 |
| rs2274224 | C | G | 0.435459 | -0.0159065 | 0.00155477 | 1.40E-24 | 0.211593585 | 104.6687302 | Body fat percentage | ukb-b-8909 |
| rs2291127 | T | C | 0.156234 | -0.0137926 | 0.00211992 | 7.70E-11 | 0.097912349 | 42.33049417 | Body fat percentage | ukb-b-8909 |
| rs2307111 | C | T | 0.39509 | -0.0171461 | 0.00157693 | 1.50E-27 | 0.232621928 | 118.2240611 | Body fat percentage | ukb-b-8909 |
| rs2371767 | C | G | 0.277759 | 0.0117998 | 0.00171613 | 6.20E-12 | 0.108116604 | 47.27689285 | Body fat percentage | ukb-b-8909 |
| rs240999 | T | G | 0.840258 | -0.0120088 | 0.00210945 | 0.000000012 | 0.076723422 | 32.40863594 | Body fat percentage | ukb-b-8909 |
| rs2415142 | G | T | 0.548783 | 0.0133595 | 0.00155278 | 7.70E-18 | 0.159522527 | 74.02195503 | Body fat percentage | ukb-b-8909 |
| rs2481899 | G | A | 0.551171 | 0.00930927 | 0.00156135 | 2.50E-09 | 0.083537383 | 35.54927252 | Body fat percentage | ukb-b-8909 |
| rs2499468 | A | C | 0.650982 | 0.0100294 | 0.00161777 | 5.70E-10 | 0.089708243 | 38.43406762 | Body fat percentage | ukb-b-8909 |
| rs2508782 | G | A | 0.569716 | 0.00937803 | 0.00155792 | 1.70E-09 | 0.085012625 | 36.23538917 | Body fat percentage | ukb-b-8909 |
| rs252749 | A | G | 0.245982 | -0.0102553 | 0.00178627 | 9.40E-09 | 0.077929527 | 32.96116323 | Body fat percentage | ukb-b-8909 |
| rs256904 | T | A | 0.746327 | -0.0111835 | 0.00176815 | 2.50E-10 | 0.093034385 | 40.00527623 | Body fat percentage | ukb-b-8909 |
| rs262953 | A | G | 0.625978 | -0.0102573 | 0.00159162 | 1.20E-10 | 0.096244052 | 41.53242982 | Body fat percentage | ukb-b-8909 |
| rs2640465 | G | A | 0.902017 | 0.0158823 | 0.00259576 | 9.40E-10 | 0.087584226 | 37.43671375 | Body fat percentage | ukb-b-8909 |
| rs2660241 | C | T | 0.36485 | 0.0108857 | 0.00160421 | 1.20E-11 | 0.105598597 | 46.04582773 | Body fat percentage | ukb-b-8909 |
| rs2678204 | G | T | 0.340185 | 0.0136291 | 0.00162387 | 4.70E-17 | 0.152987829 | 70.44202611 | Body fat percentage | ukb-b-8909 |
| rs2692741 | C | G | 0.367194 | 0.00909731 | 0.0016107 | 0.000000016 | 0.075611298 | 31.9004398 | Body fat percentage | ukb-b-8909 |
| rs2702123 | C | T | 0.086 | -0.0152608 | 0.00274222 | 2.60E-08 | 0.07356957 | 30.97062813 | Body fat percentage | ukb-b-8909 |
| rs2731238 | T | G | 0.067529 | -0.0218753 | 0.00336997 | 8.50E-11 | 0.09750688 | 42.13625813 | Body fat percentage | ukb-b-8909 |
| rs2731238 | T | G | 0.067529 | -0.0218753 | 0.00336997 | 8.50E-11 | 0.09750688 | 42.13625813 | Body fat percentage | ukb-b-8909 |
| rs2785988 | A | C | 0.297382 | 0.0210728 | 0.00168127 | 4.90E-36 | 0.287147309 | 157.0976047 | Body fat percentage | ukb-b-8909 |
| rs2802774 | A | C | 0.547302 | 0.0105954 | 0.00156929 | 1.50E-11 | 0.104653732 | 45.58566549 | Body fat percentage | ukb-b-8909 |
| rs2814993 | A | G | 0.13976 | 0.0259158 | 0.00221742 | 1.50E-31 | 0.259392236 | 136.5945333 | Body fat percentage | ukb-b-8909 |
| rs2855818 | A | G | 0.241541 | 0.0153102 | 0.00181772 | 3.70E-17 | 0.153907863 | 70.94270709 | Body fat percentage | ukb-b-8909 |
| rs28651380 | G | A | 0.281193 | 0.0104262 | 0.00171657 | 1.20E-09 | 0.086419415 | 36.89173383 | Body fat percentage | ukb-b-8909 |
| rs28672845 | C | A | 0.839976 | 0.0115052 | 0.00209731 | 4.10E-08 | 0.071633771 | 30.09283404 | Body fat percentage | ukb-b-8909 |
| rs28714450 | T | C | 0.675249 | -0.0101175 | 0.00165029 | 8.70E-10 | 0.087902744 | 37.58598101 | Body fat percentage | ukb-b-8909 |
| rs28742003 | T | C | 0.205276 | -0.021134 | 0.00191016 | 1.90E-28 | 0.238893669 | 122.4119774 | Body fat percentage | ukb-b-8909 |
| rs2888778 | G | T | 0.652153 | 0.00998353 | 0.00161504 | 6.30E-10 | 0.089236532 | 38.21216888 | Body fat percentage | ukb-b-8909 |
| rs2954033 | G | A | 0.695224 | 0.0124143 | 0.00167398 | 1.20E-13 | 0.12359083 | 54.9976259 | Body fat percentage | ukb-b-8909 |
| rs2960420 | G | C | 0.350731 | -0.00912091 | 0.00162066 | 1.80E-08 | 0.075113236 | 31.67324164 | Body fat percentage | ukb-b-8909 |
| rs2966859 | G | A | 0.789007 | -0.0111483 | 0.00189749 | 4.20E-09 | 0.08131322 | 34.51900768 | Body fat percentage | ukb-b-8909 |
| rs2984618 | T | G | 0.416215 | 0.0117369 | 0.0015617 | 5.70E-14 | 0.126504927 | 56.4821979 | Body fat percentage | ukb-b-8909 |
| rs3113509 | T | C | 0.731962 | -0.0107277 | 0.00174038 | 7.10E-10 | 0.088774145 | 37.99487957 | Body fat percentage | ukb-b-8909 |
| rs319775 | C | T | 0.60881 | 0.00883995 | 0.00158047 | 2.20E-08 | 0.074259459 | 31.28434769 | Body fat percentage | ukb-b-8909 |
| rs33503 | A | G | 0.805882 | -0.0134993 | 0.00194516 | 3.90E-12 | 0.109919899 | 48.16281223 | Body fat percentage | ukb-b-8909 |
| rs33836 | T | C | 0.464327 | -0.0163804 | 0.00156772 | 1.50E-25 | 0.218706469 | 109.1721864 | Body fat percentage | ukb-b-8909 |
| rs34338229 | A | G | 0.332008 | 0.00897307 | 0.00164195 | 4.60E-08 | 0.071129966 | 29.86498187 | Body fat percentage | ukb-b-8909 |
| rs34483452 | A | C | 0.13631 | 0.0245244 | 0.00226433 | 2.50E-27 | 0.231232039 | 117.3052207 | Body fat percentage | ukb-b-8909 |
| rs34580448 | C | T | 0.041248 | -0.0308051 | 0.00388142 | 2.10E-15 | 0.139051736 | 62.98889174 | Body fat percentage | ukb-b-8909 |
| rs34656389 | G | A | 0.367474 | 0.0103136 | 0.00159872 | 1.10E-10 | 0.096422132 | 41.61747733 | Body fat percentage | ukb-b-8909 |
| rs347551 | G | C | 0.472318 | 0.00988966 | 0.00156805 | 2.80E-10 | 0.092554754 | 39.77799681 | Body fat percentage | ukb-b-8909 |
| rs35154152 | C | T | 0.106824 | -0.0177569 | 0.00249206 | 1.00E-12 | 0.115187171 | 50.77118606 | Body fat percentage | ukb-b-8909 |
| rs35523808 | A | T | 0.049077 | 0.0204619 | 0.0036589 | 2.20E-08 | 0.074237892 | 31.27453315 | Body fat percentage | ukb-b-8909 |
| rs3743861 | C | G | 0.415256 | -0.0103504 | 0.00156479 | 3.70E-11 | 0.100869583 | 43.75242646 | Body fat percentage | ukb-b-8909 |
| rs3754963 | T | A | 0.255074 | -0.0114802 | 0.00176393 | 7.60E-11 | 0.097969895 | 42.35807501 | Body fat percentage | ukb-b-8909 |
| rs3764002 | T | C | 0.261517 | -0.0186517 | 0.00175335 | 2.00E-26 | 0.224901317 | 113.1617377 | Body fat percentage | ukb-b-8909 |
| rs3765971 | T | C | 0.65847 | -0.0102079 | 0.00162025 | 3.00E-10 | 0.092374256 | 39.69252743 | Body fat percentage | ukb-b-8909 |
| rs3766823 | A | G | 0.172004 | 0.0140143 | 0.00203896 | 6.30E-12 | 0.108044794 | 47.24168813 | Body fat percentage | ukb-b-8909 |
| rs3791709 | T | A | 0.282417 | 0.0103311 | 0.00171169 | 1.60E-09 | 0.085427273 | 36.42863546 | Body fat percentage | ukb-b-8909 |
| rs3803286 | G | A | 0.666725 | -0.0115932 | 0.00163624 | 1.40E-12 | 0.11404115 | 50.20103205 | Body fat percentage | ukb-b-8909 |
| rs3817428 | G | C | 0.265007 | -0.0152755 | 0.00174905 | 2.50E-18 | 0.163585046 | 76.2757382 | Body fat percentage | ukb-b-8909 |
| rs3826408 | T | C | 0.456883 | 0.00915425 | 0.00154807 | 3.40E-09 | 0.082282722 | 34.96748106 | Body fat percentage | ukb-b-8909 |
| rs3911063 | C | T | 0.322 | -0.0121841 | 0.00165127 | 1.60E-13 | 0.122499296 | 54.44408809 | Body fat percentage | ukb-b-8909 |
| rs3923501 | T | C | 0.475818 | 0.0102177 | 0.00154181 | 3.40E-11 | 0.101213095 | 43.91820467 | Body fat percentage | ukb-b-8909 |
| rs394608 | C | T | 0.537747 | 0.0123902 | 0.00155475 | 1.60E-15 | 0.14003918 | 63.50903334 | Body fat percentage | ukb-b-8909 |
| rs396354 | C | T | 0.715662 | -0.00986214 | 0.00170937 | 0.000000008 | 0.078638567 | 33.28665627 | Body fat percentage | ukb-b-8909 |
| rs396755 | G | C | 0.571071 | -0.0102893 | 0.00155919 | 4.10E-11 | 0.100446741 | 43.54853788 | Body fat percentage | ukb-b-8909 |
| rs40071 | C | T | 0.179498 | -0.014887 | 0.00201352 | 1.40E-13 | 0.122933537 | 54.66413512 | Body fat percentage | ukb-b-8909 |
| rs41307479 | G | C | 0.220577 | 0.0124051 | 0.00185992 | 2.60E-11 | 0.102385248 | 44.48483767 | Body fat percentage | ukb-b-8909 |
| rs41310284 | A | C | 0.101004 | -0.0201214 | 0.00257596 | 5.70E-15 | 0.135284165 | 61.01521689 | Body fat percentage | ukb-b-8909 |
| rs429343 | G | A | 0.576575 | -0.0111228 | 0.00155872 | 9.60E-13 | 0.11548666 | 50.92042745 | Body fat percentage | ukb-b-8909 |
| rs429358 | C | T | 0.154044 | -0.0215029 | 0.00213952 | 9.20E-24 | 0.205717695 | 101.0093018 | Body fat percentage | ukb-b-8909 |
| rs4320040 | G | T | 0.560267 | -0.0101838 | 0.00154798 | 4.70E-11 | 0.099889586 | 43.28017744 | Body fat percentage | ukb-b-8909 |
| rs4398538 | C | T | 0.642513 | -0.010008 | 0.00161101 | 5.20E-10 | 0.090043835 | 38.59207418 | Body fat percentage | ukb-b-8909 |
| rs441792 | G | A | 0.486601 | 0.0119298 | 0.00154217 | 1.00E-14 | 0.133027795 | 59.84141095 | Body fat percentage | ukb-b-8909 |
| rs4466418 | A | G | 0.562278 | 0.00997781 | 0.00155638 | 1.40E-10 | 0.095336983 | 41.09974968 | Body fat percentage | ukb-b-8909 |
| rs4482463 | A | C | 0.923053 | -0.0211076 | 0.00289361 | 3.00E-13 | 0.12005704 | 53.21054629 | Body fat percentage | ukb-b-8909 |
| rs4496901 | T | G | 0.65522 | -0.00954008 | 0.00162185 | 0.000000004 | 0.081489591 | 34.60052296 | Body fat percentage | ukb-b-8909 |
| rs4500770 | T | A | 0.362615 | -0.00977478 | 0.00160607 | 1.20E-09 | 0.086739171 | 37.04119961 | Body fat percentage | ukb-b-8909 |
| rs4547574 | T | A | 0.227697 | -0.0105978 | 0.00183563 | 7.80E-09 | 0.078737218 | 33.33198249 | Body fat percentage | ukb-b-8909 |
| rs4690324 | A | G | 0.823217 | -0.0126754 | 0.00202574 | 3.90E-10 | 0.091231458 | 39.15217915 | Body fat percentage | ukb-b-8909 |
| rs4709745 | C | T | 0.307185 | 0.00934269 | 0.00167115 | 2.30E-08 | 0.074193938 | 31.25453277 | Body fat percentage | ukb-b-8909 |
| rs4718964 | T | G | 0.413126 | 0.0111105 | 0.00157055 | 1.50E-12 | 0.113727697 | 50.04534334 | Body fat percentage | ukb-b-8909 |
| rs4722398 | T | C | 0.13612 | 0.0143436 | 0.00224286 | 1.60E-10 | 0.094915377 | 40.89893506 | Body fat percentage | ukb-b-8909 |
| rs4759318 | T | C | 0.362201 | 0.0104428 | 0.00160707 | 8.10E-11 | 0.097691093 | 42.22448225 | Body fat percentage | ukb-b-8909 |
| rs4762951 | G | A | 0.780444 | -0.0113662 | 0.00186311 | 1.10E-09 | 0.087117219 | 37.21804816 | Body fat percentage | ukb-b-8909 |
| rs4776337 | A | G | 0.468075 | 0.00998636 | 0.00154808 | 1.10E-10 | 0.09641248 | 41.61286678 | Body fat percentage | ukb-b-8909 |
| rs479018 | A | G | 0.332499 | -0.0144775 | 0.00166392 | 3.30E-18 | 0.162559269 | 75.704599 | Body fat percentage | ukb-b-8909 |
| rs4790841 | T | C | 0.154504 | -0.0208407 | 0.00214396 | 2.50E-22 | 0.195031777 | 94.49117449 | Body fat percentage | ukb-b-8909 |
| rs482787 | C | T | 0.325766 | 0.0092676 | 0.00164764 | 1.90E-08 | 0.075036073 | 31.63806457 | Body fat percentage | ukb-b-8909 |
| rs4837119 | A | T | 0.517271 | -0.0101722 | 0.00154703 | 4.90E-11 | 0.099795071 | 43.23468605 | Body fat percentage | ukb-b-8909 |
| rs4876611 | G | A | 0.720188 | 0.0173998 | 0.00171871 | 4.30E-24 | 0.208106436 | 102.4904276 | Body fat percentage | ukb-b-8909 |
| rs4894808 | C | G | 0.40013 | -0.0100803 | 0.00160438 | 3.30E-10 | 0.091916526 | 39.47593596 | Body fat percentage | ukb-b-8909 |
| rs4908676 | G | A | 0.457718 | 0.00966112 | 0.00154844 | 4.40E-10 | 0.090757269 | 38.92836733 | Body fat percentage | ukb-b-8909 |
| rs4959613 | A | C | 0.594024 | 0.00955622 | 0.00161789 | 3.50E-09 | 0.082110742 | 34.88785731 | Body fat percentage | ukb-b-8909 |
| rs543874 | G | A | 0.20527 | 0.0301643 | 0.00190436 | 1.70E-56 | 0.391474005 | 250.8929172 | Body fat percentage | ukb-b-8909 |
| rs55707359 | G | T | 0.015449 | 0.0375397 | 0.00633223 | 3.10E-09 | 0.082666732 | 35.14537918 | Body fat percentage | ukb-b-8909 |
| rs55810445 | T | C | 0.151196 | -0.0140163 | 0.00215528 | 7.90E-11 | 0.097832254 | 42.29211175 | Body fat percentage | ukb-b-8909 |
| rs55924785 | T | C | 0.164678 | -0.0123455 | 0.00207928 | 2.90E-09 | 0.082898069 | 35.25262126 | Body fat percentage | ukb-b-8909 |
| rs56218501 | T | C | 0.211814 | -0.0128916 | 0.00188796 | 8.60E-12 | 0.106787024 | 46.62598999 | Body fat percentage | ukb-b-8909 |
| rs56328878 | A | C | 0.267194 | -0.00975461 | 0.00173683 | 0.00000002 | 0.07482783 | 31.54315986 | Body fat percentage | ukb-b-8909 |
| rs56369689 | G | A | 0.348594 | -0.00952112 | 0.00165613 | 9.00E-09 | 0.078125768 | 33.05119977 | Body fat percentage | ukb-b-8909 |
| rs56399737 | T | C | 0.44917 | -0.0118589 | 0.00155668 | 2.60E-14 | 0.129532326 | 58.03501786 | Body fat percentage | ukb-b-8909 |
| rs57636386 | C | T | 0.083836 | -0.0252015 | 0.00279375 | 1.90E-19 | 0.172628879 | 81.37250783 | Body fat percentage | ukb-b-8909 |
| rs57800857 | C | A | 0.3649 | -0.0133302 | 0.00160952 | 1.20E-16 | 0.149572943 | 68.59312286 | Body fat percentage | ukb-b-8909 |
| rs58120873 | A | G | 0.08686 | -0.0163391 | 0.00274979 | 2.80E-09 | 0.083014695 | 35.30670631 | Body fat percentage | ukb-b-8909 |
| rs58862095 | T | C | 0.419262 | -0.0131217 | 0.00156604 | 5.30E-17 | 0.152553504 | 70.20604487 | Body fat percentage | ukb-b-8909 |
| rs59227842 | G | A | 0.311468 | 0.0170384 | 0.00167907 | 3.40E-24 | 0.208880347 | 102.9722056 | Body fat percentage | ukb-b-8909 |
| rs59499656 | T | A | 0.343189 | -0.0137829 | 0.00163035 | 2.80E-17 | 0.154873354 | 71.46929775 | Body fat percentage | ukb-b-8909 |
| rs6021948 | A | T | 0.32177 | -0.0130773 | 0.0016559 | 2.80E-15 | 0.137871734 | 62.36888231 | Body fat percentage | ukb-b-8909 |
| rs6064113 | C | T | 0.765465 | -0.0105166 | 0.00185641 | 0.000000015 | 0.076031809 | 32.09245295 | Body fat percentage | ukb-b-8909 |
| rs6103254 | C | T | 0.126684 | -0.0147849 | 0.00232906 | 2.20E-10 | 0.093649801 | 40.29725183 | Body fat percentage | ukb-b-8909 |
| rs61754230 | T | C | 0.019711 | 0.0312346 | 0.00554241 | 0.000000017 | 0.075302545 | 31.75956885 | Body fat percentage | ukb-b-8909 |
| rs61903695 | G | A | 0.254879 | 0.0118139 | 0.00177153 | 2.60E-11 | 0.102359338 | 44.47229667 | Body fat percentage | ukb-b-8909 |
| rs61910767 | T | C | 0.164197 | -0.0142941 | 0.00208312 | 6.80E-12 | 0.107725591 | 47.08526889 | Body fat percentage | ukb-b-8909 |
| rs61969510 | C | T | 0.279026 | 0.0127422 | 0.00173502 | 2.10E-13 | 0.121495268 | 53.93614021 | Body fat percentage | ukb-b-8909 |
| rs61975147 | C | T | 0.167185 | -0.0142041 | 0.00207301 | 7.30E-12 | 0.107446947 | 46.94881642 | Body fat percentage | ukb-b-8909 |
| rs61986205 | G | A | 0.082024 | 0.0189406 | 0.00283307 | 2.30E-11 | 0.102822173 | 44.69643184 | Body fat percentage | ukb-b-8909 |
| rs62107261 | C | T | 0.048327 | -0.0479135 | 0.00359643 | 1.70E-40 | 0.312762567 | 177.4894606 | Body fat percentage | ukb-b-8909 |
| rs62190394 | T | C | 0.317033 | 0.0151644 | 0.00165383 | 4.80E-20 | 0.17734598 | 84.07535927 | Body fat percentage | ukb-b-8909 |
| rs62218301 | G | A | 0.166049 | -0.0127956 | 0.00208683 | 8.70E-10 | 0.087925136 | 37.59647844 | Body fat percentage | ukb-b-8909 |
| rs62413414 | T | C | 0.151703 | 0.0126824 | 0.00214424 | 3.30E-09 | 0.08231607 | 34.982924 | Body fat percentage | ukb-b-8909 |
| rs62443626 | A | G | 0.463946 | -0.0106141 | 0.00155316 | 8.30E-12 | 0.106942157 | 46.70183612 | Body fat percentage | ukb-b-8909 |
| rs62621197 | T | C | 0.037191 | -0.0249637 | 0.00423712 | 3.80E-09 | 0.081730116 | 34.71173982 | Body fat percentage | ukb-b-8909 |
| rs6480350 | C | T | 0.575106 | -0.00953851 | 0.00156462 | 1.10E-09 | 0.087005531 | 37.16578617 | Body fat percentage | ukb-b-8909 |
| rs6491427 | G | A | 0.289013 | -0.013993 | 0.00170162 | 2.00E-16 | 0.147770708 | 67.62332258 | Body fat percentage | ukb-b-8909 |
| rs6500594 | G | T | 0.246636 | 0.0110602 | 0.00179199 | 6.70E-10 | 0.088984797 | 38.09384383 | Body fat percentage | ukb-b-8909 |
| rs6561937 | A | T | 0.753684 | -0.0117153 | 0.00179629 | 6.90E-11 | 0.098340391 | 42.5357333 | Body fat percentage | ukb-b-8909 |
| rs6567160 | C | T | 0.232676 | 0.0254579 | 0.00182642 | 3.70E-44 | 0.332519858 | 194.2870455 | Body fat percentage | ukb-b-8909 |
| rs6575340 | A | G | 0.636023 | 0.0133993 | 0.00160775 | 7.80E-17 | 0.151175225 | 69.45878545 | Body fat percentage | ukb-b-8909 |
| rs657685 | C | G | 0.456088 | -0.00989059 | 0.00154908 | 1.70E-10 | 0.094635792 | 40.76586901 | Body fat percentage | ukb-b-8909 |
| rs6602997 | T | C | 0.711856 | 0.0226029 | 0.00170655 | 4.80E-40 | 0.310252762 | 175.4245184 | Body fat percentage | ukb-b-8909 |
| rs6688826 | C | T | 0.298206 | 0.0105922 | 0.0016789 | 2.80E-10 | 0.092608875 | 39.80363056 | Body fat percentage | ukb-b-8909 |
| rs6693294 | G | A | 0.688566 | -0.0136932 | 0.0016614 | 1.70E-16 | 0.148341413 | 67.9299806 | Body fat percentage | ukb-b-8909 |
| rs6699744 | T | A | 0.616045 | 0.0142768 | 0.00158851 | 2.50E-19 | 0.171580376 | 80.77590733 | Body fat percentage | ukb-b-8909 |
| rs6699744 | T | A | 0.616045 | 0.0142768 | 0.00158851 | 2.50E-19 | 0.171580376 | 80.77590733 | Body fat percentage | ukb-b-8909 |
| rs6744646 | G | A | 0.828299 | 0.028139 | 0.00203811 | 2.30E-43 | 0.328300984 | 190.6171971 | Body fat percentage | ukb-b-8909 |
| rs6749911 | A | G | 0.126972 | 0.0126967 | 0.00230925 | 3.80E-08 | 0.071937052 | 30.23011586 | Body fat percentage | ukb-b-8909 |
| rs6750646 | T | C | 0.201154 | -0.0117765 | 0.00192871 | 0.000000001 | 0.087253742 | 37.28194903 | Body fat percentage | ukb-b-8909 |
| rs6752378 | A | C | 0.486256 | 0.0224027 | 0.00153822 | 4.80E-48 | 0.352279228 | 212.1113064 | Body fat percentage | ukb-b-8909 |
| rs6754292 | T | C | 0.644629 | -0.00932639 | 0.00161898 | 8.40E-09 | 0.078417633 | 33.18517996 | Body fat percentage | ukb-b-8909 |
| rs67609008 | C | T | 0.283593 | 0.0113458 | 0.00171702 | 3.90E-11 | 0.100685416 | 43.66359998 | Body fat percentage | ukb-b-8909 |
| rs6782581 | G | C | 0.439266 | -0.0101996 | 0.00155143 | 4.90E-11 | 0.0997681 | 43.22170654 | Body fat percentage | ukb-b-8909 |
| rs6840236 | C | T | 0.464881 | 0.0121284 | 0.00154596 | 4.30E-15 | 0.136303517 | 61.54751429 | Body fat percentage | ukb-b-8909 |
| rs6843910 | A | T | 0.45449 | -0.0104169 | 0.00155088 | 1.90E-11 | 0.103685155 | 45.11496235 | Body fat percentage | ukb-b-8909 |
| rs6847975 | A | G | 0.35617 | 0.0113515 | 0.00161543 | 2.10E-12 | 0.112380842 | 49.37762752 | Body fat percentage | ukb-b-8909 |
| rs685149 | G | A | 0.645411 | -0.00954107 | 0.00161396 | 3.40E-09 | 0.08223827 | 34.94689782 | Body fat percentage | ukb-b-8909 |
| rs6875585 | C | A | 0.670422 | 0.011204 | 0.00164464 | 9.60E-12 | 0.106343405 | 46.40924496 | Body fat percentage | ukb-b-8909 |
| rs6927268 | G | T | 0.206332 | -0.0138777 | 0.00190462 | 3.20E-13 | 0.119819 | 53.09068257 | Body fat percentage | ukb-b-8909 |
| rs6948959 | A | G | 0.744356 | -0.0105935 | 0.00177956 | 2.60E-09 | 0.083294953 | 35.43673257 | Body fat percentage | ukb-b-8909 |
| rs6973656 | G | A | 0.397021 | 0.00928324 | 0.00157441 | 3.70E-09 | 0.081848932 | 34.76670061 | Body fat percentage | ukb-b-8909 |
| rs6977416 | A | G | 0.334244 | -0.0118159 | 0.00164828 | 7.60E-13 | 0.116425995 | 51.38917382 | Body fat percentage | ukb-b-8909 |
| rs7020 | A | G | 0.437471 | 0.0130676 | 0.00155587 | 4.50E-17 | 0.153170886 | 70.54155853 | Body fat percentage | ukb-b-8909 |
| rs7027304 | T | C | 0.652657 | 0.011578 | 0.0016271 | 1.10E-12 | 0.114910844 | 50.63357597 | Body fat percentage | ukb-b-8909 |
| rs704061 | C | T | 0.455038 | 0.0136927 | 0.00154796 | 9.10E-19 | 0.167103372 | 78.2453825 | Body fat percentage | ukb-b-8909 |
| rs7046679 | G | C | 0.678548 | -0.00914252 | 0.00165304 | 0.000000032 | 0.072728933 | 30.58898826 | Body fat percentage | ukb-b-8909 |
| rs7124681 | A | C | 0.408419 | 0.0222563 | 0.0015648 | 6.60E-46 | 0.341545942 | 202.2964485 | Body fat percentage | ukb-b-8909 |
| rs7132908 | A | G | 0.38447 | 0.0197321 | 0.00158551 | 1.50E-35 | 0.284252305 | 154.8847448 | Body fat percentage | ukb-b-8909 |
| rs7133378 | A | G | 0.319439 | 0.0185779 | 0.00165616 | 3.30E-29 | 0.243938851 | 125.831293 | Body fat percentage | ukb-b-8909 |
| rs71658797 | A | T | 0.120747 | 0.0236276 | 0.00236585 | 1.70E-23 | 0.203657383 | 99.73895369 | Body fat percentage | ukb-b-8909 |
| rs719802 | C | T | 0.6144 | -0.0115343 | 0.0015826 | 3.10E-13 | 0.119872885 | 53.11781024 | Body fat percentage | ukb-b-8909 |
| rs7206608 | G | C | 0.321575 | 0.0101676 | 0.00165236 | 7.60E-10 | 0.088495608 | 37.86409305 | Body fat percentage | ukb-b-8909 |
| rs7216121 | G | A | 0.626339 | -0.0109061 | 0.00161091 | 1.30E-11 | 0.105165764 | 45.83491138 | Body fat percentage | ukb-b-8909 |
| rs7218014 | C | T | 0.197302 | 0.0211077 | 0.00194282 | 1.70E-27 | 0.232338774 | 118.036601 | Body fat percentage | ukb-b-8909 |
| rs72634814 | A | G | 0.345475 | -0.0128866 | 0.00169042 | 2.50E-14 | 0.129687486 | 58.11489423 | Body fat percentage | ukb-b-8909 |
| rs72681698 | C | T | 0.010721 | -0.052548 | 0.00748992 | 2.30E-12 | 0.112066053 | 49.22186041 | Body fat percentage | ukb-b-8909 |
| rs72697297 | C | T | 0.179917 | -0.0153167 | 0.00203008 | 4.50E-14 | 0.127370637 | 56.92513995 | Body fat percentage | ukb-b-8909 |
| rs72703757 | C | G | 0.198824 | 0.0115499 | 0.00194851 | 3.10E-09 | 0.082646294 | 35.1359071 | Body fat percentage | ukb-b-8909 |
| rs72755233 | A | G | 0.11145 | -0.0163172 | 0.0024497 | 2.70E-11 | 0.102142879 | 44.36755221 | Body fat percentage | ukb-b-8909 |
| rs72767957 | G | A | 0.200131 | -0.012668 | 0.00193068 | 5.30E-11 | 0.099415763 | 43.05221634 | Body fat percentage | ukb-b-8909 |
| rs72803260 | G | T | 0.084979 | -0.0156141 | 0.0027727 | 1.80E-08 | 0.075198963 | 31.71232971 | Body fat percentage | ukb-b-8909 |
| rs72892910 | T | G | 0.172194 | 0.0221404 | 0.00204337 | 2.30E-27 | 0.231379245 | 117.4023796 | Body fat percentage | ukb-b-8909 |
| rs72917533 | C | T | 0.185499 | -0.0138669 | 0.0019837 | 2.70E-12 | 0.111346058 | 48.86599888 | Body fat percentage | ukb-b-8909 |
| rs72976986 | A | G | 0.190192 | -0.0171142 | 0.00198547 | 6.70E-18 | 0.160025144 | 74.29961223 | Body fat percentage | ukb-b-8909 |
| rs72995085 | C | T | 0.177033 | -0.0117629 | 0.00202255 | 6.00E-09 | 0.07980761 | 33.82441363 | Body fat percentage | ukb-b-8909 |
| rs7321331 | A | G | 0.742296 | 0.0107397 | 0.00177343 | 1.40E-09 | 0.085952833 | 36.67382416 | Body fat percentage | ukb-b-8909 |
| rs73213501 | C | A | 0.171839 | -0.0152527 | 0.00204286 | 8.20E-14 | 0.125062894 | 55.74632559 | Body fat percentage | ukb-b-8909 |
| rs7357754 | G | A | 0.500129 | 0.0116159 | 0.00154667 | 5.90E-14 | 0.126352042 | 56.40406515 | Body fat percentage | ukb-b-8909 |
| rs74288880 | T | C | 0.114819 | -0.0135582 | 0.00241631 | 0.00000002 | 0.074699383 | 31.48464273 | Body fat percentage | ukb-b-8909 |
| rs7442885 | G | C | 0.214136 | -0.0128176 | 0.00188108 | 9.50E-12 | 0.10638595 | 46.4300228 | Body fat percentage | ukb-b-8909 |
| rs74576293 | C | T | 0.089728 | -0.0204973 | 0.002693 | 2.70E-14 | 0.129332672 | 57.93227829 | Body fat percentage | ukb-b-8909 |
| rs74618095 | C | T | 0.157976 | 0.0132675 | 0.00213178 | 4.90E-10 | 0.090345266 | 38.73409611 | Body fat percentage | ukb-b-8909 |
| rs7463186 | G | A | 0.516044 | 0.0088055 | 0.00154188 | 1.10E-08 | 0.07717254 | 32.6142122 | Body fat percentage | ukb-b-8909 |
| rs7498665 | G | A | 0.399562 | 0.0232064 | 0.00157608 | 4.50E-49 | 0.357283983 | 216.799877 | Body fat percentage | ukb-b-8909 |
| rs75135487 | G | C | 0.136931 | -0.0144347 | 0.00223876 | 1.10E-10 | 0.096326822 | 41.57195486 | Body fat percentage | ukb-b-8909 |
| rs7535438 | A | C | 0.290883 | -0.00950812 | 0.00169742 | 0.000000021 | 0.074462893 | 31.37694659 | Body fat percentage | ukb-b-8909 |
| rs75412871 | T | C | 0.051916 | -0.0216364 | 0.00347856 | 5.00E-10 | 0.090246456 | 38.68753027 | Body fat percentage | ukb-b-8909 |
| rs7575523 | G | T | 0.603242 | -0.0153971 | 0.00157743 | 1.70E-22 | 0.196331508 | 95.27471726 | Body fat percentage | ukb-b-8909 |
| rs7598246 | C | T | 0.583694 | 0.0085494 | 0.00156226 | 4.40E-08 | 0.071313107 | 29.9477809 | Body fat percentage | ukb-b-8909 |
| rs76115890 | C | T | 0.117876 | -0.0139023 | 0.00241521 | 8.60E-09 | 0.078304443 | 33.13321019 | Body fat percentage | ukb-b-8909 |
| rs7630228 | C | T | 0.434322 | -0.0108516 | 0.0015618 | 3.70E-12 | 0.110150996 | 48.27660451 | Body fat percentage | ukb-b-8909 |
| rs76345589 | G | C | 0.067492 | -0.0198865 | 0.00308056 | 1.10E-10 | 0.096538874 | 41.6732494 | Body fat percentage | ukb-b-8909 |
| rs7680610 | G | A | 0.652245 | 0.0089709 | 0.00163023 | 3.70E-08 | 0.072050039 | 30.28128281 | Body fat percentage | ukb-b-8909 |
| rs76856798 | T | C | 0.018465 | 0.0322372 | 0.00573378 | 1.90E-08 | 0.074975793 | 31.61058814 | Body fat percentage | ukb-b-8909 |
| rs7704382 | G | C | 0.433417 | 0.0101719 | 0.00155683 | 6.40E-11 | 0.098660964 | 42.68957 | Body fat percentage | ukb-b-8909 |
| rs7707394 | A | G | 0.357305 | -0.0126609 | 0.00160546 | 3.10E-15 | 0.137533312 | 62.19137775 | Body fat percentage | ukb-b-8909 |
| rs7762794 | G | A | 0.285523 | 0.00930584 | 0.00170461 | 4.80E-08 | 0.070992992 | 29.80307658 | Body fat percentage | ukb-b-8909 |
| rs7773916 | T | C | 0.213136 | -0.0109353 | 0.00188165 | 6.20E-09 | 0.079698346 | 33.77409452 | Body fat percentage | ukb-b-8909 |
| rs7789056 | A | G | 0.563037 | -0.0127186 | 0.00155559 | 2.90E-16 | 0.146324355 | 66.84798682 | Body fat percentage | ukb-b-8909 |
| rs7796825 | A | G | 0.825769 | 0.0113141 | 0.00203645 | 2.80E-08 | 0.073341162 | 30.86686502 | Body fat percentage | ukb-b-8909 |
| rs78296744 | A | G | 0.272344 | -0.0132887 | 0.00173423 | 1.80E-14 | 0.130852096 | 58.71534334 | Body fat percentage | ukb-b-8909 |
| rs7843109 | T | C | 0.688667 | 0.0103657 | 0.00166877 | 5.20E-10 | 0.090026137 | 38.58373833 | Body fat percentage | ukb-b-8909 |
| rs78744936 | A | G | 0.265987 | 0.0113474 | 0.00175799 | 1.10E-10 | 0.096519307 | 41.66390072 | Body fat percentage | ukb-b-8909 |
| rs7893571 | T | G | 0.665915 | 0.0100326 | 0.00163856 | 9.20E-10 | 0.087695537 | 37.48886542 | Body fat percentage | ukb-b-8909 |
| rs7925725 | C | A | 0.411245 | 0.0111592 | 0.00156858 | 1.10E-12 | 0.114867333 | 50.61191569 | Body fat percentage | ukb-b-8909 |
| rs7942368 | T | C | 0.217538 | -0.0105396 | 0.00189331 | 2.60E-08 | 0.073609566 | 30.98880293 | Body fat percentage | ukb-b-8909 |
| rs79518326 | A | C | 0.02864 | -0.0322259 | 0.00466633 | 5.00E-12 | 0.108965525 | 47.69350205 | Body fat percentage | ukb-b-8909 |
| rs7960609 | G | A | 0.326919 | 0.00918558 | 0.00164856 | 0.000000025 | 0.073735097 | 31.04585692 | Body fat percentage | ukb-b-8909 |
| rs7966251 | A | G | 0.255023 | -0.00981406 | 0.00177103 | 3.00E-08 | 0.072990316 | 30.70757928 | Body fat percentage | ukb-b-8909 |
| rs7972728 | A | C | 0.73765 | 0.0167619 | 0.00175434 | 1.20E-21 | 0.189676238 | 91.28910713 | Body fat percentage | ukb-b-8909 |
| rs7975187 | G | A | 0.213862 | 0.0115294 | 0.00188095 | 8.80E-10 | 0.087871923 | 37.57153263 | Body fat percentage | ukb-b-8909 |
| rs798549 | A | C | 0.730794 | -0.00992339 | 0.00174424 | 1.30E-08 | 0.076633253 | 32.36738669 | Body fat percentage | ukb-b-8909 |
| rs7987928 | A | G | 0.799871 | -0.0138119 | 0.00193112 | 8.50E-13 | 0.115957033 | 51.15502806 | Body fat percentage | ukb-b-8909 |
| rs8096564 | T | G | 0.298122 | 0.0107485 | 0.00169686 | 2.40E-10 | 0.093284625 | 40.12395154 | Body fat percentage | ukb-b-8909 |
| rs811054 | T | C | 0.537276 | 0.0095458 | 0.00155694 | 8.70E-10 | 0.087912902 | 37.59074293 | Body fat percentage | ukb-b-8909 |
| rs8112818 | G | A | 0.400376 | -0.0143589 | 0.00158003 | 1.00E-19 | 0.17475512 | 82.58699723 | Body fat percentage | ukb-b-8909 |
| rs812949 | C | T | 0.729218 | 0.0133557 | 0.00173358 | 1.30E-14 | 0.13208622 | 59.35339076 | Body fat percentage | ukb-b-8909 |
| rs815163 | C | T | 0.563216 | -0.00909072 | 0.00154941 | 4.40E-09 | 0.081108004 | 34.42419979 | Body fat percentage | ukb-b-8909 |
| rs843901 | G | T | 0.936414 | 0.0194862 | 0.00316613 | 7.50E-10 | 0.088527052 | 37.87885352 | Body fat percentage | ukb-b-8909 |
| rs853961 | T | G | 0.505909 | 0.0122048 | 0.00154046 | 2.30E-15 | 0.138637807 | 62.77120742 | Body fat percentage | ukb-b-8909 |
| rs879620 | T | C | 0.613285 | 0.014719 | 0.00158851 | 1.90E-20 | 0.180426391 | 85.85719669 | Body fat percentage | ukb-b-8909 |
| rs881929 | T | G | 0.375396 | -0.0147342 | 0.00159151 | 2.10E-20 | 0.180173775 | 85.71056889 | Body fat percentage | ukb-b-8909 |
| rs9289630 | C | G | 0.38912 | 0.0123827 | 0.00158474 | 5.60E-15 | 0.135358661 | 61.05407594 | Body fat percentage | ukb-b-8909 |
| rs9304665 | A | T | 0.763642 | 0.0118765 | 0.00182093 | 6.90E-11 | 0.098347848 | 42.53931028 | Body fat percentage | ukb-b-8909 |
| rs9321191 | C | T | 0.1994 | -0.0108699 | 0.0019277 | 0.000000017 | 0.075382367 | 31.79597909 | Body fat percentage | ukb-b-8909 |
| rs9358912 | T | G | 0.27332 | -0.0232653 | 0.00172866 | 2.70E-41 | 0.317147078 | 181.133237 | Body fat percentage | ukb-b-8909 |
| rs9372414 | T | C | 0.347629 | -0.00933072 | 0.00162144 | 8.70E-09 | 0.078265404 | 33.11528896 | Body fat percentage | ukb-b-8909 |
| rs9389857 | T | C | 0.052433 | 0.0198061 | 0.00346457 | 1.10E-08 | 0.077318999 | 32.68129476 | Body fat percentage | ukb-b-8909 |
| rs9568867 | A | G | 0.129188 | 0.0193443 | 0.00231898 | 7.30E-17 | 0.151407271 | 69.58442317 | Body fat percentage | ukb-b-8909 |
| rs957919 | T | C | 0.278023 | 0.0138914 | 0.00171911 | 6.40E-16 | 0.143413863 | 65.29571782 | Body fat percentage | ukb-b-8909 |
| rs9645335 | T | C | 0.367818 | -0.00955748 | 0.00159404 | 0.000000002 | 0.084397704 | 35.94912842 | Body fat percentage | ukb-b-8909 |
| rs972283 | G | A | 0.511944 | -0.0143133 | 0.00154037 | 1.50E-20 | 0.181263031 | 86.34345929 | Body fat percentage | ukb-b-8909 |
| rs9788550 | C | G | 0.24751 | -0.0158356 | 0.00179488 | 1.10E-18 | 0.166380194 | 77.83917237 | Body fat percentage | ukb-b-8909 |
| rs9814758 | G | T | 0.355786 | -0.0136044 | 0.00161607 | 3.80E-17 | 0.153767197 | 70.8660863 | Body fat percentage | ukb-b-8909 |
| rs9843653 | C | T | 0.51154 | 0.0167794 | 0.00154077 | 1.30E-27 | 0.233186074 | 118.5979622 | Body fat percentage | ukb-b-8909 |
| rs9865173 | A | T | 0.703199 | -0.0120222 | 0.00168776 | 1.10E-12 | 0.115123638 | 50.73953904 | Body fat percentage | ukb-b-8909 |
| rs9892466 | A | T | 0.333275 | 0.0100634 | 0.00163774 | 8.00E-10 | 0.088267789 | 37.75718067 | Body fat percentage | ukb-b-8909 |
| rs9955276 | T | C | 0.144431 | 0.0127569 | 0.00220651 | 7.40E-09 | 0.078940786 | 33.42554539 | Body fat percentage | ukb-b-8909 |
| rs998584 | A | C | 0.482871 | -0.00938942 | 0.00154401 | 1.20E-09 | 0.086610168 | 36.98088622 | Body fat percentage | ukb-b-8909 |

Supplementary Table 9: Information on all instrumental variables (IVs) ultimately used for Trunk fat percentage in our study

| SNPs | effect_allele.exposure | other_allele.exposure | eaf.exposure | beta.exposure | se.exposure | pval.exposure | R^2^ | F | trait | id.exposure |
| --- | --- | --- | --- | --- | --- | --- | --- | --- | --- | --- |
| rs10050620 | T | C | 0.325414 | -0.0123737 | 0.00197604 | 3.80E-10 | 0.092432733 | 39.21098044 | Trunk fat percentage | ukb-b-16407 |
| rs10099330 | G | A | 0.452943 | 0.0107002 | 0.00186159 | 9.00E-09 | 0.079031423 | 33.03814963 | Trunk fat percentage | ukb-b-16407 |
| rs10100245 | A | G | 0.564468 | 0.0183786 | 0.00186847 | 7.90E-23 | 0.200830905 | 96.75036072 | Trunk fat percentage | ukb-b-16407 |
| rs10116857 | A | C | 0.061828 | -0.0265435 | 0.00384844 | 5.30E-12 | 0.109973747 | 47.5715099 | Trunk fat percentage | ukb-b-16407 |
| rs10146997 | G | A | 0.221858 | 0.0211581 | 0.00222918 | 2.30E-21 | 0.18962244 | 90.08719297 | Trunk fat percentage | ukb-b-16407 |
| rs10172196 | A | G | 0.305528 | 0.0173947 | 0.00200943 | 4.90E-18 | 0.162926267 | 74.93558851 | Trunk fat percentage | ukb-b-16407 |
| rs1020048 | C | A | 0.809087 | -0.0136658 | 0.00238119 | 9.50E-09 | 0.078808191 | 32.93684689 | Trunk fat percentage | ukb-b-16407 |
| rs10210468 | C | T | 0.46439 | -0.0112883 | 0.00187561 | 1.80E-09 | 0.085992583 | 36.22196499 | Trunk fat percentage | ukb-b-16407 |
| rs10278040 | A | G | 0.059768 | -0.0215138 | 0.00391021 | 3.80E-08 | 0.072895707 | 30.27151034 | Trunk fat percentage | ukb-b-16407 |
| rs1042317 | C | T | 0.679299 | -0.0144208 | 0.00199314 | 4.60E-13 | 0.119694885 | 52.34836205 | Trunk fat percentage | ukb-b-16407 |
| rs10423928 | A | T | 0.194428 | -0.0251417 | 0.00233759 | 5.60E-27 | 0.231043454 | 115.6784867 | Trunk fat percentage | ukb-b-16407 |
| rs10510025 | T | C | 0.247083 | 0.0141852 | 0.00215172 | 4.30E-11 | 0.101435073 | 43.46096985 | Trunk fat percentage | ukb-b-16407 |
| rs10518707 | G | A | 0.463408 | 0.0138834 | 0.00185698 | 7.60E-14 | 0.126777302 | 55.89554825 | Trunk fat percentage | ukb-b-16407 |
| rs1056441 | C | T | 0.675264 | 0.0141199 | 0.00197998 | 9.90E-13 | 0.116680605 | 50.85593403 | Trunk fat percentage | ukb-b-16407 |
| rs10746056 | C | G | 0.471721 | 0.011026 | 0.00185204 | 2.60E-09 | 0.084300029 | 35.44338986 | Trunk fat percentage | ukb-b-16407 |
| rs10756798 | T | C | 0.642449 | -0.0163653 | 0.00193124 | 2.40E-17 | 0.157195943 | 71.80843239 | Trunk fat percentage | ukb-b-16407 |
| rs10788497 | C | G | 0.499602 | 0.0117039 | 0.00185392 | 2.70E-10 | 0.093807774 | 39.85467075 | Trunk fat percentage | ukb-b-16407 |
| rs10795422 | G | A | 0.690165 | 0.0117303 | 0.00200215 | 4.70E-09 | 0.081860252 | 34.32614365 | Trunk fat percentage | ukb-b-16407 |
| rs10799319 | G | A | 0.705006 | -0.0121003 | 0.00202384 | 2.20E-09 | 0.084960854 | 35.74702674 | Trunk fat percentage | ukb-b-16407 |
| rs10799778 | G | T | 0.833684 | -0.0155064 | 0.00248058 | 4.10E-10 | 0.092144899 | 39.07648488 | Trunk fat percentage | ukb-b-16407 |
| rs10852238 | A | T | 0.862078 | 0.0174571 | 0.00277761 | 3.30E-10 | 0.093051536 | 39.50041562 | Trunk fat percentage | ukb-b-16407 |
| rs10854853 | T | G | 0.456801 | 0.0119385 | 0.00186147 | 1.40E-10 | 0.096525611 | 41.13272147 | Trunk fat percentage | ukb-b-16407 |
| rs1086103 | A | C | 0.045766 | -0.0288841 | 0.00442801 | 6.90E-11 | 0.099520716 | 42.55009106 | Trunk fat percentage | ukb-b-16407 |
| rs10938397 | G | A | 0.4343 | 0.023014 | 0.00186793 | 7.00E-35 | 0.2827828 | 151.7969422 | Trunk fat percentage | ukb-b-16407 |
| rs10959841 | C | T | 0.387377 | -0.010495 | 0.00190448 | 3.60E-08 | 0.073110427 | 30.36771069 | Trunk fat percentage | ukb-b-16407 |
| rs10982576 | C | T | 0.239528 | -0.0129878 | 0.00217715 | 2.40E-09 | 0.084613249 | 35.58725423 | Trunk fat percentage | ukb-b-16407 |
| rs10999460 | T | C | 0.265497 | 0.0217604 | 0.00210548 | 4.90E-25 | 0.217185035 | 106.8148187 | Trunk fat percentage | ukb-b-16407 |
| rs1100237 | C | T | 0.600144 | -0.0113842 | 0.0018871 | 1.60E-09 | 0.086363079 | 36.3927777 | Trunk fat percentage | ukb-b-16407 |
| rs1101152 | A | G | 0.75909 | -0.0119527 | 0.00216631 | 3.40E-08 | 0.073278933 | 30.44323707 | Trunk fat percentage | ukb-b-16407 |
| rs11022718 | T | C | 0.202981 | -0.0134016 | 0.00231687 | 7.30E-09 | 0.079957148 | 33.45876991 | Trunk fat percentage | ukb-b-16407 |
| rs11030016 | T | C | 0.739773 | 0.015054 | 0.00211382 | 1.10E-12 | 0.116402325 | 50.71866551 | Trunk fat percentage | ukb-b-16407 |
| rs11030108 | G | A | 0.679964 | -0.020427 | 0.00198183 | 6.50E-25 | 0.216264475 | 106.2371426 | Trunk fat percentage | ukb-b-16407 |
| rs11045172 | C | A | 0.197851 | 0.0132185 | 0.00233517 | 0.000000015 | 0.076832852 | 32.04257028 | Trunk fat percentage | ukb-b-16407 |
| rs1106761 | A | G | 0.38401 | 0.0122542 | 0.00192716 | 2.00E-10 | 0.095039328 | 40.43285259 | Trunk fat percentage | ukb-b-16407 |
| rs11079849 | T | C | 0.328552 | -0.0135906 | 0.00197625 | 6.10E-12 | 0.109399581 | 47.29263313 | Trunk fat percentage | ukb-b-16407 |
| rs11080884 | A | G | 0.644836 | -0.0116424 | 0.00193858 | 1.90E-09 | 0.085657565 | 36.0676276 | Trunk fat percentage | ukb-b-16407 |
| rs11105842 | A | G | 0.367523 | -0.0119628 | 0.0019284 | 5.50E-10 | 0.090873076 | 38.4832231 | Trunk fat percentage | ukb-b-16407 |
| rs11119208 | G | A | 0.614905 | -0.0105894 | 0.00189611 | 2.30E-08 | 0.074941777 | 31.19001974 | Trunk fat percentage | ukb-b-16407 |
| rs11122450 | G | T | 0.611709 | -0.0117894 | 0.00189597 | 5.00E-10 | 0.091263616 | 38.66521983 | Trunk fat percentage | ukb-b-16407 |
| rs11135450 | G | A | 0.668896 | 0.0133742 | 0.0019733 | 1.20E-11 | 0.106595046 | 45.93560018 | Trunk fat percentage | ukb-b-16407 |
| rs11150745 | G | A | 0.317691 | -0.0140471 | 0.00199291 | 1.80E-12 | 0.114294793 | 49.68187474 | Trunk fat percentage | ukb-b-16407 |
| rs11156614 | A | G | 0.591269 | -0.0116674 | 0.00188735 | 6.30E-10 | 0.090298676 | 38.21582888 | Trunk fat percentage | ukb-b-16407 |
| rs11159110 | T | C | 0.142833 | -0.015414 | 0.00265913 | 6.80E-09 | 0.080269648 | 33.60095104 | Trunk fat percentage | ukb-b-16407 |
| rs11165643 | T | C | 0.590143 | 0.0173083 | 0.00187612 | 2.80E-20 | 0.181045128 | 85.11137404 | Trunk fat percentage | ukb-b-16407 |
| rs11205303 | C | T | 0.406636 | 0.028955 | 0.00188076 | 1.80E-53 | 0.381046335 | 237.0174816 | Trunk fat percentage | ukb-b-16407 |
| rs11208779 | C | G | 0.528889 | 0.0118697 | 0.00185406 | 1.50E-10 | 0.096213705 | 40.98565842 | Trunk fat percentage | ukb-b-16407 |
| rs11222371 | T | C | 0.409216 | 0.0148444 | 0.00188286 | 3.20E-15 | 0.139004646 | 62.15688453 | Trunk fat percentage | ukb-b-16407 |
| rs11241192 | T | C | 0.433986 | 0.0117535 | 0.00187159 | 3.40E-10 | 0.092917798 | 39.43782824 | Trunk fat percentage | ukb-b-16407 |
| rs112710809 | T | C | 0.063528 | 0.0313597 | 0.00383684 | 3.00E-16 | 0.147858822 | 66.80306989 | Trunk fat percentage | ukb-b-16407 |
| rs112852122 | A | G | 0.157839 | -0.017849 | 0.00258153 | 4.70E-12 | 0.110453923 | 47.80501143 | Trunk fat percentage | ukb-b-16407 |
| rs113019802 | A | G | 0.225075 | -0.015202 | 0.00222096 | 7.70E-12 | 0.108489056 | 46.85112057 | Trunk fat percentage | ukb-b-16407 |
| rs113848804 | A | G | 0.050918 | -0.0263332 | 0.00421317 | 4.10E-10 | 0.092120597 | 39.06513316 | Trunk fat percentage | ukb-b-16407 |
| rs11538 | G | A | 0.171836 | 0.0139925 | 0.00245565 | 0.000000012 | 0.077773982 | 32.46816139 | Trunk fat percentage | ukb-b-16407 |
| rs11549690 | A | G | 0.075976 | -0.0204437 | 0.00349125 | 4.70E-09 | 0.081779311 | 34.28918014 | Trunk fat percentage | ukb-b-16407 |
| rs11659979 | A | G | 0.261741 | 0.012441 | 0.00210783 | 3.60E-09 | 0.082977208 | 34.83689322 | Trunk fat percentage | ukb-b-16407 |
| rs11664106 | T | A | 0.373945 | 0.0141557 | 0.00196258 | 5.50E-13 | 0.119042537 | 52.02450571 | Trunk fat percentage | ukb-b-16407 |
| rs11664848 | G | C | 0.659619 | 0.0118405 | 0.00196351 | 1.60E-09 | 0.08630108 | 36.36418423 | Trunk fat percentage | ukb-b-16407 |
| rs11666808 | C | T | 0.625302 | -0.0225597 | 0.00192138 | 7.80E-32 | 0.263666046 | 137.8605823 | Trunk fat percentage | ukb-b-16407 |
| rs117642733 | T | C | 0.045315 | 0.0272561 | 0.00471075 | 7.20E-09 | 0.079997361 | 33.47706028 | Trunk fat percentage | ukb-b-16407 |
| rs11782341 | G | A | 0.188826 | 0.0138598 | 0.00238337 | 6.10E-09 | 0.080743397 | 33.8166817 | Trunk fat percentage | ukb-b-16407 |
| rs11786089 | G | A | 0.459702 | 0.013852 | 0.0018606 | 9.70E-14 | 0.125847706 | 55.42668854 | Trunk fat percentage | ukb-b-16407 |
| rs11790018 | G | C | 0.367489 | -0.0140904 | 0.00192604 | 2.60E-13 | 0.122046869 | 53.51999196 | Trunk fat percentage | ukb-b-16407 |
| rs1182199 | A | C | 0.304436 | -0.0168202 | 0.00201191 | 6.30E-17 | 0.153650574 | 69.89485567 | Trunk fat percentage | ukb-b-16407 |
| rs11856579 | A | G | 0.267381 | -0.0114968 | 0.0020953 | 4.10E-08 | 0.072527365 | 30.10658712 | Trunk fat percentage | ukb-b-16407 |
| rs11866219 | C | A | 0.583801 | -0.0184739 | 0.00190279 | 2.80E-22 | 0.19668113 | 94.26174086 | Trunk fat percentage | ukb-b-16407 |
| rs12042959 | G | A | 0.143935 | -0.0163479 | 0.00264444 | 6.30E-10 | 0.090301177 | 38.21699271 | Trunk fat percentage | ukb-b-16407 |
| rs12072739 | G | A | 0.224599 | 0.0159189 | 0.0022163 | 6.80E-13 | 0.118166622 | 51.59041439 | Trunk fat percentage | ukb-b-16407 |
| rs12124748 | T | A | 0.011794 | 0.0512641 | 0.00870248 | 3.80E-09 | 0.082680047 | 34.70088945 | Trunk fat percentage | ukb-b-16407 |
| rs1218822 | A | G | 0.661446 | 0.0121275 | 0.00195964 | 6.10E-10 | 0.090477898 | 38.29922406 | Trunk fat percentage | ukb-b-16407 |
| rs12204724 | T | C | 0.302316 | 0.012907 | 0.0020171 | 1.60E-10 | 0.096126411 | 40.94451785 | Trunk fat percentage | ukb-b-16407 |
| rs12218858 | T | C | 0.573614 | 0.0123395 | 0.00187181 | 4.30E-11 | 0.101429236 | 43.4581864 | Trunk fat percentage | ukb-b-16407 |
| rs12251016 | T | A | 0.344281 | 0.0184185 | 0.00194859 | 3.30E-21 | 0.188353526 | 89.34444964 | Trunk fat percentage | ukb-b-16407 |
| rs1229984 | C | T | 0.972782 | 0.0349561 | 0.00561236 | 4.70E-10 | 0.091537908 | 38.79313741 | Trunk fat percentage | ukb-b-16407 |
| rs12402939 | C | A | 0.392361 | -0.0123225 | 0.00189401 | 7.70E-11 | 0.099053822 | 42.32852336 | Trunk fat percentage | ukb-b-16407 |
| rs12441543 | A | G | 0.287098 | 0.0141101 | 0.00205095 | 6.00E-12 | 0.109479595 | 47.33147488 | Trunk fat percentage | ukb-b-16407 |
| rs12475388 | A | G | 0.485701 | -0.0122487 | 0.00185722 | 4.20E-11 | 0.101509366 | 43.49639781 | Trunk fat percentage | ukb-b-16407 |
| rs12530737 | C | T | 0.261664 | -0.0124208 | 0.00210743 | 3.80E-09 | 0.082759055 | 34.73704102 | Trunk fat percentage | ukb-b-16407 |
| rs12533489 | C | G | 0.156999 | -0.0145158 | 0.00256291 | 0.000000015 | 0.076912574 | 32.07858775 | Trunk fat percentage | ukb-b-16407 |
| rs12562784 | G | A | 0.112118 | 0.0174695 | 0.00293104 | 2.50E-09 | 0.084474775 | 35.52363994 | Trunk fat percentage | ukb-b-16407 |
| rs12601535 | G | C | 0.505765 | -0.010376 | 0.00185465 | 2.20E-08 | 0.075184829 | 31.299399 | Trunk fat percentage | ukb-b-16407 |
| rs12619178 | T | C | 0.400827 | -0.0162459 | 0.00188302 | 6.30E-18 | 0.162014367 | 74.4350845 | Trunk fat percentage | ukb-b-16407 |
| rs12722976 | G | C | 0.499946 | -0.0109885 | 0.00185193 | 3.00E-09 | 0.083784662 | 35.20689224 | Trunk fat percentage | ukb-b-16407 |
| rs12724928 | C | T | 0.204874 | -0.0192804 | 0.00228655 | 3.40E-17 | 0.155887192 | 71.10017577 | Trunk fat percentage | ukb-b-16407 |
| rs12890931 | G | T | 0.362447 | 0.0136316 | 0.0019389 | 2.10E-12 | 0.113779474 | 49.42911633 | Trunk fat percentage | ukb-b-16407 |
| rs12926311 | C | G | 0.353543 | -0.0106277 | 0.00194311 | 4.50E-08 | 0.072098302 | 29.91464129 | Trunk fat percentage | ukb-b-16407 |
| rs12926311 | C | G | 0.353543 | -0.0106277 | 0.00194311 | 4.50E-08 | 0.072098302 | 29.91464129 | Trunk fat percentage | ukb-b-16407 |
| rs12997625 | T | C | 0.526093 | 0.0118515 | 0.00185215 | 1.60E-10 | 0.096126125 | 40.94438288 | Trunk fat percentage | ukb-b-16407 |
| rs13084418 | A | G | 0.129841 | -0.018531 | 0.0027504 | 1.60E-11 | 0.105472439 | 45.39478813 | Trunk fat percentage | ukb-b-16407 |
| rs13106087 | C | T | 0.829778 | 0.0144489 | 0.00246059 | 4.30E-09 | 0.082201141 | 34.48188994 | Trunk fat percentage | ukb-b-16407 |
| rs13107325 | T | C | 0.074856 | 0.0374858 | 0.00351679 | 1.60E-26 | 0.22786321 | 113.6163141 | Trunk fat percentage | ukb-b-16407 |
| rs13132853 | G | A | 0.360206 | -0.0107249 | 0.00193409 | 2.90E-08 | 0.073960829 | 30.74915205 | Trunk fat percentage | ukb-b-16407 |
| rs13174863 | G | A | 0.14813 | 0.015568 | 0.00262339 | 3.00E-09 | 0.083804502 | 35.21599215 | Trunk fat percentage | ukb-b-16407 |
| rs13292699 | C | A | 0.433727 | -0.0228139 | 0.00187073 | 3.30E-34 | 0.278651437 | 148.7225576 | Trunk fat percentage | ukb-b-16407 |
| rs1329733 | G | A | 0.474965 | 0.0130543 | 0.00185771 | 2.10E-12 | 0.113679326 | 49.38002894 | Trunk fat percentage | ukb-b-16407 |
| rs13389219 | T | C | 0.392419 | 0.0222149 | 0.00188901 | 6.30E-32 | 0.264283468 | 138.299373 | Trunk fat percentage | ukb-b-16407 |
| rs13408397 | T | C | 0.411071 | -0.0165034 | 0.00187489 | 1.30E-18 | 0.167533385 | 77.48100879 | Trunk fat percentage | ukb-b-16407 |
| rs13598 | A | C | 0.872983 | 0.0162263 | 0.00278897 | 6.00E-09 | 0.080815263 | 33.84942665 | Trunk fat percentage | ukb-b-16407 |
| rs1377184 | T | A | 0.748943 | 0.0164247 | 0.00213817 | 1.60E-14 | 0.132898382 | 59.00793655 | Trunk fat percentage | ukb-b-16407 |
| rs1421334 | C | A | 0.548865 | -0.0126513 | 0.00186937 | 1.30E-11 | 0.106316962 | 45.80150734 | Trunk fat percentage | ukb-b-16407 |
| rs1441264 | A | G | 0.593697 | 0.0132439 | 0.0019268 | 6.30E-12 | 0.109302033 | 47.24528906 | Trunk fat percentage | ukb-b-16407 |
| rs1453055 | A | G | 0.273372 | 0.0148704 | 0.00208208 | 9.20E-13 | 0.116991577 | 51.00943072 | Trunk fat percentage | ukb-b-16407 |
| rs1454687 | G | C | 0.515347 | -0.0149943 | 0.0018484 | 5.00E-16 | 0.145972658 | 65.80523917 | Trunk fat percentage | ukb-b-16407 |
| rs1475860 | C | G | 0.505361 | 0.0107914 | 0.00186299 | 6.90E-09 | 0.08016482 | 33.55324545 | Trunk fat percentage | ukb-b-16407 |
| rs1526579 | C | T | 0.330477 | 0.0118312 | 0.0019722 | 0.000000002 | 0.085484257 | 35.98783188 | Trunk fat percentage | ukb-b-16407 |
| rs1568488 | C | G | 0.594846 | 0.0141627 | 0.00189811 | 8.60E-14 | 0.126337511 | 55.67360672 | Trunk fat percentage | ukb-b-16407 |
| rs1605898 | A | T | 0.837613 | 0.0156999 | 0.00251029 | 4.00E-10 | 0.092227854 | 39.11523829 | Trunk fat percentage | ukb-b-16407 |
| rs1624064 | C | T | 0.420353 | -0.013572 | 0.00187682 | 4.80E-13 | 0.11958317 | 52.2928674 | Trunk fat percentage | ukb-b-16407 |
| rs1689265 | G | A | 0.909222 | 0.0192477 | 0.0032208 | 2.30E-09 | 0.084887532 | 35.71331521 | Trunk fat percentage | ukb-b-16407 |
| rs16916303 | G | A | 0.119771 | -0.0172216 | 0.00288209 | 2.30E-09 | 0.084869961 | 35.70523687 | Trunk fat percentage | ukb-b-16407 |
| rs16934748 | C | T | 0.150343 | 0.0153166 | 0.00259189 | 3.40E-09 | 0.083161747 | 34.92139702 | Trunk fat percentage | ukb-b-16407 |
| rs16996657 | C | T | 0.127758 | 0.015297 | 0.00278338 | 3.90E-08 | 0.07274544 | 30.20421332 | Trunk fat percentage | ukb-b-16407 |
| rs1701839 | A | G | 0.548765 | -0.0144443 | 0.00186456 | 9.40E-15 | 0.134855374 | 60.01230026 | Trunk fat percentage | ukb-b-16407 |
| rs17024393 | C | T | 0.025892 | 0.0567648 | 0.00583222 | 2.20E-22 | 0.197466296 | 94.73063061 | Trunk fat percentage | ukb-b-16407 |
| rs17055384 | T | C | 0.182958 | -0.0140905 | 0.00240617 | 4.70E-09 | 0.081786762 | 34.29258258 | Trunk fat percentage | ukb-b-16407 |
| rs17055653 | A | C | 0.30815 | 0.0180764 | 0.00200339 | 1.80E-19 | 0.174551019 | 81.41283583 | Trunk fat percentage | ukb-b-16407 |
| rs17122891 | C | T | 0.221559 | -0.0123988 | 0.00222581 | 0.000000025 | 0.074586183 | 31.03009683 | Trunk fat percentage | ukb-b-16407 |
| rs17172722 | T | C | 0.418951 | -0.0126808 | 0.00187753 | 1.40E-11 | 0.105932477 | 45.61624534 | Trunk fat percentage | ukb-b-16407 |
| rs1724557 | A | C | 0.586762 | -0.0122174 | 0.00188788 | 9.70E-11 | 0.098107633 | 41.88020653 | Trunk fat percentage | ukb-b-16407 |
| rs17522122 | T | G | 0.471222 | 0.0140161 | 0.00186256 | 5.30E-14 | 0.128226237 | 56.62834001 | Trunk fat percentage | ukb-b-16407 |
| rs17639546 | A | G | 0.148475 | -0.0156775 | 0.00260062 | 1.70E-09 | 0.086251339 | 36.34124676 | Trunk fat percentage | ukb-b-16407 |
| rs17681686 | C | G | 0.304791 | 0.01546 | 0.00198534 | 6.90E-15 | 0.136071255 | 60.63860389 | Trunk fat percentage | ukb-b-16407 |
| rs17704028 | T | C | 0.147185 | -0.0175921 | 0.00261591 | 1.80E-11 | 0.10512184 | 45.22616616 | Trunk fat percentage | ukb-b-16407 |
| rs17724992 | G | A | 0.267757 | -0.0152835 | 0.00209798 | 3.20E-13 | 0.121143519 | 53.06925062 | Trunk fat percentage | ukb-b-16407 |
| rs1775255 | T | G | 0.476694 | 0.0107023 | 0.00185558 | 0.000000008 | 0.079532158 | 33.26556281 | Trunk fat percentage | ukb-b-16407 |
| rs17770336 | T | C | 0.322441 | 0.0169741 | 0.0019763 | 8.80E-18 | 0.160795797 | 73.76795967 | Trunk fat percentage | ukb-b-16407 |
| rs1782508 | G | C | 0.655503 | -0.0109085 | 0.00194486 | 0.00000002 | 0.075540611 | 31.45961355 | Trunk fat percentage | ukb-b-16407 |
| rs1801282 | G | C | 0.119541 | 0.0367594 | 0.00284203 | 2.90E-38 | 0.302907095 | 167.2936714 | Trunk fat percentage | ukb-b-16407 |
| rs1808629 | A | G | 0.685427 | -0.0191182 | 0.00200111 | 1.30E-21 | 0.191643567 | 91.27504939 | Trunk fat percentage | ukb-b-16407 |
| rs1813039 | A | G | 0.709755 | 0.01239 | 0.00204985 | 1.50E-09 | 0.086669389 | 36.53410295 | Trunk fat percentage | ukb-b-16407 |
| rs1859927 | C | T | 0.157061 | 0.0143929 | 0.00253398 | 1.30E-08 | 0.077318157 | 32.261923 | Trunk fat percentage | ukb-b-16407 |
| rs1861410 | T | C | 0.555358 | -0.0156751 | 0.001864 | 4.10E-17 | 0.155178954 | 70.71781346 | Trunk fat percentage | ukb-b-16407 |
| rs1866745 | A | G | 0.350327 | -0.0108851 | 0.00194543 | 2.20E-08 | 0.075200461 | 31.30643597 | Trunk fat percentage | ukb-b-16407 |
| rs1881505 | C | T | 0.943027 | -0.0225376 | 0.00402418 | 0.000000021 | 0.075332984 | 31.36610102 | Trunk fat percentage | ukb-b-16407 |
| rs1893659 | A | C | 0.460062 | -0.0189545 | 0.00186906 | 3.60E-24 | 0.210812994 | 102.8438151 | Trunk fat percentage | ukb-b-16407 |
| rs1893659 | A | C | 0.460062 | -0.0189545 | 0.00186906 | 3.60E-24 | 0.210812994 | 102.8438151 | Trunk fat percentage | ukb-b-16407 |
| rs1906252 | A | C | 0.484423 | -0.0185546 | 0.00185721 | 1.70E-23 | 0.205877143 | 99.81163406 | Trunk fat percentage | ukb-b-16407 |
| rs1916039 | G | A | 0.252678 | 0.0134683 | 0.00214056 | 3.10E-10 | 0.093240022 | 39.58865543 | Trunk fat percentage | ukb-b-16407 |
| rs2002023 | T | C | 0.409042 | 0.0123494 | 0.0018848 | 5.70E-11 | 0.100320204 | 42.93002776 | Trunk fat percentage | ukb-b-16407 |
| rs2008018 | A | G | 0.322077 | 0.0120878 | 0.00197659 | 9.60E-10 | 0.08853976 | 37.39911659 | Trunk fat percentage | ukb-b-16407 |
| rs2008882 | G | A | 0.138333 | 0.0153904 | 0.00269877 | 0.000000012 | 0.077891384 | 32.52131269 | Trunk fat percentage | ukb-b-16407 |
| rs2034947 | C | T | 0.209729 | 0.0141094 | 0.00227738 | 5.80E-10 | 0.090659229 | 38.38363378 | Trunk fat percentage | ukb-b-16407 |
| rs2043016 | T | C | 0.37587 | 0.0135282 | 0.00191063 | 1.40E-12 | 0.115213789 | 50.13336378 | Trunk fat percentage | ukb-b-16407 |
| rs2058526 | G | A | 0.730204 | 0.0115682 | 0.0020862 | 2.90E-08 | 0.073958708 | 30.74819982 | Trunk fat percentage | ukb-b-16407 |
| rs2062506 | G | A | 0.736727 | 0.0204061 | 0.00210283 | 2.90E-22 | 0.196527009 | 94.16980967 | Trunk fat percentage | ukb-b-16407 |
| rs2108635 | G | A | 0.338505 | 0.011141 | 0.00196211 | 1.40E-08 | 0.077270763 | 32.24049092 | Trunk fat percentage | ukb-b-16407 |
| rs2111281 | C | A | 0.366939 | 0.0149623 | 0.00192128 | 6.80E-15 | 0.136089174 | 60.64784766 | Trunk fat percentage | ukb-b-16407 |
| rs215614 | A | G | 0.627841 | -0.0132009 | 0.00191694 | 5.70E-12 | 0.109668309 | 47.42311124 | Trunk fat percentage | ukb-b-16407 |
| rs217672 | C | A | 0.271729 | 0.0152415 | 0.00208752 | 2.90E-13 | 0.121622667 | 53.30821379 | Trunk fat percentage | ukb-b-16407 |
| rs2178899 | T | A | 0.128825 | -0.0220277 | 0.0027575 | 1.40E-15 | 0.142181177 | 63.81272107 | Trunk fat percentage | ukb-b-16407 |
| rs2190788 | T | G | 0.319705 | 0.012624 | 0.00199285 | 2.40E-10 | 0.094389851 | 40.12774452 | Trunk fat percentage | ukb-b-16407 |
| rs2192527 | G | A | 0.465491 | 0.0177804 | 0.00185556 | 9.50E-22 | 0.192565908 | 91.8191039 | Trunk fat percentage | ukb-b-16407 |
| rs2243928 | G | C | 0.649 | -0.0135528 | 0.00196453 | 5.20E-12 | 0.110017431 | 47.59274217 | Trunk fat percentage | ukb-b-16407 |
| rs2253310 | G | C | 0.626164 | 0.0124104 | 0.00191071 | 8.30E-11 | 0.098756035 | 42.18732667 | Trunk fat percentage | ukb-b-16407 |
| rs2274224 | C | G | 0.435476 | -0.0193757 | 0.00186649 | 3.00E-25 | 0.218688897 | 107.7614603 | Trunk fat percentage | ukb-b-16407 |
| rs2279178 | G | A | 0.822637 | -0.0147999 | 0.00242307 | 0.000000001 | 0.088340077 | 37.30659742 | Trunk fat percentage | ukb-b-16407 |
| rs2291127 | T | C | 0.156219 | -0.016829 | 0.00254498 | 3.80E-11 | 0.101992262 | 43.72681785 | Trunk fat percentage | ukb-b-16407 |
| rs2307111 | C | T | 0.395083 | -0.0196208 | 0.00189298 | 3.60E-25 | 0.218169191 | 107.4339072 | Trunk fat percentage | ukb-b-16407 |
| rs239458 | A | G | 0.911483 | 0.0209557 | 0.00327922 | 1.70E-10 | 0.095900074 | 40.83788453 | Trunk fat percentage | ukb-b-16407 |
| rs2447832 | T | C | 0.439637 | 0.0128706 | 0.00186281 | 4.90E-12 | 0.110315324 | 47.73758726 | Trunk fat percentage | ukb-b-16407 |
| rs2460 | A | G | 0.26262 | 0.0137667 | 0.0021091 | 6.70E-11 | 0.099637335 | 42.60546932 | Trunk fat percentage | ukb-b-16407 |
| rs2481899 | G | A | 0.551186 | 0.0105067 | 0.00187431 | 0.000000021 | 0.075459678 | 31.42315729 | Trunk fat percentage | ukb-b-16407 |
| rs252749 | A | G | 0.245982 | -0.0137038 | 0.00214429 | 1.60E-10 | 0.095910405 | 40.84275064 | Trunk fat percentage | ukb-b-16407 |
| rs253443 | A | T | 0.178208 | -0.0134911 | 0.00242017 | 0.000000025 | 0.074684756 | 31.07441607 | Trunk fat percentage | ukb-b-16407 |
| rs256112 | A | G | 0.768437 | -0.0153995 | 0.00219431 | 2.30E-12 | 0.113416488 | 49.25125207 | Trunk fat percentage | ukb-b-16407 |
| rs262953 | A | G | 0.62597 | -0.0122351 | 0.00191069 | 1.50E-10 | 0.096254292 | 41.00478936 | Trunk fat percentage | ukb-b-16407 |
| rs2678204 | G | T | 0.34018 | 0.0150388 | 0.00194932 | 1.20E-14 | 0.133896481 | 59.51961182 | Trunk fat percentage | ukb-b-16407 |
| rs2692741 | C | G | 0.367168 | 0.0105802 | 0.00193412 | 4.50E-08 | 0.072119428 | 29.92408799 | Trunk fat percentage | ukb-b-16407 |
| rs2702123 | C | T | 0.08602 | -0.0184408 | 0.00329235 | 0.000000021 | 0.075346937 | 31.37238387 | Trunk fat percentage | ukb-b-16407 |
| rs2717609 | T | A | 0.466079 | -0.0124485 | 0.00187142 | 2.90E-11 | 0.103082147 | 44.24778287 | Trunk fat percentage | ukb-b-16407 |
| rs2731238 | T | G | 0.067518 | -0.0259387 | 0.00404566 | 1.40E-10 | 0.096471449 | 41.10717666 | Trunk fat percentage | ukb-b-16407 |
| rs2731238 | T | G | 0.067518 | -0.0259387 | 0.00404566 | 1.40E-10 | 0.096471449 | 41.10717666 | Trunk fat percentage | ukb-b-16407 |
| rs2737265 | G | A | 0.280686 | -0.0210238 | 0.00206118 | 2.00E-24 | 0.212739574 | 104.0376643 | Trunk fat percentage | ukb-b-16407 |
| rs2785988 | A | C | 0.29737 | 0.0281228 | 0.00201822 | 3.90E-44 | 0.335254585 | 194.1690946 | Trunk fat percentage | ukb-b-16407 |
| rs2802774 | A | C | 0.547303 | 0.0137664 | 0.00188379 | 2.70E-13 | 0.121815076 | 53.40424682 | Trunk fat percentage | ukb-b-16407 |
| rs2814993 | A | G | 0.139756 | 0.033122 | 0.00266207 | 1.50E-35 | 0.286783808 | 154.8082721 | Trunk fat percentage | ukb-b-16407 |
| rs28375268 | T | G | 0.645136 | -0.0133749 | 0.00194362 | 5.90E-12 | 0.109526372 | 47.35418523 | Trunk fat percentage | ukb-b-16407 |
| rs2855818 | A | G | 0.241528 | 0.0199972 | 0.00218201 | 5.00E-20 | 0.179086069 | 83.98948285 | Trunk fat percentage | ukb-b-16407 |
| rs28651380 | G | A | 0.281237 | 0.0125919 | 0.00206059 | 9.90E-10 | 0.088416827 | 37.3421532 | Trunk fat percentage | ukb-b-16407 |
| rs2868182 | A | G | 0.254038 | 0.0125739 | 0.00212604 | 3.30E-09 | 0.083285696 | 34.97817464 | Trunk fat percentage | ukb-b-16407 |
| rs28742003 | T | C | 0.20527 | -0.0245178 | 0.00229262 | 1.10E-26 | 0.229023177 | 114.3665032 | Trunk fat percentage | ukb-b-16407 |
| rs2875238 | C | T | 0.637428 | 0.0107561 | 0.00193883 | 2.90E-08 | 0.074023472 | 30.7772775 | Trunk fat percentage | ukb-b-16407 |
| rs2943653 | T | C | 0.672837 | -0.0203304 | 0.00196799 | 5.10E-25 | 0.217034184 | 106.7200628 | Trunk fat percentage | ukb-b-16407 |
| rs2954033 | G | A | 0.695193 | 0.0154166 | 0.00200957 | 1.70E-14 | 0.132596318 | 58.85331515 | Trunk fat percentage | ukb-b-16407 |
| rs2960420 | G | C | 0.350751 | -0.0114396 | 0.0019455 | 4.10E-09 | 0.082404295 | 34.57476268 | Trunk fat percentage | ukb-b-16407 |
| rs2966859 | G | A | 0.789002 | -0.0139664 | 0.00227794 | 8.70E-10 | 0.088953717 | 37.5910443 | Trunk fat percentage | ukb-b-16407 |
| rs2984618 | T | G | 0.416213 | 0.0132292 | 0.00187482 | 1.70E-12 | 0.114516427 | 49.79067441 | Trunk fat percentage | ukb-b-16407 |
| rs301808 | G | C | 0.339813 | 0.0129987 | 0.00194964 | 2.60E-11 | 0.10350859 | 44.45196752 | Trunk fat percentage | ukb-b-16407 |
| rs3113509 | T | C | 0.731977 | -0.0129746 | 0.0020893 | 5.30E-10 | 0.091047268 | 38.5643794 | Trunk fat percentage | ukb-b-16407 |
| rs314288 | C | T | 0.886236 | -0.0228972 | 0.00291624 | 4.10E-15 | 0.13802347 | 61.64789188 | Trunk fat percentage | ukb-b-16407 |
| rs3213182 | C | A | 0.110389 | 0.0179102 | 0.00295862 | 1.40E-09 | 0.086911023 | 36.64565543 | Trunk fat percentage | ukb-b-16407 |
| rs3218036 | A | G | 0.326373 | 0.0143382 | 0.00198163 | 4.60E-13 | 0.119704841 | 52.35330834 | Trunk fat percentage | ukb-b-16407 |
| rs33503 | A | G | 0.805912 | -0.0162143 | 0.0023352 | 3.80E-12 | 0.11128805 | 48.21123363 | Trunk fat percentage | ukb-b-16407 |
| rs34483452 | A | C | 0.136313 | 0.0279556 | 0.00271811 | 8.20E-25 | 0.215534462 | 105.7800039 | Trunk fat percentage | ukb-b-16407 |
| rs34580448 | C | T | 0.04126 | -0.042294 | 0.00465889 | 1.10E-19 | 0.176316262 | 82.41240893 | Trunk fat percentage | ukb-b-16407 |
| rs34656389 | G | A | 0.367466 | 0.0117072 | 0.00191915 | 1.10E-09 | 0.088136784 | 37.21244748 | Trunk fat percentage | ukb-b-16407 |
| rs34675417 | G | A | 0.25472 | 0.0157555 | 0.00213556 | 1.60E-13 | 0.123865629 | 54.43031211 | Trunk fat percentage | ukb-b-16407 |
| rs34898535 | T | C | 0.377923 | -0.0167055 | 0.00191051 | 2.20E-18 | 0.165687055 | 76.45754113 | Trunk fat percentage | ukb-b-16407 |
| rs35142762 | C | T | 0.172365 | -0.0313638 | 0.00244355 | 1.00E-37 | 0.299676584 | 164.7460048 | Trunk fat percentage | ukb-b-16407 |
| rs35154152 | C | T | 0.106839 | -0.0249985 | 0.00299152 | 6.50E-17 | 0.1535305 | 69.83032753 | Trunk fat percentage | ukb-b-16407 |
| rs35307904 | A | G | 0.122373 | -0.019949 | 0.00284002 | 2.20E-12 | 0.113597716 | 49.3400362 | Trunk fat percentage | ukb-b-16407 |
| rs35523808 | A | T | 0.049075 | 0.025985 | 0.00439243 | 3.30E-09 | 0.083327652 | 34.99739701 | Trunk fat percentage | ukb-b-16407 |
| rs35697691 | G | C | 0.089408 | 0.0240825 | 0.00329382 | 2.60E-13 | 0.121920447 | 53.45685609 | Trunk fat percentage | ukb-b-16407 |
| rs3730071 | A | C | 0.030234 | -0.0318104 | 0.00540497 | 0.000000004 | 0.082542377 | 34.63791043 | Trunk fat percentage | ukb-b-16407 |
| rs3743861 | C | G | 0.41526 | -0.0136258 | 0.00187841 | 4.00E-13 | 0.120239452 | 52.61907835 | Trunk fat percentage | ukb-b-16407 |
| rs3754963 | T | A | 0.255089 | -0.0123494 | 0.00211809 | 5.50E-09 | 0.081132534 | 33.99404898 | Trunk fat percentage | ukb-b-16407 |
| rs3764002 | T | C | 0.261487 | -0.0201098 | 0.0021049 | 1.30E-21 | 0.191643742 | 91.27515236 | Trunk fat percentage | ukb-b-16407 |
| rs3766823 | A | G | 0.172024 | 0.0156538 | 0.00244761 | 1.60E-10 | 0.096038292 | 40.90299638 | Trunk fat percentage | ukb-b-16407 |
| rs3796074 | A | G | 0.617254 | 0.0120027 | 0.00190456 | 2.90E-10 | 0.093512501 | 39.71628191 | Trunk fat percentage | ukb-b-16407 |
| rs3803286 | G | A | 0.666745 | -0.0126363 | 0.00196427 | 1.30E-10 | 0.097059065 | 41.38447865 | Trunk fat percentage | ukb-b-16407 |
| rs3817428 | G | C | 0.265015 | -0.0227113 | 0.00209911 | 2.80E-27 | 0.233161472 | 117.0613674 | Trunk fat percentage | ukb-b-16407 |
| rs3826408 | T | C | 0.456846 | 0.0110021 | 0.00185839 | 3.20E-09 | 0.083440587 | 35.04914727 | Trunk fat percentage | ukb-b-16407 |
| rs3911063 | C | T | 0.321995 | -0.0126723 | 0.00198232 | 1.60E-10 | 0.095960009 | 40.86611625 | Trunk fat percentage | ukb-b-16407 |
| rs3923501 | T | C | 0.475803 | 0.0118532 | 0.00185097 | 1.50E-10 | 0.09626188 | 41.008366 | Trunk fat percentage | ukb-b-16407 |
| rs3943933 | A | T | 0.480747 | 0.0123464 | 0.0018504 | 2.50E-11 | 0.103649365 | 44.51941479 | Trunk fat percentage | ukb-b-16407 |
| rs394608 | C | T | 0.537741 | 0.0139743 | 0.00186625 | 7.00E-14 | 0.127120105 | 56.06869957 | Trunk fat percentage | ukb-b-16407 |
| rs40067 | A | G | 0.170174 | -0.0148852 | 0.00246871 | 1.60E-09 | 0.086282081 | 36.3554225 | Trunk fat percentage | ukb-b-16407 |
| rs41307479 | G | C | 0.220559 | 0.0135984 | 0.00223291 | 1.10E-09 | 0.087867891 | 37.08798054 | Trunk fat percentage | ukb-b-16407 |
| rs41310284 | A | C | 0.101003 | -0.0256494 | 0.00309243 | 1.10E-16 | 0.151598636 | 68.79464997 | Trunk fat percentage | ukb-b-16407 |
| rs41313250 | G | A | 0.121484 | -0.02685 | 0.00291111 | 2.90E-20 | 0.180971311 | 85.06900396 | Trunk fat percentage | ukb-b-16407 |
| rs4245812 | G | A | 0.364368 | -0.0111356 | 0.00192172 | 6.80E-09 | 0.080217893 | 33.57739693 | Trunk fat percentage | ukb-b-16407 |
| rs429343 | G | A | 0.576551 | -0.0120072 | 0.00187166 | 1.40E-10 | 0.096574242 | 41.15566005 | Trunk fat percentage | ukb-b-16407 |
| rs429358 | C | T | 0.154032 | -0.0238871 | 0.00256823 | 1.40E-20 | 0.183471856 | 86.50854863 | Trunk fat percentage | ukb-b-16407 |
| rs4311684 | C | T | 0.706685 | 0.0117758 | 0.00203405 | 7.10E-09 | 0.080083878 | 33.51641763 | Trunk fat percentage | ukb-b-16407 |
| rs4377469 | T | G | 0.886785 | 0.0169483 | 0.00291048 | 5.80E-09 | 0.080947363 | 33.90962969 | Trunk fat percentage | ukb-b-16407 |
| rs4398538 | C | T | 0.642499 | -0.011567 | 0.00193394 | 2.20E-09 | 0.085017358 | 35.77300959 | Trunk fat percentage | ukb-b-16407 |
| rs441792 | G | A | 0.486583 | 0.0143157 | 0.00185187 | 1.10E-14 | 0.134362886 | 59.75911882 | Trunk fat percentage | ukb-b-16407 |
| rs4466418 | A | G | 0.562236 | 0.0120158 | 0.00186837 | 1.30E-10 | 0.097007039 | 41.35991251 | Trunk fat percentage | ukb-b-16407 |
| rs4500770 | T | A | 0.36261 | -0.0116386 | 0.00192806 | 1.60E-09 | 0.086462183 | 36.43849199 | Trunk fat percentage | ukb-b-16407 |
| rs4549685 | T | C | 0.329793 | -0.0150579 | 0.00196928 | 2.10E-14 | 0.131841509 | 58.46741283 | Trunk fat percentage | ukb-b-16407 |
| rs458741 | C | T | 0.74305 | -0.0172523 | 0.00211903 | 3.90E-16 | 0.146881882 | 66.28569169 | Trunk fat percentage | ukb-b-16407 |
| rs4616635 | G | C | 0.278738 | 0.016131 | 0.00205891 | 4.70E-15 | 0.137511888 | 61.38296415 | Trunk fat percentage | ukb-b-16407 |
| rs464881 | T | C | 0.545285 | 0.0101978 | 0.00186199 | 4.30E-08 | 0.072279435 | 29.99565101 | Trunk fat percentage | ukb-b-16407 |
| rs4709745 | C | T | 0.307179 | 0.0116873 | 0.00200628 | 5.70E-09 | 0.081002581 | 33.93480019 | Trunk fat percentage | ukb-b-16407 |
| rs4718964 | T | G | 0.413116 | 0.013029 | 0.00188531 | 4.80E-12 | 0.110359662 | 47.75915409 | Trunk fat percentage | ukb-b-16407 |
| rs4721319 | G | A | 0.832639 | 0.0143858 | 0.00248271 | 6.90E-09 | 0.080212631 | 33.57500216 | Trunk fat percentage | ukb-b-16407 |
| rs4722398 | T | C | 0.136099 | 0.0174497 | 0.00269253 | 9.10E-11 | 0.098361787 | 42.00053553 | Trunk fat percentage | ukb-b-16407 |
| rs4759318 | T | C | 0.362187 | 0.0129903 | 0.0019292 | 1.70E-11 | 0.105359061 | 45.34024423 | Trunk fat percentage | ukb-b-16407 |
| rs479018 | A | G | 0.332501 | -0.0169252 | 0.00199738 | 2.40E-17 | 0.157187029 | 71.80360097 | Trunk fat percentage | ukb-b-16407 |
| rs4790841 | T | C | 0.154518 | -0.023748 | 0.00257358 | 2.80E-20 | 0.18111039 | 85.14883978 | Trunk fat percentage | ukb-b-16407 |
| rs4820325 | A | G | 0.580936 | -0.0223221 | 0.00188289 | 2.00E-32 | 0.267429313 | 140.5465537 | Trunk fat percentage | ukb-b-16407 |
| rs4880340 | G | C | 0.575755 | -0.0118022 | 0.00187422 | 3.00E-10 | 0.093379123 | 39.65379928 | Trunk fat percentage | ukb-b-16407 |
| rs4945884 | C | T | 0.709609 | 0.0132713 | 0.00204295 | 8.20E-11 | 0.098782568 | 42.1999035 | Trunk fat percentage | ukb-b-16407 |
| rs4959158 | A | G | 0.590478 | 0.0127642 | 0.00193652 | 4.40E-11 | 0.101402299 | 43.44534273 | Trunk fat percentage | ukb-b-16407 |
| rs543874 | G | A | 0.205268 | 0.0341053 | 0.00228605 | 2.50E-50 | 0.366331192 | 222.572907 | Trunk fat percentage | ukb-b-16407 |
| rs55707359 | G | T | 0.015446 | 0.0431563 | 0.00760203 | 1.40E-08 | 0.077242464 | 32.22769528 | Trunk fat percentage | ukb-b-16407 |
| rs56218501 | T | C | 0.211813 | -0.0157429 | 0.00226625 | 3.70E-12 | 0.111380451 | 48.25627995 | Trunk fat percentage | ukb-b-16407 |
| rs56254492 | T | C | 0.301061 | 0.0117344 | 0.00202603 | 7.00E-09 | 0.080147078 | 33.54517277 | Trunk fat percentage | ukb-b-16407 |
| rs56299211 | A | G | 0.190712 | 0.0132609 | 0.00239149 | 2.90E-08 | 0.073957 | 30.74743285 | Trunk fat percentage | ukb-b-16407 |
| rs56399737 | T | C | 0.449181 | -0.0126968 | 0.00186874 | 1.10E-11 | 0.107065526 | 46.1626566 | Trunk fat percentage | ukb-b-16407 |
| rs57636386 | C | T | 0.083839 | -0.0294964 | 0.00335374 | 1.40E-18 | 0.167303574 | 77.35337137 | Trunk fat percentage | ukb-b-16407 |
| rs57800857 | C | A | 0.364909 | -0.0147919 | 0.00193204 | 1.90E-14 | 0.132132167 | 58.61593502 | Trunk fat percentage | ukb-b-16407 |
| rs57989773 | C | T | 0.244919 | 0.0142888 | 0.00221248 | 1.10E-10 | 0.097746386 | 41.70929104 | Trunk fat percentage | ukb-b-16407 |
| rs58862095 | T | C | 0.419268 | -0.0140171 | 0.00187992 | 8.90E-14 | 0.126182116 | 55.59523967 | Trunk fat percentage | ukb-b-16407 |
| rs59397548 | G | A | 0.318902 | -0.0136317 | 0.00199037 | 7.40E-12 | 0.108603228 | 46.90643268 | Trunk fat percentage | ukb-b-16407 |
| rs59499656 | T | A | 0.343225 | -0.0174621 | 0.00195715 | 4.60E-19 | 0.171340523 | 79.60580118 | Trunk fat percentage | ukb-b-16407 |
| rs595127 | C | T | 0.569832 | 0.0115072 | 0.00187097 | 7.70E-10 | 0.089462829 | 37.82732885 | Trunk fat percentage | ukb-b-16407 |
| rs6008598 | T | C | 0.10707 | 0.0178374 | 0.0030121 | 3.20E-09 | 0.083484071 | 35.06907659 | Trunk fat percentage | ukb-b-16407 |
| rs6021948 | A | T | 0.321776 | -0.0147096 | 0.00198768 | 1.40E-13 | 0.124533851 | 54.76571857 | Trunk fat percentage | ukb-b-16407 |
| rs60498692 | A | C | 0.191223 | -0.0131082 | 0.00236107 | 2.80E-08 | 0.074124259 | 30.82253767 | Trunk fat percentage | ukb-b-16407 |
| rs6103254 | C | T | 0.126713 | -0.016265 | 0.00279547 | 5.90E-09 | 0.080823328 | 33.8531015 | Trunk fat percentage | ukb-b-16407 |
| rs61412028 | A | T | 0.149521 | 0.015109 | 0.002595 | 5.80E-09 | 0.080925725 | 33.89976738 | Trunk fat percentage | ukb-b-16407 |
| rs61903695 | G | A | 0.254902 | 0.0137916 | 0.00212648 | 8.80E-11 | 0.098494985 | 42.06362535 | Trunk fat percentage | ukb-b-16407 |
| rs61910767 | T | C | 0.164194 | -0.0164797 | 0.00250052 | 4.40E-11 | 0.10138021 | 43.43481117 | Trunk fat percentage | ukb-b-16407 |
| rs61969510 | C | T | 0.279007 | 0.0152793 | 0.00208295 | 2.20E-13 | 0.122623695 | 53.80829439 | Trunk fat percentage | ukb-b-16407 |
| rs61975147 | C | T | 0.167197 | -0.017911 | 0.00248851 | 6.10E-13 | 0.118597236 | 51.80371283 | Trunk fat percentage | ukb-b-16407 |
| rs61986205 | G | A | 0.082021 | 0.022475 | 0.00340087 | 3.90E-11 | 0.101880851 | 43.67363484 | Trunk fat percentage | ukb-b-16407 |
| rs62025831 | C | T | 0.280576 | -0.0308425 | 0.0020644 | 1.80E-50 | 0.366993775 | 223.2088685 | Trunk fat percentage | ukb-b-16407 |
| rs62218301 | G | A | 0.166069 | -0.0145373 | 0.00250476 | 6.50E-09 | 0.080454061 | 33.68490039 | Trunk fat percentage | ukb-b-16407 |
| rs62413414 | T | C | 0.15172 | 0.0145355 | 0.00257397 | 0.000000016 | 0.07649475 | 31.88988765 | Trunk fat percentage | ukb-b-16407 |
| rs62499697 | C | T | 0.292749 | 0.0122759 | 0.00206034 | 2.50E-09 | 0.084423402 | 35.50004436 | Trunk fat percentage | ukb-b-16407 |
| rs62621197 | T | C | 0.037196 | -0.0425108 | 0.00508522 | 6.30E-17 | 0.153630768 | 69.88421048 | Trunk fat percentage | ukb-b-16407 |
| rs6436891 | G | A | 0.330003 | 0.0170992 | 0.00196657 | 3.50E-18 | 0.164137204 | 75.60190948 | Trunk fat percentage | ukb-b-16407 |
| rs6438086 | G | A | 0.647981 | -0.0106138 | 0.00193678 | 4.30E-08 | 0.072360216 | 30.03178984 | Trunk fat percentage | ukb-b-16407 |
| rs6466053 | T | C | 0.655173 | -0.0118551 | 0.00194716 | 1.10E-09 | 0.087826188 | 37.0686837 | Trunk fat percentage | ukb-b-16407 |
| rs6491427 | G | A | 0.288991 | -0.0158078 | 0.00204286 | 1.00E-14 | 0.134593766 | 59.8777752 | Trunk fat percentage | ukb-b-16407 |
| rs6500594 | G | T | 0.246615 | 0.0136543 | 0.00215137 | 2.20E-10 | 0.094717862 | 40.28178105 | Trunk fat percentage | ukb-b-16407 |
| rs6561937 | A | T | 0.753704 | -0.0122324 | 0.00215651 | 1.40E-08 | 0.077126208 | 32.17513631 | Trunk fat percentage | ukb-b-16407 |
| rs6567160 | C | T | 0.232647 | 0.0291238 | 0.0021927 | 2.90E-40 | 0.314233848 | 176.4158684 | Trunk fat percentage | ukb-b-16407 |
| rs6575340 | A | G | 0.636011 | 0.0152351 | 0.00192998 | 2.90E-15 | 0.13930689 | 62.3139098 | Trunk fat percentage | ukb-b-16407 |
| rs6688826 | C | T | 0.298206 | 0.0119576 | 0.0020155 | 3.00E-09 | 0.083766059 | 35.19836075 | Trunk fat percentage | ukb-b-16407 |
| rs6693294 | G | A | 0.688576 | -0.0154383 | 0.00199445 | 9.90E-15 | 0.134670756 | 59.91735665 | Trunk fat percentage | ukb-b-16407 |
| rs6699744 | T | A | 0.616053 | 0.0146069 | 0.00190693 | 1.90E-14 | 0.132245989 | 58.67412339 | Trunk fat percentage | ukb-b-16407 |
| rs6699744 | T | A | 0.616053 | 0.0146069 | 0.00190693 | 1.90E-14 | 0.132245989 | 58.67412339 | Trunk fat percentage | ukb-b-16407 |
| rs6711390 | T | C | 0.371273 | -0.0142351 | 0.00190003 | 6.80E-14 | 0.127242703 | 56.13065736 | Trunk fat percentage | ukb-b-16407 |
| rs6735393 | C | A | 0.18053 | 0.0148328 | 0.00241648 | 8.30E-10 | 0.089139653 | 37.67730855 | Trunk fat percentage | ukb-b-16407 |
| rs6752378 | A | C | 0.486262 | 0.0235654 | 0.00184711 | 2.80E-37 | 0.297145334 | 162.7661581 | Trunk fat percentage | ukb-b-16407 |
| rs6754292 | T | C | 0.64463 | -0.0121627 | 0.00194407 | 3.90E-10 | 0.092283815 | 39.14138533 | Trunk fat percentage | ukb-b-16407 |
| rs6777968 | A | G | 0.560158 | -0.0110162 | 0.00186013 | 3.20E-09 | 0.083493356 | 35.07333229 | Trunk fat percentage | ukb-b-16407 |
| rs6782581 | G | C | 0.439298 | -0.0119274 | 0.00186243 | 1.50E-10 | 0.096273736 | 41.01395503 | Trunk fat percentage | ukb-b-16407 |
| rs6791296 | C | T | 0.881901 | 0.0177141 | 0.00287357 | 7.10E-10 | 0.089836591 | 38.00096472 | Trunk fat percentage | ukb-b-16407 |
| rs6840236 | C | T | 0.464888 | 0.0154746 | 0.00185594 | 7.60E-17 | 0.152952934 | 69.5201979 | Trunk fat percentage | ukb-b-16407 |
| rs6843910 | A | T | 0.454542 | -0.0123957 | 0.00186186 | 2.80E-11 | 0.10324332 | 44.32493123 | Trunk fat percentage | ukb-b-16407 |
| rs6847975 | A | G | 0.356168 | 0.0136974 | 0.00193921 | 1.60E-12 | 0.114721725 | 49.89150363 | Trunk fat percentage | ukb-b-16407 |
| rs685149 | G | A | 0.645395 | -0.0117355 | 0.00193742 | 1.40E-09 | 0.087008493 | 36.69066965 | Trunk fat percentage | ukb-b-16407 |
| rs6875133 | C | G | 0.25351 | -0.0123498 | 0.00220332 | 0.000000021 | 0.075446005 | 31.41699881 | Trunk fat percentage | ukb-b-16407 |
| rs6948959 | A | G | 0.744368 | -0.0154997 | 0.00213629 | 4.00E-13 | 0.120284017 | 52.64124713 | Trunk fat percentage | ukb-b-16407 |
| rs6950442 | G | A | 0.799787 | 0.013862 | 0.00231201 | 0.000000002 | 0.085397297 | 35.94780463 | Trunk fat percentage | ukb-b-16407 |
| rs6973700 | G | A | 0.206156 | -0.0135025 | 0.00228958 | 3.70E-09 | 0.082850674 | 34.77897083 | Trunk fat percentage | ukb-b-16407 |
| rs6977416 | A | G | 0.334253 | -0.0133896 | 0.0019787 | 1.30E-11 | 0.106294108 | 45.79049082 | Trunk fat percentage | ukb-b-16407 |
| rs7020 | A | G | 0.437446 | 0.0162957 | 0.00186755 | 2.60E-18 | 0.165108958 | 76.13801771 | Trunk fat percentage | ukb-b-16407 |
| rs704061 | C | T | 0.455053 | 0.0166826 | 0.0018581 | 2.70E-19 | 0.173127852 | 80.61007161 | Trunk fat percentage | ukb-b-16407 |
| rs704255 | A | G | 0.417154 | -0.012397 | 0.00187873 | 4.20E-11 | 0.101604155 | 43.54160781 | Trunk fat percentage | ukb-b-16407 |
| rs7102705 | G | A | 0.796614 | 0.0143774 | 0.00229922 | 4.00E-10 | 0.09219965 | 39.10206147 | Trunk fat percentage | ukb-b-16407 |
| rs7124681 | A | C | 0.408409 | 0.0261686 | 0.00187831 | 4.10E-44 | 0.335175734 | 194.1004027 | Trunk fat percentage | ukb-b-16407 |
| rs7132908 | A | G | 0.384448 | 0.0230288 | 0.00190329 | 1.10E-33 | 0.275494826 | 146.397171 | Trunk fat percentage | ukb-b-16407 |
| rs7133378 | A | G | 0.319424 | 0.0238076 | 0.00198809 | 4.80E-33 | 0.271389868 | 143.4033024 | Trunk fat percentage | ukb-b-16407 |
| rs71476581 | A | G | 0.022886 | 0.0359315 | 0.00629452 | 1.10E-08 | 0.078033299 | 32.58558032 | Trunk fat percentage | ukb-b-16407 |
| rs7151700 | A | C | 0.457499 | -0.0103135 | 0.00185659 | 2.80E-08 | 0.074205168 | 30.85887804 | Trunk fat percentage | ukb-b-16407 |
| rs71658797 | A | T | 0.120746 | 0.02941 | 0.00284017 | 4.00E-25 | 0.217839459 | 107.2263141 | Trunk fat percentage | ukb-b-16407 |
| rs7171864 | A | G | 0.660091 | 0.0164885 | 0.00196437 | 4.70E-17 | 0.154692634 | 70.45563141 | Trunk fat percentage | ukb-b-16407 |
| rs718609 | A | G | 0.273824 | 0.0119132 | 0.00207203 | 8.90E-09 | 0.079073176 | 33.05710274 | Trunk fat percentage | ukb-b-16407 |
| rs718947 | G | A | 0.361042 | 0.0111337 | 0.00197936 | 1.90E-08 | 0.075939725 | 31.63948828 | Trunk fat percentage | ukb-b-16407 |
| rs719802 | C | T | 0.614394 | -0.0132609 | 0.00189971 | 2.90E-12 | 0.112345254 | 48.72719171 | Trunk fat percentage | ukb-b-16407 |
| rs7206608 | G | C | 0.321558 | 0.0112148 | 0.00198368 | 0.000000016 | 0.076655427 | 31.96243344 | Trunk fat percentage | ukb-b-16407 |
| rs7218014 | C | T | 0.197303 | 0.0258438 | 0.00233217 | 1.50E-28 | 0.241824944 | 122.7982942 | Trunk fat percentage | ukb-b-16407 |
| rs7246182 | C | T | 0.222968 | -0.0123977 | 0.00223241 | 2.80E-08 | 0.074166295 | 30.8414173 | Trunk fat percentage | ukb-b-16407 |
| rs72617140 | C | A | 0.213877 | 0.0150368 | 0.00225386 | 2.50E-11 | 0.103629585 | 44.50993649 | Trunk fat percentage | ukb-b-16407 |
| rs72634814 | A | G | 0.345468 | -0.0146917 | 0.0020293 | 4.50E-13 | 0.119828025 | 52.41451752 | Trunk fat percentage | ukb-b-16407 |
| rs72681698 | C | T | 0.01072 | -0.057665 | 0.00899135 | 1.40E-10 | 0.096523069 | 41.13152254 | Trunk fat percentage | ukb-b-16407 |
| rs72697297 | C | T | 0.179924 | -0.0182516 | 0.00243696 | 6.90E-14 | 0.127167277 | 56.09253698 | Trunk fat percentage | ukb-b-16407 |
| rs72755233 | A | G | 0.11146 | -0.0258695 | 0.00293999 | 1.40E-18 | 0.167433567 | 77.42556144 | Trunk fat percentage | ukb-b-16407 |
| rs72767957 | G | A | 0.200143 | -0.0148861 | 0.00231716 | 1.30E-10 | 0.096819662 | 41.27145866 | Trunk fat percentage | ukb-b-16407 |
| rs72892910 | T | G | 0.172208 | 0.0242367 | 0.00245304 | 5.10E-23 | 0.202270533 | 97.61975524 | Trunk fat percentage | ukb-b-16407 |
| rs72917533 | C | T | 0.18553 | -0.0165247 | 0.00238192 | 4.00E-12 | 0.111120682 | 48.12966352 | Trunk fat percentage | ukb-b-16407 |
| rs7295386 | G | C | 0.35486 | -0.0109157 | 0.00194532 | 0.00000002 | 0.075599757 | 31.48626011 | Trunk fat percentage | ukb-b-16407 |
| rs72976986 | A | G | 0.190219 | -0.0188685 | 0.00238298 | 2.40E-15 | 0.140039827 | 62.6951517 | Trunk fat percentage | ukb-b-16407 |
| rs72995085 | C | T | 0.177045 | -0.0151826 | 0.00242798 | 4.00E-10 | 0.092200119 | 39.10228084 | Trunk fat percentage | ukb-b-16407 |
| rs73213501 | C | A | 0.171849 | -0.0177083 | 0.00245233 | 5.20E-13 | 0.119281311 | 52.14298864 | Trunk fat percentage | ukb-b-16407 |
| rs73374119 | G | A | 0.14437 | 0.0145208 | 0.00264143 | 3.90E-08 | 0.072782002 | 30.22058559 | Trunk fat percentage | ukb-b-16407 |
| rs7426945 | G | A | 0.546465 | 0.0113725 | 0.00185713 | 9.10E-10 | 0.088756654 | 37.49965565 | Trunk fat percentage | ukb-b-16407 |
| rs7442885 | G | C | 0.214119 | -0.0126453 | 0.00225823 | 0.000000021 | 0.075310756 | 31.35609193 | Trunk fat percentage | ukb-b-16407 |
| rs74576293 | C | T | 0.089741 | -0.0224582 | 0.00323254 | 3.70E-12 | 0.11140512 | 48.26830796 | Trunk fat percentage | ukb-b-16407 |
| rs74618095 | C | T | 0.157979 | 0.0154601 | 0.00255885 | 1.50E-09 | 0.086603156 | 36.50353646 | Trunk fat percentage | ukb-b-16407 |
| rs7477105 | T | C | 0.328597 | -0.0115737 | 0.00198121 | 5.20E-09 | 0.081421477 | 34.1258454 | Trunk fat percentage | ukb-b-16407 |
| rs75135487 | G | C | 0.136925 | -0.0152404 | 0.00268759 | 1.40E-08 | 0.077084639 | 32.15634639 | Trunk fat percentage | ukb-b-16407 |
| rs7575523 | G | T | 0.603246 | -0.0176526 | 0.00189414 | 1.20E-20 | 0.184070819 | 86.85467705 | Trunk fat percentage | ukb-b-16407 |
| rs7589069 | G | C | 0.11953 | 0.0156165 | 0.00284469 | 0.00000004 | 0.072594924 | 30.13682657 | Trunk fat percentage | ukb-b-16407 |
| rs7598246 | C | T | 0.583707 | 0.0103722 | 0.00187595 | 0.000000032 | 0.073562212 | 30.57026807 | Trunk fat percentage | ukb-b-16407 |
| rs7604537 | A | T | 0.95596 | -0.0269517 | 0.00450419 | 2.20E-09 | 0.085086065 | 35.80460803 | Trunk fat percentage | ukb-b-16407 |
| rs76345589 | G | C | 0.067509 | -0.0223247 | 0.00369767 | 1.60E-09 | 0.086490299 | 36.45146299 | Trunk fat percentage | ukb-b-16407 |
| rs7637852 | G | A | 0.703892 | -0.0136448 | 0.00202448 | 1.60E-11 | 0.105537929 | 45.42630027 | Trunk fat percentage | ukb-b-16407 |
| rs764729 | C | A | 0.728027 | 0.0116921 | 0.00207874 | 1.90E-08 | 0.075932494 | 31.63622796 | Trunk fat percentage | ukb-b-16407 |
| rs7680610 | G | A | 0.652258 | 0.0108295 | 0.00195703 | 3.10E-08 | 0.073675685 | 30.62117454 | Trunk fat percentage | ukb-b-16407 |
| rs7707394 | A | G | 0.357279 | -0.0141784 | 0.00192729 | 1.90E-13 | 0.123247121 | 54.12031466 | Trunk fat percentage | ukb-b-16407 |
| rs7715639 | A | G | 0.367244 | -0.0118061 | 0.00192646 | 8.90E-10 | 0.088880695 | 37.55717545 | Trunk fat percentage | ukb-b-16407 |
| rs77165542 | T | C | 0.03548 | -0.0560257 | 0.00504921 | 1.30E-28 | 0.242304591 | 123.1197473 | Trunk fat percentage | ukb-b-16407 |
| rs7762794 | G | A | 0.28551 | 0.0111969 | 0.00204636 | 4.50E-08 | 0.072151885 | 29.93860241 | Trunk fat percentage | ukb-b-16407 |
| rs7826247 | G | C | 0.250102 | -0.0124407 | 0.00213998 | 6.10E-09 | 0.080698839 | 33.79638166 | Trunk fat percentage | ukb-b-16407 |
| rs78744936 | A | G | 0.265973 | 0.0142471 | 0.00211034 | 1.50E-11 | 0.105851498 | 45.57724645 | Trunk fat percentage | ukb-b-16407 |
| rs7906395 | G | A | 0.230442 | -0.0123326 | 0.00219704 | 0.00000002 | 0.075650024 | 31.50890883 | Trunk fat percentage | ukb-b-16407 |
| rs7925725 | C | A | 0.41127 | 0.0124676 | 0.00188294 | 3.60E-11 | 0.102233964 | 43.84224228 | Trunk fat percentage | ukb-b-16407 |
| rs79518326 | A | C | 0.02864 | -0.0394091 | 0.00560195 | 2.00E-12 | 0.113902989 | 49.4896722 | Trunk fat percentage | ukb-b-16407 |
| rs7975187 | G | A | 0.21388 | 0.0128166 | 0.00225782 | 1.40E-08 | 0.077232253 | 32.2230785 | Trunk fat percentage | ukb-b-16407 |
| rs8049439 | C | T | 0.402335 | 0.0296219 | 0.00188676 | 1.50E-55 | 0.390327077 | 246.486139 | Trunk fat percentage | ukb-b-16407 |
| rs8051058 | C | T | 0.755268 | 0.0148764 | 0.00215914 | 5.60E-12 | 0.109768182 | 47.47162389 | Trunk fat percentage | ukb-b-16407 |
| rs8059064 | T | G | 0.085317 | -0.0193278 | 0.00332065 | 5.90E-09 | 0.080878114 | 33.87806837 | Trunk fat percentage | ukb-b-16407 |
| rs8090017 | A | G | 0.334474 | 0.0130533 | 0.00197358 | 3.70E-11 | 0.102030924 | 43.7452767 | Trunk fat percentage | ukb-b-16407 |
| rs8103728 | G | C | 0.669971 | 0.0226791 | 0.00197459 | 1.60E-30 | 0.255198264 | 131.9160885 | Trunk fat percentage | ukb-b-16407 |
| rs811054 | T | C | 0.537263 | 0.0111312 | 0.00186905 | 2.60E-09 | 0.084354591 | 35.46844371 | Trunk fat percentage | ukb-b-16407 |
| rs8112818 | G | A | 0.400382 | -0.0163077 | 0.00189645 | 8.00E-18 | 0.161117601 | 73.94394772 | Trunk fat percentage | ukb-b-16407 |
| rs812949 | C | T | 0.72921 | 0.0160637 | 0.00208105 | 1.20E-14 | 0.134020978 | 59.58351775 | Trunk fat percentage | ukb-b-16407 |
| rs8139212 | A | G | 0.116506 | 0.0167989 | 0.00289146 | 6.30E-09 | 0.08060615 | 33.75416059 | Trunk fat percentage | ukb-b-16407 |
| rs8176166 | C | T | 0.148715 | -0.0145 | 0.00261388 | 2.90E-08 | 0.074013137 | 30.77263693 | Trunk fat percentage | ukb-b-16407 |
| rs8180470 | T | C | 0.53484 | -0.0110003 | 0.00186217 | 3.50E-09 | 0.083105373 | 34.89557888 | Trunk fat percentage | ukb-b-16407 |
| rs879620 | T | C | 0.613274 | 0.017349 | 0.00190691 | 9.20E-20 | 0.176951148 | 82.77296296 | Trunk fat percentage | ukb-b-16407 |
| rs890796 | A | C | 0.299066 | 0.0122278 | 0.00202298 | 1.50E-09 | 0.086672127 | 36.53536707 | Trunk fat percentage | ukb-b-16407 |
| rs9289630 | C | G | 0.389101 | 0.0174843 | 0.00190243 | 3.90E-20 | 0.179918361 | 84.46545532 | Trunk fat percentage | ukb-b-16407 |
| rs9289981 | T | A | 0.40174 | 0.011346 | 0.00188657 | 1.80E-09 | 0.085878221 | 36.16926723 | Trunk fat percentage | ukb-b-16407 |
| rs9304665 | A | T | 0.763639 | 0.0132645 | 0.00218566 | 1.30E-09 | 0.087312777 | 36.83125858 | Trunk fat percentage | ukb-b-16407 |
| rs9316661 | C | T | 0.801443 | -0.0171726 | 0.00232613 | 1.60E-13 | 0.124006462 | 54.50095889 | Trunk fat percentage | ukb-b-16407 |
| rs9358912 | T | G | 0.273345 | -0.0323318 | 0.00207523 | 1.00E-54 | 0.386681082 | 242.7321447 | Trunk fat percentage | ukb-b-16407 |
| rs9375441 | A | G | 0.455651 | -0.0132287 | 0.00185603 | 1.00E-12 | 0.116567384 | 50.80007482 | Trunk fat percentage | ukb-b-16407 |
| rs9382464 | G | A | 0.255809 | -0.0121135 | 0.00212596 | 0.000000012 | 0.077769261 | 32.46602429 | Trunk fat percentage | ukb-b-16407 |
| rs9568867 | A | G | 0.12917 | 0.0228829 | 0.00278407 | 2.00E-16 | 0.149275939 | 67.55567291 | Trunk fat percentage | ukb-b-16407 |
| rs957919 | T | C | 0.277991 | 0.0156495 | 0.00206389 | 3.40E-14 | 0.129933089 | 57.49470358 | Trunk fat percentage | ukb-b-16407 |
| rs9654453 | C | T | 0.129277 | 0.0151086 | 0.00276078 | 4.40E-08 | 0.072175523 | 29.94917383 | Trunk fat percentage | ukb-b-16407 |
| rs9657704 | A | T | 0.331061 | -0.0110623 | 0.00196584 | 1.80E-08 | 0.075998735 | 31.66609634 | Trunk fat percentage | ukb-b-16407 |
| rs972283 | G | A | 0.511941 | -0.0171558 | 0.00184907 | 1.70E-20 | 0.182733514 | 86.08257394 | Trunk fat percentage | ukb-b-16407 |
| rs9770544 | G | C | 0.820325 | -0.0190079 | 0.00243163 | 5.40E-15 | 0.136973538 | 61.10451359 | Trunk fat percentage | ukb-b-16407 |
| rs9783304 | T | G | 0.689277 | -0.0196216 | 0.00200076 | 1.00E-22 | 0.199881436 | 96.17868695 | Trunk fat percentage | ukb-b-16407 |
| rs9788550 | C | G | 0.247492 | -0.0180635 | 0.00215472 | 5.10E-17 | 0.154363649 | 70.27844139 | Trunk fat percentage | ukb-b-16407 |
| rs9814758 | G | T | 0.35578 | -0.015885 | 0.00194004 | 2.70E-16 | 0.148311004 | 67.04294288 | Trunk fat percentage | ukb-b-16407 |
| rs9843653 | C | T | 0.511542 | 0.0180268 | 0.0018497 | 1.90E-22 | 0.197884167 | 94.98055167 | Trunk fat percentage | ukb-b-16407 |
| rs987469 | G | C | 0.46359 | -0.0158953 | 0.00185697 | 1.10E-17 | 0.159884372 | 73.27025158 | Trunk fat percentage | ukb-b-16407 |
| rs9892466 | A | T | 0.333256 | 0.0122472 | 0.001966 | 4.70E-10 | 0.091566958 | 38.80668926 | Trunk fat percentage | ukb-b-16407 |

Supplementary Table 10: Information on all instrumental variables (IVs) ultimately used for Arm fat percentage (left) in our study

| SNPs | effect_allele.exposure | other_allele.exposure | eaf.exposure | beta.exposure | se.exposure | pval.exposure | R^2^ | F | trait | id.exposure |
| --- | --- | --- | --- | --- | --- | --- | --- | --- | --- | --- |
| rs10050620 | T | C | 0.325414 | -0.0116255 | 0.00162492 | 8.40E-13 | 0.116021012 | 51.18695708 | Arm fat percentage (left) | ukb-b-20188 |
| rs10070510 | C | T | 0.105229 | -0.0138041 | 0.00250534 | 3.60E-08 | 0.072220889 | 30.35867751 | Arm fat percentage (left) | ukb-b-20188 |
| rs10099330 | G | A | 0.452932 | 0.00882541 | 0.00153101 | 8.20E-09 | 0.07851246 | 33.22872872 | Arm fat percentage (left) | ukb-b-20188 |
| rs10100245 | A | G | 0.564469 | 0.016792 | 0.0015367 | 8.50E-28 | 0.234402636 | 119.4061426 | Arm fat percentage (left) | ukb-b-20188 |
| rs10116857 | A | C | 0.061827 | -0.0223594 | 0.00316473 | 1.60E-12 | 0.113468781 | 49.91682602 | Arm fat percentage (left) | ukb-b-20188 |
| rs1013402 | G | A | 0.318442 | 0.0205712 | 0.00163157 | 1.90E-36 | 0.289575187 | 158.9673119 | Arm fat percentage (left) | ukb-b-20188 |
| rs10144067 | T | C | 0.591295 | 0.0112414 | 0.00156213 | 6.20E-13 | 0.117218241 | 51.78529531 | Arm fat percentage (left) | ukb-b-20188 |
| rs10187101 | T | C | 0.363606 | -0.0106962 | 0.00157779 | 1.20E-11 | 0.105418306 | 45.95794821 | Arm fat percentage (left) | ukb-b-20188 |
| rs10195099 | G | T | 0.154278 | -0.0116774 | 0.00209936 | 2.70E-08 | 0.073501875 | 30.93986949 | Arm fat percentage (left) | ukb-b-20188 |
| rs10269783 | A | G | 0.387852 | 0.00859857 | 0.00156362 | 3.80E-08 | 0.071960148 | 30.24057389 | Arm fat percentage (left) | ukb-b-20188 |
| rs10280907 | T | G | 0.189668 | 0.010758 | 0.00194369 | 3.10E-08 | 0.072828995 | 30.63437888 | Arm fat percentage (left) | ukb-b-20188 |
| rs1038088 | G | T | 0.519316 | 0.0120221 | 0.0015223 | 2.80E-15 | 0.137869604 | 62.36776449 | Arm fat percentage (left) | ukb-b-20188 |
| rs10402950 | C | T | 0.28935 | 0.0161131 | 0.00168315 | 1.00E-21 | 0.190276338 | 91.64579901 | Arm fat percentage (left) | ukb-b-20188 |
| rs10423928 | A | T | 0.194424 | -0.0225049 | 0.00192264 | 1.20E-31 | 0.259978733 | 137.0118808 | Arm fat percentage (left) | ukb-b-20188 |
| rs1046080 | A | C | 0.721641 | 0.0162411 | 0.00169308 | 8.60E-22 | 0.190902699 | 92.01866363 | Arm fat percentage (left) | ukb-b-20188 |
| rs1048013 | T | C | 0.555522 | 0.00920245 | 0.00152736 | 1.70E-09 | 0.085154438 | 36.30146128 | Arm fat percentage (left) | ukb-b-20188 |
| rs10513935 | A | G | 0.301529 | -0.00958352 | 0.00166255 | 8.20E-09 | 0.078510286 | 33.22773028 | Arm fat percentage (left) | ukb-b-20188 |
| rs10756792 | T | C | 0.743073 | -0.015703 | 0.0017495 | 2.80E-19 | 0.171206124 | 80.56332234 | Arm fat percentage (left) | ukb-b-20188 |
| rs10760724 | C | T | 0.477318 | 0.010682 | 0.00152305 | 2.30E-12 | 0.11200164 | 49.19000031 | Arm fat percentage (left) | ukb-b-20188 |
| rs10783779 | G | T | 0.402968 | -0.00872902 | 0.00155424 | 0.00000002 | 0.074826175 | 31.54240571 | Arm fat percentage (left) | ukb-b-20188 |
| rs1078455 | C | T | 0.309495 | 0.0108112 | 0.00165704 | 6.80E-11 | 0.098407358 | 42.56786012 | Arm fat percentage (left) | ukb-b-20188 |
| rs10788497 | C | G | 0.499613 | 0.0102466 | 0.00152482 | 1.80E-11 | 0.103771169 | 45.1567218 | Arm fat percentage (left) | ukb-b-20188 |
| rs10799778 | G | T | 0.83368 | -0.0145844 | 0.00203999 | 8.70E-13 | 0.11587037 | 51.11178602 | Arm fat percentage (left) | ukb-b-20188 |
| rs10854853 | T | G | 0.456805 | 0.00952314 | 0.00153106 | 5.00E-10 | 0.090247384 | 38.68796775 | Arm fat percentage (left) | ukb-b-20188 |
| rs10886022 | C | A | 0.763356 | -0.0124009 | 0.00182694 | 1.10E-11 | 0.105656798 | 46.07420408 | Arm fat percentage (left) | ukb-b-20188 |
| rs10892501 | A | G | 0.607115 | -0.00951763 | 0.00157026 | 1.40E-09 | 0.086090111 | 36.73791452 | Arm fat percentage (left) | ukb-b-20188 |
| rs10922199 | A | G | 0.329252 | 0.00903956 | 0.0016378 | 3.40E-08 | 0.072451111 | 30.46301259 | Arm fat percentage (left) | ukb-b-20188 |
| rs10938398 | A | G | 0.433505 | 0.0211619 | 0.00153761 | 4.30E-43 | 0.326908608 | 189.4161155 | Arm fat percentage (left) | ukb-b-20188 |
| rs10998 | A | G | 0.746786 | 0.0165592 | 0.00175144 | 3.20E-21 | 0.186465868 | 89.38984309 | Arm fat percentage (left) | ukb-b-20188 |
| rs10999460 | T | C | 0.265498 | 0.0125838 | 0.0017317 | 3.70E-13 | 0.119251924 | 52.8053956 | Arm fat percentage (left) | ukb-b-20188 |
| rs11012732 | G | A | 0.331706 | 0.0144403 | 0.00161756 | 4.40E-19 | 0.169674119 | 79.69510258 | Arm fat percentage (left) | ukb-b-20188 |
| rs11017772 | T | C | 0.205913 | -0.0110707 | 0.00188723 | 4.50E-09 | 0.081079976 | 34.41125415 | Arm fat percentage (left) | ukb-b-20188 |
| rs11030016 | T | C | 0.739769 | 0.0130365 | 0.00173835 | 6.40E-14 | 0.126031409 | 56.24029294 | Arm fat percentage (left) | ukb-b-20188 |
| rs1103804 | G | A | 0.766885 | -0.010778 | 0.00180295 | 2.30E-09 | 0.083939883 | 35.73625124 | Arm fat percentage (left) | ukb-b-20188 |
| rs11078687 | A | G | 0.613595 | 0.01104 | 0.00156623 | 1.80E-12 | 0.113001763 | 49.68520318 | Arm fat percentage (left) | ukb-b-20188 |
| rs11079849 | T | C | 0.328546 | -0.0156965 | 0.00162538 | 4.60E-22 | 0.192981153 | 93.26008902 | Arm fat percentage (left) | ukb-b-20188 |
| rs11105842 | A | G | 0.367522 | -0.0104761 | 0.0015861 | 4.00E-11 | 0.100605924 | 43.62527126 | Arm fat percentage (left) | ukb-b-20188 |
| rs11150745 | G | A | 0.317686 | -0.0132422 | 0.00163907 | 6.50E-16 | 0.143368776 | 65.27175392 | Arm fat percentage (left) | ukb-b-20188 |
| rs11165643 | T | C | 0.590152 | 0.0148041 | 0.00154287 | 8.40E-22 | 0.190984309 | 92.0672881 | Arm fat percentage (left) | ukb-b-20188 |
| rs11172106 | G | C | 0.451611 | 0.00862449 | 0.00153333 | 1.90E-08 | 0.075033783 | 31.63702081 | Arm fat percentage (left) | ukb-b-20188 |
| rs11173522 | A | C | 0.21428 | 0.0112539 | 0.00185601 | 1.30E-09 | 0.086150092 | 36.76592356 | Arm fat percentage (left) | ukb-b-20188 |
| rs11215381 | C | T | 0.525804 | 0.00844505 | 0.00152644 | 0.000000032 | 0.072772403 | 30.6087063 | Arm fat percentage (left) | ukb-b-20188 |
| rs11222371 | T | C | 0.409212 | 0.0125038 | 0.00154842 | 6.70E-16 | 0.143250387 | 65.20884269 | Arm fat percentage (left) | ukb-b-20188 |
| rs11223970 | G | A | 0.164579 | 0.0127845 | 0.00205566 | 5.00E-10 | 0.090226403 | 38.67808153 | Arm fat percentage (left) | ukb-b-20188 |
| rs112852122 | A | G | 0.157855 | -0.0175006 | 0.00212298 | 1.70E-16 | 0.148385805 | 67.95385066 | Arm fat percentage (left) | ukb-b-20188 |
| rs114964326 | A | G | 0.028988 | -0.0253037 | 0.00456286 | 2.90E-08 | 0.073091368 | 30.753445 | Arm fat percentage (left) | ukb-b-20188 |
| rs11648621 | G | A | 0.214847 | -0.0106967 | 0.00186088 | 9.00E-09 | 0.078105151 | 33.04173889 | Arm fat percentage (left) | ukb-b-20188 |
| rs11659979 | A | G | 0.26175 | 0.0103215 | 0.00173322 | 2.60E-09 | 0.083352038 | 35.46322685 | Arm fat percentage (left) | ukb-b-20188 |
| rs11664848 | G | C | 0.659624 | 0.0108278 | 0.00161457 | 2.00E-11 | 0.103395791 | 44.97453619 | Arm fat percentage (left) | ukb-b-20188 |
| rs11667638 | G | A | 0.52853 | 0.00874098 | 0.00152715 | 0.00000001 | 0.077492909 | 32.76097815 | Arm fat percentage (left) | ukb-b-20188 |
| rs11691869 | A | C | 0.361916 | -0.0138888 | 0.0015835 | 1.80E-18 | 0.164756263 | 76.92957138 | Arm fat percentage (left) | ukb-b-20188 |
| rs11699828 | A | G | 0.035832 | -0.0271623 | 0.0044843 | 1.40E-09 | 0.085986771 | 36.68966655 | Arm fat percentage (left) | ukb-b-20188 |
| rs117176448 | G | C | 0.096163 | 0.0157671 | 0.00257952 | 9.80E-10 | 0.087423928 | 37.36163258 | Arm fat percentage (left) | ukb-b-20188 |
| rs11767811 | A | G | 0.181438 | -0.0116151 | 0.00197283 | 3.90E-09 | 0.081624798 | 34.66303451 | Arm fat percentage (left) | ukb-b-20188 |
| rs11782074 | T | G | 0.383631 | 0.00975079 | 0.00159084 | 8.80E-10 | 0.08786596 | 37.56873759 | Arm fat percentage (left) | ukb-b-20188 |
| rs11786089 | G | A | 0.459693 | 0.0102596 | 0.00153022 | 2.00E-11 | 0.103350208 | 44.95242343 | Arm fat percentage (left) | ukb-b-20188 |
| rs118136827 | T | G | 0.281119 | -0.0106316 | 0.00169468 | 3.50E-10 | 0.091664952 | 39.35698749 | Arm fat percentage (left) | ukb-b-20188 |
| rs11852419 | T | A | 0.261665 | 0.0104615 | 0.00173398 | 1.60E-09 | 0.08536556 | 36.39986315 | Arm fat percentage (left) | ukb-b-20188 |
| rs11866219 | C | A | 0.583791 | -0.0193718 | 0.00156441 | 3.20E-35 | 0.28220962 | 153.3341134 | Arm fat percentage (left) | ukb-b-20188 |
| rs11967262 | G | C | 0.488654 | -0.0118527 | 0.00152223 | 6.90E-15 | 0.134541341 | 60.62811016 | Arm fat percentage (left) | ukb-b-20188 |
| rs12001437 | C | T | 0.367765 | 0.00929209 | 0.00157835 | 3.90E-09 | 0.081616791 | 34.65933182 | Arm fat percentage (left) | ukb-b-20188 |
| rs12033257 | G | A | 0.382348 | -0.0092296 | 0.00157428 | 4.60E-09 | 0.080994462 | 34.37176256 | Arm fat percentage (left) | ukb-b-20188 |
| rs12042959 | G | A | 0.143927 | -0.0134376 | 0.00217476 | 6.50E-10 | 0.089165268 | 38.17866563 | Arm fat percentage (left) | ukb-b-20188 |
| rs12054057 | A | G | 0.157626 | 0.0143311 | 0.00209166 | 7.30E-12 | 0.107436397 | 46.94365176 | Arm fat percentage (left) | ukb-b-20188 |
| rs12072739 | G | A | 0.224594 | 0.013523 | 0.00182267 | 1.20E-13 | 0.123687149 | 55.04653734 | Arm fat percentage (left) | ukb-b-20188 |
| rs12088284 | T | C | 0.300907 | 0.00946395 | 0.0016546 | 1.10E-08 | 0.077394528 | 32.71589716 | Arm fat percentage (left) | ukb-b-20188 |
| rs12140153 | T | G | 0.094279 | -0.0253502 | 0.00266362 | 1.80E-21 | 0.18847563 | 90.57706537 | Arm fat percentage (left) | ukb-b-20188 |
| rs12206087 | A | G | 0.482449 | -0.0145617 | 0.00152413 | 1.20E-21 | 0.189662498 | 91.28094675 | Arm fat percentage (left) | ukb-b-20188 |
| rs12282785 | A | C | 0.215759 | -0.0109944 | 0.00186982 | 4.10E-09 | 0.081431151 | 34.57350943 | Arm fat percentage (left) | ukb-b-20188 |
| rs1229984 | C | T | 0.972779 | 0.0286209 | 0.00461468 | 5.60E-10 | 0.089777282 | 38.46656366 | Arm fat percentage (left) | ukb-b-20188 |
| rs12338803 | C | A | 0.274778 | 0.0101545 | 0.00170678 | 2.70E-09 | 0.083208548 | 35.39663663 | Arm fat percentage (left) | ukb-b-20188 |
| rs12367809 | T | C | 0.369193 | 0.0218128 | 0.00158294 | 3.40E-43 | 0.327454515 | 189.8864291 | Arm fat percentage (left) | ukb-b-20188 |
| rs12375196 | A | C | 0.424294 | 0.0114177 | 0.00154916 | 1.70E-13 | 0.122255453 | 54.32061846 | Arm fat percentage (left) | ukb-b-20188 |
| rs12376870 | A | G | 0.237674 | -0.0114619 | 0.00179345 | 1.60E-10 | 0.094801242 | 40.8446034 | Arm fat percentage (left) | ukb-b-20188 |
| rs12422552 | C | G | 0.268302 | -0.00941263 | 0.00172401 | 4.80E-08 | 0.071005342 | 29.80865726 | Arm fat percentage (left) | ukb-b-20188 |
| rs12441543 | A | G | 0.28711 | 0.0104659 | 0.00168724 | 5.50E-10 | 0.08979912 | 38.47684342 | Arm fat percentage (left) | ukb-b-20188 |
| rs12459368 | G | A | 0.268188 | -0.013616 | 0.00171802 | 2.30E-15 | 0.138715414 | 62.81200476 | Arm fat percentage (left) | ukb-b-20188 |
| rs12471400 | C | T | 0.517362 | 0.00926823 | 0.00152568 | 1.20E-09 | 0.086444439 | 36.90342706 | Arm fat percentage (left) | ukb-b-20188 |
| rs12628603 | A | G | 0.617013 | -0.00982848 | 0.00157076 | 3.90E-10 | 0.091230907 | 39.15191868 | Arm fat percentage (left) | ukb-b-20188 |
| rs12635343 | A | G | 0.361431 | 0.0100059 | 0.00158162 | 2.50E-10 | 0.093071451 | 40.02285082 | Arm fat percentage (left) | ukb-b-20188 |
| rs12788343 | C | T | 0.409213 | 0.0114589 | 0.00154627 | 1.30E-13 | 0.123434102 | 54.91806147 | Arm fat percentage (left) | ukb-b-20188 |
| rs12881869 | T | C | 0.070527 | 0.0173623 | 0.00297767 | 5.50E-09 | 0.080185701 | 33.99862718 | Arm fat percentage (left) | ukb-b-20188 |
| rs12883788 | T | C | 0.459755 | 0.0130739 | 0.00153412 | 1.60E-17 | 0.156986277 | 72.62592119 | Arm fat percentage (left) | ukb-b-20188 |
| rs12885458 | G | T | 0.507979 | -0.0102054 | 0.00152574 | 2.20E-11 | 0.102912727 | 44.740311 | Arm fat percentage (left) | ukb-b-20188 |
| rs12890931 | G | T | 0.362434 | 0.0099162 | 0.00159454 | 5.00E-10 | 0.090217859 | 38.67405571 | Arm fat percentage (left) | ukb-b-20188 |
| rs12992672 | A | G | 0.828112 | 0.0314729 | 0.00200938 | 2.70E-55 | 0.386145081 | 245.3292737 | Arm fat percentage (left) | ukb-b-20188 |
| rs13030 | T | C | 0.327792 | 0.0089725 | 0.00162144 | 3.10E-08 | 0.072800405 | 30.6214089 | Arm fat percentage (left) | ukb-b-20188 |
| rs13084418 | A | G | 0.129837 | -0.0139191 | 0.00226171 | 7.50E-10 | 0.088518069 | 37.87463651 | Arm fat percentage (left) | ukb-b-20188 |
| rs13107325 | T | C | 0.07486 | 0.0369248 | 0.00289172 | 2.40E-37 | 0.294821084 | 163.0511349 | Arm fat percentage (left) | ukb-b-20188 |
| rs13160735 | A | G | 0.322387 | 0.0106401 | 0.00163465 | 7.60E-11 | 0.097991394 | 42.3683803 | Arm fat percentage (left) | ukb-b-20188 |
| rs13174863 | G | A | 0.148134 | 0.0151808 | 0.0021572 | 2.00E-12 | 0.112674779 | 49.5231766 | Arm fat percentage (left) | ukb-b-20188 |
| rs1320251 | T | C | 0.454747 | -0.0104228 | 0.00153479 | 1.10E-11 | 0.105746702 | 46.1180448 | Arm fat percentage (left) | ukb-b-20188 |
| rs1322842 | G | A | 0.609479 | -0.0111755 | 0.00156726 | 1.00E-12 | 0.115336162 | 50.84541854 | Arm fat percentage (left) | ukb-b-20188 |
| rs13292699 | C | A | 0.433717 | -0.0188416 | 0.00153837 | 1.70E-34 | 0.277788262 | 150.0078392 | Arm fat percentage (left) | ukb-b-20188 |
| rs13317303 | A | C | 0.150182 | -0.0153064 | 0.00212985 | 6.60E-13 | 0.11694252 | 51.64735446 | Arm fat percentage (left) | ukb-b-20188 |
| rs13340130 | T | A | 0.345041 | 0.00906216 | 0.00159869 | 1.40E-08 | 0.076117913 | 32.13179109 | Arm fat percentage (left) | ukb-b-20188 |
| rs1336899 | C | T | 0.393499 | -0.00872887 | 0.00155521 | 0.00000002 | 0.074737462 | 31.50198866 | Arm fat percentage (left) | ukb-b-20188 |
| rs13375176 | C | T | 0.201965 | -0.0130227 | 0.00190007 | 7.20E-12 | 0.107499563 | 46.97457613 | Arm fat percentage (left) | ukb-b-20188 |
| rs13389219 | T | C | 0.392416 | 0.0153266 | 0.00155283 | 5.60E-23 | 0.199867111 | 97.4190344 | Arm fat percentage (left) | ukb-b-20188 |
| rs13427822 | G | A | 0.271195 | -0.00976317 | 0.00172652 | 0.000000016 | 0.075779134 | 31.97705588 | Arm fat percentage (left) | ukb-b-20188 |
| rs1357079 | C | T | 0.570066 | 0.011137 | 0.00153838 | 4.50E-13 | 0.118463577 | 52.40940015 | Arm fat percentage (left) | ukb-b-20188 |
| rs1377184 | T | A | 0.748944 | 0.0111422 | 0.00175841 | 2.40E-10 | 0.093342662 | 40.15148475 | Arm fat percentage (left) | ukb-b-20188 |
| rs1383723 | T | A | 0.783151 | -0.0106374 | 0.00184993 | 8.90E-09 | 0.078154489 | 33.0643805 | Arm fat percentage (left) | ukb-b-20188 |
| rs14057 | A | G | 0.356592 | -0.00920871 | 0.00158932 | 6.90E-09 | 0.079258863 | 33.57182075 | Arm fat percentage (left) | ukb-b-20188 |
| rs1421334 | C | A | 0.548851 | -0.0105763 | 0.00153744 | 6.00E-12 | 0.108210392 | 47.32288033 | Arm fat percentage (left) | ukb-b-20188 |
| rs1436348 | G | A | 0.582742 | 0.0101006 | 0.00154125 | 5.60E-11 | 0.099200054 | 42.94851639 | Arm fat percentage (left) | ukb-b-20188 |
| rs1441264 | A | G | 0.59369 | 0.0132514 | 0.00158442 | 6.10E-17 | 0.152080316 | 69.94922313 | Arm fat percentage (left) | ukb-b-20188 |
| rs144376234 | T | C | 0.025644 | 0.0554265 | 0.0048133 | 1.10E-30 | 0.253733752 | 132.6016868 | Arm fat percentage (left) | ukb-b-20188 |
| rs1451963 | T | G | 0.082254 | 0.0171034 | 0.00277565 | 7.20E-10 | 0.088720223 | 37.96955446 | Arm fat percentage (left) | ukb-b-20188 |
| rs1453055 | A | G | 0.273342 | 0.0120533 | 0.00171158 | 1.90E-12 | 0.11281506 | 49.59267402 | Arm fat percentage (left) | ukb-b-20188 |
| rs1454687 | G | C | 0.515333 | -0.0144904 | 0.00151994 | 1.50E-21 | 0.189000813 | 90.88827469 | Arm fat percentage (left) | ukb-b-20188 |
| rs147819910 | T | G | 0.018205 | 0.0328444 | 0.0056924 | 7.90E-09 | 0.078648857 | 33.29138362 | Arm fat percentage (left) | ukb-b-20188 |
| rs147860177 | G | T | 0.232167 | 0.0121384 | 0.00181598 | 2.30E-11 | 0.102785626 | 44.6787246 | Arm fat percentage (left) | ukb-b-20188 |
| rs1503526 | C | T | 0.479968 | 0.0117572 | 0.00152204 | 1.10E-14 | 0.132697216 | 59.66995081 | Arm fat percentage (left) | ukb-b-20188 |
| rs1559900 | T | C | 0.28612 | 0.0105958 | 0.00168375 | 3.10E-10 | 0.092182066 | 39.60155934 | Arm fat percentage (left) | ukb-b-20188 |
| rs156914 | A | G | 0.491816 | 0.00868925 | 0.0015198 | 1.10E-08 | 0.077334187 | 32.68825256 | Arm fat percentage (left) | ukb-b-20188 |
| rs159037 | C | T | 0.253901 | 0.00975217 | 0.00174609 | 2.30E-08 | 0.074060601 | 31.19387133 | Arm fat percentage (left) | ukb-b-20188 |
| rs1689265 | G | A | 0.909227 | 0.0155158 | 0.00264879 | 4.70E-09 | 0.080866266 | 34.31257329 | Arm fat percentage (left) | ukb-b-20188 |
| rs16895838 | C | T | 0.12749 | 0.0133732 | 0.0022747 | 4.10E-09 | 0.081410306 | 34.56387486 | Arm fat percentage (left) | ukb-b-20188 |
| rs16916303 | G | A | 0.119778 | -0.0133138 | 0.00236995 | 1.90E-08 | 0.074862985 | 31.55917853 | Arm fat percentage (left) | ukb-b-20188 |
| rs17193211 | T | C | 0.066797 | -0.0175561 | 0.00310548 | 0.000000016 | 0.075740462 | 31.95940009 | Arm fat percentage (left) | ukb-b-20188 |
| rs1724557 | A | C | 0.586758 | -0.011907 | 0.0015524 | 1.70E-14 | 0.131073756 | 58.8298091 | Arm fat percentage (left) | ukb-b-20188 |
| rs17446299 | G | C | 0.165545 | 0.0119971 | 0.00205127 | 0.000000005 | 0.080636139 | 34.20636305 | Arm fat percentage (left) | ukb-b-20188 |
| rs17698176 | G | T | 0.226628 | 0.0101802 | 0.0018368 | 3.00E-08 | 0.073012645 | 30.71771304 | Arm fat percentage (left) | ukb-b-20188 |
| rs17770336 | T | C | 0.322443 | 0.0167569 | 0.00162515 | 6.30E-25 | 0.214211008 | 106.3164463 | Arm fat percentage (left) | ukb-b-20188 |
| rs17781552 | A | G | 0.545293 | 0.00857816 | 0.00154564 | 2.90E-08 | 0.073197177 | 30.80148024 | Arm fat percentage (left) | ukb-b-20188 |
| rs17820010 | G | T | 0.2956 | 0.0105805 | 0.00166673 | 2.20E-10 | 0.093651061 | 40.2978502 | Arm fat percentage (left) | ukb-b-20188 |
| rs1782508 | G | C | 0.655509 | -0.00889517 | 0.00159941 | 2.70E-08 | 0.073481557 | 30.93063891 | Arm fat percentage (left) | ukb-b-20188 |
| rs1788808 | G | A | 0.494876 | -0.0144077 | 0.00152636 | 3.80E-21 | 0.185972884 | 89.09951935 | Arm fat percentage (left) | ukb-b-20188 |
| rs1801282 | G | C | 0.119545 | 0.0229058 | 0.00233696 | 1.10E-22 | 0.197646641 | 96.07012834 | Arm fat percentage (left) | ukb-b-20188 |
| rs1808629 | A | G | 0.685432 | -0.0147576 | 0.00164579 | 3.00E-19 | 0.170927041 | 80.4049213 | Arm fat percentage (left) | ukb-b-20188 |
| rs1840126 | C | A | 0.563601 | -0.00845786 | 0.00153092 | 3.30E-08 | 0.072581686 | 30.52221104 | Arm fat percentage (left) | ukb-b-20188 |
| rs1852006 | A | G | 0.356957 | -0.0106449 | 0.00158794 | 2.00E-11 | 0.103320741 | 44.93812983 | Arm fat percentage (left) | ukb-b-20188 |
| rs1861410 | T | C | 0.555358 | -0.0138082 | 0.00153224 | 2.00E-19 | 0.172347166 | 81.21206364 | Arm fat percentage (left) | ukb-b-20188 |
| rs1912638 | C | G | 0.341483 | -0.00977216 | 0.0016198 | 1.60E-09 | 0.085358117 | 36.39639326 | Arm fat percentage (left) | ukb-b-20188 |
| rs1945160 | A | G | 0.375862 | -0.00870874 | 0.00157955 | 3.50E-08 | 0.072307498 | 30.39792184 | Arm fat percentage (left) | ukb-b-20188 |
| rs1964675 | T | C | 0.711088 | 0.0105482 | 0.00167921 | 3.30E-10 | 0.091880833 | 39.45905567 | Arm fat percentage (left) | ukb-b-20188 |
| rs1991002 | G | T | 0.495156 | -0.00929829 | 0.00152591 | 1.10E-09 | 0.086933312 | 37.13199949 | Arm fat percentage (left) | ukb-b-20188 |
| rs2034946 | G | T | 0.20882 | 0.0121868 | 0.00187678 | 8.40E-11 | 0.097567009 | 42.16505143 | Arm fat percentage (left) | ukb-b-20188 |
| rs2035936 | T | G | 0.055875 | 0.026095 | 0.00335635 | 7.60E-15 | 0.134194803 | 60.44774678 | Arm fat percentage (left) | ukb-b-20188 |
| rs2052607 | A | G | 0.343898 | -0.013924 | 0.00160842 | 4.80E-18 | 0.161186886 | 74.94265931 | Arm fat percentage (left) | ukb-b-20188 |
| rs2058527 | G | T | 0.730592 | 0.0104521 | 0.00171656 | 1.10E-09 | 0.086812906 | 37.07568093 | Arm fat percentage (left) | ukb-b-20188 |
| rs2111281 | C | A | 0.366931 | 0.0100307 | 0.00158027 | 2.20E-10 | 0.093635038 | 40.29024352 | Arm fat percentage (left) | ukb-b-20188 |
| rs215498 | T | G | 0.324133 | 0.00955001 | 0.00163597 | 5.30E-09 | 0.080354946 | 34.07665692 | Arm fat percentage (left) | ukb-b-20188 |
| rs215638 | T | G | 0.62754 | -0.010985 | 0.0015811 | 3.70E-12 | 0.110138522 | 48.27046085 | Arm fat percentage (left) | ukb-b-20188 |
| rs2172131 | C | T | 0.578562 | -0.0111735 | 0.0015428 | 4.40E-13 | 0.118547768 | 52.45165624 | Arm fat percentage (left) | ukb-b-20188 |
| rs217672 | C | A | 0.271726 | 0.0123193 | 0.00171679 | 7.20E-13 | 0.116631299 | 51.4917572 | Arm fat percentage (left) | ukb-b-20188 |
| rs2182634 | G | C | 0.164045 | 0.0115812 | 0.00207319 | 2.30E-08 | 0.074085812 | 31.20533955 | Arm fat percentage (left) | ukb-b-20188 |
| rs2192527 | G | A | 0.465482 | 0.0135324 | 0.00152581 | 7.40E-19 | 0.167838552 | 78.65905765 | Arm fat percentage (left) | ukb-b-20188 |
| rs2237025 | C | T | 0.555397 | -0.00877923 | 0.00154246 | 1.30E-08 | 0.076694765 | 32.39552578 | Arm fat percentage (left) | ukb-b-20188 |
| rs2242189 | C | T | 0.381124 | -0.00980095 | 0.00157852 | 5.30E-10 | 0.08995672 | 38.5510463 | Arm fat percentage (left) | ukb-b-20188 |
| rs2281819 | A | T | 0.229879 | -0.0123712 | 0.00181019 | 8.20E-12 | 0.106951256 | 46.70628588 | Arm fat percentage (left) | ukb-b-20188 |
| rs2289687 | G | C | 0.442894 | -0.00937982 | 0.00161519 | 6.30E-09 | 0.079589998 | 33.72420917 | Arm fat percentage (left) | ukb-b-20188 |
| rs2307111 | C | T | 0.395092 | -0.0172199 | 0.00155662 | 1.90E-28 | 0.238840136 | 122.3759392 | Arm fat percentage (left) | ukb-b-20188 |
| rs2356505 | G | A | 0.650288 | 0.00918715 | 0.0016005 | 9.50E-09 | 0.077904336 | 32.9496084 | Arm fat percentage (left) | ukb-b-20188 |
| rs2370982 | T | C | 0.214158 | 0.0193387 | 0.00187056 | 4.70E-25 | 0.215108 | 106.8836475 | Arm fat percentage (left) | ukb-b-20188 |
| rs2455818 | A | G | 0.597707 | -0.00917109 | 0.00154739 | 3.10E-09 | 0.082627297 | 35.12710339 | Arm fat percentage (left) | ukb-b-20188 |
| rs2466103 | G | T | 0.303562 | 0.0104286 | 0.00165403 | 2.90E-10 | 0.092501073 | 39.75257405 | Arm fat percentage (left) | ukb-b-20188 |
| rs2499468 | A | C | 0.650994 | 0.0113931 | 0.00159714 | 9.80E-13 | 0.115417481 | 50.88594514 | Arm fat percentage (left) | ukb-b-20188 |
| rs252748 | T | C | 0.590945 | -0.00875651 | 0.00155477 | 1.80E-08 | 0.075215241 | 31.71975263 | Arm fat percentage (left) | ukb-b-20188 |
| rs2569881 | A | G | 0.128664 | -0.0137196 | 0.00228697 | 0.000000002 | 0.08448201 | 35.98835234 | Arm fat percentage (left) | ukb-b-20188 |
| rs2610245 | G | A | 0.517873 | -0.00911323 | 0.00154772 | 3.90E-09 | 0.081640903 | 34.67048148 | Arm fat percentage (left) | ukb-b-20188 |
| rs262956 | G | T | 0.646875 | -0.0102704 | 0.00159469 | 1.20E-10 | 0.09613092 | 41.47841715 | Arm fat percentage (left) | ukb-b-20188 |
| rs2678204 | G | T | 0.340185 | 0.0142488 | 0.00160307 | 6.20E-19 | 0.168451404 | 79.00445977 | Arm fat percentage (left) | ukb-b-20188 |
| rs2692741 | C | G | 0.367181 | 0.00884898 | 0.00158984 | 2.60E-08 | 0.073589908 | 30.97986979 | Arm fat percentage (left) | ukb-b-20188 |
| rs2731238 | T | G | 0.067521 | -0.0206349 | 0.00332751 | 5.60E-10 | 0.089755196 | 38.4561673 | Arm fat percentage (left) | ukb-b-20188 |
| rs2731238 | T | G | 0.067521 | -0.0206349 | 0.00332751 | 5.60E-10 | 0.089755196 | 38.4561673 | Arm fat percentage (left) | ukb-b-20188 |
| rs2737263 | T | G | 0.280559 | -0.017384 | 0.00169518 | 1.10E-24 | 0.212382426 | 105.1641672 | Arm fat percentage (left) | ukb-b-20188 |
| rs2765041 | A | T | 0.710451 | -0.0114205 | 0.00167903 | 1.00E-11 | 0.106048095 | 46.26508074 | Arm fat percentage (left) | ukb-b-20188 |
| rs2798297 | A | G | 0.366705 | 0.00942015 | 0.00159011 | 3.10E-09 | 0.082560816 | 35.09629718 | Arm fat percentage (left) | ukb-b-20188 |
| rs2820446 | G | C | 0.297287 | 0.0182523 | 0.00165904 | 3.70E-28 | 0.236847251 | 121.0379285 | Arm fat percentage (left) | ukb-b-20188 |
| rs28734088 | T | C | 0.125778 | 0.0149805 | 0.00230568 | 8.20E-11 | 0.097668814 | 42.21381019 | Arm fat percentage (left) | ukb-b-20188 |
| rs2930575 | C | T | 0.864958 | 0.0122762 | 0.00223054 | 3.70E-08 | 0.072070611 | 30.2906002 | Arm fat percentage (left) | ukb-b-20188 |
| rs2954021 | G | A | 0.504589 | 0.0117081 | 0.0015222 | 1.50E-14 | 0.131712858 | 59.16016969 | Arm fat percentage (left) | ukb-b-20188 |
| rs2962334 | T | G | 0.020069 | 0.0325927 | 0.00541564 | 1.80E-09 | 0.084978273 | 36.21938767 | Arm fat percentage (left) | ukb-b-20188 |
| rs2980237 | T | G | 0.751224 | 0.0110547 | 0.00176509 | 3.80E-10 | 0.091385113 | 39.22475265 | Arm fat percentage (left) | ukb-b-20188 |
| rs3087523 | A | G | 0.124619 | 0.0135562 | 0.00231182 | 4.50E-09 | 0.081022934 | 34.38491079 | Arm fat percentage (left) | ukb-b-20188 |
| rs3113509 | T | C | 0.731977 | -0.00993318 | 0.00171804 | 7.40E-09 | 0.078946071 | 33.42797501 | Arm fat percentage (left) | ukb-b-20188 |
| rs3114914 | T | C | 0.743244 | -0.00981322 | 0.00176315 | 2.60E-08 | 0.073584407 | 30.97737032 | Arm fat percentage (left) | ukb-b-20188 |
| rs319788 | T | C | 0.696263 | 0.00934408 | 0.00165488 | 0.000000016 | 0.075570013 | 31.881598 | Arm fat percentage (left) | ukb-b-20188 |
| rs34045894 | A | G | 0.156083 | 0.0123589 | 0.00209787 | 3.80E-09 | 0.081717348 | 34.7058345 | Arm fat percentage (left) | ukb-b-20188 |
| rs34234296 | A | G | 0.392489 | -0.0102543 | 0.00157136 | 6.80E-11 | 0.098443898 | 42.58539213 | Arm fat percentage (left) | ukb-b-20188 |
| rs34292685 | T | C | 0.161833 | -0.0143896 | 0.0020638 | 3.10E-12 | 0.110835693 | 48.61409743 | Arm fat percentage (left) | ukb-b-20188 |
| rs34331990 | G | T | 0.418756 | 0.0105643 | 0.00155302 | 1.00E-11 | 0.106064256 | 46.27296772 | Arm fat percentage (left) | ukb-b-20188 |
| rs34338229 | A | G | 0.332002 | 0.00967977 | 0.00162097 | 2.30E-09 | 0.083775568 | 35.65990008 | Arm fat percentage (left) | ukb-b-20188 |
| rs34483452 | A | C | 0.136308 | 0.0249615 | 0.00223514 | 5.90E-29 | 0.242304719 | 124.7187926 | Arm fat percentage (left) | ukb-b-20188 |
| rs34580448 | C | T | 0.041254 | -0.0234802 | 0.0038313 | 8.90E-10 | 0.087844678 | 37.55876163 | Arm fat percentage (left) | ukb-b-20188 |
| rs34606703 | A | G | 0.357996 | -0.0134022 | 0.00158728 | 3.10E-17 | 0.154549823 | 71.29270629 | Arm fat percentage (left) | ukb-b-20188 |
| rs34796300 | C | T | 0.570551 | 0.0121294 | 0.00153751 | 3.00E-15 | 0.137618703 | 62.23615281 | Arm fat percentage (left) | ukb-b-20188 |
| rs34882821 | T | G | 0.338504 | 0.00934946 | 0.00161338 | 6.80E-09 | 0.07927984 | 33.58147114 | Arm fat percentage (left) | ukb-b-20188 |
| rs34898535 | T | C | 0.377909 | -0.0157119 | 0.00157078 | 1.50E-23 | 0.20416643 | 100.0522102 | Arm fat percentage (left) | ukb-b-20188 |
| rs34994596 | C | T | 0.297375 | -0.0107346 | 0.00166658 | 1.20E-10 | 0.096150374 | 41.48770399 | Arm fat percentage (left) | ukb-b-20188 |
| rs35023999 | C | A | 0.508301 | -0.00964293 | 0.00152299 | 2.40E-10 | 0.093210687 | 40.08888015 | Arm fat percentage (left) | ukb-b-20188 |
| rs35949039 | T | G | 0.105572 | -0.0153871 | 0.0024967 | 7.10E-10 | 0.088747282 | 37.98226242 | Arm fat percentage (left) | ukb-b-20188 |
| rs36090025 | C | A | 0.299697 | -0.00962955 | 0.00166199 | 6.90E-09 | 0.079255548 | 33.57029613 | Arm fat percentage (left) | ukb-b-20188 |
| rs3751859 | A | G | 0.156472 | -0.0132885 | 0.00209485 | 2.20E-10 | 0.093526867 | 40.23889622 | Arm fat percentage (left) | ukb-b-20188 |
| rs3764002 | T | C | 0.261503 | -0.0145944 | 0.00173119 | 3.40E-17 | 0.154140464 | 71.06946083 | Arm fat percentage (left) | ukb-b-20188 |
| rs3766823 | A | G | 0.172019 | 0.0130137 | 0.00201287 | 1.00E-10 | 0.096802837 | 41.79940766 | Arm fat percentage (left) | ukb-b-20188 |
| rs3770821 | C | T | 0.332691 | 0.0113299 | 0.00162042 | 2.70E-12 | 0.111389396 | 48.88740261 | Arm fat percentage (left) | ukb-b-20188 |
| rs3774063 | T | C | 0.102123 | 0.0156864 | 0.00250923 | 4.10E-10 | 0.091080697 | 39.08099641 | Arm fat percentage (left) | ukb-b-20188 |
| rs3803286 | G | A | 0.666751 | -0.0112441 | 0.00161536 | 3.40E-12 | 0.110506748 | 48.45189302 | Arm fat percentage (left) | ukb-b-20188 |
| rs3826408 | T | C | 0.456867 | 0.00956246 | 0.00152841 | 3.90E-10 | 0.091213054 | 39.14348798 | Arm fat percentage (left) | ukb-b-20188 |
| rs3901286 | A | C | 0.152426 | -0.0156077 | 0.00212196 | 1.90E-13 | 0.121820929 | 54.10076816 | Arm fat percentage (left) | ukb-b-20188 |
| rs3911063 | C | T | 0.321991 | -0.0110089 | 0.00163005 | 1.40E-11 | 0.104709346 | 45.61272318 | Arm fat percentage (left) | ukb-b-20188 |
| rs394608 | C | T | 0.537727 | 0.0129019 | 0.00153496 | 4.30E-17 | 0.153370552 | 70.65017107 | Arm fat percentage (left) | ukb-b-20188 |
| rs40071 | C | T | 0.179496 | -0.0148201 | 0.0019876 | 8.90E-14 | 0.124767914 | 55.59609547 | Arm fat percentage (left) | ukb-b-20188 |
| rs4055791 | T | C | 0.416764 | -0.0110359 | 0.00154597 | 9.40E-13 | 0.115562265 | 50.95811904 | Arm fat percentage (left) | ukb-b-20188 |
| rs4234589 | G | A | 0.13719 | -0.0182348 | 0.00220923 | 1.50E-16 | 0.148707929 | 68.12713797 | Arm fat percentage (left) | ukb-b-20188 |
| rs42523 | C | T | 0.245211 | 0.00969216 | 0.00177201 | 4.50E-08 | 0.071243626 | 29.9163643 | Arm fat percentage (left) | ukb-b-20188 |
| rs429343 | G | A | 0.576554 | -0.0118182 | 0.00153857 | 1.60E-14 | 0.13140736 | 59.00219255 | Arm fat percentage (left) | ukb-b-20188 |
| rs429358 | C | T | 0.154041 | -0.0188481 | 0.00211229 | 4.50E-19 | 0.169543243 | 79.62108081 | Arm fat percentage (left) | ukb-b-20188 |
| rs4307239 | G | A | 0.458849 | 0.00873582 | 0.00153065 | 1.10E-08 | 0.07708218 | 32.57283521 | Arm fat percentage (left) | ukb-b-20188 |
| rs4311684 | C | T | 0.706694 | 0.0107777 | 0.0016729 | 1.20E-10 | 0.096188965 | 41.50612782 | Arm fat percentage (left) | ukb-b-20188 |
| rs4398538 | C | T | 0.642501 | -0.0110904 | 0.00159027 | 3.10E-12 | 0.11087892 | 48.63542195 | Arm fat percentage (left) | ukb-b-20188 |
| rs4430672 | C | T | 0.800935 | -0.0106017 | 0.00191122 | 2.90E-08 | 0.073128175 | 30.77015319 | Arm fat percentage (left) | ukb-b-20188 |
| rs445077 | A | T | 0.480134 | 0.0122791 | 0.00152645 | 8.70E-16 | 0.142309502 | 64.70947942 | Arm fat percentage (left) | ukb-b-20188 |
| rs4466418 | A | G | 0.56225 | 0.00874963 | 0.00153661 | 0.000000012 | 0.076754632 | 32.42291562 | Arm fat percentage (left) | ukb-b-20188 |
| rs4482463 | A | C | 0.923059 | -0.0222646 | 0.00285623 | 6.40E-15 | 0.134801464 | 60.76359234 | Arm fat percentage (left) | ukb-b-20188 |
| rs4549685 | T | C | 0.329796 | -0.01277 | 0.00161962 | 3.20E-15 | 0.137485618 | 62.16637337 | Arm fat percentage (left) | ukb-b-20188 |
| rs4562625 | G | C | 0.614934 | -0.0100671 | 0.00155989 | 1.10E-10 | 0.096491379 | 41.65055735 | Arm fat percentage (left) | ukb-b-20188 |
| rs4605363 | C | A | 0.341588 | 0.0119031 | 0.00160001 | 1.00E-13 | 0.124273531 | 55.34453851 | Arm fat percentage (left) | ukb-b-20188 |
| rs4673617 | T | C | 0.271779 | 0.0108941 | 0.00171159 | 2.00E-10 | 0.094101818 | 40.51195803 | Arm fat percentage (left) | ukb-b-20188 |
| rs4722398 | T | C | 0.13611 | 0.0144685 | 0.0022144 | 6.40E-11 | 0.098663644 | 42.69085686 | Arm fat percentage (left) | ukb-b-20188 |
| rs4737188 | T | A | 0.473839 | -0.00850912 | 0.00152623 | 0.000000025 | 0.073817808 | 31.0834578 | Arm fat percentage (left) | ukb-b-20188 |
| rs4762951 | G | A | 0.780431 | -0.0111126 | 0.00183946 | 1.50E-09 | 0.085572702 | 36.49645377 | Arm fat percentage (left) | ukb-b-20188 |
| rs4771121 | A | G | 0.275111 | -0.0107506 | 0.00171452 | 3.60E-10 | 0.091580333 | 39.31699302 | Arm fat percentage (left) | ukb-b-20188 |
| rs4776985 | G | T | 0.224995 | -0.0215162 | 0.00182121 | 3.30E-32 | 0.263561947 | 139.5761107 | Arm fat percentage (left) | ukb-b-20188 |
| rs4790841 | T | C | 0.154513 | -0.0202147 | 0.00211665 | 1.30E-21 | 0.18954092 | 91.20874901 | Arm fat percentage (left) | ukb-b-20188 |
| rs4793085 | C | A | 0.656341 | -0.0126911 | 0.00161092 | 3.30E-15 | 0.137293247 | 62.06554674 | Arm fat percentage (left) | ukb-b-20188 |
| rs4820323 | G | C | 0.580953 | -0.0137802 | 0.00154866 | 5.70E-19 | 0.168757154 | 79.17697024 | Arm fat percentage (left) | ukb-b-20188 |
| rs4837119 | A | T | 0.517251 | -0.0124247 | 0.00152727 | 4.10E-16 | 0.145078145 | 66.18204469 | Arm fat percentage (left) | ukb-b-20188 |
| rs4850788 | A | G | 0.384643 | 0.00878627 | 0.00156125 | 1.80E-08 | 0.075108705 | 31.67117581 | Arm fat percentage (left) | ukb-b-20188 |
| rs4887872 | C | T | 0.19458 | 0.0110497 | 0.00192971 | 0.00000001 | 0.077552192 | 32.78814744 | Arm fat percentage (left) | ukb-b-20188 |
| rs4930387 | G | A | 0.531141 | 0.00952105 | 0.00152602 | 4.40E-10 | 0.090754049 | 38.92684813 | Arm fat percentage (left) | ukb-b-20188 |
| rs4933220 | A | G | 0.425122 | -0.00865272 | 0.00154016 | 1.90E-08 | 0.074870629 | 31.56266171 | Arm fat percentage (left) | ukb-b-20188 |
| rs4947124 | A | G | 0.709595 | 0.0102471 | 0.00168016 | 1.10E-09 | 0.087070914 | 37.19637921 | Arm fat percentage (left) | ukb-b-20188 |
| rs522110 | G | A | 0.555026 | 0.0141373 | 0.00153256 | 2.80E-20 | 0.179109492 | 85.09381124 | Arm fat percentage (left) | ukb-b-20188 |
| rs543874 | G | A | 0.205275 | 0.0329544 | 0.00187993 | 8.50E-69 | 0.440689002 | 307.2864859 | Arm fat percentage (left) | ukb-b-20188 |
| rs55707359 | G | T | 0.015445 | 0.0367288 | 0.00625173 | 4.20E-09 | 0.081305437 | 34.51541121 | Arm fat percentage (left) | ukb-b-20188 |
| rs55714539 | C | A | 0.343601 | 0.0157155 | 0.00161661 | 2.40E-22 | 0.195051528 | 94.50306282 | Arm fat percentage (left) | ukb-b-20188 |
| rs55726687 | A | G | 0.209739 | 0.0143113 | 0.0018674 | 1.80E-14 | 0.13088663 | 58.73317282 | Arm fat percentage (left) | ukb-b-20188 |
| rs55924785 | T | C | 0.164683 | -0.012418 | 0.0020526 | 1.40E-09 | 0.085797107 | 36.60114417 | Arm fat percentage (left) | ukb-b-20188 |
| rs56218501 | T | C | 0.211817 | -0.0114937 | 0.00186378 | 7.00E-10 | 0.088849676 | 38.0303586 | Arm fat percentage (left) | ukb-b-20188 |
| rs56226325 | T | C | 0.155789 | -0.0150493 | 0.00209707 | 7.20E-13 | 0.116647677 | 51.49994255 | Arm fat percentage (left) | ukb-b-20188 |
| rs56356382 | C | T | 0.192335 | -0.0169593 | 0.0019412 | 2.40E-18 | 0.163676072 | 76.32648806 | Arm fat percentage (left) | ukb-b-20188 |
| rs56369689 | G | A | 0.34861 | -0.012077 | 0.00163448 | 1.50E-13 | 0.122798651 | 54.59575938 | Arm fat percentage (left) | ukb-b-20188 |
| rs56399737 | T | C | 0.449166 | -0.012477 | 0.00153671 | 4.70E-16 | 0.144592271 | 65.92293207 | Arm fat percentage (left) | ukb-b-20188 |
| rs56803094 | G | A | 0.226679 | -0.010754 | 0.00182616 | 3.90E-09 | 0.081658607 | 34.67866837 | Arm fat percentage (left) | ukb-b-20188 |
| rs56858768 | A | G | 0.296903 | 0.0121639 | 0.00167325 | 3.60E-13 | 0.119335558 | 52.84744737 | Arm fat percentage (left) | ukb-b-20188 |
| rs57636386 | C | T | 0.083839 | -0.0278282 | 0.00275778 | 6.10E-24 | 0.207033729 | 101.8241976 | Arm fat percentage (left) | ukb-b-20188 |
| rs57800857 | C | A | 0.364899 | -0.0132501 | 0.00158877 | 7.40E-17 | 0.151349359 | 69.55306144 | Arm fat percentage (left) | ukb-b-20188 |
| rs58862095 | T | C | 0.419267 | -0.0148969 | 0.00154612 | 5.70E-22 | 0.192268387 | 92.83364627 | Arm fat percentage (left) | ukb-b-20188 |
| rs59086897 | A | T | 0.487597 | 0.026721 | 0.0015173 | 2.00E-69 | 0.442971229 | 310.1433677 | Arm fat percentage (left) | ukb-b-20188 |
| rs6021948 | A | T | 0.321773 | -0.0120032 | 0.00163467 | 2.10E-13 | 0.1214594 | 53.91801562 | Arm fat percentage (left) | ukb-b-20188 |
| rs6023655 | G | A | 0.765801 | -0.0102372 | 0.00180862 | 0.000000015 | 0.07591297 | 32.03817121 | Arm fat percentage (left) | ukb-b-20188 |
| rs6044084 | G | A | 0.586907 | 0.00879161 | 0.00155138 | 0.000000015 | 0.076079953 | 32.1144473 | Arm fat percentage (left) | ukb-b-20188 |
| rs6054427 | A | G | 0.620812 | 0.0137177 | 0.00157689 | 3.30E-18 | 0.16250835 | 75.67628489 | Arm fat percentage (left) | ukb-b-20188 |
| rs6103254 | C | T | 0.126698 | -0.0142295 | 0.00229909 | 6.00E-10 | 0.089436157 | 38.30604681 | Arm fat percentage (left) | ukb-b-20188 |
| rs6138536 | G | A | 0.462452 | 0.0110794 | 0.00152903 | 4.30E-13 | 0.11865398 | 52.50497682 | Arm fat percentage (left) | ukb-b-20188 |
| rs61813324 | T | C | 0.135713 | 0.0195695 | 0.00224951 | 3.30E-18 | 0.162515809 | 75.68043209 | Arm fat percentage (left) | ukb-b-20188 |
| rs61871615 | T | C | 0.091506 | -0.0204532 | 0.00276646 | 1.40E-13 | 0.122926402 | 54.6605176 | Arm fat percentage (left) | ukb-b-20188 |
| rs61903695 | G | A | 0.2549 | 0.0120354 | 0.00174877 | 5.90E-12 | 0.108295858 | 47.36479574 | Arm fat percentage (left) | ukb-b-20188 |
| rs62106258 | C | T | 0.048535 | -0.0566363 | 0.00353006 | 6.30E-58 | 0.397599754 | 257.4100938 | Arm fat percentage (left) | ukb-b-20188 |
| rs62147189 | G | T | 0.622281 | -0.00973146 | 0.00158712 | 8.70E-10 | 0.087923161 | 37.59555279 | Arm fat percentage (left) | ukb-b-20188 |
| rs62190394 | T | C | 0.317039 | 0.0140103 | 0.00163241 | 9.30E-18 | 0.158867845 | 73.66079073 | Arm fat percentage (left) | ukb-b-20188 |
| rs62218301 | G | A | 0.166065 | -0.0116896 | 0.00206017 | 1.40E-08 | 0.076257003 | 32.19535244 | Arm fat percentage (left) | ukb-b-20188 |
| rs62413414 | T | C | 0.151722 | 0.0121845 | 0.00211683 | 8.60E-09 | 0.07830111 | 33.13168022 | Arm fat percentage (left) | ukb-b-20188 |
| rs6491427 | G | A | 0.288993 | -0.0126651 | 0.00167989 | 4.70E-14 | 0.127204749 | 56.84019468 | Arm fat percentage (left) | ukb-b-20188 |
| rs6497451 | G | T | 0.129666 | -0.015741 | 0.00230355 | 8.30E-12 | 0.10692795 | 46.69488941 | Arm fat percentage (left) | ukb-b-20188 |
| rs6545714 | A | G | 0.601479 | -0.0155934 | 0.00155274 | 9.90E-24 | 0.205463019 | 100.8519168 | Arm fat percentage (left) | ukb-b-20188 |
| rs654718 | C | T | 0.32786 | -0.0100495 | 0.00162231 | 5.80E-10 | 0.089577623 | 38.3725993 | Arm fat percentage (left) | ukb-b-20188 |
| rs6567160 | C | T | 0.23266 | 0.0295724 | 0.00180298 | 1.90E-60 | 0.408215952 | 269.0241856 | Arm fat percentage (left) | ukb-b-20188 |
| rs6575340 | A | G | 0.636028 | 0.013974 | 0.00158722 | 1.30E-18 | 0.165796266 | 77.5116931 | Arm fat percentage (left) | ukb-b-20188 |
| rs6597975 | G | C | 0.543875 | 0.00852924 | 0.00153597 | 2.80E-08 | 0.073272756 | 30.8357988 | Arm fat percentage (left) | ukb-b-20188 |
| rs6669341 | G | A | 0.582716 | -0.0130045 | 0.00153958 | 3.00E-17 | 0.154651452 | 71.34816312 | Arm fat percentage (left) | ukb-b-20188 |
| rs6693294 | G | A | 0.688578 | -0.0124436 | 0.00164018 | 3.30E-14 | 0.128605429 | 57.55844608 | Arm fat percentage (left) | ukb-b-20188 |
| rs6699744 | T | A | 0.61605 | 0.0144061 | 0.0015682 | 4.10E-20 | 0.177891259 | 84.38979875 | Arm fat percentage (left) | ukb-b-20188 |
| rs6699744 | T | A | 0.61605 | 0.0144061 | 0.0015682 | 4.10E-20 | 0.177891259 | 84.38979875 | Arm fat percentage (left) | ukb-b-20188 |
| rs6705567 | C | T | 0.375911 | -0.0105511 | 0.00157834 | 2.30E-11 | 0.1028055 | 44.68835335 | Arm fat percentage (left) | ukb-b-20188 |
| rs6711390 | T | C | 0.371296 | -0.0102463 | 0.00156183 | 5.40E-11 | 0.099389192 | 43.03944016 | Arm fat percentage (left) | ukb-b-20188 |
| rs6736194 | G | C | 0.58102 | 0.00952323 | 0.00154402 | 6.90E-10 | 0.088874337 | 38.04194414 | Arm fat percentage (left) | ukb-b-20188 |
| rs67373773 | A | G | 0.439101 | -0.00919865 | 0.00153054 | 1.90E-09 | 0.084766834 | 36.12092147 | Arm fat percentage (left) | ukb-b-20188 |
| rs67609008 | C | T | 0.283591 | 0.0116886 | 0.00169538 | 5.40E-12 | 0.108637694 | 47.53252467 | Arm fat percentage (left) | ukb-b-20188 |
| rs67807996 | A | G | 0.395644 | 0.0156864 | 0.00160191 | 1.20E-22 | 0.19734811 | 95.88934355 | Arm fat percentage (left) | ukb-b-20188 |
| rs6782581 | G | C | 0.439286 | -0.00949242 | 0.0015315 | 5.70E-10 | 0.08967131 | 38.41668522 | Arm fat percentage (left) | ukb-b-20188 |
| rs6840236 | C | T | 0.464873 | 0.00962404 | 0.00152613 | 2.90E-10 | 0.092533265 | 39.76781955 | Arm fat percentage (left) | ukb-b-20188 |
| rs6847975 | A | G | 0.356166 | 0.0110402 | 0.00159462 | 4.40E-12 | 0.109453906 | 47.93353609 | Arm fat percentage (left) | ukb-b-20188 |
| rs6869862 | G | T | 0.480449 | 0.00903123 | 0.0015223 | 3.00E-09 | 0.082775944 | 35.19600013 | Arm fat percentage (left) | ukb-b-20188 |
| rs687621 | G | A | 0.320097 | 0.01142 | 0.00163219 | 2.60E-12 | 0.111524807 | 48.95429295 | Arm fat percentage (left) | ukb-b-20188 |
| rs6903068 | T | G | 0.262802 | -0.0102529 | 0.00172474 | 2.80E-09 | 0.083082898 | 35.3383422 | Arm fat percentage (left) | ukb-b-20188 |
| rs6927268 | G | T | 0.206318 | -0.012669 | 0.00188033 | 1.60E-11 | 0.104263476 | 45.39588865 | Arm fat percentage (left) | ukb-b-20188 |
| rs6963767 | A | G | 0.800046 | 0.0125667 | 0.00190312 | 4.00E-11 | 0.100558433 | 43.60237526 | Arm fat percentage (left) | ukb-b-20188 |
| rs6977416 | A | G | 0.33425 | -0.00896428 | 0.00162734 | 3.60E-08 | 0.072188709 | 30.34409767 | Arm fat percentage (left) | ukb-b-20188 |
| rs7027304 | T | C | 0.652641 | 0.0121845 | 0.00160627 | 3.30E-14 | 0.128571699 | 57.54112229 | Arm fat percentage (left) | ukb-b-20188 |
| rs7034554 | G | A | 0.373725 | -0.00965599 | 0.00157198 | 8.10E-10 | 0.088212218 | 37.73111003 | Arm fat percentage (left) | ukb-b-20188 |
| rs704061 | C | T | 0.455052 | 0.0132493 | 0.00152829 | 4.30E-18 | 0.161574929 | 75.15784598 | Arm fat percentage (left) | ukb-b-20188 |
| rs705158 | A | T | 0.244694 | 0.0108909 | 0.00177425 | 8.30E-10 | 0.088100853 | 37.67887375 | Arm fat percentage (left) | ukb-b-20188 |
| rs7070670 | T | C | 0.327988 | -0.00913103 | 0.00163151 | 2.20E-08 | 0.074343865 | 31.32276262 | Arm fat percentage (left) | ukb-b-20188 |
| rs7124681 | A | C | 0.408395 | 0.0244832 | 0.00154469 | 1.40E-56 | 0.391784125 | 251.2196988 | Arm fat percentage (left) | ukb-b-20188 |
| rs7133378 | A | G | 0.319424 | 0.0181924 | 0.00163517 | 9.40E-29 | 0.240921793 | 123.7810526 | Arm fat percentage (left) | ukb-b-20188 |
| rs71658797 | A | T | 0.120738 | 0.0238357 | 0.00233573 | 1.90E-24 | 0.210747298 | 104.1383144 | Arm fat percentage (left) | ukb-b-20188 |
| rs7171864 | A | G | 0.660085 | 0.0130459 | 0.00161599 | 6.90E-16 | 0.14318378 | 65.17345619 | Arm fat percentage (left) | ukb-b-20188 |
| rs7187776 | G | A | 0.401997 | 0.0243354 | 0.00155158 | 1.90E-55 | 0.386788917 | 245.9963326 | Arm fat percentage (left) | ukb-b-20188 |
| rs7201895 | A | G | 0.354221 | -0.0107282 | 0.00160169 | 2.10E-11 | 0.103167632 | 44.86387627 | Arm fat percentage (left) | ukb-b-20188 |
| rs7218014 | C | T | 0.197305 | 0.0180363 | 0.00191806 | 5.30E-21 | 0.184823631 | 88.42407468 | Arm fat percentage (left) | ukb-b-20188 |
| rs723672 | T | C | 0.431522 | 0.00997263 | 0.00154476 | 1.10E-10 | 0.096546911 | 41.67708945 | Arm fat percentage (left) | ukb-b-20188 |
| rs7246182 | C | T | 0.222966 | -0.0110787 | 0.00183618 | 1.60E-09 | 0.085374073 | 36.40383181 | Arm fat percentage (left) | ukb-b-20188 |
| rs725959 | T | G | 0.412477 | -0.00886742 | 0.00155347 | 1.10E-08 | 0.077104011 | 32.58283106 | Arm fat percentage (left) | ukb-b-20188 |
| rs72744924 | G | A | 0.089261 | 0.0156893 | 0.00269488 | 5.80E-09 | 0.079959592 | 33.89442511 | Arm fat percentage (left) | ukb-b-20188 |
| rs72892910 | T | G | 0.172213 | 0.0249715 | 0.00201732 | 3.40E-35 | 0.282070125 | 153.2285432 | Arm fat percentage (left) | ukb-b-20188 |
| rs7321331 | A | G | 0.742308 | 0.00990806 | 0.00175075 | 0.000000015 | 0.075890564 | 32.02793848 | Arm fat percentage (left) | ukb-b-20188 |
| rs73213501 | C | A | 0.171849 | -0.0150625 | 0.00201656 | 8.10E-14 | 0.125152511 | 55.79198626 | Arm fat percentage (left) | ukb-b-20188 |
| rs73216116 | A | G | 0.318588 | 0.00918086 | 0.00165869 | 3.10E-08 | 0.072833223 | 30.63629741 | Arm fat percentage (left) | ukb-b-20188 |
| rs7341329 | G | A | 0.328076 | 0.0186284 | 0.00161795 | 1.10E-30 | 0.25367789 | 132.5625705 | Arm fat percentage (left) | ukb-b-20188 |
| rs7357754 | G | A | 0.500121 | 0.0113996 | 0.00152682 | 8.30E-14 | 0.125059694 | 55.74469517 | Arm fat percentage (left) | ukb-b-20188 |
| rs7422252 | T | C | 0.591448 | 0.0125544 | 0.00154888 | 5.30E-16 | 0.144171244 | 65.69864001 | Arm fat percentage (left) | ukb-b-20188 |
| rs7442885 | G | C | 0.21412 | -0.0122915 | 0.00185695 | 3.60E-11 | 0.100996459 | 43.81364153 | Arm fat percentage (left) | ukb-b-20188 |
| rs7463186 | G | A | 0.516028 | 0.00842667 | 0.00152234 | 3.10E-08 | 0.072841368 | 30.63999258 | Arm fat percentage (left) | ukb-b-20188 |
| rs7498044 | A | G | 0.217388 | -0.0123922 | 0.00186602 | 3.10E-11 | 0.101594886 | 44.10260469 | Arm fat percentage (left) | ukb-b-20188 |
| rs750090 | C | T | 0.356722 | -0.00912285 | 0.00160466 | 1.30E-08 | 0.076533496 | 32.32176099 | Arm fat percentage (left) | ukb-b-20188 |
| rs7519259 | A | G | 0.52834 | 0.00969498 | 0.00152807 | 2.20E-10 | 0.093558355 | 40.25384185 | Arm fat percentage (left) | ukb-b-20188 |
| rs75412871 | T | C | 0.051929 | -0.0213344 | 0.00343397 | 5.20E-10 | 0.090056996 | 38.59827299 | Arm fat percentage (left) | ukb-b-20188 |
| rs75499503 | T | C | 0.22013 | -0.0181862 | 0.00186199 | 1.60E-22 | 0.196531979 | 95.39579624 | Arm fat percentage (left) | ukb-b-20188 |
| rs7612882 | A | G | 0.546453 | 0.00836752 | 0.00152972 | 4.50E-08 | 0.071252892 | 29.92055386 | Arm fat percentage (left) | ukb-b-20188 |
| rs7628689 | G | A | 0.843359 | 0.0114129 | 0.00208862 | 4.60E-08 | 0.071116413 | 29.85885565 | Arm fat percentage (left) | ukb-b-20188 |
| rs7630228 | C | T | 0.434302 | -0.0104019 | 0.00154177 | 1.50E-11 | 0.104515269 | 45.51831395 | Arm fat percentage (left) | ukb-b-20188 |
| rs764729 | C | A | 0.72802 | 0.010159 | 0.00170873 | 2.80E-09 | 0.083101995 | 35.3472012 | Arm fat percentage (left) | ukb-b-20188 |
| rs7656367 | G | A | 0.488528 | 0.00896065 | 0.00152302 | 0.000000004 | 0.081521507 | 34.61527735 | Arm fat percentage (left) | ukb-b-20188 |
| rs7663885 | C | T | 0.500243 | -0.00922852 | 0.00151846 | 1.20E-09 | 0.08651551 | 36.93664144 | Arm fat percentage (left) | ukb-b-20188 |
| rs76702514 | G | C | 0.210645 | -0.0121318 | 0.00187363 | 9.50E-11 | 0.09706743 | 41.92594121 | Arm fat percentage (left) | ukb-b-20188 |
| rs7678322 | C | T | 0.329085 | 0.00962082 | 0.00162172 | 3.00E-09 | 0.082772294 | 35.19430823 | Arm fat percentage (left) | ukb-b-20188 |
| rs7704382 | G | C | 0.433421 | 0.0105454 | 0.00153677 | 6.80E-12 | 0.107730751 | 47.08779648 | Arm fat percentage (left) | ukb-b-20188 |
| rs7707394 | A | G | 0.357282 | -0.0119231 | 0.00158482 | 5.30E-14 | 0.126735857 | 56.60026785 | Arm fat percentage (left) | ukb-b-20188 |
| rs7730898 | A | G | 0.729117 | 0.0146563 | 0.00171141 | 1.10E-17 | 0.158285387 | 73.33994187 | Arm fat percentage (left) | ukb-b-20188 |
| rs7755574 | T | G | 0.282689 | 0.0109694 | 0.00169006 | 8.60E-11 | 0.09748774 | 42.12709388 | Arm fat percentage (left) | ukb-b-20188 |
| rs7761673 | A | T | 0.219979 | -0.010308 | 0.0018398 | 0.000000021 | 0.074494147 | 31.39117635 | Arm fat percentage (left) | ukb-b-20188 |
| rs7796825 | A | G | 0.825768 | 0.0116825 | 0.00201054 | 6.20E-09 | 0.079675115 | 33.76339764 | Arm fat percentage (left) | ukb-b-20188 |
| rs7824675 | A | C | 0.519413 | -0.0113917 | 0.00152919 | 9.40E-14 | 0.124569381 | 55.49504143 | Arm fat percentage (left) | ukb-b-20188 |
| rs78296744 | A | G | 0.272341 | -0.0126204 | 0.00171223 | 1.70E-13 | 0.122269619 | 54.32778964 | Arm fat percentage (left) | ukb-b-20188 |
| rs7893571 | T | G | 0.665926 | 0.00997477 | 0.0016179 | 7.00E-10 | 0.088807186 | 38.01039921 | Arm fat percentage (left) | ukb-b-20188 |
| rs79113395 | A | G | 0.26521 | -0.0141803 | 0.0017275 | 2.20E-16 | 0.147318405 | 67.38057702 | Arm fat percentage (left) | ukb-b-20188 |
| rs7950748 | T | A | 0.249492 | 0.0117377 | 0.00177323 | 3.60E-11 | 0.101002003 | 43.81631703 | Arm fat percentage (left) | ukb-b-20188 |
| rs79518326 | A | C | 0.028639 | -0.0277127 | 0.00460696 | 1.80E-09 | 0.084904482 | 36.1850184 | Arm fat percentage (left) | ukb-b-20188 |
| rs79603598 | T | A | 0.079431 | 0.0157574 | 0.00283016 | 2.60E-08 | 0.073631919 | 30.99896132 | Arm fat percentage (left) | ukb-b-20188 |
| rs79781120 | A | C | 0.113997 | -0.0139419 | 0.00239904 | 6.20E-09 | 0.079695853 | 33.77294622 | Arm fat percentage (left) | ukb-b-20188 |
| rs8008772 | T | A | 0.25047 | 0.0100257 | 0.00176563 | 1.40E-08 | 0.07636036 | 32.24259672 | Arm fat percentage (left) | ukb-b-20188 |
| rs8016513 | C | T | 0.148794 | -0.0120853 | 0.00214037 | 0.000000016 | 0.075569544 | 31.88138361 | Arm fat percentage (left) | ukb-b-20188 |
| rs801738 | G | C | 0.357526 | -0.011473 | 0.001588 | 5.00E-13 | 0.118041935 | 52.19789519 | Arm fat percentage (left) | ukb-b-20188 |
| rs80243702 | A | G | 0.16068 | 0.0132881 | 0.00208985 | 2.00E-10 | 0.09392772 | 40.42923693 | Arm fat percentage (left) | ukb-b-20188 |
| rs8031704 | A | C | 0.327605 | 0.0124192 | 0.0016258 | 2.20E-14 | 0.130147026 | 58.35163155 | Arm fat percentage (left) | ukb-b-20188 |
| rs8076669 | C | T | 0.561566 | 0.0090491 | 0.00153652 | 3.90E-09 | 0.081671027 | 34.68441228 | Arm fat percentage (left) | ukb-b-20188 |
| rs8090017 | A | G | 0.334467 | 0.0108336 | 0.00162285 | 2.50E-11 | 0.102549751 | 44.56447896 | Arm fat percentage (left) | ukb-b-20188 |
| rs8096658 | G | C | 0.487503 | -0.00849388 | 0.00154289 | 3.70E-08 | 0.072106719 | 30.30695566 | Arm fat percentage (left) | ukb-b-20188 |
| rs811054 | T | C | 0.537257 | 0.0108333 | 0.0015367 | 1.80E-12 | 0.113028604 | 49.69850874 | Arm fat percentage (left) | ukb-b-20188 |
| rs8112818 | G | A | 0.40037 | -0.0142304 | 0.00155986 | 7.30E-20 | 0.175870776 | 83.22675706 | Arm fat percentage (left) | ukb-b-20188 |
| rs811494 | G | T | 0.753303 | 0.00984383 | 0.00176215 | 2.30E-08 | 0.074087952 | 31.20631309 | Arm fat percentage (left) | ukb-b-20188 |
| rs8118253 | A | T | 0.131607 | 0.0126368 | 0.00225956 | 2.20E-08 | 0.074243531 | 31.27709924 | Arm fat percentage (left) | ukb-b-20188 |
| rs8126575 | G | T | 0.139301 | -0.0122891 | 0.00224891 | 4.60E-08 | 0.071119888 | 29.86042641 | Arm fat percentage (left) | ukb-b-20188 |
| rs8132491 | A | G | 0.312991 | -0.00986925 | 0.00168786 | 0.000000005 | 0.080600053 | 34.18971325 | Arm fat percentage (left) | ukb-b-20188 |
| rs815163 | C | T | 0.563223 | -0.0106301 | 0.00152967 | 3.70E-12 | 0.110183158 | 48.29244584 | Arm fat percentage (left) | ukb-b-20188 |
| rs8180470 | T | C | 0.534853 | -0.0107412 | 0.00153125 | 2.30E-12 | 0.112032921 | 49.20547209 | Arm fat percentage (left) | ukb-b-20188 |
| rs8192675 | C | T | 0.28866 | 0.0124029 | 0.00167476 | 1.30E-13 | 0.123291201 | 54.84554103 | Arm fat percentage (left) | ukb-b-20188 |
| rs879620 | T | C | 0.613269 | 0.0160153 | 0.00156782 | 1.70E-24 | 0.211079628 | 104.3464687 | Arm fat percentage (left) | ukb-b-20188 |
| rs890793 | T | C | 0.385663 | 0.0091834 | 0.0015652 | 4.40E-09 | 0.0811086 | 34.42447487 | Arm fat percentage (left) | ukb-b-20188 |
| rs9304665 | A | T | 0.76364 | 0.0131545 | 0.00179767 | 2.50E-13 | 0.12072296 | 53.54621147 | Arm fat percentage (left) | ukb-b-20188 |
| rs935166 | A | G | 0.506865 | -0.0119534 | 0.00151959 | 3.70E-15 | 0.136933499 | 61.87711426 | Arm fat percentage (left) | ukb-b-20188 |
| rs9372414 | T | C | 0.347613 | -0.00954503 | 0.00160084 | 2.50E-09 | 0.083542323 | 35.55156641 | Arm fat percentage (left) | ukb-b-20188 |
| rs9446091 | C | T | 0.257406 | -0.00984444 | 0.00174301 | 0.000000016 | 0.075608981 | 31.89938243 | Arm fat percentage (left) | ukb-b-20188 |
| rs946185 | G | A | 0.594807 | -0.0103912 | 0.00156055 | 2.80E-11 | 0.102081775 | 44.33799311 | Arm fat percentage (left) | ukb-b-20188 |
| rs9480184 | T | C | 0.210757 | 0.0103046 | 0.00186898 | 3.50E-08 | 0.072308899 | 30.39855699 | Arm fat percentage (left) | ukb-b-20188 |
| rs9491652 | C | G | 0.49621 | -0.0127443 | 0.00152277 | 5.80E-17 | 0.152252746 | 70.04277565 | Arm fat percentage (left) | ukb-b-20188 |
| rs9515455 | A | G | 0.414832 | 0.0101356 | 0.00155491 | 7.10E-11 | 0.098245389 | 42.4901645 | Arm fat percentage (left) | ukb-b-20188 |
| rs9522183 | T | G | 0.56175 | -0.0106643 | 0.00154542 | 5.20E-12 | 0.108811906 | 47.61805485 | Arm fat percentage (left) | ukb-b-20188 |
| rs9536637 | G | T | 0.883926 | -0.0163457 | 0.00240278 | 1.00E-11 | 0.106075539 | 46.27847411 | Arm fat percentage (left) | ukb-b-20188 |
| rs9568867 | A | G | 0.129179 | 0.0204518 | 0.00228928 | 4.10E-19 | 0.169879761 | 79.81145824 | Arm fat percentage (left) | ukb-b-20188 |
| rs9579775 | C | A | 0.135547 | 0.0176133 | 0.0023143 | 2.70E-14 | 0.129312331 | 57.9218139 | Arm fat percentage (left) | ukb-b-20188 |
| rs9641499 | A | C | 0.434712 | -0.0136991 | 0.00153493 | 4.50E-19 | 0.169601235 | 79.65387772 | Arm fat percentage (left) | ukb-b-20188 |
| rs972283 | G | A | 0.511934 | -0.0118013 | 0.00152077 | 8.50E-15 | 0.133754663 | 60.21887364 | Arm fat percentage (left) | ukb-b-20188 |
| rs9770544 | G | C | 0.820331 | -0.0162412 | 0.00199988 | 4.60E-16 | 0.144646914 | 65.95205837 | Arm fat percentage (left) | ukb-b-20188 |
| rs9783304 | T | G | 0.689281 | -0.016753 | 0.00164535 | 2.40E-24 | 0.210004498 | 103.673697 | Arm fat percentage (left) | ukb-b-20188 |
| rs9788550 | C | G | 0.247498 | -0.0160583 | 0.001772 | 1.30E-19 | 0.173946359 | 82.12430346 | Arm fat percentage (left) | ukb-b-20188 |
| rs979259 | G | A | 0.58262 | -0.0108199 | 0.00154854 | 2.80E-12 | 0.111253785 | 48.82043423 | Arm fat percentage (left) | ukb-b-20188 |
| rs9839081 | A | G | 0.325076 | -0.0114425 | 0.00164842 | 3.90E-12 | 0.109963669 | 48.18435967 | Arm fat percentage (left) | ukb-b-20188 |
| rs9843007 | T | A | 0.373492 | -0.01049 | 0.00157103 | 2.40E-11 | 0.102590697 | 44.58430692 | Arm fat percentage (left) | ukb-b-20188 |
| rs9843653 | C | T | 0.511541 | 0.0169984 | 0.001521 | 5.40E-29 | 0.242569352 | 124.8986261 | Arm fat percentage (left) | ukb-b-20188 |
| rs9847672 | T | C | 0.276757 | 0.0157894 | 0.00170164 | 1.70E-20 | 0.180841881 | 86.0985589 | Arm fat percentage (left) | ukb-b-20188 |
| rs9860326 | G | C | 0.328364 | 0.0105287 | 0.00161971 | 8.00E-11 | 0.097754162 | 42.25469542 | Arm fat percentage (left) | ukb-b-20188 |
| rs9873289 | A | G | 0.917959 | 0.0151778 | 0.00277546 | 4.50E-08 | 0.071219084 | 29.90526852 | Arm fat percentage (left) | ukb-b-20188 |
| rs9955276 | T | C | 0.144433 | 0.0144121 | 0.00217818 | 3.70E-11 | 0.100924908 | 43.77911738 | Arm fat percentage (left) | ukb-b-20188 |
| rs9968060 | T | C | 0.642611 | 0.0104043 | 0.0016153 | 1.20E-10 | 0.096150363 | 41.48769879 | Arm fat percentage (left) | ukb-b-20188 |

Supplementary Table 11: Information on all instrumental variables (IVs) ultimately used for Arm fat percentage (right) in our study

| SNPs | effect_allele.exposure | other_allele.exposure | eaf.exposure | beta.exposure | se.exposure | pval.exposure | R^2^ | F | trait | id.exposure |
| --- | --- | --- | --- | --- | --- | --- | --- | --- | --- | --- |
| rs10050620 | T | C | 0.325413 | -0.011488 | 0.00163405 | 2.10E-12 | 0.112735726 | 49.42630801 | Arm fat percentage (right) | ukb-b-12854 |
| rs10070510 | C | T | 0.105231 | -0.0146466 | 0.00251941 | 6.10E-09 | 0.079936333 | 33.79682824 | Arm fat percentage (right) | ukb-b-12854 |
| rs10116857 | A | C | 0.061824 | -0.0225216 | 0.00318253 | 1.50E-12 | 0.114054139 | 50.07874875 | Arm fat percentage (right) | ukb-b-12854 |
| rs1013402 | G | A | 0.318435 | 0.0210424 | 0.00164078 | 1.20E-37 | 0.297163057 | 164.4711907 | Arm fat percentage (right) | ukb-b-12854 |
| rs10139619 | G | A | 0.508028 | -0.00945746 | 0.00153444 | 7.10E-10 | 0.088967889 | 37.98824269 | Arm fat percentage (right) | ukb-b-12854 |
| rs10144067 | T | C | 0.591291 | 0.0112397 | 0.00157086 | 8.40E-13 | 0.11630237 | 51.19581689 | Arm fat percentage (right) | ukb-b-12854 |
| rs10187101 | T | C | 0.363611 | -0.0103288 | 0.00158671 | 7.50E-11 | 0.098231358 | 42.37450311 | Arm fat percentage (right) | ukb-b-12854 |
| rs10245306 | C | G | 0.685975 | 0.00940615 | 0.00166276 | 0.000000015 | 0.076011876 | 32.00108206 | Arm fat percentage (right) | ukb-b-12854 |
| rs10269783 | A | G | 0.387848 | 0.00895858 | 0.00157238 | 0.000000012 | 0.07702041 | 32.46110731 | Arm fat percentage (right) | ukb-b-12854 |
| rs1038088 | G | T | 0.519315 | 0.0123791 | 0.00153079 | 6.10E-16 | 0.143917307 | 65.39535617 | Arm fat percentage (right) | ukb-b-12854 |
| rs10402950 | C | T | 0.289352 | 0.0162923 | 0.00169255 | 6.20E-22 | 0.192372604 | 92.65775693 | Arm fat percentage (right) | ukb-b-12854 |
| rs10405979 | G | A | 0.206466 | -0.010647 | 0.00190027 | 0.000000021 | 0.074673942 | 31.39235406 | Arm fat percentage (right) | ukb-b-12854 |
| rs10423928 | A | T | 0.194427 | -0.0220794 | 0.00193334 | 3.30E-30 | 0.251093756 | 130.4241641 | Arm fat percentage (right) | ukb-b-12854 |
| rs1046080 | A | C | 0.721635 | 0.0163543 | 0.00170276 | 7.60E-22 | 0.191684982 | 92.24801743 | Arm fat percentage (right) | ukb-b-12854 |
| rs10490869 | T | A | 0.209557 | 0.0117671 | 0.00188561 | 4.40E-10 | 0.09100153 | 38.94351456 | Arm fat percentage (right) | ukb-b-12854 |
| rs10505836 | C | A | 0.859995 | 0.0123267 | 0.00222198 | 2.90E-08 | 0.073315478 | 30.77608423 | Arm fat percentage (right) | ukb-b-12854 |
| rs10745787 | A | G | 0.668317 | -0.00922637 | 0.00162396 | 1.30E-08 | 0.076619966 | 32.27833154 | Arm fat percentage (right) | ukb-b-12854 |
| rs10756792 | T | C | 0.743075 | -0.0154943 | 0.00175928 | 1.30E-18 | 0.166249547 | 77.5664633 | Arm fat percentage (right) | ukb-b-12854 |
| rs10760724 | C | T | 0.477321 | 0.0107924 | 0.00153155 | 1.80E-12 | 0.113200777 | 49.6562258 | Arm fat percentage (right) | ukb-b-12854 |
| rs1078455 | C | T | 0.309499 | 0.0114289 | 0.00166636 | 7.00E-12 | 0.107880873 | 47.04042116 | Arm fat percentage (right) | ukb-b-12854 |
| rs10788497 | C | G | 0.499604 | 0.0103178 | 0.00153337 | 1.70E-11 | 0.10425891 | 45.27727554 | Arm fat percentage (right) | ukb-b-12854 |
| rs10799778 | G | T | 0.833684 | -0.015348 | 0.00205156 | 7.40E-14 | 0.125778653 | 55.96740043 | Arm fat percentage (right) | ukb-b-12854 |
| rs10846920 | T | C | 0.739972 | 0.0163648 | 0.00174778 | 7.70E-21 | 0.183920708 | 87.6693675 | Arm fat percentage (right) | ukb-b-12854 |
| rs10854853 | T | G | 0.456803 | 0.0100313 | 0.00153961 | 7.20E-11 | 0.098392277 | 42.45149518 | Arm fat percentage (right) | ukb-b-12854 |
| rs10886022 | C | A | 0.763353 | -0.013575 | 0.00183717 | 1.50E-13 | 0.123080962 | 54.59853459 | Arm fat percentage (right) | ukb-b-12854 |
| rs10892501 | A | G | 0.607105 | -0.00896494 | 0.00157909 | 1.40E-08 | 0.076517387 | 32.23153642 | Arm fat percentage (right) | ukb-b-12854 |
| rs10938398 | A | G | 0.433507 | 0.0207668 | 0.00154627 | 4.00E-41 | 0.316790822 | 180.3717424 | Arm fat percentage (right) | ukb-b-12854 |
| rs10999460 | T | C | 0.265504 | 0.0129715 | 0.00174142 | 9.40E-14 | 0.124829294 | 55.48471301 | Arm fat percentage (right) | ukb-b-12854 |
| rs11012732 | G | A | 0.331707 | 0.0145982 | 0.00162665 | 2.80E-19 | 0.171529176 | 80.53976973 | Arm fat percentage (right) | ukb-b-12854 |
| rs11017772 | T | C | 0.205917 | -0.0112764 | 0.00189779 | 2.80E-09 | 0.083208135 | 35.30568467 | Arm fat percentage (right) | ukb-b-12854 |
| rs11030016 | T | C | 0.739773 | 0.013069 | 0.00174818 | 7.70E-14 | 0.125621074 | 55.88720895 | Arm fat percentage (right) | ukb-b-12854 |
| rs1103804 | G | A | 0.766892 | -0.0108552 | 0.00181301 | 2.10E-09 | 0.08438025 | 35.84885259 | Arm fat percentage (right) | ukb-b-12854 |
| rs11079849 | T | C | 0.328556 | -0.0159716 | 0.00163443 | 1.50E-22 | 0.197096149 | 95.49138659 | Arm fat percentage (right) | ukb-b-12854 |
| rs11105842 | A | G | 0.367521 | -0.0101023 | 0.00159498 | 2.40E-10 | 0.093487634 | 40.11714694 | Arm fat percentage (right) | ukb-b-12854 |
| rs11150745 | G | A | 0.317692 | -0.0133656 | 0.00164823 | 5.10E-16 | 0.144597904 | 65.7568938 | Arm fat percentage (right) | ukb-b-12854 |
| rs11159110 | T | C | 0.142822 | -0.012988 | 0.00219915 | 3.50E-09 | 0.082287158 | 34.87986973 | Arm fat percentage (right) | ukb-b-12854 |
| rs11165643 | T | C | 0.590148 | 0.0151691 | 0.00155156 | 1.40E-22 | 0.197248607 | 95.58340099 | Arm fat percentage (right) | ukb-b-12854 |
| rs11222371 | T | C | 0.409217 | 0.012838 | 0.00155715 | 1.70E-16 | 0.148745449 | 67.97259377 | Arm fat percentage (right) | ukb-b-12854 |
| rs11223970 | G | A | 0.16458 | 0.0130693 | 0.00206727 | 2.60E-10 | 0.09317203 | 39.96780058 | Arm fat percentage (right) | ukb-b-12854 |
| rs112852122 | A | G | 0.157856 | -0.0174076 | 0.00213483 | 3.50E-16 | 0.145973192 | 66.48921463 | Arm fat percentage (right) | ukb-b-12854 |
| rs113079574 | T | C | 0.192748 | -0.0132061 | 0.00194821 | 1.20E-11 | 0.105642611 | 45.94916531 | Arm fat percentage (right) | ukb-b-12854 |
| rs113603865 | T | C | 0.212066 | 0.0123375 | 0.00187963 | 5.20E-11 | 0.099710766 | 43.08336293 | Arm fat percentage (right) | ukb-b-12854 |
| rs114964326 | A | G | 0.028986 | -0.0254193 | 0.0045888 | 3.00E-08 | 0.073114794 | 30.68519688 | Arm fat percentage (right) | ukb-b-12854 |
| rs11648621 | G | A | 0.214852 | -0.0111767 | 0.00187133 | 2.30E-09 | 0.083998762 | 35.67191528 | Arm fat percentage (right) | ukb-b-12854 |
| rs11664106 | T | A | 0.373944 | 0.0096478 | 0.00162279 | 2.80E-09 | 0.08329377 | 35.34532165 | Arm fat percentage (right) | ukb-b-12854 |
| rs11664848 | G | C | 0.659623 | 0.0104257 | 0.00162358 | 1.40E-10 | 0.095842384 | 41.23472117 | Arm fat percentage (right) | ukb-b-12854 |
| rs11691869 | A | C | 0.361918 | -0.0137831 | 0.00159244 | 4.90E-18 | 0.161483973 | 74.91480584 | Arm fat percentage (right) | ukb-b-12854 |
| rs11699828 | A | G | 0.035831 | -0.0263719 | 0.00450941 | 0.000000005 | 0.080815811 | 34.20136138 | Arm fat percentage (right) | ukb-b-12854 |
| rs117118217 | C | G | 0.017707 | 0.0349835 | 0.00610068 | 9.80E-09 | 0.077943178 | 32.88289332 | Arm fat percentage (right) | ukb-b-12854 |
| rs117176448 | G | C | 0.096167 | 0.0146056 | 0.00259398 | 1.80E-08 | 0.075358034 | 31.70337944 | Arm fat percentage (right) | ukb-b-12854 |
| rs11767811 | A | G | 0.181443 | -0.0111755 | 0.00198383 | 1.80E-08 | 0.07542536 | 31.73401472 | Arm fat percentage (right) | ukb-b-12854 |
| rs11786089 | G | A | 0.459691 | 0.0106121 | 0.0015388 | 5.30E-12 | 0.108941853 | 47.55961303 | Arm fat percentage (right) | ukb-b-12854 |
| rs11795079 | C | T | 0.164692 | -0.0121618 | 0.0020639 | 3.80E-09 | 0.081947607 | 34.7230937 | Arm fat percentage (right) | ukb-b-12854 |
| rs118136827 | T | G | 0.281118 | -0.0110126 | 0.00170414 | 1.00E-10 | 0.096946688 | 41.76083649 | Arm fat percentage (right) | ukb-b-12854 |
| rs11866219 | C | A | 0.583795 | -0.0191094 | 0.00157325 | 6.00E-34 | 0.274979036 | 147.5362096 | Arm fat percentage (right) | ukb-b-12854 |
| rs12001437 | C | T | 0.367756 | 0.0090339 | 0.0015872 | 1.30E-08 | 0.076877129 | 32.39569102 | Arm fat percentage (right) | ukb-b-12854 |
| rs12033257 | G | A | 0.382343 | -0.00891963 | 0.0015832 | 1.80E-08 | 0.075440953 | 31.74111048 | Arm fat percentage (right) | ukb-b-12854 |
| rs12042959 | G | A | 0.14393 | -0.0147191 | 0.00218704 | 1.70E-11 | 0.104295208 | 45.29487436 | Arm fat percentage (right) | ukb-b-12854 |
| rs12054057 | A | G | 0.157627 | 0.0138779 | 0.0021034 | 4.20E-11 | 0.100643565 | 43.53151364 | Arm fat percentage (right) | ukb-b-12854 |
| rs12072739 | G | A | 0.224594 | 0.0132583 | 0.00183296 | 4.70E-13 | 0.118553905 | 52.32023768 | Arm fat percentage (right) | ukb-b-12854 |
| rs12101393 | G | C | 0.217309 | -0.0110647 | 0.00186633 | 3.10E-09 | 0.082867615 | 35.14814524 | Arm fat percentage (right) | ukb-b-12854 |
| rs12140153 | T | G | 0.094275 | -0.0257811 | 0.00267877 | 6.30E-22 | 0.192319038 | 92.62581317 | Arm fat percentage (right) | ukb-b-12854 |
| rs12254515 | G | T | 0.56315 | -0.00921589 | 0.00154468 | 2.40E-09 | 0.083834336 | 35.59569836 | Arm fat percentage (right) | ukb-b-12854 |
| rs1229984 | C | T | 0.97278 | 0.0275366 | 0.00464078 | 3.00E-09 | 0.08299655 | 35.20778231 | Arm fat percentage (right) | ukb-b-12854 |
| rs12338803 | C | A | 0.274781 | 0.0107717 | 0.00171634 | 3.50E-10 | 0.091944286 | 39.38781151 | Arm fat percentage (right) | ukb-b-12854 |
| rs12367809 | T | C | 0.369186 | 0.0209867 | 0.00159183 | 1.10E-39 | 0.308835276 | 173.818076 | Arm fat percentage (right) | ukb-b-12854 |
| rs12375196 | A | C | 0.424288 | 0.0116088 | 0.0015578 | 9.20E-14 | 0.12492439 | 55.53301581 | Arm fat percentage (right) | ukb-b-12854 |
| rs12376870 | A | G | 0.237671 | -0.0116899 | 0.00180349 | 9.10E-11 | 0.097477129 | 42.01400811 | Arm fat percentage (right) | ukb-b-12854 |
| rs12441543 | A | G | 0.287111 | 0.0101005 | 0.0016967 | 2.60E-09 | 0.08349507 | 35.43852407 | Arm fat percentage (right) | ukb-b-12854 |
| rs12459368 | G | A | 0.268185 | -0.0137909 | 0.00172761 | 1.40E-15 | 0.140754271 | 63.72264588 | Arm fat percentage (right) | ukb-b-12854 |
| rs12471400 | C | T | 0.517357 | 0.00870135 | 0.00153428 | 1.40E-08 | 0.076368322 | 32.16355376 | Arm fat percentage (right) | ukb-b-12854 |
| rs12475388 | A | G | 0.48571 | -0.00983399 | 0.00153527 | 1.50E-10 | 0.095409659 | 41.0289117 | Arm fat percentage (right) | ukb-b-12854 |
| rs12530791 | A | G | 0.567682 | -0.0107193 | 0.00154453 | 3.90E-12 | 0.110177731 | 48.16595271 | Arm fat percentage (right) | ukb-b-12854 |
| rs12628603 | A | G | 0.617031 | -0.009741 | 0.00157954 | 7.00E-10 | 0.089060618 | 38.031708 | Arm fat percentage (right) | ukb-b-12854 |
| rs12762744 | T | C | 0.248363 | 0.0098917 | 0.00177527 | 0.000000025 | 0.073912118 | 31.0465284 | Arm fat percentage (right) | ukb-b-12854 |
| rs12788343 | C | T | 0.409216 | 0.0115459 | 0.00155499 | 1.10E-13 | 0.124133424 | 55.13157283 | Arm fat percentage (right) | ukb-b-12854 |
| rs12807357 | T | C | 0.315242 | 0.0103998 | 0.00164993 | 2.90E-10 | 0.092669118 | 39.73003397 | Arm fat percentage (right) | ukb-b-12854 |
| rs12881869 | T | C | 0.070529 | 0.0182658 | 0.00299426 | 1.10E-09 | 0.087311483 | 37.21331669 | Arm fat percentage (right) | ukb-b-12854 |
| rs12887636 | G | T | 0.346494 | -0.0125985 | 0.00162104 | 7.70E-15 | 0.134404981 | 60.40184684 | Arm fat percentage (right) | ukb-b-12854 |
| rs12890931 | G | T | 0.362421 | 0.0101123 | 0.00160345 | 2.90E-10 | 0.092760173 | 39.77306378 | Arm fat percentage (right) | ukb-b-12854 |
| rs12926311 | C | G | 0.353553 | -0.0100612 | 0.00160659 | 3.80E-10 | 0.09158496 | 39.21836151 | Arm fat percentage (right) | ukb-b-12854 |
| rs12926311 | C | G | 0.353553 | -0.0100612 | 0.00160659 | 3.80E-10 | 0.09158496 | 39.21836151 | Arm fat percentage (right) | ukb-b-12854 |
| rs1296328 | C | A | 0.558991 | -0.0121652 | 0.00154771 | 3.80E-15 | 0.137054445 | 61.78162531 | Arm fat percentage (right) | ukb-b-12854 |
| rs12975415 | G | A | 0.171165 | -0.0119974 | 0.00204527 | 4.50E-09 | 0.081266737 | 34.40907367 | Arm fat percentage (right) | ukb-b-12854 |
| rs12992672 | A | G | 0.828115 | 0.0322277 | 0.00202075 | 2.90E-57 | 0.395353392 | 254.3510001 | Arm fat percentage (right) | ukb-b-12854 |
| rs13107325 | T | C | 0.074866 | 0.0368686 | 0.00290787 | 7.70E-37 | 0.292411517 | 160.7545667 | Arm fat percentage (right) | ukb-b-12854 |
| rs13160735 | A | G | 0.322388 | 0.0109893 | 0.00164384 | 2.30E-11 | 0.103048214 | 44.69109245 | Arm fat percentage (right) | ukb-b-12854 |
| rs13174863 | G | A | 0.148136 | 0.0158979 | 0.00216936 | 2.30E-13 | 0.12131141 | 53.70519101 | Arm fat percentage (right) | ukb-b-12854 |
| rs1318408 | G | A | 0.119506 | 0.0146216 | 0.00236187 | 6.00E-10 | 0.089685019 | 38.32461652 | Arm fat percentage (right) | ukb-b-12854 |
| rs1320251 | T | C | 0.454747 | -0.00974119 | 0.00154337 | 2.80E-10 | 0.092894911 | 39.83675183 | Arm fat percentage (right) | ukb-b-12854 |
| rs13252030 | G | A | 0.732619 | 0.0115133 | 0.00173491 | 3.20E-11 | 0.101699282 | 44.03984093 | Arm fat percentage (right) | ukb-b-12854 |
| rs13292699 | C | A | 0.433719 | -0.017846 | 0.001547 | 8.70E-31 | 0.254898372 | 133.0764325 | Arm fat percentage (right) | ukb-b-12854 |
| rs13317303 | A | C | 0.150185 | -0.0148447 | 0.00214183 | 4.20E-12 | 0.109914498 | 48.03666583 | Arm fat percentage (right) | ukb-b-12854 |
| rs13375176 | C | T | 0.201967 | -0.0126658 | 0.00191081 | 3.40E-11 | 0.101485864 | 43.93698391 | Arm fat percentage (right) | ukb-b-12854 |
| rs13389219 | T | C | 0.392416 | 0.0152502 | 0.00156161 | 1.60E-22 | 0.1968928 | 95.36871167 | Arm fat percentage (right) | ukb-b-12854 |
| rs1357079 | C | T | 0.570062 | 0.0105126 | 0.00154703 | 1.10E-11 | 0.106110212 | 46.17669104 | Arm fat percentage (right) | ukb-b-12854 |
| rs1366836 | G | T | 0.384725 | 0.00969192 | 0.00156974 | 6.60E-10 | 0.089251038 | 38.1209918 | Arm fat percentage (right) | ukb-b-12854 |
| rs1377184 | T | A | 0.748944 | 0.0110429 | 0.00176838 | 4.20E-10 | 0.091112053 | 38.99555357 | Arm fat percentage (right) | ukb-b-12854 |
| rs1383723 | T | A | 0.783146 | -0.0106672 | 0.00186031 | 9.80E-09 | 0.077936594 | 32.8798808 | Arm fat percentage (right) | ukb-b-12854 |
| rs14057 | A | G | 0.356579 | -0.00894448 | 0.00159833 | 2.20E-08 | 0.074507596 | 31.31679378 | Arm fat percentage (right) | ukb-b-12854 |
| rs141008767 | T | C | 0.018215 | -0.0334445 | 0.00583181 | 9.80E-09 | 0.077955202 | 32.88839521 | Arm fat percentage (right) | ukb-b-12854 |
| rs1412239 | G | C | 0.323103 | 0.0171644 | 0.00163417 | 8.30E-26 | 0.220943861 | 110.3221677 | Arm fat percentage (right) | ukb-b-12854 |
| rs1421334 | C | A | 0.548845 | -0.0103703 | 0.00154608 | 2.00E-11 | 0.103666566 | 44.99028206 | Arm fat percentage (right) | ukb-b-12854 |
| rs1441264 | A | G | 0.593692 | 0.0133437 | 0.00159333 | 5.50E-17 | 0.152756505 | 70.13601268 | Arm fat percentage (right) | ukb-b-12854 |
| rs144376234 | T | C | 0.025647 | 0.053775 | 0.00484029 | 1.10E-28 | 0.240870806 | 123.4292451 | Arm fat percentage (right) | ukb-b-12854 |
| rs1454687 | G | C | 0.515343 | -0.0148595 | 0.0015285 | 2.40E-22 | 0.195466392 | 94.50994414 | Arm fat percentage (right) | ukb-b-12854 |
| rs147819910 | T | G | 0.018204 | 0.033022 | 0.00572461 | 0.000000008 | 0.078798838 | 33.27476036 | Arm fat percentage (right) | ukb-b-12854 |
| rs149423876 | A | G | 0.258748 | 0.00972829 | 0.00175083 | 2.80E-08 | 0.073530353 | 30.87344252 | Arm fat percentage (right) | ukb-b-12854 |
| rs1495181 | G | A | 0.282289 | -0.00926603 | 0.00169874 | 4.90E-08 | 0.071051859 | 29.75319287 | Arm fat percentage (right) | ukb-b-12854 |
| rs1503526 | C | T | 0.479962 | 0.0117844 | 0.0015306 | 1.40E-14 | 0.132234377 | 59.27772571 | Arm fat percentage (right) | ukb-b-12854 |
| rs1559677 | G | A | 0.395922 | 0.0103072 | 0.00156664 | 4.70E-11 | 0.100131855 | 43.28555441 | Arm fat percentage (right) | ukb-b-12854 |
| rs1559900 | T | C | 0.286127 | 0.0101351 | 0.00169318 | 2.20E-09 | 0.084340153 | 35.83024818 | Arm fat percentage (right) | ukb-b-12854 |
| rs1605898 | A | T | 0.837615 | 0.0119344 | 0.00207606 | 9.00E-09 | 0.078299934 | 33.04618866 | Arm fat percentage (right) | ukb-b-12854 |
| rs1609010 | G | A | 0.565696 | 0.0164601 | 0.00154548 | 1.70E-26 | 0.225766909 | 113.4326714 | Arm fat percentage (right) | ukb-b-12854 |
| rs1657930 | A | G | 0.803029 | -0.0115307 | 0.00192443 | 2.10E-09 | 0.084492723 | 35.90104656 | Arm fat percentage (right) | ukb-b-12854 |
| rs16916303 | G | A | 0.119773 | -0.0136218 | 0.00238327 | 1.10E-08 | 0.077473266 | 32.66799697 | Arm fat percentage (right) | ukb-b-12854 |
| rs17193211 | T | C | 0.066795 | -0.0178038 | 0.00312285 | 0.000000012 | 0.077112098 | 32.50297886 | Arm fat percentage (right) | ukb-b-12854 |
| rs172558 | T | C | 0.251402 | 0.0122 | 0.00178482 | 8.20E-12 | 0.107230991 | 46.72301005 | Arm fat percentage (right) | ukb-b-12854 |
| rs17446299 | G | C | 0.165547 | 0.0120902 | 0.00206277 | 4.60E-09 | 0.081145167 | 34.3530544 | Arm fat percentage (right) | ukb-b-12854 |
| rs17698176 | G | T | 0.226632 | 0.0106297 | 0.00184703 | 8.70E-09 | 0.078461732 | 33.12028891 | Arm fat percentage (right) | ukb-b-12854 |
| rs17820010 | G | T | 0.2956 | 0.0105712 | 0.00167611 | 2.80E-10 | 0.092770734 | 39.77805506 | Arm fat percentage (right) | ukb-b-12854 |
| rs1787013 | C | T | 0.450724 | 0.00865155 | 0.00153869 | 1.90E-08 | 0.075162531 | 31.61444641 | Arm fat percentage (right) | ukb-b-12854 |
| rs1801282 | G | C | 0.119544 | 0.0242518 | 0.00235013 | 5.80E-25 | 0.214916761 | 106.4888613 | Arm fat percentage (right) | ukb-b-12854 |
| rs1808579 | T | C | 0.482049 | -0.0145167 | 0.00153492 | 3.10E-21 | 0.186952274 | 89.44669818 | Arm fat percentage (right) | ukb-b-12854 |
| rs1808629 | A | G | 0.685439 | -0.0149997 | 0.00165503 | 1.30E-19 | 0.174342654 | 82.1397554 | Arm fat percentage (right) | ukb-b-12854 |
| rs1852006 | A | G | 0.35697 | -0.010828 | 0.00159679 | 1.20E-11 | 0.105712957 | 45.98337948 | Arm fat percentage (right) | ukb-b-12854 |
| rs1861410 | T | C | 0.555357 | -0.0137315 | 0.0015409 | 5.00E-19 | 0.16953481 | 79.4121679 | Arm fat percentage (right) | ukb-b-12854 |
| rs1888623 | T | A | 0.227344 | -0.0125672 | 0.00182195 | 5.30E-12 | 0.108978925 | 47.5777768 | Arm fat percentage (right) | ukb-b-12854 |
| rs1945160 | A | G | 0.37586 | -0.00927727 | 0.00158836 | 5.20E-09 | 0.080627709 | 34.11477484 | Arm fat percentage (right) | ukb-b-12854 |
| rs1964675 | T | C | 0.71109 | 0.00985324 | 0.0016887 | 5.40E-09 | 0.080476034 | 34.04498253 | Arm fat percentage (right) | ukb-b-12854 |
| rs1991002 | G | T | 0.495154 | -0.00923461 | 0.00153451 | 1.80E-09 | 0.085170327 | 36.2157657 | Arm fat percentage (right) | ukb-b-12854 |
| rs2033531 | C | T | 0.404545 | 0.0106275 | 0.0015625 | 1.00E-11 | 0.106284922 | 46.26176256 | Arm fat percentage (right) | ukb-b-12854 |
| rs2035831 | C | G | 0.33186 | -0.0088838 | 0.00162803 | 4.80E-08 | 0.071103422 | 29.77643762 | Arm fat percentage (right) | ukb-b-12854 |
| rs2035936 | T | G | 0.055879 | 0.0253919 | 0.00337516 | 5.30E-14 | 0.127015979 | 56.59807589 | Arm fat percentage (right) | ukb-b-12854 |
| rs2052607 | A | G | 0.343892 | -0.0134855 | 0.00161739 | 7.60E-17 | 0.151616721 | 69.51917338 | Arm fat percentage (right) | ukb-b-12854 |
| rs2058527 | G | T | 0.730584 | 0.00986094 | 0.00172622 | 1.10E-08 | 0.077394612 | 32.632049 | Arm fat percentage (right) | ukb-b-12854 |
| rs2111281 | C | A | 0.366941 | 0.00951192 | 0.00158914 | 2.20E-09 | 0.084333439 | 35.82713328 | Arm fat percentage (right) | ukb-b-12854 |
| rs2119263 | T | C | 0.223475 | -0.0104268 | 0.00183953 | 1.40E-08 | 0.076291098 | 32.12834367 | Arm fat percentage (right) | ukb-b-12854 |
| rs215498 | T | G | 0.324131 | 0.00924787 | 0.00164514 | 1.90E-08 | 0.075129266 | 31.59931792 | Arm fat percentage (right) | ukb-b-12854 |
| rs2161097 | T | C | 0.437923 | 0.00948719 | 0.00153971 | 7.20E-10 | 0.088920894 | 37.96621788 | Arm fat percentage (right) | ukb-b-12854 |
| rs2169935 | T | C | 0.491696 | -0.00840088 | 0.00153824 | 4.70E-08 | 0.071214311 | 29.82643596 | Arm fat percentage (right) | ukb-b-12854 |
| rs2172131 | C | T | 0.578565 | -0.010172 | 0.00155144 | 5.50E-11 | 0.099511094 | 42.98755397 | Arm fat percentage (right) | ukb-b-12854 |
| rs217672 | C | A | 0.271725 | 0.0123986 | 0.0017264 | 6.90E-13 | 0.117068452 | 51.57775604 | Arm fat percentage (right) | ukb-b-12854 |
| rs2192527 | G | A | 0.465491 | 0.0131186 | 0.0015344 | 1.20E-17 | 0.158184865 | 73.09670502 | Arm fat percentage (right) | ukb-b-12854 |
| rs2234458 | T | C | 0.639605 | -0.0106206 | 0.00159177 | 2.50E-11 | 0.102690472 | 44.5181875 | Arm fat percentage (right) | ukb-b-12854 |
| rs2237025 | C | T | 0.5554 | -0.00867191 | 0.00155115 | 2.30E-08 | 0.074371897 | 31.25517462 | Arm fat percentage (right) | ukb-b-12854 |
| rs2239647 | C | A | 0.551117 | -0.0139533 | 0.00154259 | 1.50E-19 | 0.173779719 | 81.81875 | Arm fat percentage (right) | ukb-b-12854 |
| rs2253310 | G | C | 0.626168 | 0.00998196 | 0.00158038 | 2.70E-10 | 0.093016188 | 39.89409344 | Arm fat percentage (right) | ukb-b-12854 |
| rs2279574 | A | C | 0.541385 | -0.0142551 | 0.00153345 | 1.50E-20 | 0.181771674 | 86.4174201 | Arm fat percentage (right) | ukb-b-12854 |
| rs2292238 | C | A | 0.407675 | -0.00915468 | 0.00155925 | 4.30E-09 | 0.081401251 | 34.47107529 | Arm fat percentage (right) | ukb-b-12854 |
| rs2307111 | C | T | 0.395094 | -0.0168907 | 0.00156536 | 3.80E-27 | 0.230359099 | 116.4305188 | Arm fat percentage (right) | ukb-b-12854 |
| rs2326844 | C | G | 0.634235 | 0.0141508 | 0.00159332 | 6.60E-19 | 0.168586709 | 78.87801459 | Arm fat percentage (right) | ukb-b-12854 |
| rs2370982 | T | C | 0.214154 | 0.0190581 | 0.00188101 | 4.00E-24 | 0.208793601 | 102.6542641 | Arm fat percentage (right) | ukb-b-12854 |
| rs2388334 | G | A | 0.482465 | -0.0137976 | 0.00153361 | 2.30E-19 | 0.172239362 | 80.94261617 | Arm fat percentage (right) | ukb-b-12854 |
| rs2439823 | G | A | 0.545574 | 0.0136319 | 0.00154154 | 9.30E-19 | 0.167378992 | 78.1993578 | Arm fat percentage (right) | ukb-b-12854 |
| rs2452774 | G | A | 0.884359 | 0.0132057 | 0.00239035 | 3.30E-08 | 0.072752191 | 30.52107754 | Arm fat percentage (right) | ukb-b-12854 |
| rs2465043 | A | G | 0.351577 | -0.009967 | 0.00160897 | 5.80E-10 | 0.089789445 | 38.37364234 | Arm fat percentage (right) | ukb-b-12854 |
| rs2466103 | G | T | 0.303562 | 0.0101391 | 0.00166331 | 1.10E-09 | 0.087193029 | 37.1580073 | Arm fat percentage (right) | ukb-b-12854 |
| rs252748 | T | C | 0.590936 | -0.00878406 | 0.00156351 | 1.90E-08 | 0.075051154 | 31.56379838 | Arm fat percentage (right) | ukb-b-12854 |
| rs2569881 | A | G | 0.12866 | -0.0141204 | 0.00230011 | 8.30E-10 | 0.088325665 | 37.6874529 | Arm fat percentage (right) | ukb-b-12854 |
| rs2583636 | T | G | 0.449879 | -0.00888591 | 0.00153191 | 6.60E-09 | 0.079608648 | 33.6463004 | Arm fat percentage (right) | ukb-b-12854 |
| rs2606227 | C | T | 0.63138 | -0.0105045 | 0.00159968 | 5.10E-11 | 0.099788293 | 43.12057473 | Arm fat percentage (right) | ukb-b-12854 |
| rs2610245 | G | A | 0.51788 | -0.00905303 | 0.00155645 | 6.00E-09 | 0.080011154 | 33.83121316 | Arm fat percentage (right) | ukb-b-12854 |
| rs2678204 | G | T | 0.340181 | 0.014592 | 0.00161217 | 1.40E-19 | 0.173963325 | 81.92340059 | Arm fat percentage (right) | ukb-b-12854 |
| rs2693823 | A | T | 0.260546 | -0.00960075 | 0.00174701 | 3.90E-08 | 0.072043924 | 30.20087599 | Arm fat percentage (right) | ukb-b-12854 |
| rs2717609 | T | A | 0.46612 | -0.00925176 | 0.00154774 | 2.30E-09 | 0.084127524 | 35.7316197 | Arm fat percentage (right) | ukb-b-12854 |
| rs2765041 | A | T | 0.710445 | -0.0116518 | 0.00168841 | 5.20E-12 | 0.109074225 | 47.62447614 | Arm fat percentage (right) | ukb-b-12854 |
| rs2798297 | A | G | 0.366707 | 0.00893707 | 0.00159909 | 2.30E-08 | 0.074327934 | 31.23521534 | Arm fat percentage (right) | ukb-b-12854 |
| rs2820446 | G | C | 0.29729 | 0.0180629 | 0.00166842 | 2.60E-27 | 0.231544022 | 117.209869 | Arm fat percentage (right) | ukb-b-12854 |
| rs28350 | G | A | 0.820612 | -0.0118376 | 0.00199956 | 3.20E-09 | 0.082650183 | 35.04761269 | Arm fat percentage (right) | ukb-b-12854 |
| rs28375268 | T | G | 0.645125 | -0.0103978 | 0.00160701 | 9.80E-11 | 0.097163928 | 41.86448573 | Arm fat percentage (right) | ukb-b-12854 |
| rs2943653 | T | C | 0.672841 | -0.0149593 | 0.00162687 | 3.70E-20 | 0.178546123 | 84.55062888 | Arm fat percentage (right) | ukb-b-12854 |
| rs2954021 | G | A | 0.504594 | 0.0118757 | 0.00153075 | 8.60E-15 | 0.133992891 | 60.18799828 | Arm fat percentage (right) | ukb-b-12854 |
| rs2966859 | G | A | 0.789009 | -0.0108616 | 0.00188343 | 8.10E-09 | 0.078761023 | 33.25742669 | Arm fat percentage (right) | ukb-b-12854 |
| rs2980237 | T | G | 0.751219 | 0.0107019 | 0.00177498 | 1.60E-09 | 0.085464511 | 36.35254739 | Arm fat percentage (right) | ukb-b-12854 |
| rs3087523 | A | G | 0.124621 | 0.0133671 | 0.00232487 | 8.90E-09 | 0.078325806 | 33.05803592 | Arm fat percentage (right) | ukb-b-12854 |
| rs3113509 | T | C | 0.731973 | -0.00955767 | 0.00172772 | 0.000000032 | 0.072932188 | 30.60253065 | Arm fat percentage (right) | ukb-b-12854 |
| rs316611 | T | C | 0.251319 | 0.0104311 | 0.00176884 | 3.70E-09 | 0.082062827 | 34.7762794 | Arm fat percentage (right) | ukb-b-12854 |
| rs319788 | T | C | 0.696268 | 0.00924182 | 0.00166414 | 2.80E-08 | 0.073459834 | 30.84148579 | Arm fat percentage (right) | ukb-b-12854 |
| rs323782 | A | G | 0.021388 | 0.0325295 | 0.0052909 | 7.80E-10 | 0.088566792 | 37.80033679 | Arm fat percentage (right) | ukb-b-12854 |
| rs324014 | C | T | 0.45245 | 0.00868808 | 0.00154215 | 1.80E-08 | 0.0754365 | 31.73908381 | Arm fat percentage (right) | ukb-b-12854 |
| rs326894 | T | C | 0.602056 | 0.00862166 | 0.0015628 | 3.50E-08 | 0.072562158 | 30.43511726 | Arm fat percentage (right) | ukb-b-12854 |
| rs34234296 | A | G | 0.392486 | -0.0103643 | 0.00158024 | 5.40E-11 | 0.099571176 | 43.01637894 | Arm fat percentage (right) | ukb-b-12854 |
| rs34331990 | G | T | 0.41875 | 0.0108282 | 0.0015617 | 4.10E-12 | 0.109992119 | 48.07478126 | Arm fat percentage (right) | ukb-b-12854 |
| rs34483452 | A | C | 0.136321 | 0.0240508 | 0.00224761 | 1.00E-26 | 0.227412858 | 114.503073 | Arm fat percentage (right) | ukb-b-12854 |
| rs34580448 | C | T | 0.041254 | -0.0259364 | 0.00385288 | 1.70E-11 | 0.1043381 | 45.31567204 | Arm fat percentage (right) | ukb-b-12854 |
| rs347551 | G | C | 0.472311 | 0.0112393 | 0.00155656 | 5.20E-13 | 0.118187956 | 52.13709093 | Arm fat percentage (right) | ukb-b-12854 |
| rs34882821 | T | G | 0.338499 | 0.00915016 | 0.00162242 | 0.000000017 | 0.075587021 | 31.80759213 | Arm fat percentage (right) | ukb-b-12854 |
| rs34898535 | T | C | 0.377903 | -0.0159011 | 0.00157964 | 7.80E-24 | 0.20665676 | 101.3300117 | Arm fat percentage (right) | ukb-b-12854 |
| rs34914000 | C | T | 0.206895 | 0.0104549 | 0.00188994 | 0.000000032 | 0.072930069 | 30.60157172 | Arm fat percentage (right) | ukb-b-12854 |
| rs34930419 | C | T | 0.033187 | 0.0263035 | 0.00431827 | 1.10E-09 | 0.08707491 | 37.10286915 | Arm fat percentage (right) | ukb-b-12854 |
| rs34994596 | C | T | 0.297379 | -0.0113108 | 0.00167594 | 1.50E-11 | 0.104817046 | 45.5480421 | Arm fat percentage (right) | ukb-b-12854 |
| rs35023999 | C | A | 0.508314 | -0.010075 | 0.0015316 | 4.80E-11 | 0.100102067 | 43.27124534 | Arm fat percentage (right) | ukb-b-12854 |
| rs35160042 | A | G | 0.238397 | 0.0105699 | 0.00179315 | 3.80E-09 | 0.081997876 | 34.74629608 | Arm fat percentage (right) | ukb-b-12854 |
| rs35198068 | C | T | 0.291522 | -0.00937845 | 0.00168453 | 2.60E-08 | 0.073800543 | 30.99592741 | Arm fat percentage (right) | ukb-b-12854 |
| rs35449999 | G | A | 0.13641 | -0.0133413 | 0.00223382 | 2.30E-09 | 0.083994071 | 35.66974053 | Arm fat percentage (right) | ukb-b-12854 |
| rs35697691 | G | C | 0.089406 | 0.0202386 | 0.00272498 | 1.10E-13 | 0.124191995 | 55.16127498 | Arm fat percentage (right) | ukb-b-12854 |
| rs35949039 | T | G | 0.105573 | -0.0153811 | 0.00251079 | 9.00E-10 | 0.087984585 | 37.52787824 | Arm fat percentage (right) | ukb-b-12854 |
| rs3732103 | T | C | 0.561693 | 0.00895974 | 0.00154321 | 6.40E-09 | 0.079744304 | 33.70860318 | Arm fat percentage (right) | ukb-b-12854 |
| rs3733336 | G | A | 0.35985 | -0.00916461 | 0.00167301 | 4.30E-08 | 0.07161583 | 30.00757518 | Arm fat percentage (right) | ukb-b-12854 |
| rs3751859 | A | G | 0.156478 | -0.0138881 | 0.00210662 | 4.30E-11 | 0.100499744 | 43.46235591 | Arm fat percentage (right) | ukb-b-12854 |
| rs3764002 | T | C | 0.261508 | -0.0146118 | 0.00174089 | 4.70E-17 | 0.153330661 | 70.44736882 | Arm fat percentage (right) | ukb-b-12854 |
| rs3766823 | A | G | 0.17202 | 0.0130996 | 0.00202427 | 9.70E-11 | 0.097190883 | 41.87734997 | Arm fat percentage (right) | ukb-b-12854 |
| rs3770799 | G | A | 0.369632 | 0.0118869 | 0.00158186 | 5.70E-14 | 0.126760744 | 56.46783405 | Arm fat percentage (right) | ukb-b-12854 |
| rs3771653 | T | C | 0.626577 | 0.0091373 | 0.00157989 | 7.30E-09 | 0.079178609 | 33.44891753 | Arm fat percentage (right) | ukb-b-12854 |
| rs3772882 | A | C | 0.373219 | 0.00943485 | 0.00157947 | 2.30E-09 | 0.084020168 | 35.68183963 | Arm fat percentage (right) | ukb-b-12854 |
| rs3784710 | C | T | 0.226633 | -0.0216654 | 0.00182692 | 1.90E-32 | 0.265532347 | 140.6353061 | Arm fat percentage (right) | ukb-b-12854 |
| rs3802422 | A | G | 0.373594 | -0.00989225 | 0.00158077 | 3.90E-10 | 0.091463071 | 39.16091192 | Arm fat percentage (right) | ukb-b-12854 |
| rs3803286 | G | A | 0.66675 | -0.0117433 | 0.00162442 | 4.90E-13 | 0.118437002 | 52.2617148 | Arm fat percentage (right) | ukb-b-12854 |
| rs3806114 | A | G | 0.668517 | -0.0102569 | 0.00164282 | 4.30E-10 | 0.091081013 | 38.98093751 | Arm fat percentage (right) | ukb-b-12854 |
| rs383701 | A | G | 0.868882 | -0.0127433 | 0.00226583 | 1.90E-08 | 0.075198326 | 31.63072667 | Arm fat percentage (right) | ukb-b-12854 |
| rs3901286 | A | C | 0.152427 | -0.0158677 | 0.00213384 | 1.00E-13 | 0.124460214 | 55.29734221 | Arm fat percentage (right) | ukb-b-12854 |
| rs3911063 | C | T | 0.321995 | -0.010884 | 0.00163922 | 3.10E-11 | 0.101795421 | 44.08619099 | Arm fat percentage (right) | ukb-b-12854 |
| rs396354 | C | T | 0.715649 | -0.0105363 | 0.00169678 | 5.30E-10 | 0.090183935 | 38.55894853 | Arm fat percentage (right) | ukb-b-12854 |
| rs40067 | A | G | 0.170164 | -0.0157748 | 0.00204146 | 1.10E-14 | 0.133070066 | 59.70984913 | Arm fat percentage (right) | ukb-b-12854 |
| rs400997 | A | T | 0.559372 | 0.0130854 | 0.00155516 | 4.00E-17 | 0.15397717 | 70.79846653 | Arm fat percentage (right) | ukb-b-12854 |
| rs4055791 | T | C | 0.416764 | -0.0108922 | 0.00155465 | 2.40E-12 | 0.112048397 | 49.08693912 | Arm fat percentage (right) | ukb-b-12854 |
| rs4088476 | T | C | 0.660795 | 0.00922221 | 0.00161402 | 1.10E-08 | 0.077428792 | 32.64766991 | Arm fat percentage (right) | ukb-b-12854 |
| rs4123668 | C | T | 0.402711 | 0.010167 | 0.00156053 | 7.30E-11 | 0.09838174 | 42.44645284 | Arm fat percentage (right) | ukb-b-12854 |
| rs4234589 | G | A | 0.137194 | -0.0186952 | 0.00222169 | 3.90E-17 | 0.153998006 | 70.80979069 | Arm fat percentage (right) | ukb-b-12854 |
| rs42523 | C | T | 0.245209 | 0.0098926 | 0.00178192 | 2.80E-08 | 0.073414272 | 30.8208416 | Arm fat percentage (right) | ukb-b-12854 |
| rs429343 | G | A | 0.57656 | -0.0113197 | 0.00154726 | 2.60E-13 | 0.120950329 | 53.52334383 | Arm fat percentage (right) | ukb-b-12854 |
| rs429358 | C | T | 0.154046 | -0.0192068 | 0.00212406 | 1.50E-19 | 0.173688367 | 81.76669939 | Arm fat percentage (right) | ukb-b-12854 |
| rs4307239 | G | A | 0.458845 | 0.00851542 | 0.00153921 | 0.000000032 | 0.072941382 | 30.60669206 | Arm fat percentage (right) | ukb-b-12854 |
| rs4377779 | C | T | 0.346836 | -0.0110138 | 0.00160747 | 7.30E-12 | 0.107685442 | 46.94492167 | Arm fat percentage (right) | ukb-b-12854 |
| rs4419475 | T | A | 0.407329 | 0.00903241 | 0.00155655 | 6.50E-09 | 0.079666673 | 33.67294758 | Arm fat percentage (right) | ukb-b-12854 |
| rs4430672 | C | T | 0.800929 | -0.0116049 | 0.00192184 | 1.60E-09 | 0.085701199 | 36.46266005 | Arm fat percentage (right) | ukb-b-12854 |
| rs445077 | A | T | 0.480126 | 0.0125588 | 0.00153506 | 2.80E-16 | 0.14680599 | 66.93381489 | Arm fat percentage (right) | ukb-b-12854 |
| rs4466418 | A | G | 0.562255 | 0.00897333 | 0.00154523 | 6.40E-09 | 0.079774768 | 33.72259731 | Arm fat percentage (right) | ukb-b-12854 |
| rs4482463 | A | C | 0.923053 | -0.0218383 | 0.00287226 | 2.90E-14 | 0.129380531 | 57.8082943 | Arm fat percentage (right) | ukb-b-12854 |
| rs4489042 | C | G | 0.373325 | -0.00941757 | 0.00158348 | 2.70E-09 | 0.083350156 | 35.3714244 | Arm fat percentage (right) | ukb-b-12854 |
| rs4549685 | T | C | 0.329799 | -0.0117922 | 0.00162867 | 4.50E-13 | 0.118759492 | 52.42319417 | Arm fat percentage (right) | ukb-b-12854 |
| rs4562625 | G | C | 0.614927 | -0.0103189 | 0.00156871 | 4.80E-11 | 0.100098351 | 43.26946021 | Arm fat percentage (right) | ukb-b-12854 |
| rs4605363 | C | A | 0.341583 | 0.0118838 | 0.00160907 | 1.50E-13 | 0.122976573 | 54.54573432 | Arm fat percentage (right) | ukb-b-12854 |
| rs4673617 | T | C | 0.271776 | 0.0106849 | 0.00172127 | 5.40E-10 | 0.090130623 | 38.53389635 | Arm fat percentage (right) | ukb-b-12854 |
| rs4705778 | C | T | 0.538609 | -0.010673 | 0.00153499 | 3.60E-12 | 0.110544365 | 48.34615252 | Arm fat percentage (right) | ukb-b-12854 |
| rs4722398 | T | C | 0.136116 | 0.0144677 | 0.00222667 | 8.20E-11 | 0.097901965 | 42.21699078 | Arm fat percentage (right) | ukb-b-12854 |
| rs4737188 | T | A | 0.473836 | -0.00911242 | 0.00153481 | 2.90E-09 | 0.083087643 | 35.24992658 | Arm fat percentage (right) | ukb-b-12854 |
| rs474376 | A | C | 0.78906 | 0.0109786 | 0.00188106 | 5.30E-09 | 0.080516172 | 34.06344964 | Arm fat percentage (right) | ukb-b-12854 |
| rs4762951 | G | A | 0.78043 | -0.0106894 | 0.00184979 | 7.50E-09 | 0.079057775 | 33.39348945 | Arm fat percentage (right) | ukb-b-12854 |
| rs4771121 | A | G | 0.27512 | -0.0105244 | 0.00172411 | 0.000000001 | 0.087415462 | 37.26187891 | Arm fat percentage (right) | ukb-b-12854 |
| rs477895 | T | C | 0.836499 | 0.0147005 | 0.00206689 | 1.10E-12 | 0.115076258 | 50.58590041 | Arm fat percentage (right) | ukb-b-12854 |
| rs4790841 | T | C | 0.154508 | -0.0204477 | 0.00212852 | 7.50E-22 | 0.19174793 | 92.28549775 | Arm fat percentage (right) | ukb-b-12854 |
| rs4793085 | C | A | 0.656342 | -0.0124383 | 0.0016199 | 1.60E-14 | 0.131615781 | 58.95839385 | Arm fat percentage (right) | ukb-b-12854 |
| rs4800754 | T | A | 0.611525 | -0.00887605 | 0.00157338 | 0.000000017 | 0.075625874 | 31.82527971 | Arm fat percentage (right) | ukb-b-12854 |
| rs4820323 | G | C | 0.580959 | -0.0137244 | 0.00155733 | 1.20E-18 | 0.166425623 | 77.66501575 | Arm fat percentage (right) | ukb-b-12854 |
| rs4837119 | A | T | 0.517264 | -0.0130038 | 0.00153581 | 2.50E-17 | 0.155616522 | 71.69115556 | Arm fat percentage (right) | ukb-b-12854 |
| rs4864201 | C | T | 0.65096 | -0.0105982 | 0.00160183 | 3.70E-11 | 0.101150651 | 43.77552623 | Arm fat percentage (right) | ukb-b-12854 |
| rs4876611 | G | A | 0.720196 | 0.0178598 | 0.00170653 | 1.20E-25 | 0.219702872 | 109.5280429 | Arm fat percentage (right) | ukb-b-12854 |
| rs4887872 | C | T | 0.194574 | 0.0107455 | 0.00194061 | 3.10E-08 | 0.073059857 | 30.66032309 | Arm fat percentage (right) | ukb-b-12854 |
| rs4929923 | C | T | 0.64521 | 0.0140363 | 0.00159829 | 1.60E-18 | 0.165459573 | 77.12481216 | Arm fat percentage (right) | ukb-b-12854 |
| rs4945884 | C | T | 0.709602 | 0.00985685 | 0.00168976 | 5.40E-09 | 0.080437386 | 34.02720242 | Arm fat percentage (right) | ukb-b-12854 |
| rs518640 | T | C | 0.679001 | 0.01012 | 0.00164105 | 7.00E-10 | 0.089055307 | 38.02921826 | Arm fat percentage (right) | ukb-b-12854 |
| rs538656 | T | G | 0.233829 | 0.0300779 | 0.00180879 | 4.30E-62 | 0.415490276 | 276.5150193 | Arm fat percentage (right) | ukb-b-12854 |
| rs539515 | C | A | 0.204986 | 0.0338661 | 0.00189162 | 1.10E-71 | 0.451746268 | 320.5254943 | Arm fat percentage (right) | ukb-b-12854 |
| rs55707359 | G | T | 0.015448 | 0.0391959 | 0.0062864 | 4.50E-10 | 0.090857278 | 38.8756138 | Arm fat percentage (right) | ukb-b-12854 |
| rs55714539 | C | A | 0.343607 | 0.0157869 | 0.00162562 | 2.70E-22 | 0.1951328 | 94.30954476 | Arm fat percentage (right) | ukb-b-12854 |
| rs55726687 | A | G | 0.209741 | 0.0144378 | 0.00187791 | 1.50E-14 | 0.131907344 | 59.10884779 | Arm fat percentage (right) | ukb-b-12854 |
| rs55769038 | A | G | 0.590297 | 0.0117802 | 0.00155687 | 3.80E-14 | 0.128297789 | 57.25331365 | Arm fat percentage (right) | ukb-b-12854 |
| rs55810445 | T | C | 0.151188 | -0.0149876 | 0.00213971 | 2.50E-12 | 0.111999931 | 49.06302879 | Arm fat percentage (right) | ukb-b-12854 |
| rs56203712 | G | A | 0.233843 | -0.0141249 | 0.00184603 | 2.00E-14 | 0.130814563 | 58.54546431 | Arm fat percentage (right) | ukb-b-12854 |
| rs56218501 | T | C | 0.21181 | -0.0120066 | 0.00187421 | 1.50E-10 | 0.095432234 | 41.03964384 | Arm fat percentage (right) | ukb-b-12854 |
| rs56356382 | C | T | 0.192338 | -0.0163725 | 0.00195201 | 5.00E-17 | 0.153151735 | 70.35029482 | Arm fat percentage (right) | ukb-b-12854 |
| rs56369689 | G | A | 0.348604 | -0.0123395 | 0.00164372 | 6.00E-14 | 0.126541282 | 56.35590725 | Arm fat percentage (right) | ukb-b-12854 |
| rs56375099 | T | C | 0.266252 | -0.0113035 | 0.00173975 | 8.20E-11 | 0.09789483 | 42.2135802 | Arm fat percentage (right) | ukb-b-12854 |
| rs56399737 | T | C | 0.449163 | -0.0121198 | 0.00154534 | 4.40E-15 | 0.136533359 | 61.50958744 | Arm fat percentage (right) | ukb-b-12854 |
| rs56858768 | A | G | 0.296903 | 0.0122288 | 0.00168265 | 3.70E-13 | 0.119546515 | 52.81777532 | Arm fat percentage (right) | ukb-b-12854 |
| rs57636386 | C | T | 0.08384 | -0.0278968 | 0.00277315 | 8.30E-24 | 0.206439386 | 101.1956991 | Arm fat percentage (right) | ukb-b-12854 |
| rs57989773 | C | T | 0.244921 | 0.0122955 | 0.00182992 | 1.80E-11 | 0.10398997 | 45.14692555 | Arm fat percentage (right) | ukb-b-12854 |
| rs58862095 | T | C | 0.419267 | -0.014967 | 0.00155477 | 6.20E-22 | 0.192392435 | 92.66958392 | Arm fat percentage (right) | ukb-b-12854 |
| rs58948644 | A | G | 0.582744 | 0.0101852 | 0.00154993 | 5.00E-11 | 0.099918766 | 43.18321315 | Arm fat percentage (right) | ukb-b-12854 |
| rs59086897 | A | T | 0.487602 | 0.0262812 | 0.00152586 | 1.80E-66 | 0.432664557 | 296.6613754 | Arm fat percentage (right) | ukb-b-12854 |
| rs6021948 | A | T | 0.321773 | -0.0118982 | 0.00164381 | 4.50E-13 | 0.118695843 | 52.3913143 | Arm fat percentage (right) | ukb-b-12854 |
| rs6023655 | G | A | 0.765799 | -0.0100981 | 0.00181869 | 2.80E-08 | 0.073432676 | 30.82918022 | Arm fat percentage (right) | ukb-b-12854 |
| rs6044084 | G | A | 0.586913 | 0.00881235 | 0.00156004 | 0.000000016 | 0.075809544 | 31.90891282 | Arm fat percentage (right) | ukb-b-12854 |
| rs6103254 | C | T | 0.126697 | -0.0139389 | 0.00231193 | 1.60E-09 | 0.085459615 | 36.35027023 | Arm fat percentage (right) | ukb-b-12854 |
| rs6138536 | G | A | 0.462462 | 0.0112564 | 0.00153757 | 2.50E-13 | 0.121093864 | 53.59561287 | Arm fat percentage (right) | ukb-b-12854 |
| rs61754230 | T | C | 0.019707 | 0.0305377 | 0.00550326 | 2.90E-08 | 0.07334978 | 30.79162335 | Arm fat percentage (right) | ukb-b-12854 |
| rs61813324 | T | C | 0.13571 | 0.019371 | 0.00226223 | 1.10E-17 | 0.158593944 | 73.32136939 | Arm fat percentage (right) | ukb-b-12854 |
| rs61871615 | T | C | 0.091505 | -0.0194776 | 0.00278196 | 2.50E-12 | 0.111911741 | 49.01952775 | Arm fat percentage (right) | ukb-b-12854 |
| rs61903695 | G | A | 0.2549 | 0.0119507 | 0.00175864 | 1.10E-11 | 0.106112393 | 46.17775285 | Arm fat percentage (right) | ukb-b-12854 |
| rs61983990 | A | G | 0.082492 | 0.0165123 | 0.00279599 | 3.50E-09 | 0.082281778 | 34.87738482 | Arm fat percentage (right) | ukb-b-12854 |
| rs62106258 | C | T | 0.048536 | -0.0574442 | 0.00354996 | 6.80E-59 | 0.402316097 | 261.8457034 | Arm fat percentage (right) | ukb-b-12854 |
| rs62136933 | A | G | 0.184264 | -0.0166104 | 0.00197743 | 4.50E-17 | 0.153537979 | 70.55989805 | Arm fat percentage (right) | ukb-b-12854 |
| rs62190394 | T | C | 0.317035 | 0.0139759 | 0.00164161 | 1.70E-17 | 0.157060323 | 72.48023472 | Arm fat percentage (right) | ukb-b-12854 |
| rs62246311 | A | G | 0.102332 | 0.0161477 | 0.00252092 | 1.50E-10 | 0.095412286 | 41.03016061 | Arm fat percentage (right) | ukb-b-12854 |
| rs62366251 | G | A | 0.231575 | -0.0121163 | 0.00181221 | 2.30E-11 | 0.103069928 | 44.70159204 | Arm fat percentage (right) | ukb-b-12854 |
| rs62413425 | T | C | 0.114067 | 0.0135922 | 0.00242235 | 0.00000002 | 0.074878143 | 31.485147 | Arm fat percentage (right) | ukb-b-12854 |
| rs6497451 | G | T | 0.129665 | -0.0165017 | 0.00231657 | 1.10E-12 | 0.115390138 | 50.74187593 | Arm fat percentage (right) | ukb-b-12854 |
| rs6545714 | A | G | 0.601476 | -0.0149384 | 0.00156151 | 1.10E-21 | 0.190461264 | 91.52055155 | Arm fat percentage (right) | ukb-b-12854 |
| rs6575340 | A | G | 0.636027 | 0.0140823 | 0.00159609 | 1.10E-18 | 0.166747539 | 77.84530611 | Arm fat percentage (right) | ukb-b-12854 |
| rs6669341 | G | A | 0.582712 | -0.0127024 | 0.00154828 | 2.30E-16 | 0.147507333 | 67.30891061 | Arm fat percentage (right) | ukb-b-12854 |
| rs6688826 | C | T | 0.298192 | 0.00980474 | 0.00166689 | 4.10E-09 | 0.081677754 | 34.59858049 | Arm fat percentage (right) | ukb-b-12854 |
| rs6693294 | G | A | 0.688567 | -0.0134572 | 0.00164946 | 3.40E-16 | 0.146109489 | 66.56191948 | Arm fat percentage (right) | ukb-b-12854 |
| rs6699744 | T | A | 0.61605 | 0.014163 | 0.00157711 | 2.70E-19 | 0.171717784 | 80.64668853 | Arm fat percentage (right) | ukb-b-12854 |
| rs6699744 | T | A | 0.61605 | 0.014163 | 0.00157711 | 2.70E-19 | 0.171717784 | 80.64668853 | Arm fat percentage (right) | ukb-b-12854 |
| rs6711390 | T | C | 0.371288 | -0.0100625 | 0.00157066 | 1.50E-10 | 0.095440948 | 41.04378645 | Arm fat percentage (right) | ukb-b-12854 |
| rs6741769 | A | G | 0.305768 | 0.0113497 | 0.00166187 | 8.50E-12 | 0.107064426 | 46.64173191 | Arm fat percentage (right) | ukb-b-12854 |
| rs67609008 | C | T | 0.283586 | 0.0115634 | 0.0017049 | 1.20E-11 | 0.105750489 | 46.00163592 | Arm fat percentage (right) | ukb-b-12854 |
| rs67807996 | A | G | 0.39565 | 0.0156657 | 0.00161097 | 2.40E-22 | 0.195555919 | 94.56375413 | Arm fat percentage (right) | ukb-b-12854 |
| rs6782581 | G | C | 0.439293 | -0.00925154 | 0.00154014 | 1.90E-09 | 0.084885497 | 36.08341731 | Arm fat percentage (right) | ukb-b-12854 |
| rs6840236 | C | T | 0.464869 | 0.0100281 | 0.0015347 | 6.40E-11 | 0.098903575 | 42.69630832 | Arm fat percentage (right) | ukb-b-12854 |
| rs6864305 | C | T | 0.306959 | 0.00913482 | 0.00166401 | 0.00000004 | 0.071900677 | 30.13617467 | Arm fat percentage (right) | ukb-b-12854 |
| rs687621 | G | A | 0.320087 | 0.0113067 | 0.00164133 | 5.60E-12 | 0.108727818 | 47.45477552 | Arm fat percentage (right) | ukb-b-12854 |
| rs6998660 | G | A | 0.452667 | 0.00866517 | 0.00153956 | 1.80E-08 | 0.07530276 | 31.67823214 | Arm fat percentage (right) | ukb-b-12854 |
| rs7027304 | T | C | 0.652642 | 0.0124524 | 0.00161526 | 1.30E-14 | 0.132533154 | 59.43212363 | Arm fat percentage (right) | ukb-b-12854 |
| rs705158 | A | T | 0.244697 | 0.0109257 | 0.0017842 | 9.10E-10 | 0.087921316 | 37.4982908 | Arm fat percentage (right) | ukb-b-12854 |
| rs7107409 | T | C | 0.215341 | -0.0111688 | 0.00187149 | 2.40E-09 | 0.083876877 | 35.61541477 | Arm fat percentage (right) | ukb-b-12854 |
| rs7124681 | A | C | 0.408392 | 0.0236151 | 0.00155343 | 3.40E-52 | 0.37267981 | 231.0980079 | Arm fat percentage (right) | ukb-b-12854 |
| rs7133378 | A | G | 0.31942 | 0.0185267 | 0.00164436 | 1.90E-29 | 0.246038 | 126.9411214 | Arm fat percentage (right) | ukb-b-12854 |
| rs71658797 | A | T | 0.120742 | 0.0243254 | 0.00234892 | 3.90E-25 | 0.216115535 | 107.2465991 | Arm fat percentage (right) | ukb-b-12854 |
| rs7171864 | A | G | 0.66009 | 0.0126503 | 0.00162506 | 7.00E-15 | 0.134783852 | 60.59863598 | Arm fat percentage (right) | ukb-b-12854 |
| rs7187776 | G | A | 0.401993 | 0.0241272 | 0.00156036 | 6.20E-54 | 0.380663538 | 239.0915521 | Arm fat percentage (right) | ukb-b-12854 |
| rs7218014 | C | T | 0.197301 | 0.0174341 | 0.00192879 | 1.60E-19 | 0.173573628 | 81.70133895 | Arm fat percentage (right) | ukb-b-12854 |
| rs7223257 | C | T | 0.336261 | 0.00929424 | 0.00162126 | 9.90E-09 | 0.077902215 | 32.86415195 | Arm fat percentage (right) | ukb-b-12854 |
| rs7228316 | A | C | 0.277883 | 0.00946291 | 0.00171195 | 0.000000032 | 0.072824827 | 30.55394318 | Arm fat percentage (right) | ukb-b-12854 |
| rs723672 | T | C | 0.431522 | 0.00968127 | 0.00155344 | 4.60E-10 | 0.090780931 | 38.83968483 | Arm fat percentage (right) | ukb-b-12854 |
| rs725375 | T | G | 0.232286 | -0.010374 | 0.00181105 | 0.00000001 | 0.077788028 | 32.81191714 | Arm fat percentage (right) | ukb-b-12854 |
| rs725959 | T | G | 0.412477 | -0.00877465 | 0.00156214 | 1.90E-08 | 0.075024055 | 31.55147733 | Arm fat percentage (right) | ukb-b-12854 |
| rs72634826 | A | G | 0.259875 | -0.0141752 | 0.00176602 | 1.00E-15 | 0.142088813 | 64.42688812 | Arm fat percentage (right) | ukb-b-12854 |
| rs72649373 | C | T | 0.143267 | 0.0153857 | 0.00222809 | 5.00E-12 | 0.109194855 | 47.68360274 | Arm fat percentage (right) | ukb-b-12854 |
| rs72767957 | G | A | 0.200136 | -0.0122436 | 0.00191692 | 1.70E-10 | 0.094918007 | 40.79531475 | Arm fat percentage (right) | ukb-b-12854 |
| rs72892910 | T | G | 0.17221 | 0.0244699 | 0.00202888 | 1.70E-33 | 0.272166244 | 145.4627076 | Arm fat percentage (right) | ukb-b-12854 |
| rs72938355 | T | G | 0.553831 | 0.00914348 | 0.0015345 | 2.50E-09 | 0.083638544 | 35.50497842 | Arm fat percentage (right) | ukb-b-12854 |
| rs7321331 | A | G | 0.742306 | 0.0104424 | 0.00176057 | 3.00E-09 | 0.082936178 | 35.17985585 | Arm fat percentage (right) | ukb-b-12854 |
| rs73213484 | T | A | 0.141224 | -0.0157759 | 0.00219681 | 6.90E-13 | 0.117054376 | 51.57073199 | Arm fat percentage (right) | ukb-b-12854 |
| rs7357754 | G | A | 0.500118 | 0.0119384 | 0.00153538 | 7.50E-15 | 0.134514925 | 60.45893513 | Arm fat percentage (right) | ukb-b-12854 |
| rs7422252 | T | C | 0.591449 | 0.0128323 | 0.00155764 | 1.70E-16 | 0.148553413 | 67.86952753 | Arm fat percentage (right) | ukb-b-12854 |
| rs74618095 | C | T | 0.157984 | 0.0115911 | 0.00211627 | 4.30E-08 | 0.071596887 | 29.999026 | Arm fat percentage (right) | ukb-b-12854 |
| rs7463186 | G | A | 0.516029 | 0.00843096 | 0.00153089 | 3.60E-08 | 0.072328692 | 30.32955828 | Arm fat percentage (right) | ukb-b-12854 |
| rs75035127 | G | A | 0.030382 | -0.0267709 | 0.00445224 | 1.80E-09 | 0.085039653 | 36.15503692 | Arm fat percentage (right) | ukb-b-12854 |
| rs7511673 | T | A | 0.392849 | -0.00897132 | 0.00156366 | 9.60E-09 | 0.078019005 | 32.91759083 | Arm fat percentage (right) | ukb-b-12854 |
| rs7516508 | A | T | 0.512553 | -0.0084986 | 0.00154168 | 3.50E-08 | 0.07245861 | 30.38829272 | Arm fat percentage (right) | ukb-b-12854 |
| rs7519259 | A | G | 0.528341 | 0.00958476 | 0.00153673 | 4.50E-10 | 0.090912468 | 38.90158945 | Arm fat percentage (right) | ukb-b-12854 |
| rs75412871 | T | C | 0.051927 | -0.0211978 | 0.00345333 | 8.30E-10 | 0.088308725 | 37.67952485 | Arm fat percentage (right) | ukb-b-12854 |
| rs75499503 | T | C | 0.220141 | -0.0177816 | 0.00187261 | 2.20E-21 | 0.188174258 | 90.1668705 | Arm fat percentage (right) | ukb-b-12854 |
| rs76115890 | C | T | 0.117891 | -0.013558 | 0.00239755 | 0.000000016 | 0.07596196 | 31.97833981 | Arm fat percentage (right) | ukb-b-12854 |
| rs7619139 | A | T | 0.588637 | 0.00937156 | 0.00155702 | 1.80E-09 | 0.085195039 | 36.22725248 | Arm fat percentage (right) | ukb-b-12854 |
| rs7630228 | C | T | 0.4343 | -0.0103314 | 0.00155045 | 2.70E-11 | 0.102449981 | 44.40202961 | Arm fat percentage (right) | ukb-b-12854 |
| rs764729 | C | A | 0.728019 | 0.0100122 | 0.00171841 | 5.70E-09 | 0.0802637 | 33.94731654 | Arm fat percentage (right) | ukb-b-12854 |
| rs7707394 | A | G | 0.35729 | -0.0120754 | 0.00159371 | 3.50E-14 | 0.128602952 | 57.40959143 | Arm fat percentage (right) | ukb-b-12854 |
| rs7730898 | A | G | 0.729112 | 0.0151944 | 0.001721 | 1.10E-18 | 0.166930919 | 77.9480706 | Arm fat percentage (right) | ukb-b-12854 |
| rs7755574 | T | G | 0.282695 | 0.0103986 | 0.00169974 | 9.50E-10 | 0.087768784 | 37.42697747 | Arm fat percentage (right) | ukb-b-12854 |
| rs7774 | A | C | 0.310482 | 0.0109708 | 0.00166561 | 4.50E-11 | 0.100336814 | 43.38403647 | Arm fat percentage (right) | ukb-b-12854 |
| rs7776021 | A | G | 0.287593 | 0.0101577 | 0.00169005 | 1.90E-09 | 0.084972107 | 36.12365264 | Arm fat percentage (right) | ukb-b-12854 |
| rs7796825 | A | G | 0.825769 | 0.0112887 | 0.0020218 | 2.40E-08 | 0.074196069 | 31.17536013 | Arm fat percentage (right) | ukb-b-12854 |
| rs7806643 | T | C | 0.434819 | -0.013363 | 0.00154322 | 4.80E-18 | 0.161603919 | 74.98117653 | Arm fat percentage (right) | ukb-b-12854 |
| rs7824675 | A | C | 0.51942 | -0.0110485 | 0.00153778 | 6.70E-13 | 0.117153156 | 51.62002654 | Arm fat percentage (right) | ukb-b-12854 |
| rs78296744 | A | G | 0.272339 | -0.0119905 | 0.00172185 | 3.30E-12 | 0.110844029 | 48.49354764 | Arm fat percentage (right) | ukb-b-12854 |
| rs7849634 | C | T | 0.188526 | -0.010839 | 0.00195922 | 0.000000032 | 0.072940701 | 30.60638392 | Arm fat percentage (right) | ukb-b-12854 |
| rs7893571 | T | G | 0.665919 | 0.00917057 | 0.00162699 | 0.000000017 | 0.075505345 | 31.77041539 | Arm fat percentage (right) | ukb-b-12854 |
| rs7950748 | T | A | 0.249491 | 0.0114774 | 0.00178326 | 1.20E-10 | 0.096241121 | 41.42454042 | Arm fat percentage (right) | ukb-b-12854 |
| rs79603598 | T | A | 0.079432 | 0.0165588 | 0.00284597 | 5.90E-09 | 0.080058542 | 33.85299412 | Arm fat percentage (right) | ukb-b-12854 |
| rs7966251 | A | G | 0.25502 | -0.00983427 | 0.00175844 | 2.20E-08 | 0.074420609 | 31.27729216 | Arm fat percentage (right) | ukb-b-12854 |
| rs796839 | C | A | 0.615049 | 0.00891608 | 0.001568 | 1.30E-08 | 0.076741374 | 32.33372918 | Arm fat percentage (right) | ukb-b-12854 |
| rs8008772 | T | A | 0.250475 | 0.0106705 | 0.00177546 | 1.90E-09 | 0.084964248 | 36.12000116 | Arm fat percentage (right) | ukb-b-12854 |
| rs8031704 | A | C | 0.327607 | 0.012355 | 0.0016349 | 4.10E-14 | 0.128015428 | 57.10881015 | Arm fat percentage (right) | ukb-b-12854 |
| rs8076669 | C | T | 0.561564 | 0.0088507 | 0.0015451 | 0.00000001 | 0.077789754 | 32.81270663 | Arm fat percentage (right) | ukb-b-12854 |
| rs8090017 | A | G | 0.334473 | 0.0100028 | 0.00163192 | 8.80E-10 | 0.088075427 | 37.57036731 | Arm fat percentage (right) | ukb-b-12854 |
| rs8096658 | G | C | 0.487495 | -0.00949415 | 0.00155149 | 9.40E-10 | 0.087811096 | 37.44675734 | Arm fat percentage (right) | ukb-b-12854 |
| rs811054 | T | C | 0.537266 | 0.0115354 | 0.00154535 | 8.40E-14 | 0.125292444 | 55.72006371 | Arm fat percentage (right) | ukb-b-12854 |
| rs8112818 | G | A | 0.400377 | -0.0147263 | 0.00156856 | 6.10E-21 | 0.184729808 | 88.1424292 | Arm fat percentage (right) | ukb-b-12854 |
| rs8118253 | A | T | 0.131606 | 0.0126024 | 0.00227219 | 2.90E-08 | 0.073284802 | 30.76218869 | Arm fat percentage (right) | ukb-b-12854 |
| rs8132491 | A | G | 0.312987 | -0.010175 | 0.00169727 | 0.000000002 | 0.084574666 | 35.93908086 | Arm fat percentage (right) | ukb-b-12854 |
| rs815163 | C | T | 0.563226 | -0.0101739 | 0.00153833 | 3.80E-11 | 0.101076259 | 43.73971108 | Arm fat percentage (right) | ukb-b-12854 |
| rs879620 | T | C | 0.613272 | 0.0160777 | 0.00157669 | 2.00E-24 | 0.210923406 | 103.9812934 | Arm fat percentage (right) | ukb-b-12854 |
| rs890793 | T | C | 0.385657 | 0.00942413 | 0.00157399 | 2.10E-09 | 0.084380844 | 35.84912822 | Arm fat percentage (right) | ukb-b-12854 |
| rs9304665 | A | T | 0.76364 | 0.0133029 | 0.00180769 | 1.90E-13 | 0.122204819 | 54.15577083 | Arm fat percentage (right) | ukb-b-12854 |
| rs9366863 | C | T | 0.671967 | -0.0186391 | 0.00162671 | 2.10E-30 | 0.252339153 | 131.2893819 | Arm fat percentage (right) | ukb-b-12854 |
| rs9370068 | T | C | 0.517654 | 0.0107734 | 0.00154053 | 2.70E-12 | 0.111682212 | 48.90634976 | Arm fat percentage (right) | ukb-b-12854 |
| rs946185 | G | A | 0.5948 | -0.0105331 | 0.0015693 | 1.90E-11 | 0.1037911 | 45.05058815 | Arm fat percentage (right) | ukb-b-12854 |
| rs9491652 | C | G | 0.496215 | -0.012713 | 0.0015315 | 1.00E-16 | 0.150482153 | 68.90680155 | Arm fat percentage (right) | ukb-b-12854 |
| rs9522183 | T | G | 0.56174 | -0.0102734 | 0.0015541 | 3.80E-11 | 0.100991468 | 43.69889698 | Arm fat percentage (right) | ukb-b-12854 |
| rs9522279 | T | C | 0.423104 | 0.00936879 | 0.00155415 | 1.70E-09 | 0.085436852 | 36.33968373 | Arm fat percentage (right) | ukb-b-12854 |
| rs9536637 | G | T | 0.883926 | -0.015158 | 0.00241627 | 3.50E-10 | 0.091873379 | 39.35436252 | Arm fat percentage (right) | ukb-b-12854 |
| rs9568867 | A | G | 0.129184 | 0.0208511 | 0.0023021 | 1.30E-19 | 0.174162474 | 82.03696264 | Arm fat percentage (right) | ukb-b-12854 |
| rs9579775 | C | A | 0.135543 | 0.0160521 | 0.00232734 | 5.30E-12 | 0.108965488 | 47.5711933 | Arm fat percentage (right) | ukb-b-12854 |
| rs9584855 | G | T | 0.289058 | -0.0124681 | 0.00168828 | 1.50E-13 | 0.122964357 | 54.53955654 | Arm fat percentage (right) | ukb-b-12854 |
| rs972283 | G | A | 0.511931 | -0.0117273 | 0.00152926 | 1.70E-14 | 0.131323352 | 58.80759409 | Arm fat percentage (right) | ukb-b-12854 |
| rs9770544 | G | C | 0.820331 | -0.0176471 | 0.00201105 | 1.70E-18 | 0.165239301 | 77.00181403 | Arm fat percentage (right) | ukb-b-12854 |
| rs9783304 | T | G | 0.689272 | -0.0168153 | 0.00165465 | 2.90E-24 | 0.209791943 | 103.2754159 | Arm fat percentage (right) | ukb-b-12854 |
| rs9816797 | G | A | 0.27738 | 0.0160072 | 0.00170876 | 7.40E-21 | 0.184066159 | 87.75433997 | Arm fat percentage (right) | ukb-b-12854 |
| rs9839081 | A | G | 0.325084 | -0.011285 | 0.00165769 | 9.90E-12 | 0.106454399 | 46.34431759 | Arm fat percentage (right) | ukb-b-12854 |
| rs9843007 | T | A | 0.3735 | -0.0105913 | 0.00157986 | 2.00E-11 | 0.103568747 | 44.94292484 | Arm fat percentage (right) | ukb-b-12854 |
| rs9843653 | C | T | 0.511539 | 0.0169062 | 0.00152954 | 2.10E-28 | 0.239003133 | 122.1716183 | Arm fat percentage (right) | ukb-b-12854 |
| rs9908480 | T | G | 0.454685 | 0.00963724 | 0.0015419 | 4.10E-10 | 0.09126048 | 39.06545907 | Arm fat percentage (right) | ukb-b-12854 |
| rs9951619 | G | T | 0.767275 | 0.00997554 | 0.00182499 | 4.60E-08 | 0.071328695 | 29.87802254 | Arm fat percentage (right) | ukb-b-12854 |
| rs9955276 | T | C | 0.144431 | 0.0149029 | 0.00219034 | 1.00E-11 | 0.106349753 | 46.29333897 | Arm fat percentage (right) | ukb-b-12854 |
| rs9968060 | T | C | 0.642607 | 0.0112547 | 0.00162439 | 4.30E-12 | 0.109850172 | 48.00508376 | Arm fat percentage (right) | ukb-b-12854 |

Supplementary Table 12: Information on all instrumental variables (IVs) ultimately used for Leg fat percentage (left) in our study

| SNPs | effect_allele.exposure | other_allele.exposure | eaf.exposure | beta.exposure | se.exposure | pval.exposure | R^2^ | F | trait | id.exposure |
| --- | --- | --- | --- | --- | --- | --- | --- | --- | --- | --- |
| rs1005551 | T | C | 0.408067 | -0.00956592 | 0.00128293 | 8.90E-14 | 0.127340721 | 55.59651478 | Leg fat percentage (left) | ukb-b-18377 |
| rs10100245 | A | G | 0.56447 | 0.0112458 | 0.00127228 | 9.70E-19 | 0.170168851 | 78.1295478 | Leg fat percentage (left) | ukb-b-18377 |
| rs10119967 | C | A | 0.204095 | -0.0101015 | 0.00156656 | 1.10E-10 | 0.098394178 | 41.57934752 | Leg fat percentage (left) | ukb-b-18377 |
| rs1013293 | A | G | 0.430271 | -0.0100242 | 0.00127435 | 3.70E-15 | 0.139713987 | 61.87596704 | Leg fat percentage (left) | ukb-b-18377 |
| rs10144067 | T | C | 0.591285 | 0.00792623 | 0.00129321 | 8.80E-10 | 0.089749339 | 37.56602406 | Leg fat percentage (left) | ukb-b-18377 |
| rs10185199 | A | G | 0.28081 | -0.008515 | 0.0014367 | 3.10E-09 | 0.084413432 | 35.12668135 | Leg fat percentage (left) | ukb-b-18377 |
| rs10203386 | A | T | 0.451928 | 0.0185319 | 0.00126282 | 9.30E-49 | 0.361119877 | 215.3560085 | Leg fat percentage (left) | ukb-b-18377 |
| rs10209821 | T | C | 0.342619 | 0.0106446 | 0.00132327 | 8.70E-16 | 0.145181333 | 64.70856356 | Leg fat percentage (left) | ukb-b-18377 |
| rs10404726 | T | C | 0.46553 | -0.0112769 | 0.00126669 | 5.50E-19 | 0.172201994 | 79.25720895 | Leg fat percentage (left) | ukb-b-18377 |
| rs10423928 | A | T | 0.19443 | -0.0199402 | 0.0015918 | 5.30E-36 | 0.29171801 | 156.921344 | Leg fat percentage (left) | ukb-b-18377 |
| rs10499014 | G | C | 0.268659 | -0.0102912 | 0.00142939 | 6.00E-13 | 0.119758761 | 51.8358898 | Leg fat percentage (left) | ukb-b-18377 |
| rs10505836 | C | A | 0.859995 | 0.0124316 | 0.00182952 | 1.10E-11 | 0.108087841 | 46.17211117 | Leg fat percentage (left) | ukb-b-18377 |
| rs10514963 | A | G | 0.480665 | 0.0100347 | 0.00126032 | 1.70E-15 | 0.142652402 | 63.39385034 | Leg fat percentage (left) | ukb-b-18377 |
| rs10516044 | C | G | 0.115988 | 0.0110487 | 0.00197693 | 2.30E-08 | 0.075769609 | 31.23487551 | Leg fat percentage (left) | ukb-b-18377 |
| rs10756792 | T | C | 0.743078 | -0.0126581 | 0.00144853 | 2.40E-18 | 0.166963363 | 76.36283728 | Leg fat percentage (left) | ukb-b-18377 |
| rs10765273 | T | C | 0.697695 | 0.00827317 | 0.00137292 | 1.70E-09 | 0.08701463 | 36.3122729 | Leg fat percentage (left) | ukb-b-18377 |
| rs10822155 | A | C | 0.416311 | -0.00716725 | 0.00127889 | 0.000000021 | 0.076157339 | 31.40788728 | Leg fat percentage (left) | ukb-b-18377 |
| rs10823242 | A | G | 0.574817 | -0.00899728 | 0.00127839 | 2.00E-12 | 0.115050675 | 49.53312667 | Leg fat percentage (left) | ukb-b-18377 |
| rs10854853 | T | G | 0.456803 | 0.00801821 | 0.00126731 | 2.50E-10 | 0.095077061 | 40.03032621 | Leg fat percentage (left) | ukb-b-18377 |
| rs10883026 | T | C | 0.521466 | -0.00995704 | 0.001271 | 4.70E-15 | 0.138733676 | 61.37187651 | Leg fat percentage (left) | ukb-b-18377 |
| rs10885421 | T | G | 0.59824 | -0.00800276 | 0.00129234 | 5.90E-10 | 0.091443526 | 38.34652485 | Leg fat percentage (left) | ukb-b-18377 |
| rs10887578 | C | G | 0.497529 | 0.00812121 | 0.00126758 | 1.50E-10 | 0.097258821 | 41.04787933 | Leg fat percentage (left) | ukb-b-18377 |
| rs10896012 | C | T | 0.217391 | 0.0116149 | 0.00153166 | 3.40E-14 | 0.131138913 | 57.50507956 | Leg fat percentage (left) | ukb-b-18377 |
| rs10938397 | G | A | 0.434306 | 0.0153799 | 0.00127219 | 1.20E-33 | 0.277247526 | 146.1514298 | Leg fat percentage (left) | ukb-b-18377 |
| rs10951992 | C | T | 0.439094 | 0.00778163 | 0.00128567 | 1.40E-09 | 0.087717547 | 36.63381383 | Leg fat percentage (left) | ukb-b-18377 |
| rs10960293 | T | G | 0.333748 | -0.00732502 | 0.00133994 | 4.60E-08 | 0.072732263 | 29.88456412 | Leg fat percentage (left) | ukb-b-18377 |
| rs10973836 | A | T | 0.469083 | 0.00730852 | 0.0012693 | 8.50E-09 | 0.080051396 | 33.15357145 | Leg fat percentage (left) | ukb-b-18377 |
| rs10982884 | T | C | 0.093726 | 0.0137671 | 0.00216895 | 2.20E-10 | 0.095632563 | 40.28894109 | Leg fat percentage (left) | ukb-b-18377 |
| rs10985968 | G | C | 0.501373 | -0.00731337 | 0.001265 | 7.40E-09 | 0.080650952 | 33.42366277 | Leg fat percentage (left) | ukb-b-18377 |
| rs10989067 | A | G | 0.315884 | 0.00804178 | 0.0013536 | 2.80E-09 | 0.084785503 | 35.29585319 | Leg fat percentage (left) | ukb-b-18377 |
| rs10992854 | C | T | 0.681936 | -0.0102067 | 0.00135886 | 5.90E-14 | 0.12898053 | 56.41846559 | Leg fat percentage (left) | ukb-b-18377 |
| rs10997982 | A | G | 0.493184 | -0.00867629 | 0.00125998 | 5.70E-12 | 0.110681076 | 47.4177362 | Leg fat percentage (left) | ukb-b-18377 |
| rs10999460 | T | C | 0.265498 | 0.011352 | 0.00143334 | 2.40E-15 | 0.141361657 | 62.7258165 | Leg fat percentage (left) | ukb-b-18377 |
| rs11012732 | G | A | 0.331713 | 0.0144347 | 0.00133886 | 4.20E-27 | 0.233766119 | 116.2372137 | Leg fat percentage (left) | ukb-b-18377 |
| rs11062590 | G | C | 0.093176 | -0.0123685 | 0.00218928 | 0.000000016 | 0.077297947 | 31.91768965 | Leg fat percentage (left) | ukb-b-18377 |
| rs11122450 | G | T | 0.611696 | -0.00785008 | 0.00129129 | 1.20E-09 | 0.088423692 | 36.95733016 | Leg fat percentage (left) | ukb-b-18377 |
| rs11150745 | G | A | 0.317697 | -0.0101476 | 0.0013568 | 7.50E-14 | 0.128019675 | 55.93646408 | Leg fat percentage (left) | ukb-b-18377 |
| rs11162976 | C | A | 0.299407 | 0.00811026 | 0.00137144 | 3.30E-09 | 0.084072162 | 34.97163443 | Leg fat percentage (left) | ukb-b-18377 |
| rs11172114 | T | C | 0.353397 | 0.00725588 | 0.00132098 | 0.00000004 | 0.073377915 | 30.17086062 | Leg fat percentage (left) | ukb-b-18377 |
| rs11208779 | C | G | 0.528886 | 0.00806433 | 0.00126271 | 1.70E-10 | 0.096701992 | 40.78771187 | Leg fat percentage (left) | ukb-b-18377 |
| rs11245453 | A | G | 0.282657 | 0.00867836 | 0.00140155 | 5.90E-10 | 0.091430541 | 38.34053177 | Leg fat percentage (left) | ukb-b-18377 |
| rs112852122 | A | G | 0.157849 | -0.0148948 | 0.00175761 | 2.40E-17 | 0.158599592 | 71.81651433 | Leg fat percentage (left) | ukb-b-18377 |
| rs113963976 | T | C | 0.324826 | 0.0077101 | 0.00135026 | 1.10E-08 | 0.078831421 | 32.60507602 | Leg fat percentage (left) | ukb-b-18377 |
| rs114295766 | T | A | 0.069525 | -0.0162208 | 0.00262819 | 6.80E-10 | 0.090891213 | 38.09175848 | Leg fat percentage (left) | ukb-b-18377 |
| rs11563357 | T | A | 0.188731 | 0.00931679 | 0.00161423 | 7.80E-09 | 0.080403358 | 33.3120826 | Leg fat percentage (left) | ukb-b-18377 |
| rs11656076 | A | G | 0.224675 | -0.00837591 | 0.00151064 | 2.90E-08 | 0.074664839 | 30.74270262 | Leg fat percentage (left) | ukb-b-18377 |
| rs11664106 | T | A | 0.373941 | 0.00802152 | 0.00133603 | 1.90E-09 | 0.086436048 | 36.04797897 | Leg fat percentage (left) | ukb-b-18377 |
| rs11691869 | A | C | 0.361918 | -0.0127029 | 0.00131143 | 3.40E-22 | 0.197598004 | 93.82434256 | Leg fat percentage (left) | ukb-b-18377 |
| rs117068593 | T | C | 0.189443 | -0.0124204 | 0.00161502 | 1.50E-14 | 0.134375453 | 59.14463491 | Leg fat percentage (left) | ukb-b-18377 |
| rs117342986 | T | C | 0.026446 | 0.0227999 | 0.00412271 | 0.000000032 | 0.074308994 | 30.58442437 | Leg fat percentage (left) | ukb-b-18377 |
| rs11777625 | C | T | 0.545834 | 0.00728038 | 0.00126587 | 8.90E-09 | 0.079881916 | 33.07728694 | Leg fat percentage (left) | ukb-b-18377 |
| rs11782074 | T | G | 0.383638 | 0.00831416 | 0.00131708 | 2.70E-10 | 0.094686185 | 39.84854292 | Leg fat percentage (left) | ukb-b-18377 |
| rs11789898 | T | G | 0.167117 | -0.0101631 | 0.00169265 | 1.90E-09 | 0.086442802 | 36.05106231 | Leg fat percentage (left) | ukb-b-18377 |
| rs11841757 | G | A | 0.239515 | -0.01169 | 0.00147787 | 2.60E-15 | 0.141057358 | 62.56861703 | Leg fat percentage (left) | ukb-b-18377 |
| rs11866219 | C | A | 0.583797 | -0.0110066 | 0.00129572 | 2.00E-17 | 0.159233508 | 72.15792612 | Leg fat percentage (left) | ukb-b-18377 |
| rs1188162 | G | A | 0.526999 | 0.00754315 | 0.00126668 | 2.60E-09 | 0.085152139 | 35.46268882 | Leg fat percentage (left) | ukb-b-18377 |
| rs12042959 | G | A | 0.14393 | -0.0118125 | 0.00180103 | 5.40E-11 | 0.101451463 | 43.01716141 | Leg fat percentage (left) | ukb-b-18377 |
| rs12055234 | A | G | 0.328065 | 0.00835509 | 0.00134209 | 4.80E-10 | 0.092329837 | 38.75600328 | Leg fat percentage (left) | ukb-b-18377 |
| rs12071132 | G | C | 0.147802 | 0.00989735 | 0.00178101 | 2.70E-08 | 0.074977741 | 30.88198026 | Leg fat percentage (left) | ukb-b-18377 |
| rs12072739 | G | A | 0.224587 | 0.0100367 | 0.00150946 | 2.90E-11 | 0.103976049 | 44.21184802 | Leg fat percentage (left) | ukb-b-18377 |
| rs12122664 | C | A | 0.315978 | 0.00758472 | 0.00135651 | 2.30E-08 | 0.075833057 | 31.26317678 | Leg fat percentage (left) | ukb-b-18377 |
| rs12124126 | G | A | 0.334232 | -0.0117202 | 0.0013345 | 1.60E-18 | 0.168361408 | 77.1316975 | Leg fat percentage (left) | ukb-b-18377 |
| rs12156460 | T | A | 0.537606 | -0.00710492 | 0.00127994 | 2.80E-08 | 0.074823621 | 30.81336733 | Leg fat percentage (left) | ukb-b-18377 |
| rs12197124 | T | C | 0.217869 | -0.0131904 | 0.0015221 | 4.50E-18 | 0.164653587 | 75.09820552 | Leg fat percentage (left) | ukb-b-18377 |
| rs1229984 | C | T | 0.97278 | 0.0243428 | 0.00382207 | 1.90E-10 | 0.096223241 | 40.56428155 | Leg fat percentage (left) | ukb-b-18377 |
| rs12316080 | T | C | 0.335378 | 0.0104969 | 0.00133338 | 3.50E-15 | 0.139905681 | 61.97467335 | Leg fat percentage (left) | ukb-b-18377 |
| rs12320285 | T | G | 0.32358 | 0.00801153 | 0.00134701 | 2.70E-09 | 0.08495819 | 35.37441666 | Leg fat percentage (left) | ukb-b-18377 |
| rs12375196 | A | C | 0.424286 | 0.00889462 | 0.0012825 | 4.10E-12 | 0.112093931 | 48.09944329 | Leg fat percentage (left) | ukb-b-18377 |
| rs12432026 | G | T | 0.540103 | 0.00892737 | 0.00126372 | 1.60E-12 | 0.115814695 | 49.90514845 | Leg fat percentage (left) | ukb-b-18377 |
| rs12462975 | A | G | 0.329602 | 0.00864292 | 0.00135133 | 1.60E-10 | 0.096957491 | 40.90704886 | Leg fat percentage (left) | ukb-b-18377 |
| rs12475388 | A | G | 0.48571 | -0.00704443 | 0.00126434 | 0.000000025 | 0.075339268 | 31.04301966 | Leg fat percentage (left) | ukb-b-18377 |
| rs1250603 | G | A | 0.663437 | 0.00781318 | 0.00133827 | 5.30E-09 | 0.082116557 | 34.08538269 | Leg fat percentage (left) | ukb-b-18377 |
| rs12538435 | G | A | 0.261843 | -0.0086377 | 0.00143296 | 1.70E-09 | 0.087064886 | 36.33524538 | Leg fat percentage (left) | ukb-b-18377 |
| rs12583872 | C | A | 0.309026 | -0.00952346 | 0.00136488 | 3.00E-12 | 0.113305183 | 48.68560645 | Leg fat percentage (left) | ukb-b-18377 |
| rs12642970 | C | G | 0.441526 | -0.00758437 | 0.00127397 | 2.60E-09 | 0.08510712 | 35.44219585 | Leg fat percentage (left) | ukb-b-18377 |
| rs12679106 | T | G | 0.70917 | -0.0131841 | 0.00139416 | 3.20E-21 | 0.190100017 | 89.42845762 | Leg fat percentage (left) | ukb-b-18377 |
| rs12724928 | C | T | 0.204876 | -0.00987499 | 0.0015573 | 2.30E-10 | 0.095462015 | 40.20950825 | Leg fat percentage (left) | ukb-b-18377 |
| rs1274232 | G | A | 0.912556 | 0.0123178 | 0.00223424 | 3.50E-08 | 0.073883474 | 30.39531518 | Leg fat percentage (left) | ukb-b-18377 |
| rs12788343 | C | T | 0.409209 | 0.00915499 | 0.00128043 | 8.70E-13 | 0.118303648 | 51.1215563 | Leg fat percentage (left) | ukb-b-18377 |
| rs12806052 | T | C | 0.163632 | -0.0117635 | 0.00170585 | 5.40E-12 | 0.110964841 | 47.55447966 | Leg fat percentage (left) | ukb-b-18377 |
| rs12890931 | G | T | 0.362431 | 0.00949822 | 0.00132004 | 6.20E-13 | 0.119632528 | 51.77382677 | Leg fat percentage (left) | ukb-b-18377 |
| rs1296685 | G | A | 0.211087 | 0.0104085 | 0.00156006 | 2.50E-11 | 0.104611672 | 44.51369951 | Leg fat percentage (left) | ukb-b-18377 |
| rs12986231 | C | T | 0.268008 | -0.00994593 | 0.00143933 | 4.80E-12 | 0.111369503 | 47.7496334 | Leg fat percentage (left) | ukb-b-18377 |
| rs13068928 | A | G | 0.191312 | -0.00959486 | 0.00159871 | 0.000000002 | 0.086373699 | 36.01951838 | Leg fat percentage (left) | ukb-b-18377 |
| rs13107325 | T | C | 0.074862 | 0.0242976 | 0.00239492 | 3.50E-24 | 0.212697116 | 102.9306546 | Leg fat percentage (left) | ukb-b-18377 |
| rs13132853 | G | A | 0.360215 | -0.00744086 | 0.00131727 | 0.000000016 | 0.077275815 | 31.90778554 | Leg fat percentage (left) | ukb-b-18377 |
| rs13177679 | T | C | 0.340433 | -0.00869624 | 0.0013586 | 1.50E-10 | 0.097095006 | 40.97130654 | Leg fat percentage (left) | ukb-b-18377 |
| rs1322842 | G | A | 0.609487 | -0.00899295 | 0.00129774 | 4.20E-12 | 0.111931075 | 48.02075407 | Leg fat percentage (left) | ukb-b-18377 |
| rs13249935 | C | T | 0.366659 | 0.00741035 | 0.00131944 | 0.00000002 | 0.07645912 | 31.542648 | Leg fat percentage (left) | ukb-b-18377 |
| rs13320698 | T | C | 0.239486 | -0.00824511 | 0.00147483 | 2.30E-08 | 0.075813029 | 31.25424281 | Leg fat percentage (left) | ukb-b-18377 |
| rs13389219 | T | C | 0.392421 | 0.0100714 | 0.00128604 | 4.80E-15 | 0.138651525 | 61.32968532 | Leg fat percentage (left) | ukb-b-18377 |
| rs13427822 | G | A | 0.271195 | -0.00972628 | 0.00142992 | 1.00E-11 | 0.108285634 | 46.26686303 | Leg fat percentage (left) | ukb-b-18377 |
| rs136309 | A | C | 0.205652 | 0.00951115 | 0.00156556 | 1.20E-09 | 0.088317223 | 36.90851984 | Leg fat percentage (left) | ukb-b-18377 |
| rs1377184 | T | A | 0.748948 | 0.00850242 | 0.00145628 | 5.30E-09 | 0.082121272 | 34.08751471 | Leg fat percentage (left) | ukb-b-18377 |
| rs1394092 | T | C | 0.72684 | 0.0105207 | 0.00141264 | 9.50E-14 | 0.127079626 | 55.46592686 | Leg fat percentage (left) | ukb-b-18377 |
| rs1428 | T | C | 0.098042 | -0.012417 | 0.00224056 | 3.00E-08 | 0.074597758 | 30.71285614 | Leg fat percentage (left) | ukb-b-18377 |
| rs1428 | T | C | 0.098042 | -0.012417 | 0.00224056 | 3.00E-08 | 0.074597758 | 30.71285614 | Leg fat percentage (left) | ukb-b-18377 |
| rs143453062 | G | A | 0.056813 | -0.018703 | 0.002717 | 5.80E-12 | 0.110613657 | 47.38526032 | Leg fat percentage (left) | ukb-b-18377 |
| rs1441264 | A | G | 0.593691 | 0.00954366 | 0.00131169 | 3.40E-13 | 0.12199438 | 52.9379967 | Leg fat percentage (left) | ukb-b-18377 |
| rs145828988 | C | T | 0.053198 | -0.0175862 | 0.00288592 | 1.10E-09 | 0.088809541 | 37.13431695 | Leg fat percentage (left) | ukb-b-18377 |
| rs1471093 | A | G | 0.616762 | 0.00758938 | 0.00130108 | 5.40E-09 | 0.081984133 | 34.0255062 | Leg fat percentage (left) | ukb-b-18377 |
| rs1475535 | A | T | 0.498077 | 0.0090974 | 0.00126448 | 6.30E-13 | 0.119608372 | 51.76195272 | Leg fat percentage (left) | ukb-b-18377 |
| rs147730268 | T | G | 0.087268 | -0.0200251 | 0.00228355 | 1.80E-18 | 0.167941241 | 76.90035356 | Leg fat percentage (left) | ukb-b-18377 |
| rs1497981 | G | C | 0.424712 | 0.00712464 | 0.001285 | 2.90E-08 | 0.074661268 | 30.74111349 | Leg fat percentage (left) | ukb-b-18377 |
| rs1504369 | A | C | 0.70108 | -0.00777695 | 0.00138473 | 0.00000002 | 0.076457537 | 31.54194071 | Leg fat percentage (left) | ukb-b-18377 |
| rs1512065 | G | A | 0.769916 | -0.00865754 | 0.00152612 | 1.40E-08 | 0.077887966 | 32.18189739 | Leg fat percentage (left) | ukb-b-18377 |
| rs1520455 | T | C | 0.612401 | -0.00884566 | 0.00131194 | 1.60E-11 | 0.10659919 | 45.46032516 | Leg fat percentage (left) | ukb-b-18377 |
| rs1554654 | T | C | 0.470559 | -0.00979463 | 0.00126368 | 9.10E-15 | 0.136203511 | 60.07611577 | Leg fat percentage (left) | ukb-b-18377 |
| rs1568489 | G | A | 0.573044 | 0.00877587 | 0.00127618 | 6.10E-12 | 0.11041294 | 47.28860408 | Leg fat percentage (left) | ukb-b-18377 |
| rs156914 | A | G | 0.491812 | 0.00745426 | 0.00125863 | 3.20E-09 | 0.084302414 | 35.07623047 | Leg fat percentage (left) | ukb-b-18377 |
| rs1618725 | T | C | 0.49403 | -0.012071 | 0.0012625 | 1.20E-21 | 0.19350796 | 91.41631824 | Leg fat percentage (left) | ukb-b-18377 |
| rs16871902 | A | G | 0.486595 | 0.00688834 | 0.00125957 | 4.50E-08 | 0.072784708 | 29.90780456 | Leg fat percentage (left) | ukb-b-18377 |
| rs16916303 | G | A | 0.119775 | -0.0108838 | 0.00196229 | 2.90E-08 | 0.074711414 | 30.76342773 | Leg fat percentage (left) | ukb-b-18377 |
| rs16996657 | C | T | 0.127758 | 0.0107988 | 0.00189515 | 0.000000012 | 0.078527408 | 32.46861893 | Leg fat percentage (left) | ukb-b-18377 |
| rs17024393 | C | T | 0.025905 | 0.0309681 | 0.00397117 | 6.30E-15 | 0.137643041 | 60.8124028 | Leg fat percentage (left) | ukb-b-18377 |
| rs17056301 | C | T | 0.256497 | 0.00912241 | 0.00144705 | 2.90E-10 | 0.094457404 | 39.74221785 | Leg fat percentage (left) | ukb-b-18377 |
| rs17120131 | C | T | 0.060193 | 0.0187801 | 0.00265273 | 1.40E-12 | 0.116255013 | 50.11984314 | Leg fat percentage (left) | ukb-b-18377 |
| rs1724556 | A | C | 0.58663 | -0.00908954 | 0.00128557 | 1.50E-12 | 0.115990946 | 49.99106074 | Leg fat percentage (left) | ukb-b-18377 |
| rs1724729 | C | T | 0.568217 | -0.00906479 | 0.00126871 | 9.00E-13 | 0.118156426 | 51.04941491 | Leg fat percentage (left) | ukb-b-18377 |
| rs17446091 | C | T | 0.201818 | 0.0107947 | 0.00157076 | 6.30E-12 | 0.11028748 | 47.22821024 | Leg fat percentage (left) | ukb-b-18377 |
| rs17522122 | T | G | 0.471218 | 0.00946488 | 0.00126804 | 8.40E-14 | 0.127575478 | 55.71399711 | Leg fat percentage (left) | ukb-b-18377 |
| rs17639996 | A | G | 0.150085 | -0.012824 | 0.00176991 | 4.30E-13 | 0.121103676 | 52.49822896 | Leg fat percentage (left) | ukb-b-18377 |
| rs17770336 | T | C | 0.322453 | 0.012308 | 0.00134559 | 5.90E-20 | 0.180056371 | 83.66608998 | Leg fat percentage (left) | ukb-b-18377 |
| rs1778830 | A | G | 0.362116 | 0.00839789 | 0.00131147 | 1.50E-10 | 0.097164434 | 41.00375618 | Leg fat percentage (left) | ukb-b-18377 |
| rs17820064 | C | T | 0.295297 | 0.00894811 | 0.00138044 | 9.00E-11 | 0.099327496 | 42.01724359 | Leg fat percentage (left) | ukb-b-18377 |
| rs1782508 | G | C | 0.655506 | -0.00741301 | 0.00132442 | 2.20E-08 | 0.075979162 | 31.32836364 | Leg fat percentage (left) | ukb-b-18377 |
| rs1801282 | G | C | 0.119541 | 0.0218855 | 0.00193563 | 1.20E-29 | 0.251238758 | 127.8404401 | Leg fat percentage (left) | ukb-b-18377 |
| rs1861410 | T | C | 0.555369 | -0.0104763 | 0.00126899 | 1.50E-16 | 0.151741004 | 68.15527168 | Leg fat percentage (left) | ukb-b-18377 |
| rs1928496 | T | C | 0.743608 | 0.00968309 | 0.00144318 | 2.00E-11 | 0.105671752 | 45.01807637 | Leg fat percentage (left) | ukb-b-18377 |
| rs1945160 | A | G | 0.375863 | -0.00809202 | 0.00130769 | 6.10E-10 | 0.091324658 | 38.29166843 | Leg fat percentage (left) | ukb-b-18377 |
| rs197374 | T | C | 0.38065 | 0.00710736 | 0.00129473 | 0.00000004 | 0.07329506 | 30.13409863 | Leg fat percentage (left) | ukb-b-18377 |
| rs1974991 | G | C | 0.500672 | -0.0079872 | 0.00126083 | 2.40E-10 | 0.095292608 | 40.13063678 | Leg fat percentage (left) | ukb-b-18377 |
| rs1981612 | A | C | 0.455996 | 0.00732491 | 0.00128004 | 1.10E-08 | 0.07914505 | 32.74594316 | Leg fat percentage (left) | ukb-b-18377 |
| rs2008012 | T | C | 0.190158 | 0.00948706 | 0.00161003 | 3.80E-09 | 0.083520509 | 34.72125047 | Leg fat percentage (left) | ukb-b-18377 |
| rs2016962 | C | G | 0.037466 | 0.0199331 | 0.00347377 | 9.60E-09 | 0.079547069 | 32.92665177 | Leg fat percentage (left) | ukb-b-18377 |
| rs2034768 | G | A | 0.512163 | -0.0104935 | 0.00125835 | 7.50E-17 | 0.154349059 | 69.54050286 | Leg fat percentage (left) | ukb-b-18377 |
| rs2035936 | T | G | 0.055878 | 0.0204246 | 0.00277985 | 2.00E-13 | 0.124105548 | 53.98391731 | Leg fat percentage (left) | ukb-b-18377 |
| rs2045293 | T | C | 0.419167 | 0.00797304 | 0.00128185 | 5.00E-10 | 0.092182223 | 38.68774979 | Leg fat percentage (left) | ukb-b-18377 |
| rs2094260 | A | G | 0.728224 | 0.00817735 | 0.00141857 | 8.20E-09 | 0.080219993 | 33.22948654 | Leg fat percentage (left) | ukb-b-18377 |
| rs2111281 | C | A | 0.366944 | 0.00763572 | 0.00130847 | 5.40E-09 | 0.082047904 | 34.05433857 | Leg fat percentage (left) | ukb-b-18377 |
| rs215669 | A | G | 0.611673 | -0.00999061 | 0.00129831 | 1.40E-14 | 0.134512604 | 59.21438287 | Leg fat percentage (left) | ukb-b-18377 |
| rs2172131 | C | T | 0.578566 | -0.00982025 | 0.00127698 | 1.50E-14 | 0.134365153 | 59.1393977 | Leg fat percentage (left) | ukb-b-18377 |
| rs217672 | C | A | 0.271728 | 0.00936101 | 0.00142129 | 4.50E-11 | 0.102217695 | 43.37904808 | Leg fat percentage (left) | ukb-b-18377 |
| rs2192649 | G | T | 0.500791 | 0.00725672 | 0.00126775 | 0.00000001 | 0.079188019 | 32.7652506 | Leg fat percentage (left) | ukb-b-18377 |
| rs2216931 | A | C | 0.661952 | 0.00984013 | 0.00133077 | 1.40E-13 | 0.125496659 | 54.67586573 | Leg fat percentage (left) | ukb-b-18377 |
| rs2254614 | C | T | 0.339234 | 0.0119593 | 0.00132616 | 1.90E-19 | 0.175902928 | 81.32417603 | Leg fat percentage (left) | ukb-b-18377 |
| rs2274224 | C | G | 0.435466 | -0.0118807 | 0.00127067 | 8.80E-21 | 0.186630078 | 87.42155078 | Leg fat percentage (left) | ukb-b-18377 |
| rs2289379 | T | C | 0.395692 | -0.00911378 | 0.00129372 | 1.90E-12 | 0.115243229 | 49.62682574 | Leg fat percentage (left) | ukb-b-18377 |
| rs2307111 | C | T | 0.395096 | -0.0137922 | 0.0012892 | 1.00E-26 | 0.231006497 | 114.4528203 | Leg fat percentage (left) | ukb-b-18377 |
| rs2326844 | C | G | 0.634235 | 0.00752181 | 0.00131177 | 9.80E-09 | 0.079442899 | 32.87981226 | Leg fat percentage (left) | ukb-b-18377 |
| rs2339683 | C | T | 0.868944 | 0.0102706 | 0.00187627 | 4.40E-08 | 0.072911679 | 29.96408095 | Leg fat percentage (left) | ukb-b-18377 |
| rs2395400 | A | G | 0.230788 | -0.00939826 | 0.00149786 | 3.50E-10 | 0.093653058 | 39.36882604 | Leg fat percentage (left) | ukb-b-18377 |
| rs241459 | C | A | 0.680974 | -0.0110299 | 0.00134682 | 2.60E-16 | 0.149685181 | 67.06934054 | Leg fat percentage (left) | ukb-b-18377 |
| rs2433733 | A | G | 0.677734 | -0.0120907 | 0.00134557 | 2.60E-19 | 0.174860827 | 80.74028892 | Leg fat percentage (left) | ukb-b-18377 |
| rs2450444 | A | G | 0.348837 | -0.00726185 | 0.00132105 | 3.90E-08 | 0.07348262 | 30.21732649 | Leg fat percentage (left) | ukb-b-18377 |
| rs2456529 | T | C | 0.144487 | 0.00983145 | 0.00180099 | 4.80E-08 | 0.072540807 | 29.79974494 | Leg fat percentage (left) | ukb-b-18377 |
| rs255757 | A | G | 0.252058 | -0.00813616 | 0.00145748 | 2.40E-08 | 0.07560755 | 31.16260488 | Leg fat percentage (left) | ukb-b-18377 |
| rs2605091 | G | C | 0.68233 | -0.0119871 | 0.001349 | 6.30E-19 | 0.171666191 | 78.95949415 | Leg fat percentage (left) | ukb-b-18377 |
| rs2613498 | T | C | 0.191135 | -0.0178659 | 0.00159649 | 4.50E-29 | 0.247381538 | 125.2325986 | Leg fat percentage (left) | ukb-b-18377 |
| rs262956 | G | T | 0.646883 | -0.00723271 | 0.00132081 | 4.40E-08 | 0.07296157 | 29.98619822 | Leg fat percentage (left) | ukb-b-18377 |
| rs264959 | T | C | 0.45978 | -0.00719262 | 0.00125973 | 1.10E-08 | 0.078820398 | 32.60012674 | Leg fat percentage (left) | ukb-b-18377 |
| rs2656512 | T | C | 0.654555 | 0.00740305 | 0.00132615 | 2.40E-08 | 0.075607922 | 31.16277083 | Leg fat percentage (left) | ukb-b-18377 |
| rs2660241 | C | T | 0.364839 | 0.0079128 | 0.00131141 | 1.60E-09 | 0.08722155 | 36.40687442 | Leg fat percentage (left) | ukb-b-18377 |
| rs2670556 | G | C | 0.52473 | -0.00751447 | 0.00126305 | 2.70E-09 | 0.085005877 | 35.39611713 | Leg fat percentage (left) | ukb-b-18377 |
| rs2721965 | C | A | 0.333806 | -0.0108868 | 0.00133691 | 3.80E-16 | 0.148246688 | 66.31261366 | Leg fat percentage (left) | ukb-b-18377 |
| rs2722217 | A | G | 0.480528 | -0.00752633 | 0.001267 | 2.80E-09 | 0.084765779 | 35.28688184 | Leg fat percentage (left) | ukb-b-18377 |
| rs2725370 | C | T | 0.696118 | -0.0111538 | 0.00137521 | 5.00E-16 | 0.147235287 | 65.78208919 | Leg fat percentage (left) | ukb-b-18377 |
| rs2727340 | A | T | 0.331182 | 0.00945636 | 0.00132926 | 1.10E-12 | 0.117256678 | 50.60904267 | Leg fat percentage (left) | ukb-b-18377 |
| rs2733296 | G | A | 0.47865 | 0.00911873 | 0.00126566 | 5.80E-13 | 0.119905459 | 51.90803678 | Leg fat percentage (left) | ukb-b-18377 |
| rs2794665 | G | A | 0.659429 | 0.00727434 | 0.00133357 | 4.90E-08 | 0.072439095 | 29.75469876 | Leg fat percentage (left) | ukb-b-18377 |
| rs2798297 | A | G | 0.366713 | 0.008791 | 0.00131704 | 2.50E-11 | 0.104694724 | 44.55317203 | Leg fat percentage (left) | ukb-b-18377 |
| rs2802295 | G | A | 0.626185 | 0.0094046 | 0.00130203 | 5.10E-13 | 0.120442055 | 52.17214295 | Leg fat percentage (left) | ukb-b-18377 |
| rs2836935 | G | T | 0.161264 | 0.00944248 | 0.0017146 | 3.60E-08 | 0.073732343 | 30.32819114 | Leg fat percentage (left) | ukb-b-18377 |
| rs28457808 | G | C | 0.163079 | -0.0125605 | 0.00170933 | 2.00E-13 | 0.124129987 | 53.99605437 | Leg fat percentage (left) | ukb-b-18377 |
| rs2861685 | C | T | 0.412113 | -0.00908361 | 0.00127442 | 1.00E-12 | 0.117653773 | 50.80328588 | Leg fat percentage (left) | ukb-b-18377 |
| rs2865383 | C | T | 0.374465 | 0.00843806 | 0.00130192 | 9.10E-11 | 0.099304646 | 42.00651195 | Leg fat percentage (left) | ukb-b-18377 |
| rs28711392 | C | T | 0.36736 | -0.00927038 | 0.00131787 | 2.00E-12 | 0.114946201 | 49.48230587 | Leg fat percentage (left) | ukb-b-18377 |
| rs2871866 | C | T | 0.232322 | -0.00918433 | 0.00150264 | 9.80E-10 | 0.089296998 | 37.35812481 | Leg fat percentage (left) | ukb-b-18377 |
| rs2929458 | C | G | 0.18853 | -0.00964545 | 0.00161967 | 2.60E-09 | 0.085155772 | 35.46434245 | Leg fat percentage (left) | ukb-b-18377 |
| rs2954033 | G | A | 0.695202 | 0.00831925 | 0.00136833 | 1.20E-09 | 0.088439666 | 36.96465466 | Leg fat percentage (left) | ukb-b-18377 |
| rs302189 | C | A | 0.777517 | -0.00942884 | 0.00151877 | 5.40E-10 | 0.091866512 | 38.54184605 | Leg fat percentage (left) | ukb-b-18377 |
| rs308911 | G | A | 0.71442 | -0.00886538 | 0.00139482 | 2.10E-10 | 0.095866319 | 40.39786182 | Leg fat percentage (left) | ukb-b-18377 |
| rs3115667 | C | T | 0.721632 | 0.013882 | 0.00140166 | 4.00E-23 | 0.204740069 | 98.08864168 | Leg fat percentage (left) | ukb-b-18377 |
| rs3204853 | A | C | 0.258362 | 0.00845392 | 0.00144026 | 4.40E-09 | 0.082930055 | 34.45358902 | Leg fat percentage (left) | ukb-b-18377 |
| rs3213943 | A | C | 0.131633 | -0.0133233 | 0.00183737 | 4.10E-13 | 0.121271833 | 52.5811853 | Leg fat percentage (left) | ukb-b-18377 |
| rs34483452 | A | C | 0.13632 | 0.017644 | 0.00185111 | 1.50E-21 | 0.192541689 | 90.85098585 | Leg fat percentage (left) | ukb-b-18377 |
| rs34769775 | T | C | 0.297428 | -0.00943318 | 0.0013806 | 8.30E-12 | 0.109158144 | 46.68533774 | Leg fat percentage (left) | ukb-b-18377 |
| rs34811474 | A | G | 0.230829 | -0.012519 | 0.00149416 | 5.40E-17 | 0.155587513 | 70.2012859 | Leg fat percentage (left) | ukb-b-18377 |
| rs34898535 | T | C | 0.377907 | -0.0113327 | 0.00130095 | 3.00E-18 | 0.166088818 | 75.88318895 | Leg fat percentage (left) | ukb-b-18377 |
| rs34914000 | C | T | 0.206898 | 0.00892279 | 0.0015565 | 9.90E-09 | 0.079404811 | 32.86268876 | Leg fat percentage (left) | ukb-b-18377 |
| rs35792595 | A | T | 0.297367 | 0.0081487 | 0.00138578 | 4.10E-09 | 0.083202526 | 34.57706132 | Leg fat percentage (left) | ukb-b-18377 |
| rs35929758 | C | T | 0.602577 | 0.00802869 | 0.00129395 | 5.50E-10 | 0.091774707 | 38.49943793 | Leg fat percentage (left) | ukb-b-18377 |
| rs36007635 | A | G | 0.137721 | -0.0102275 | 0.00182829 | 2.20E-08 | 0.075900166 | 31.2931159 | Leg fat percentage (left) | ukb-b-18377 |
| rs3737992 | A | G | 0.168988 | -0.0108663 | 0.00167692 | 9.20E-11 | 0.099268008 | 41.98930566 | Leg fat percentage (left) | ukb-b-18377 |
| rs3749472 | G | A | 0.464693 | 0.0077932 | 0.00126225 | 6.70E-10 | 0.090950186 | 38.11894611 | Leg fat percentage (left) | ukb-b-18377 |
| rs3754963 | T | A | 0.255084 | -0.0107096 | 0.00144195 | 1.10E-13 | 0.126472907 | 55.16277405 | Leg fat percentage (left) | ukb-b-18377 |
| rs3759584 | C | T | 0.36342 | -0.00735607 | 0.00131849 | 2.40E-08 | 0.075527812 | 31.1270545 | Leg fat percentage (left) | ukb-b-18377 |
| rs3764002 | T | C | 0.261507 | -0.0158684 | 0.00143341 | 1.70E-28 | 0.24337719 | 122.5534154 | Leg fat percentage (left) | ukb-b-18377 |
| rs3766823 | A | G | 0.172012 | 0.0115985 | 0.00166702 | 3.50E-12 | 0.112733074 | 48.40854541 | Leg fat percentage (left) | ukb-b-18377 |
| rs3784699 | C | T | 0.60199 | 0.0137719 | 0.00128832 | 1.10E-26 | 0.230725904 | 114.2721039 | Leg fat percentage (left) | ukb-b-18377 |
| rs3791706 | C | T | 0.298586 | 0.00904646 | 0.00137646 | 5.00E-11 | 0.101827466 | 43.19466836 | Leg fat percentage (left) | ukb-b-18377 |
| rs3794497 | G | A | 0.236302 | 0.00980614 | 0.00148841 | 4.40E-11 | 0.102274974 | 43.40612534 | Leg fat percentage (left) | ukb-b-18377 |
| rs3803286 | G | A | 0.666752 | -0.0106209 | 0.00133732 | 2.00E-15 | 0.142035331 | 63.07423042 | Leg fat percentage (left) | ukb-b-18377 |
| rs383701 | A | G | 0.86888 | -0.0102548 | 0.0018659 | 3.90E-08 | 0.073454661 | 30.20491793 | Leg fat percentage (left) | ukb-b-18377 |
| rs3911063 | C | T | 0.32199 | -0.0110761 | 0.0013501 | 2.30E-16 | 0.150130558 | 67.30415277 | Leg fat percentage (left) | ukb-b-18377 |
| rs40071 | C | T | 0.179492 | -0.013903 | 0.00164614 | 3.00E-17 | 0.157698055 | 71.33185357 | Leg fat percentage (left) | ukb-b-18377 |
| rs403694 | T | C | 0.537353 | 0.0105395 | 0.00127094 | 1.10E-16 | 0.152897656 | 68.7685583 | Leg fat percentage (left) | ukb-b-18377 |
| rs429343 | G | A | 0.57656 | -0.00882694 | 0.00127422 | 4.30E-12 | 0.111863009 | 47.98787448 | Leg fat percentage (left) | ukb-b-18377 |
| rs429358 | C | T | 0.154046 | -0.0180946 | 0.00174885 | 4.30E-25 | 0.219344717 | 107.0515233 | Leg fat percentage (left) | ukb-b-18377 |
| rs4307239 | G | A | 0.458844 | 0.00695778 | 0.00126721 | 0.00000004 | 0.073324155 | 30.14700703 | Leg fat percentage (left) | ukb-b-18377 |
| rs4339600 | G | A | 0.727861 | -0.0082295 | 0.00142037 | 6.90E-09 | 0.080974196 | 33.56942616 | Leg fat percentage (left) | ukb-b-18377 |
| rs4357530 | A | G | 0.678762 | -0.00906544 | 0.00135085 | 1.90E-11 | 0.105710184 | 45.03638483 | Leg fat percentage (left) | ukb-b-18377 |
| rs4398538 | C | T | 0.642508 | -0.00734851 | 0.00131709 | 2.40E-08 | 0.075532578 | 31.12917954 | Leg fat percentage (left) | ukb-b-18377 |
| rs4469245 | T | A | 0.662765 | -0.00892137 | 0.00133257 | 2.20E-11 | 0.105258168 | 44.82115454 | Leg fat percentage (left) | ukb-b-18377 |
| rs4477562 | T | C | 0.128606 | 0.0150888 | 0.00189921 | 1.90E-15 | 0.14212275 | 63.1194822 | Leg fat percentage (left) | ukb-b-18377 |
| rs4500770 | T | A | 0.362622 | -0.00755828 | 0.00131295 | 8.60E-09 | 0.080020753 | 33.13977672 | Leg fat percentage (left) | ukb-b-18377 |
| rs4528240 | T | C | 0.342004 | -0.00760474 | 0.00134555 | 0.000000016 | 0.07735341 | 31.9425115 | Leg fat percentage (left) | ukb-b-18377 |
| rs4530352 | C | A | 0.928417 | -0.0132257 | 0.00241447 | 4.30E-08 | 0.07300399 | 30.00500527 | Leg fat percentage (left) | ukb-b-18377 |
| rs466765 | T | A | 0.798395 | -0.00961032 | 0.00157942 | 1.20E-09 | 0.088568537 | 37.02375228 | Leg fat percentage (left) | ukb-b-18377 |
| rs4711634 | A | G | 0.552641 | -0.0134069 | 0.00126516 | 3.10E-26 | 0.227644881 | 112.2964003 | Leg fat percentage (left) | ukb-b-18377 |
| rs4718964 | T | G | 0.413112 | 0.00908376 | 0.00128369 | 1.50E-12 | 0.116160723 | 50.07385029 | Leg fat percentage (left) | ukb-b-18377 |
| rs4740627 | C | T | 0.442534 | -0.016271 | 0.00127149 | 1.70E-37 | 0.300606907 | 163.7580252 | Leg fat percentage (left) | ukb-b-18377 |
| rs4762951 | G | A | 0.78043 | -0.00925534 | 0.0015231 | 1.20E-09 | 0.088354549 | 36.92563042 | Leg fat percentage (left) | ukb-b-18377 |
| rs4779523 | A | T | 0.273051 | 0.0079818 | 0.00141856 | 1.80E-08 | 0.076720998 | 31.65966125 | Leg fat percentage (left) | ukb-b-18377 |
| rs4780885 | C | G | 0.497161 | -0.00689949 | 0.00126236 | 4.60E-08 | 0.072704409 | 29.8722221 | Leg fat percentage (left) | ukb-b-18377 |
| rs479018 | A | G | 0.332507 | -0.0113092 | 0.00136018 | 9.20E-17 | 0.153579185 | 69.13070718 | Leg fat percentage (left) | ukb-b-18377 |
| rs4790292 | A | C | 0.153743 | -0.0153592 | 0.00175561 | 2.20E-18 | 0.16728356 | 76.53870314 | Leg fat percentage (left) | ukb-b-18377 |
| rs4808187 | C | A | 0.078653 | 0.0147021 | 0.00234844 | 3.80E-10 | 0.093272111 | 39.19221482 | Leg fat percentage (left) | ukb-b-18377 |
| rs4808762 | C | T | 0.290963 | 0.0138123 | 0.00138915 | 2.70E-23 | 0.206023361 | 98.86298488 | Leg fat percentage (left) | ukb-b-18377 |
| rs4820323 | G | C | 0.580967 | -0.00941838 | 0.0012819 | 2.00E-13 | 0.124100473 | 53.98139736 | Leg fat percentage (left) | ukb-b-18377 |
| rs4893977 | T | C | 0.81339 | -0.00923415 | 0.00161351 | 0.00000001 | 0.079160662 | 32.75295792 | Leg fat percentage (left) | ukb-b-18377 |
| rs4894808 | C | G | 0.40014 | -0.00920253 | 0.00131173 | 2.30E-12 | 0.114402844 | 49.21818353 | Leg fat percentage (left) | ukb-b-18377 |
| rs4895229 | C | T | 0.542717 | -0.00737212 | 0.00126677 | 5.90E-09 | 0.081635553 | 33.86797669 | Leg fat percentage (left) | ukb-b-18377 |
| rs494048 | C | T | 0.537582 | 0.00831202 | 0.00128709 | 1.10E-10 | 0.098663662 | 41.70569155 | Leg fat percentage (left) | ukb-b-18377 |
| rs4943343 | C | T | 0.925724 | -0.0133349 | 0.00241895 | 3.50E-08 | 0.073870728 | 30.38965315 | Leg fat percentage (left) | ukb-b-18377 |
| rs4958702 | C | T | 0.572317 | -0.00871742 | 0.00127219 | 7.30E-12 | 0.109717262 | 46.9539341 | Leg fat percentage (left) | ukb-b-18377 |
| rs4979236 | A | G | 0.219887 | 0.0106184 | 0.00152933 | 3.80E-12 | 0.11231773 | 48.20762621 | Leg fat percentage (left) | ukb-b-18377 |
| rs5021156 | C | G | 0.171758 | 0.00923108 | 0.00167221 | 3.40E-08 | 0.074059615 | 30.47357434 | Leg fat percentage (left) | ukb-b-18377 |
| rs525101 | C | T | 0.370366 | 0.00930375 | 0.00130849 | 1.20E-12 | 0.117148786 | 50.55629602 | Leg fat percentage (left) | ukb-b-18377 |
| rs529200 | G | A | 0.527775 | 0.00881768 | 0.00126191 | 2.80E-12 | 0.113594979 | 48.82608513 | Leg fat percentage (left) | ukb-b-18377 |
| rs539515 | C | A | 0.204983 | 0.0227591 | 0.00155778 | 2.40E-48 | 0.359072344 | 213.4508658 | Leg fat percentage (left) | ukb-b-18377 |
| rs55655049 | T | C | 0.235432 | -0.0085975 | 0.00149072 | 8.10E-09 | 0.080292837 | 33.26229513 | Leg fat percentage (left) | ukb-b-18377 |
| rs55707359 | G | T | 0.015448 | 0.0315729 | 0.0051764 | 1.10E-09 | 0.088958318 | 37.20259986 | Leg fat percentage (left) | ukb-b-18377 |
| rs55864937 | G | T | 0.21315 | -0.0124246 | 0.0015403 | 7.20E-16 | 0.145866333 | 65.06601379 | Leg fat percentage (left) | ukb-b-18377 |
| rs55871839 | C | T | 0.417106 | 0.00748384 | 0.00128579 | 5.90E-09 | 0.08165621 | 33.87730861 | Leg fat percentage (left) | ukb-b-18377 |
| rs56161855 | T | A | 0.132867 | 0.0123863 | 0.00185893 | 2.70E-11 | 0.104366791 | 44.39735695 | Leg fat percentage (left) | ukb-b-18377 |
| rs56328878 | A | C | 0.267181 | -0.00794865 | 0.00141988 | 2.20E-08 | 0.076002728 | 31.33887952 | Leg fat percentage (left) | ukb-b-18377 |
| rs56356382 | C | T | 0.192342 | -0.0155153 | 0.0016072 | 4.70E-22 | 0.19652867 | 93.19240236 | Leg fat percentage (left) | ukb-b-18377 |
| rs56399737 | T | C | 0.449164 | -0.0102987 | 0.00127221 | 5.70E-16 | 0.146755886 | 65.53106179 | Leg fat percentage (left) | ukb-b-18377 |
| rs57636386 | C | T | 0.083842 | -0.0195907 | 0.0022831 | 9.40E-18 | 0.161954389 | 73.62919295 | Leg fat percentage (left) | ukb-b-18377 |
| rs57800857 | C | A | 0.364897 | -0.0109697 | 0.00131588 | 7.70E-17 | 0.154264656 | 69.49553921 | Leg fat percentage (left) | ukb-b-18377 |
| rs58582157 | C | G | 0.296895 | 0.00849386 | 0.00138107 | 7.70E-10 | 0.09031216 | 37.82498931 | Leg fat percentage (left) | ukb-b-18377 |
| rs58862095 | T | C | 0.419271 | -0.0105948 | 0.00128002 | 1.30E-16 | 0.152409822 | 68.50969197 | Leg fat percentage (left) | ukb-b-18377 |
| rs588660 | A | G | 0.584203 | 0.0105536 | 0.00127617 | 1.30E-16 | 0.152181574 | 68.38867619 | Leg fat percentage (left) | ukb-b-18377 |
| rs58948644 | A | G | 0.582745 | 0.00941525 | 0.00127656 | 1.60E-13 | 0.124938138 | 54.39778902 | Leg fat percentage (left) | ukb-b-18377 |
| rs59104534 | T | C | 0.298469 | 0.00805293 | 0.00138236 | 5.70E-09 | 0.081786951 | 33.93638131 | Leg fat percentage (left) | ukb-b-18377 |
| rs59227842 | G | A | 0.311472 | 0.0131853 | 0.00137256 | 7.50E-22 | 0.194983247 | 92.28207572 | Leg fat percentage (left) | ukb-b-18377 |
| rs59346947 | A | G | 0.203203 | 0.0100674 | 0.00156515 | 1.30E-10 | 0.097954865 | 41.37354345 | Leg fat percentage (left) | ukb-b-18377 |
| rs59499656 | T | A | 0.343215 | -0.00888413 | 0.00133236 | 2.60E-11 | 0.104502362 | 44.46175877 | Leg fat percentage (left) | ukb-b-18377 |
| rs6011058 | T | C | 0.674615 | 0.0101621 | 0.00134633 | 4.40E-14 | 0.130082005 | 56.97231701 | Leg fat percentage (left) | ukb-b-18377 |
| rs6021948 | A | T | 0.321782 | -0.0110906 | 0.00135332 | 2.50E-16 | 0.149856723 | 67.15975203 | Leg fat percentage (left) | ukb-b-18377 |
| rs6093490 | T | C | 0.218361 | 0.0100581 | 0.00152872 | 4.70E-11 | 0.102026829 | 43.2888455 | Leg fat percentage (left) | ukb-b-18377 |
| rs61023343 | C | A | 0.637845 | 0.0074323 | 0.00131623 | 0.000000016 | 0.077224314 | 31.88474091 | Leg fat percentage (left) | ukb-b-18377 |
| rs6138536 | G | A | 0.46246 | 0.00910122 | 0.00126588 | 6.50E-13 | 0.119463815 | 51.69090648 | Leg fat percentage (left) | ukb-b-18377 |
| rs61734410 | T | C | 0.255219 | -0.00918434 | 0.00147987 | 5.40E-10 | 0.091812021 | 38.51667358 | Leg fat percentage (left) | ukb-b-18377 |
| rs61754230 | T | C | 0.019709 | 0.0262355 | 0.00453111 | 7.00E-09 | 0.08087582 | 33.52505353 | Leg fat percentage (left) | ukb-b-18377 |
| rs61782665 | G | A | 0.584626 | -0.00854848 | 0.00127733 | 2.20E-11 | 0.105190644 | 44.78902157 | Leg fat percentage (left) | ukb-b-18377 |
| rs61986205 | G | A | 0.082021 | 0.0144617 | 0.00231539 | 4.20E-10 | 0.092881542 | 39.01129702 | Leg fat percentage (left) | ukb-b-18377 |
| rs62107261 | C | T | 0.048321 | -0.0341209 | 0.00294013 | 3.90E-31 | 0.261171842 | 134.6814827 | Leg fat percentage (left) | ukb-b-18377 |
| rs62124758 | T | C | 0.143902 | 0.010722 | 0.00181786 | 3.70E-09 | 0.083667864 | 34.78810231 | Leg fat percentage (left) | ukb-b-18377 |
| rs62183912 | A | G | 0.55608 | 0.00873517 | 0.00126455 | 4.90E-12 | 0.11130131 | 47.71673406 | Leg fat percentage (left) | ukb-b-18377 |
| rs62443626 | A | G | 0.463981 | -0.00795872 | 0.00126948 | 3.60E-10 | 0.093512871 | 39.30381666 | Leg fat percentage (left) | ukb-b-18377 |
| rs6265 | T | C | 0.188493 | -0.0183336 | 0.00161074 | 5.10E-30 | 0.253749099 | 129.5521474 | Leg fat percentage (left) | ukb-b-18377 |
| rs6425839 | T | G | 0.511668 | -0.0070747 | 0.00126945 | 0.000000025 | 0.075374709 | 31.05881323 | Leg fat percentage (left) | ukb-b-18377 |
| rs6478538 | G | A | 0.677178 | -0.00830604 | 0.00134879 | 7.40E-10 | 0.090524359 | 37.92270971 | Leg fat percentage (left) | ukb-b-18377 |
| rs6480742 | G | C | 0.155162 | 0.0101162 | 0.00173774 | 5.80E-09 | 0.081683205 | 33.8895044 | Leg fat percentage (left) | ukb-b-18377 |
| rs6495017 | A | T | 0.238296 | -0.0115122 | 0.00148355 | 8.50E-15 | 0.136477473 | 60.21605172 | Leg fat percentage (left) | ukb-b-18377 |
| rs6500594 | G | T | 0.246619 | 0.00846894 | 0.00146498 | 7.40E-09 | 0.080640825 | 33.41909775 | Leg fat percentage (left) | ukb-b-18377 |
| rs6540497 | C | G | 0.578933 | -0.00727917 | 0.0012756 | 0.000000012 | 0.078739421 | 32.56377184 | Leg fat percentage (left) | ukb-b-18377 |
| rs6561937 | A | T | 0.753693 | -0.0119407 | 0.00146805 | 4.20E-16 | 0.147950875 | 66.15731631 | Leg fat percentage (left) | ukb-b-18377 |
| rs6575340 | A | G | 0.636028 | 0.0105346 | 0.00131399 | 1.10E-15 | 0.144351812 | 64.27646441 | Leg fat percentage (left) | ukb-b-18377 |
| rs6602997 | T | C | 0.711835 | 0.0152639 | 0.00139502 | 7.30E-28 | 0.239097147 | 119.7209505 | Leg fat percentage (left) | ukb-b-18377 |
| rs663129 | A | G | 0.233336 | 0.0197538 | 0.0014903 | 4.20E-40 | 0.315600961 | 175.6927748 | Leg fat percentage (left) | ukb-b-18377 |
| rs6674490 | A | C | 0.410273 | -0.00714133 | 0.00128065 | 0.000000025 | 0.075456984 | 31.09548227 | Leg fat percentage (left) | ukb-b-18377 |
| rs6744646 | G | A | 0.828312 | 0.0227628 | 0.00166618 | 1.70E-42 | 0.328801369 | 186.6412055 | Leg fat percentage (left) | ukb-b-18377 |
| rs67776659 | C | T | 0.153835 | 0.0103307 | 0.00174309 | 3.10E-09 | 0.084410363 | 35.12528666 | Leg fat percentage (left) | ukb-b-18377 |
| rs6782581 | G | C | 0.439291 | -0.00757467 | 0.00126849 | 2.40E-09 | 0.085580324 | 35.65770094 | Leg fat percentage (left) | ukb-b-18377 |
| rs6860245 | C | G | 0.247899 | -0.0196312 | 0.00146178 | 4.10E-41 | 0.321285847 | 180.3556135 | Leg fat percentage (left) | ukb-b-18377 |
| rs6935311 | G | A | 0.661286 | 0.00749448 | 0.00132951 | 0.000000017 | 0.076981313 | 31.77604146 | Leg fat percentage (left) | ukb-b-18377 |
| rs6938973 | C | T | 0.601535 | 0.0140078 | 0.00128714 | 1.40E-27 | 0.237141393 | 118.4372434 | Leg fat percentage (left) | ukb-b-18377 |
| rs6973656 | G | A | 0.39701 | 0.0072981 | 0.00128685 | 1.40E-08 | 0.077846982 | 32.16353435 | Leg fat percentage (left) | ukb-b-18377 |
| rs6977416 | A | G | 0.334246 | -0.00955804 | 0.00134729 | 1.30E-12 | 0.116682891 | 50.32867707 | Leg fat percentage (left) | ukb-b-18377 |
| rs7006629 | T | C | 0.516043 | 0.00816172 | 0.00126037 | 9.40E-11 | 0.099150423 | 41.9340944 | Leg fat percentage (left) | ukb-b-18377 |
| rs7027304 | T | C | 0.652646 | 0.00857201 | 0.00132995 | 1.20E-10 | 0.098315979 | 41.54269914 | Leg fat percentage (left) | ukb-b-18377 |
| rs7083543 | T | C | 0.26096 | 0.0114 | 0.00143726 | 2.20E-15 | 0.141723332 | 62.91280149 | Leg fat percentage (left) | ukb-b-18377 |
| rs7097348 | T | C | 0.716773 | 0.00809037 | 0.00140199 | 7.90E-09 | 0.080376999 | 33.3002072 | Leg fat percentage (left) | ukb-b-18377 |
| rs7117842 | C | T | 0.369027 | 0.00979526 | 0.00131218 | 8.30E-14 | 0.127596185 | 55.72436243 | Leg fat percentage (left) | ukb-b-18377 |
| rs7124681 | A | C | 0.408395 | 0.0158122 | 0.00127913 | 4.20E-35 | 0.286264527 | 152.8112148 | Leg fat percentage (left) | ukb-b-18377 |
| rs7132908 | A | G | 0.384442 | 0.0144829 | 0.00129616 | 5.50E-29 | 0.246814559 | 124.8515201 | Leg fat percentage (left) | ukb-b-18377 |
| rs71495038 | A | G | 0.076948 | 0.0146402 | 0.00236423 | 5.90E-10 | 0.091441337 | 38.34551471 | Leg fat percentage (left) | ukb-b-18377 |
| rs7179372 | A | G | 0.206498 | 0.00938288 | 0.00156114 | 1.90E-09 | 0.086601231 | 36.12340008 | Leg fat percentage (left) | ukb-b-18377 |
| rs7187604 | G | A | 0.369818 | 0.0143764 | 0.0013003 | 2.00E-28 | 0.242905897 | 122.239952 | Leg fat percentage (left) | ukb-b-18377 |
| rs719802 | C | T | 0.614404 | -0.00956609 | 0.00129365 | 1.40E-13 | 0.125506685 | 54.68086037 | Leg fat percentage (left) | ukb-b-18377 |
| rs7206608 | G | C | 0.321562 | 0.00940084 | 0.00135078 | 3.40E-12 | 0.112788833 | 48.43553251 | Leg fat percentage (left) | ukb-b-18377 |
| rs7210186 | G | A | 0.314741 | 0.0075274 | 0.00136761 | 3.70E-08 | 0.073656729 | 30.29461614 | Leg fat percentage (left) | ukb-b-18377 |
| rs7218014 | C | T | 0.197305 | 0.0146531 | 0.00158774 | 2.70E-20 | 0.182706268 | 85.17266861 | Leg fat percentage (left) | ukb-b-18377 |
| rs7229869 | C | A | 0.381051 | 0.00764501 | 0.00129716 | 3.80E-09 | 0.083551122 | 34.73513727 | Leg fat percentage (left) | ukb-b-18377 |
| rs723585 | G | A | 0.484913 | -0.00745137 | 0.0012576 | 3.10E-09 | 0.084368966 | 35.10647305 | Leg fat percentage (left) | ukb-b-18377 |
| rs7258937 | T | C | 0.508039 | 0.00954894 | 0.00126378 | 4.20E-14 | 0.130317601 | 57.09096353 | Leg fat percentage (left) | ukb-b-18377 |
| rs72634826 | A | G | 0.259879 | -0.0119361 | 0.0014543 | 2.30E-16 | 0.150240771 | 67.36229719 | Leg fat percentage (left) | ukb-b-18377 |
| rs72677847 | C | T | 0.035561 | -0.0243808 | 0.00344 | 1.40E-12 | 0.116484518 | 50.23183213 | Leg fat percentage (left) | ukb-b-18377 |
| rs72744607 | A | G | 0.337179 | -0.00736057 | 0.00133824 | 3.80E-08 | 0.07356086 | 30.25205497 | Leg fat percentage (left) | ukb-b-18377 |
| rs72815827 | G | T | 0.299933 | 0.00760855 | 0.0013821 | 3.70E-08 | 0.073681778 | 30.30573797 | Leg fat percentage (left) | ukb-b-18377 |
| rs72892910 | T | G | 0.172203 | 0.017685 | 0.00167042 | 3.40E-26 | 0.227318307 | 112.0879086 | Leg fat percentage (left) | ukb-b-18377 |
| rs72913721 | G | A | 0.228247 | -0.0110253 | 0.00149937 | 1.90E-13 | 0.124280563 | 54.07085002 | Leg fat percentage (left) | ukb-b-18377 |
| rs72917533 | C | T | 0.185521 | -0.0108899 | 0.00162161 | 1.90E-11 | 0.105838995 | 45.09775841 | Leg fat percentage (left) | ukb-b-18377 |
| rs73026725 | A | C | 0.153583 | -0.0113558 | 0.0017525 | 9.20E-11 | 0.099264039 | 41.98744199 | Leg fat percentage (left) | ukb-b-18377 |
| rs73091318 | C | T | 0.102623 | 0.0118561 | 0.00210259 | 0.000000017 | 0.077026272 | 31.79614862 | Leg fat percentage (left) | ukb-b-18377 |
| rs73197345 | A | T | 0.136505 | -0.0117334 | 0.00184615 | 2.10E-10 | 0.095857419 | 40.39371374 | Leg fat percentage (left) | ukb-b-18377 |
| rs73213484 | T | A | 0.141226 | -0.0129072 | 0.0018093 | 9.80E-13 | 0.117833433 | 50.89122593 | Leg fat percentage (left) | ukb-b-18377 |
| rs73759009 | G | A | 0.114593 | -0.0110166 | 0.00198443 | 2.80E-08 | 0.07483708 | 30.81935846 | Leg fat percentage (left) | ukb-b-18377 |
| rs7442885 | G | C | 0.214123 | -0.0130198 | 0.00153796 | 2.50E-17 | 0.158321472 | 71.66688783 | Leg fat percentage (left) | ukb-b-18377 |
| rs745249 | T | C | 0.281892 | 0.00912355 | 0.0014019 | 7.60E-11 | 0.100043771 | 42.35392288 | Leg fat percentage (left) | ukb-b-18377 |
| rs74752745 | C | G | 0.011472 | -0.0345814 | 0.00603399 | 0.00000001 | 0.079366591 | 32.84550704 | Leg fat percentage (left) | ukb-b-18377 |
| rs75035127 | G | A | 0.030384 | -0.0245311 | 0.00366633 | 2.20E-11 | 0.105147168 | 44.76833481 | Leg fat percentage (left) | ukb-b-18377 |
| rs7528932 | T | A | 0.392765 | 0.010952 | 0.0012849 | 1.50E-17 | 0.160149387 | 72.65210669 | Leg fat percentage (left) | ukb-b-18377 |
| rs7537581 | A | C | 0.531726 | 0.00835233 | 0.00126983 | 4.80E-11 | 0.101973706 | 43.26374634 | Leg fat percentage (left) | ukb-b-18377 |
| rs75435178 | A | G | 0.210056 | -0.00934968 | 0.00154895 | 1.60E-09 | 0.087283036 | 36.43499348 | Leg fat percentage (left) | ukb-b-18377 |
| rs75499503 | T | C | 0.220143 | -0.015939 | 0.00154173 | 4.70E-25 | 0.219073828 | 106.8822271 | Leg fat percentage (left) | ukb-b-18377 |
| rs75695257 | T | C | 0.102041 | 0.0120269 | 0.00212579 | 0.000000015 | 0.07750105 | 32.00860008 | Leg fat percentage (left) | ukb-b-18377 |
| rs7575523 | G | T | 0.603242 | -0.0116298 | 0.0012895 | 1.90E-19 | 0.175930342 | 81.33955636 | Leg fat percentage (left) | ukb-b-18377 |
| rs7581217 | C | T | 0.646473 | -0.00761031 | 0.00131277 | 6.70E-09 | 0.081057082 | 33.60681913 | Leg fat percentage (left) | ukb-b-18377 |
| rs7609467 | C | T | 0.358423 | 0.00930562 | 0.00131281 | 1.40E-12 | 0.11651008 | 50.24430872 | Leg fat percentage (left) | ukb-b-18377 |
| rs7630228 | C | T | 0.434301 | -0.00831253 | 0.00127698 | 7.50E-11 | 0.100086199 | 42.37388275 | Leg fat percentage (left) | ukb-b-18377 |
| rs76345589 | G | C | 0.067505 | -0.0171815 | 0.00251843 | 9.00E-12 | 0.108863354 | 46.54385862 | Leg fat percentage (left) | ukb-b-18377 |
| rs76458888 | T | C | 0.075921 | 0.0172149 | 0.0024054 | 8.30E-13 | 0.11850322 | 51.21938922 | Leg fat percentage (left) | ukb-b-18377 |
| rs764729 | C | A | 0.72802 | 0.00831985 | 0.00141516 | 4.10E-09 | 0.083172992 | 34.56367427 | Leg fat percentage (left) | ukb-b-18377 |
| rs7665222 | G | A | 0.527402 | -0.007246 | 0.0012685 | 1.10E-08 | 0.078886701 | 32.62989801 | Leg fat percentage (left) | ukb-b-18377 |
| rs7683620 | C | A | 0.464669 | 0.00887213 | 0.00126326 | 2.20E-12 | 0.114623419 | 49.32536457 | Leg fat percentage (left) | ukb-b-18377 |
| rs770082 | A | G | 0.436964 | 0.00970718 | 0.00127592 | 2.80E-14 | 0.131883987 | 57.88143312 | Leg fat percentage (left) | ukb-b-18377 |
| rs7707394 | A | G | 0.357297 | -0.0103559 | 0.00131255 | 3.00E-15 | 0.140441219 | 62.25066333 | Leg fat percentage (left) | ukb-b-18377 |
| rs77572543 | C | A | 0.09859 | -0.0175551 | 0.00211738 | 1.10E-16 | 0.152843709 | 68.73991682 | Leg fat percentage (left) | ukb-b-18377 |
| rs7774 | A | C | 0.310478 | 0.00869098 | 0.00137112 | 2.30E-10 | 0.095394 | 40.17783857 | Leg fat percentage (left) | ukb-b-18377 |
| rs78054636 | T | C | 0.146485 | 0.0111595 | 0.00179754 | 5.40E-10 | 0.09186648 | 38.54183132 | Leg fat percentage (left) | ukb-b-18377 |
| rs78277800 | G | A | 0.091677 | -0.0123301 | 0.00220227 | 2.20E-08 | 0.076020291 | 31.34671766 | Leg fat percentage (left) | ukb-b-18377 |
| rs785510 | C | T | 0.704568 | 0.0095002 | 0.00137872 | 5.60E-12 | 0.110810904 | 47.48028807 | Leg fat percentage (left) | ukb-b-18377 |
| rs7893571 | T | G | 0.665917 | 0.00782131 | 0.00133914 | 5.20E-09 | 0.082175387 | 34.1119883 | Leg fat percentage (left) | ukb-b-18377 |
| rs7966251 | A | G | 0.255014 | -0.00858955 | 0.00144787 | 3.00E-09 | 0.084563854 | 35.19505798 | Leg fat percentage (left) | ukb-b-18377 |
| rs7975187 | G | A | 0.213876 | 0.00943286 | 0.00153762 | 8.50E-10 | 0.089898716 | 37.63472431 | Leg fat percentage (left) | ukb-b-18377 |
| rs7987928 | A | G | 0.799883 | -0.0105169 | 0.00157831 | 2.70E-11 | 0.104374056 | 44.40080791 | Leg fat percentage (left) | ukb-b-18377 |
| rs80135274 | T | A | 0.070312 | 0.0144037 | 0.00247295 | 5.70E-09 | 0.081761351 | 33.92481309 | Leg fat percentage (left) | ukb-b-18377 |
| rs8020365 | A | T | 0.220421 | 0.0148736 | 0.00152666 | 2.00E-22 | 0.199441539 | 94.91777338 | Leg fat percentage (left) | ukb-b-18377 |
| rs8032412 | A | G | 0.92552 | -0.0138755 | 0.00240406 | 7.80E-09 | 0.080404191 | 33.31245798 | Leg fat percentage (left) | ukb-b-18377 |
| rs8051058 | C | T | 0.75527 | 0.00859393 | 0.00147029 | 5.10E-09 | 0.08229192 | 34.16470042 | Leg fat percentage (left) | ukb-b-18377 |
| rs8086738 | A | C | 0.61218 | -0.00741071 | 0.0012958 | 1.10E-08 | 0.079058866 | 32.70722404 | Leg fat percentage (left) | ukb-b-18377 |
| rs8119351 | A | G | 0.085035 | -0.0162389 | 0.00226408 | 7.40E-13 | 0.118959849 | 51.44340164 | Leg fat percentage (left) | ukb-b-18377 |
| rs812949 | C | T | 0.729202 | 0.00931092 | 0.00141728 | 5.00E-11 | 0.101752463 | 43.15924841 | Leg fat percentage (left) | ukb-b-18377 |
| rs853961 | T | G | 0.50591 | 0.00924132 | 0.00125923 | 2.20E-13 | 0.123853841 | 53.85895145 | Leg fat percentage (left) | ukb-b-18377 |
| rs866279 | G | A | 0.118146 | 0.0108892 | 0.00195459 | 0.000000025 | 0.075325899 | 31.03706201 | Leg fat percentage (left) | ukb-b-18377 |
| rs879620 | T | C | 0.613277 | 0.0109592 | 0.00129854 | 3.20E-17 | 0.157503488 | 71.22739171 | Leg fat percentage (left) | ukb-b-18377 |
| rs925826 | G | A | 0.857415 | -0.0110724 | 0.00179896 | 7.50E-10 | 0.090437413 | 37.88266466 | Leg fat percentage (left) | ukb-b-18377 |
| rs9304665 | A | T | 0.76364 | 0.00957463 | 0.00148838 | 1.30E-10 | 0.097973917 | 41.38246431 | Leg fat percentage (left) | ukb-b-18377 |
| rs9389857 | T | C | 0.052435 | 0.0167142 | 0.00283212 | 3.60E-09 | 0.083759203 | 34.82955192 | Leg fat percentage (left) | ukb-b-18377 |
| rs9449999 | G | A | 0.589168 | -0.00767026 | 0.00128286 | 2.20E-09 | 0.085780243 | 35.74881441 | Leg fat percentage (left) | ukb-b-18377 |
| rs9489620 | C | G | 0.548032 | 0.0076928 | 0.00126681 | 1.30E-09 | 0.088246665 | 36.87617925 | Leg fat percentage (left) | ukb-b-18377 |
| rs9515455 | A | G | 0.414829 | 0.0102543 | 0.00128725 | 1.60E-15 | 0.142776105 | 63.45797906 | Leg fat percentage (left) | ukb-b-18377 |
| rs9569946 | T | C | 0.182741 | -0.00940778 | 0.00164065 | 9.80E-09 | 0.079445104 | 32.88080338 | Leg fat percentage (left) | ukb-b-18377 |
| rs9641499 | A | C | 0.434713 | -0.00947278 | 0.00127073 | 9.00E-14 | 0.127289751 | 55.57101589 | Leg fat percentage (left) | ukb-b-18377 |
| rs972283 | G | A | 0.511937 | -0.0098687 | 0.00125901 | 4.60E-15 | 0.138869131 | 61.44146109 | Leg fat percentage (left) | ukb-b-18377 |
| rs9788550 | C | G | 0.247499 | -0.0116612 | 0.00146694 | 1.90E-15 | 0.142262717 | 63.1919542 | Leg fat percentage (left) | ukb-b-18377 |
| rs9835574 | C | A | 0.277143 | 0.0114482 | 0.0014087 | 4.40E-16 | 0.147736063 | 66.04461102 | Leg fat percentage (left) | ukb-b-18377 |
| rs9839081 | A | G | 0.325085 | -0.00992174 | 0.0013653 | 3.70E-13 | 0.121736143 | 52.8104057 | Leg fat percentage (left) | ukb-b-18377 |
| rs9843653 | C | T | 0.511537 | 0.0153635 | 0.00125976 | 3.30E-34 | 0.28076852 | 148.7320968 | Leg fat percentage (left) | ukb-b-18377 |
| rs9852062 | A | T | 0.556841 | -0.00829979 | 0.00127195 | 6.80E-11 | 0.100521704 | 42.57886974 | Leg fat percentage (left) | ukb-b-18377 |
| rs9860326 | G | C | 0.328355 | 0.00966646 | 0.00134155 | 5.80E-13 | 0.1199265 | 51.91838673 | Leg fat percentage (left) | ukb-b-18377 |
| rs9863890 | G | A | 0.409705 | -0.00833986 | 0.00127751 | 6.70E-11 | 0.100603923 | 42.61759165 | Leg fat percentage (left) | ukb-b-18377 |
| rs9867130 | G | A | 0.859298 | 0.0127736 | 0.00184105 | 4.00E-12 | 0.112175407 | 48.13882192 | Leg fat percentage (left) | ukb-b-18377 |
| rs9902386 | G | T | 0.675861 | -0.0128856 | 0.00136188 | 3.00E-21 | 0.190261679 | 89.52237745 | Leg fat percentage (left) | ukb-b-18377 |
| rs9906944 | T | C | 0.340578 | -0.0107598 | 0.00133633 | 8.20E-16 | 0.145415585 | 64.83073738 | Leg fat percentage (left) | ukb-b-18377 |
| rs9920515 | A | G | 0.946971 | -0.0164099 | 0.00283004 | 6.70E-09 | 0.081091274 | 33.62224599 | Leg fat percentage (left) | ukb-b-18377 |
| rs9944241 | C | T | 0.484332 | -0.00876484 | 0.0013149 | 2.60E-11 | 0.104441159 | 44.4326826 | Leg fat percentage (left) | ukb-b-18377 |

Supplementary Table 13: Information on all instrumental variables (IVs) ultimately used for Leg fat percentage (right) in our study

| SNPs | effect_allele.exposure | other_allele.exposure | eaf.exposure | beta.exposure | se.exposure | pval.exposure | R^2^ | F | trait | id.exposure |
| --- | --- | --- | --- | --- | --- | --- | --- | --- | --- | --- |
| rs10050620 | T | C | 0.32542 | -0.00799711 | 0.00136316 | 4.40E-09 | 0.082255168 | 34.41695721 | Leg fat percentage (right) | ukb-b-20531 |
| rs10100245 | A | G | 0.564469 | 0.0108997 | 0.00128865 | 2.70E-17 | 0.157047495 | 71.54167981 | Leg fat percentage (right) | ukb-b-20531 |
| rs10119967 | C | A | 0.204098 | -0.0100392 | 0.0015868 | 2.50E-10 | 0.094397446 | 40.02707261 | Leg fat percentage (right) | ukb-b-20531 |
| rs1013293 | A | G | 0.430275 | -0.010187 | 0.00129092 | 3.00E-15 | 0.139538534 | 62.27216338 | Leg fat percentage (right) | ukb-b-20531 |
| rs10144067 | T | C | 0.591283 | 0.00814978 | 0.00130998 | 4.90E-10 | 0.091564152 | 38.70458723 | Leg fat percentage (right) | ukb-b-20531 |
| rs10187101 | T | C | 0.363617 | -0.00856196 | 0.00132352 | 9.90E-11 | 0.098271981 | 41.8490277 | Leg fat percentage (right) | ukb-b-20531 |
| rs10209821 | T | C | 0.342612 | 0.0112297 | 0.00134032 | 5.40E-17 | 0.154552108 | 70.19712288 | Leg fat percentage (right) | ukb-b-20531 |
| rs1022185 | A | G | 0.760785 | -0.0087364 | 0.00150244 | 6.10E-09 | 0.08092633 | 33.81199106 | Leg fat percentage (right) | ukb-b-20531 |
| rs10245306 | C | G | 0.685983 | 0.0076504 | 0.0013867 | 3.40E-08 | 0.073442023 | 30.43709883 | Leg fat percentage (right) | ukb-b-20531 |
| rs10404726 | T | C | 0.465527 | -0.0119592 | 0.00128306 | 1.20E-20 | 0.18450232 | 86.87810232 | Leg fat percentage (right) | ukb-b-20531 |
| rs10423928 | A | T | 0.194435 | -0.0203475 | 0.00161237 | 1.60E-36 | 0.293149445 | 159.2548606 | Leg fat percentage (right) | ukb-b-20531 |
| rs10498240 | A | C | 0.318275 | 0.0124695 | 0.00136858 | 8.10E-20 | 0.177756983 | 83.01521576 | Leg fat percentage (right) | ukb-b-20531 |
| rs10499014 | G | C | 0.268657 | -0.0103759 | 0.00144785 | 7.70E-13 | 0.117966398 | 51.35756377 | Leg fat percentage (right) | ukb-b-20531 |
| rs10510025 | T | C | 0.247075 | 0.0118527 | 0.00148387 | 1.40E-15 | 0.142480563 | 63.80326087 | Leg fat percentage (right) | ukb-b-20531 |
| rs10514963 | A | G | 0.480667 | 0.00988307 | 0.00127662 | 9.80E-15 | 0.135003111 | 59.93223239 | Leg fat percentage (right) | ukb-b-20531 |
| rs10516044 | C | G | 0.115985 | 0.0116116 | 0.00200251 | 6.70E-09 | 0.08051012 | 33.62286729 | Leg fat percentage (right) | ukb-b-20531 |
| rs10732335 | C | A | 0.443 | -0.016394 | 0.00128755 | 3.90E-37 | 0.296860265 | 162.1218889 | Leg fat percentage (right) | ukb-b-20531 |
| rs10756793 | A | G | 0.74309 | -0.0127218 | 0.00146717 | 4.30E-18 | 0.16373734 | 75.18587375 | Leg fat percentage (right) | ukb-b-20531 |
| rs10761737 | C | T | 0.41632 | -0.00726337 | 0.00129612 | 0.000000021 | 0.075598846 | 31.40406818 | Leg fat percentage (right) | ukb-b-20531 |
| rs10823242 | A | G | 0.574816 | -0.00887915 | 0.00129502 | 7.10E-12 | 0.109069302 | 47.00995488 | Leg fat percentage (right) | ukb-b-20531 |
| rs10854853 | T | G | 0.456803 | 0.0079093 | 0.00128375 | 7.20E-10 | 0.089959056 | 37.95903683 | Leg fat percentage (right) | ukb-b-20531 |
| rs10883026 | T | C | 0.521463 | -0.0100488 | 0.00128755 | 6.00E-15 | 0.136907249 | 60.91162683 | Leg fat percentage (right) | ukb-b-20531 |
| rs10885421 | T | G | 0.598233 | -0.00831972 | 0.00130915 | 2.10E-10 | 0.095164904 | 40.38672159 | Leg fat percentage (right) | ukb-b-20531 |
| rs10887578 | C | G | 0.497531 | 0.00796438 | 0.00128409 | 5.60E-10 | 0.091057973 | 38.46918792 | Leg fat percentage (right) | ukb-b-20531 |
| rs10896012 | C | T | 0.217388 | 0.0119565 | 0.00155163 | 1.30E-14 | 0.133923511 | 59.37885251 | Leg fat percentage (right) | ukb-b-20531 |
| rs10938397 | G | A | 0.434304 | 0.0154101 | 0.00128867 | 5.90E-33 | 0.271343351 | 142.9971814 | Leg fat percentage (right) | ukb-b-20531 |
| rs10951992 | C | T | 0.439104 | 0.00818821 | 0.00130234 | 3.20E-10 | 0.093335055 | 39.53021581 | Leg fat percentage (right) | ukb-b-20531 |
| rs10973850 | G | A | 0.442311 | 0.00730873 | 0.00128938 | 1.40E-08 | 0.077213294 | 32.13083249 | Leg fat percentage (right) | ukb-b-20531 |
| rs10982884 | T | C | 0.093724 | 0.014725 | 0.00219703 | 2.10E-11 | 0.104727914 | 44.91988467 | Leg fat percentage (right) | ukb-b-20531 |
| rs10985968 | G | C | 0.50137 | -0.00749896 | 0.00128135 | 4.80E-09 | 0.081889853 | 34.25046938 | Leg fat percentage (right) | ukb-b-20531 |
| rs10989067 | A | G | 0.315883 | 0.00848764 | 0.0013711 | 6.00E-10 | 0.090738808 | 38.32089468 | Leg fat percentage (right) | ukb-b-20531 |
| rs10992854 | C | T | 0.681931 | -0.010404 | 0.00137643 | 4.10E-14 | 0.12951563 | 57.1337102 | Leg fat percentage (right) | ukb-b-20531 |
| rs10997982 | A | G | 0.493184 | -0.00854112 | 0.00127638 | 2.20E-11 | 0.104432735 | 44.77851287 | Leg fat percentage (right) | ukb-b-20531 |
| rs10999460 | T | C | 0.265497 | 0.0120099 | 0.00145199 | 1.30E-16 | 0.151221836 | 68.4150316 | Leg fat percentage (right) | ukb-b-20531 |
| rs11012732 | G | A | 0.331715 | 0.0147866 | 0.00135628 | 1.10E-27 | 0.236368893 | 118.8606044 | Leg fat percentage (right) | ukb-b-20531 |
| rs11071411 | C | T | 0.234912 | 0.0104922 | 0.00150824 | 3.50E-12 | 0.11192123 | 48.39407707 | Leg fat percentage (right) | ukb-b-20531 |
| rs11074452 | G | C | 0.50838 | -0.00730239 | 0.00127953 | 1.10E-08 | 0.078188022 | 32.57085069 | Leg fat percentage (right) | ukb-b-20531 |
| rs11098965 | T | C | 0.707706 | -0.00959541 | 0.00146339 | 5.50E-11 | 0.100689742 | 42.99390641 | Leg fat percentage (right) | ukb-b-20531 |
| rs11122450 | G | T | 0.611692 | -0.00763237 | 0.00130809 | 5.40E-09 | 0.08143692 | 34.04423506 | Leg fat percentage (right) | ukb-b-20531 |
| rs11150745 | G | A | 0.317698 | -0.0104232 | 0.00137432 | 3.30E-14 | 0.130279189 | 57.5209975 | Leg fat percentage (right) | ukb-b-20531 |
| rs11208779 | C | G | 0.528887 | 0.00798024 | 0.00127914 | 4.40E-10 | 0.09203127 | 38.92205351 | Leg fat percentage (right) | ukb-b-20531 |
| rs11264489 | G | A | 0.361742 | 0.00885212 | 0.00133078 | 2.90E-11 | 0.103320835 | 44.24681908 | Leg fat percentage (right) | ukb-b-20531 |
| rs112852122 | A | G | 0.157846 | -0.014683 | 0.00178046 | 1.60E-16 | 0.15045903 | 68.00880678 | Leg fat percentage (right) | ukb-b-20531 |
| rs113230003 | A | G | 0.26053 | -0.00926674 | 0.00146797 | 2.70E-10 | 0.094017398 | 39.84919895 | Leg fat percentage (right) | ukb-b-20531 |
| rs113728099 | A | G | 0.02475 | -0.0339327 | 0.00414142 | 2.50E-16 | 0.148810424 | 67.13334424 | Leg fat percentage (right) | ukb-b-20531 |
| rs113866544 | C | T | 0.068316 | 0.0206346 | 0.00253516 | 4.00E-16 | 0.147139171 | 66.24931029 | Leg fat percentage (right) | ukb-b-20531 |
| rs113963976 | T | C | 0.324826 | 0.00835087 | 0.00136777 | 0.000000001 | 0.088485044 | 37.27668623 | Leg fat percentage (right) | ukb-b-20531 |
| rs114295766 | T | A | 0.069529 | -0.0164289 | 0.00266219 | 6.80E-10 | 0.09022779 | 38.08367732 | Leg fat percentage (right) | ukb-b-20531 |
| rs115584674 | G | A | 0.058665 | -0.0185702 | 0.00270663 | 6.80E-12 | 0.109200386 | 47.07337966 | Leg fat percentage (right) | ukb-b-20531 |
| rs11563357 | T | A | 0.188737 | 0.00980305 | 0.00163515 | 0.000000002 | 0.08558891 | 35.94241365 | Leg fat percentage (right) | ukb-b-20531 |
| rs11570784 | A | G | 0.037589 | -0.0242627 | 0.00336955 | 6.00E-13 | 0.118959491 | 51.84829105 | Leg fat percentage (right) | ukb-b-20531 |
| rs11640961 | T | C | 0.395465 | -0.00993359 | 0.00130592 | 2.80E-14 | 0.130946686 | 57.86011808 | Leg fat percentage (right) | ukb-b-20531 |
| rs11656758 | G | A | 0.343114 | 0.00927849 | 0.00134653 | 5.60E-12 | 0.110042531 | 47.48129339 | Leg fat percentage (right) | ukb-b-20531 |
| rs11664106 | T | A | 0.373935 | 0.00759565 | 0.00135328 | 0.00000002 | 0.075819375 | 31.50319217 | Leg fat percentage (right) | ukb-b-20531 |
| rs1167309 | T | C | 0.68111 | -0.0117451 | 0.00136684 | 8.50E-18 | 0.16127481 | 73.83768603 | Leg fat percentage (right) | ukb-b-20531 |
| rs11691869 | A | C | 0.361916 | -0.013071 | 0.00132831 | 7.50E-23 | 0.201384209 | 96.83196498 | Leg fat percentage (right) | ukb-b-20531 |
| rs11704728 | T | C | 0.196278 | 0.0100143 | 0.00161615 | 5.80E-10 | 0.090898937 | 38.39528202 | Leg fat percentage (right) | ukb-b-20531 |
| rs117068593 | T | C | 0.189443 | -0.0133306 | 0.00163598 | 3.70E-16 | 0.147417373 | 66.39622899 | Leg fat percentage (right) | ukb-b-20531 |
| rs117113729 | T | C | 0.02862 | 0.0222475 | 0.00388759 | 0.00000001 | 0.078582652 | 32.74926204 | Leg fat percentage (right) | ukb-b-20531 |
| rs117342986 | T | C | 0.026448 | 0.0244191 | 0.00417595 | 0.000000005 | 0.081765675 | 34.19390703 | Leg fat percentage (right) | ukb-b-20531 |
| rs11777625 | C | T | 0.545834 | 0.00799664 | 0.00128215 | 4.50E-10 | 0.091981577 | 38.89890862 | Leg fat percentage (right) | ukb-b-20531 |
| rs11782074 | T | G | 0.383641 | 0.00796951 | 0.00133402 | 2.30E-09 | 0.085037526 | 35.68934343 | Leg fat percentage (right) | ukb-b-20531 |
| rs11789898 | T | G | 0.167115 | -0.0103177 | 0.00171455 | 1.80E-09 | 0.086177902 | 36.21308157 | Leg fat percentage (right) | ukb-b-20531 |
| rs11841757 | G | A | 0.239515 | -0.0123539 | 0.00149704 | 1.60E-16 | 0.150628695 | 68.09909713 | Leg fat percentage (right) | ukb-b-20531 |
| rs11852419 | T | A | 0.26167 | 0.0091362 | 0.00145413 | 3.30E-10 | 0.093217341 | 39.4752353 | Leg fat percentage (right) | ukb-b-20531 |
| rs11866219 | C | A | 0.583798 | -0.0111682 | 0.00131251 | 1.80E-17 | 0.158639711 | 72.40376055 | Leg fat percentage (right) | ukb-b-20531 |
| rs1188162 | G | A | 0.527 | 0.00760891 | 0.00128311 | 3.00E-09 | 0.083894127 | 35.16552577 | Leg fat percentage (right) | ukb-b-20531 |
| rs11926346 | G | A | 0.208371 | -0.00904756 | 0.0015687 | 0.000000008 | 0.07972078 | 33.26466473 | Leg fat percentage (right) | ukb-b-20531 |
| rs12039708 | T | C | 0.186732 | 0.0136505 | 0.00163495 | 6.90E-17 | 0.153642328 | 69.70889 | Leg fat percentage (right) | ukb-b-20531 |
| rs12042959 | G | A | 0.143929 | -0.0120352 | 0.00182447 | 4.20E-11 | 0.101784678 | 43.5144171 | Leg fat percentage (right) | ukb-b-20531 |
| rs12050481 | C | T | 0.6106 | 0.0141577 | 0.00131101 | 3.50E-27 | 0.232951291 | 116.620098 | Leg fat percentage (right) | ukb-b-20531 |
| rs12055234 | A | G | 0.328058 | 0.00900138 | 0.00135946 | 3.60E-11 | 0.102471342 | 43.84149195 | Leg fat percentage (right) | ukb-b-20531 |
| rs12072739 | G | A | 0.22459 | 0.010414 | 0.0015291 | 9.70E-12 | 0.107772454 | 46.38348436 | Leg fat percentage (right) | ukb-b-20531 |
| rs12122664 | C | A | 0.315976 | 0.00758012 | 0.00137415 | 3.50E-08 | 0.07342332 | 30.42873356 | Leg fat percentage (right) | ukb-b-20531 |
| rs12124126 | G | A | 0.334231 | -0.0122579 | 0.00135188 | 1.20E-19 | 0.176347283 | 82.21590885 | Leg fat percentage (right) | ukb-b-20531 |
| rs12186509 | G | T | 0.260931 | -0.00854432 | 0.00145723 | 4.50E-09 | 0.082172872 | 34.37944039 | Leg fat percentage (right) | ukb-b-20531 |
| rs1225004 | C | T | 0.277759 | 0.0114584 | 0.00142516 | 9.00E-16 | 0.144085384 | 64.64288175 | Leg fat percentage (right) | ukb-b-20531 |
| rs1229984 | C | T | 0.97278 | 0.0242928 | 0.00387146 | 3.50E-10 | 0.09299975 | 39.37364274 | Leg fat percentage (right) | ukb-b-20531 |
| rs12316080 | T | C | 0.335379 | 0.0109038 | 0.0013506 | 6.80E-16 | 0.145105411 | 65.17818508 | Leg fat percentage (right) | ukb-b-20531 |
| rs12375196 | A | C | 0.424276 | 0.00831572 | 0.00129914 | 1.50E-10 | 0.096411186 | 40.97206056 | Leg fat percentage (right) | ukb-b-20531 |
| rs12441543 | A | G | 0.287109 | 0.00799056 | 0.00141495 | 0.000000016 | 0.07668182 | 31.89130175 | Leg fat percentage (right) | ukb-b-20531 |
| rs12462975 | A | G | 0.329597 | 0.00865662 | 0.00136882 | 2.50E-10 | 0.094328612 | 39.99484547 | Leg fat percentage (right) | ukb-b-20531 |
| rs12467407 | T | C | 0.469327 | 0.00742513 | 0.00128011 | 6.60E-09 | 0.080557656 | 33.64445851 | Leg fat percentage (right) | ukb-b-20531 |
| rs1250603 | G | A | 0.66344 | 0.00783422 | 0.00135569 | 7.50E-09 | 0.080006331 | 33.39417673 | Leg fat percentage (right) | ukb-b-20531 |
| rs12531825 | A | G | 0.122943 | 0.0107242 | 0.00195861 | 4.40E-08 | 0.072419305 | 29.98015504 | Leg fat percentage (right) | ukb-b-20531 |
| rs12538435 | G | A | 0.26184 | -0.00879696 | 0.00145155 | 1.40E-09 | 0.087297031 | 36.72833443 | Leg fat percentage (right) | ukb-b-20531 |
| rs12588874 | A | G | 0.539767 | 0.00924473 | 0.00128011 | 5.10E-13 | 0.119578548 | 52.15475181 | Leg fat percentage (right) | ukb-b-20531 |
| rs12628603 | A | G | 0.617037 | -0.00794243 | 0.00131709 | 1.60E-09 | 0.086506752 | 36.36435485 | Leg fat percentage (right) | ukb-b-20531 |
| rs12724928 | C | T | 0.204872 | -0.0107337 | 0.00157758 | 1.00E-11 | 0.107585008 | 46.29308487 | Leg fat percentage (right) | ukb-b-20531 |
| rs1274232 | G | A | 0.912558 | 0.0131654 | 0.00226323 | 6.00E-09 | 0.080984576 | 33.83847129 | Leg fat percentage (right) | ukb-b-20531 |
| rs12806052 | T | C | 0.163636 | -0.0113533 | 0.00172804 | 5.00E-11 | 0.101050971 | 43.16548703 | Leg fat percentage (right) | ukb-b-20531 |
| rs12890931 | G | T | 0.362425 | 0.00982565 | 0.00133717 | 2.00E-13 | 0.123276614 | 53.99447605 | Leg fat percentage (right) | ukb-b-20531 |
| rs12895351 | A | G | 0.439941 | -0.00794994 | 0.00128779 | 6.70E-10 | 0.090284269 | 38.1098821 | Leg fat percentage (right) | ukb-b-20531 |
| rs12907408 | G | A | 0.215633 | 0.00949109 | 0.0015662 | 1.40E-09 | 0.087285384 | 36.72296545 | Leg fat percentage (right) | ukb-b-20531 |
| rs12932141 | C | A | 0.356842 | -0.00756141 | 0.00133405 | 1.40E-08 | 0.07720335 | 32.12634806 | Leg fat percentage (right) | ukb-b-20531 |
| rs12937411 | T | C | 0.40803 | -0.00945832 | 0.00129976 | 3.40E-13 | 0.121189669 | 52.95435366 | Leg fat percentage (right) | ukb-b-20531 |
| rs13026103 | A | G | 0.740996 | 0.00831202 | 0.00145372 | 1.10E-08 | 0.078457718 | 32.69276316 | Leg fat percentage (right) | ukb-b-20531 |
| rs13033310 | A | G | 0.252785 | 0.00820221 | 0.0014748 | 2.70E-08 | 0.074545171 | 30.93111037 | Leg fat percentage (right) | ukb-b-20531 |
| rs13107325 | T | C | 0.07486 | 0.0236345 | 0.00242591 | 2.00E-22 | 0.198190723 | 94.91688315 | Leg fat percentage (right) | ukb-b-20531 |
| rs13177679 | T | C | 0.340434 | -0.00812907 | 0.00137617 | 3.50E-09 | 0.083297989 | 34.89293925 | Leg fat percentage (right) | ukb-b-20531 |
| rs1322842 | G | A | 0.609488 | -0.00963038 | 0.0013145 | 2.40E-13 | 0.122635078 | 53.67421077 | Leg fat percentage (right) | ukb-b-20531 |
| rs13389219 | T | C | 0.392427 | 0.0110566 | 0.00130259 | 2.10E-17 | 0.157985143 | 72.04896009 | Leg fat percentage (right) | ukb-b-20531 |
| rs13427822 | G | A | 0.271197 | -0.010186 | 0.00144831 | 2.00E-12 | 0.114112131 | 49.4634364 | Leg fat percentage (right) | ukb-b-20531 |
| rs1377184 | T | A | 0.748946 | 0.0087244 | 0.00147524 | 3.30E-09 | 0.08347543 | 34.97403814 | Leg fat percentage (right) | ukb-b-20531 |
| rs1412239 | G | C | 0.323115 | 0.0127742 | 0.0013629 | 7.10E-21 | 0.186181238 | 87.84952949 | Leg fat percentage (right) | ukb-b-20531 |
| rs1414595 | T | C | 0.373013 | -0.00751579 | 0.00131713 | 0.000000012 | 0.07816527 | 32.56056936 | Leg fat percentage (right) | ukb-b-20531 |
| rs1446585 | G | A | 0.244614 | -0.0102341 | 0.00145201 | 1.80E-12 | 0.114549491 | 49.67754184 | Leg fat percentage (right) | ukb-b-20531 |
| rs145585494 | T | C | 0.011458 | -0.034291 | 0.00621391 | 3.40E-08 | 0.073477619 | 30.45302107 | Leg fat percentage (right) | ukb-b-20531 |
| rs1456014 | G | A | 0.205607 | -0.0132586 | 0.00157514 | 3.80E-17 | 0.155770834 | 70.85279979 | Leg fat percentage (right) | ukb-b-20531 |
| rs145828988 | C | T | 0.053196 | -0.0169253 | 0.00292324 | 7.00E-09 | 0.080290345 | 33.52307164 | Leg fat percentage (right) | ukb-b-20531 |
| rs1471740 | C | T | 0.740365 | 0.0108814 | 0.0014562 | 7.90E-14 | 0.126950685 | 55.83769692 | Leg fat percentage (right) | ukb-b-20531 |
| rs1475535 | A | T | 0.498071 | 0.00910661 | 0.00128084 | 1.20E-12 | 0.116327839 | 50.55029704 | Leg fat percentage (right) | ukb-b-20531 |
| rs1477290 | C | T | 0.136906 | 0.0191467 | 0.00187212 | 1.50E-24 | 0.214076715 | 104.5973061 | Leg fat percentage (right) | ukb-b-20531 |
| rs147730268 | T | G | 0.087262 | -0.0210158 | 0.00231309 | 1.00E-19 | 0.176933642 | 82.54804484 | Leg fat percentage (right) | ukb-b-20531 |
| rs148143761 | G | A | 0.048025 | 0.0220662 | 0.00301418 | 2.50E-13 | 0.122474396 | 53.59406919 | Leg fat percentage (right) | ukb-b-20531 |
| rs1554654 | T | C | 0.470551 | -0.00964894 | 0.00128006 | 4.80E-14 | 0.128895457 | 56.81965053 | Leg fat percentage (right) | ukb-b-20531 |
| rs1568489 | G | A | 0.573041 | 0.00911569 | 0.0012927 | 1.80E-12 | 0.114648435 | 49.72600823 | Leg fat percentage (right) | ukb-b-20531 |
| rs1596032 | T | C | 0.458786 | 0.00789824 | 0.00130976 | 1.60E-09 | 0.086506992 | 36.36446535 | Leg fat percentage (right) | ukb-b-20531 |
| rs16916303 | G | A | 0.119778 | -0.0109868 | 0.00198764 | 0.000000032 | 0.073703133 | 30.55392312 | Leg fat percentage (right) | ukb-b-20531 |
| rs16996657 | C | T | 0.127761 | 0.0114763 | 0.00191974 | 2.30E-09 | 0.085141565 | 35.73707117 | Leg fat percentage (right) | ukb-b-20531 |
| rs17024393 | C | T | 0.025905 | 0.0315209 | 0.00402288 | 4.70E-15 | 0.137841216 | 61.39359463 | Leg fat percentage (right) | ukb-b-20531 |
| rs17193211 | T | C | 0.066801 | -0.0154786 | 0.00260374 | 2.80E-09 | 0.08427557 | 35.34012852 | Leg fat percentage (right) | ukb-b-20531 |
| rs1724557 | A | C | 0.586759 | -0.00959286 | 0.00130232 | 1.80E-13 | 0.12380304 | 54.25762659 | Leg fat percentage (right) | ukb-b-20531 |
| rs17446091 | C | T | 0.20182 | 0.0109618 | 0.00159096 | 5.60E-12 | 0.110025097 | 47.47284142 | Leg fat percentage (right) | ukb-b-20531 |
| rs17522122 | T | G | 0.471213 | 0.0097298 | 0.00128448 | 3.60E-14 | 0.12999943 | 57.37902094 | Leg fat percentage (right) | ukb-b-20531 |
| rs17820064 | C | T | 0.295292 | 0.0094847 | 0.0013983 | 1.20E-11 | 0.106996246 | 46.00939073 | Leg fat percentage (right) | ukb-b-20531 |
| rs1782508 | G | C | 0.655502 | -0.00788508 | 0.00134166 | 4.20E-09 | 0.082525827 | 34.54039214 | Leg fat percentage (right) | ukb-b-20531 |
| rs1788808 | G | A | 0.494889 | -0.0122126 | 0.00127994 | 1.40E-21 | 0.191648729 | 91.04100484 | Leg fat percentage (right) | ukb-b-20531 |
| rs1801282 | G | C | 0.119535 | 0.0227694 | 0.00196076 | 3.60E-31 | 0.259903187 | 134.8510387 | Leg fat percentage (right) | ukb-b-20531 |
| rs1808629 | A | G | 0.685444 | -0.0132658 | 0.00138012 | 7.10E-22 | 0.193940701 | 92.39174972 | Leg fat percentage (right) | ukb-b-20531 |
| rs1861410 | T | C | 0.55537 | -0.0104151 | 0.00128533 | 5.40E-16 | 0.146020497 | 65.65950433 | Leg fat percentage (right) | ukb-b-20531 |
| rs1887340 | T | C | 0.234312 | -0.0104351 | 0.00151008 | 4.80E-12 | 0.110600927 | 47.75219279 | Leg fat percentage (right) | ukb-b-20531 |
| rs1928496 | T | C | 0.743607 | 0.0101388 | 0.0014619 | 4.10E-12 | 0.111315163 | 48.09919191 | Leg fat percentage (right) | ukb-b-20531 |
| rs1928957 | C | A | 0.441167 | 0.00715029 | 0.00129252 | 0.000000032 | 0.07381416 | 30.60361773 | Leg fat percentage (right) | ukb-b-20531 |
| rs1974991 | G | C | 0.500667 | -0.008244 | 0.00127718 | 1.10E-10 | 0.097882211 | 41.66503472 | Leg fat percentage (right) | ukb-b-20531 |
| rs1981612 | A | C | 0.455991 | 0.00732635 | 0.00129651 | 0.000000016 | 0.076771751 | 31.93181341 | Leg fat percentage (right) | ukb-b-20531 |
| rs2016962 | C | G | 0.037465 | 0.0193604 | 0.00351908 | 3.80E-08 | 0.073061731 | 30.26706937 | Leg fat percentage (right) | ukb-b-20531 |
| rs2034768 | G | A | 0.512161 | -0.010276 | 0.00127466 | 7.50E-16 | 0.144750787 | 64.99193633 | Leg fat percentage (right) | ukb-b-20531 |
| rs2034946 | G | T | 0.208814 | 0.00902717 | 0.00157362 | 9.70E-09 | 0.07893382 | 32.9081531 | Leg fat percentage (right) | ukb-b-20531 |
| rs2035936 | T | G | 0.055876 | 0.0192728 | 0.00281594 | 7.70E-12 | 0.108723643 | 46.84279876 | Leg fat percentage (right) | ukb-b-20531 |
| rs208477 | T | C | 0.188329 | 0.00901196 | 0.0016307 | 3.30E-08 | 0.073675375 | 30.54150038 | Leg fat percentage (right) | ukb-b-20531 |
| rs2108635 | G | A | 0.338512 | 0.00820608 | 0.0013535 | 1.30E-09 | 0.08736195 | 36.75826233 | Leg fat percentage (right) | ukb-b-20531 |
| rs215669 | A | G | 0.611667 | -0.00999432 | 0.00131516 | 3.00E-14 | 0.130729352 | 57.74964492 | Leg fat percentage (right) | ukb-b-20531 |
| rs2172131 | C | T | 0.578564 | -0.00955464 | 0.00129361 | 1.50E-13 | 0.124394 | 54.55341326 | Leg fat percentage (right) | ukb-b-20531 |
| rs217672 | C | A | 0.271727 | 0.00934974 | 0.00143972 | 8.40E-11 | 0.09895921 | 42.1738251 | Leg fat percentage (right) | ukb-b-20531 |
| rs2182717 | A | G | 0.691015 | 0.00790812 | 0.00138579 | 0.000000012 | 0.07817517 | 32.56504296 | Leg fat percentage (right) | ukb-b-20531 |
| rs2192158 | G | A | 0.553167 | -0.00731808 | 0.00128111 | 1.10E-08 | 0.078319622 | 32.6303298 | Leg fat percentage (right) | ukb-b-20531 |
| rs2216931 | A | C | 0.661955 | 0.0100008 | 0.00134791 | 1.20E-13 | 0.125381921 | 55.04877939 | Leg fat percentage (right) | ukb-b-20531 |
| rs2241743 | A | G | 0.573638 | -0.00911455 | 0.00128985 | 1.60E-12 | 0.115071789 | 49.9335045 | Leg fat percentage (right) | ukb-b-20531 |
| rs2254614 | C | T | 0.339236 | 0.0117136 | 0.00134341 | 2.80E-18 | 0.165265043 | 76.02625982 | Leg fat percentage (right) | ukb-b-20531 |
| rs2274224 | C | G | 0.435467 | -0.0122842 | 0.0012872 | 1.40E-21 | 0.19170747 | 91.07552744 | Leg fat percentage (right) | ukb-b-20531 |
| rs2287821 | T | C | 0.50883 | 0.0102326 | 0.00128053 | 1.30E-15 | 0.142578946 | 63.85464285 | Leg fat percentage (right) | ukb-b-20531 |
| rs2289379 | T | C | 0.395691 | -0.00930362 | 0.00131051 | 1.30E-12 | 0.116020378 | 50.39915427 | Leg fat percentage (right) | ukb-b-20531 |
| rs2301597 | C | T | 0.574335 | -0.00826421 | 0.00129444 | 1.70E-10 | 0.095961003 | 40.76043773 | Leg fat percentage (right) | ukb-b-20531 |
| rs2307111 | C | T | 0.395093 | -0.0140853 | 0.00130587 | 4.00E-27 | 0.232523222 | 116.3408716 | Leg fat percentage (right) | ukb-b-20531 |
| rs2605091 | G | C | 0.682331 | -0.012325 | 0.00136654 | 1.90E-19 | 0.174805223 | 81.34468074 | Leg fat percentage (right) | ukb-b-20531 |
| rs2613498 | T | C | 0.191129 | -0.017004 | 0.00161726 | 7.40E-26 | 0.223530056 | 110.545865 | Leg fat percentage (right) | ukb-b-20531 |
| rs2618062 | G | C | 0.946871 | 0.0159818 | 0.00285821 | 2.30E-08 | 0.075290024 | 31.26533701 | Leg fat percentage (right) | ukb-b-20531 |
| rs262956 | G | T | 0.646885 | -0.00748588 | 0.00133793 | 2.20E-08 | 0.075379176 | 31.305377 | Leg fat percentage (right) | ukb-b-20531 |
| rs2656512 | T | C | 0.654557 | 0.00776665 | 0.00134337 | 7.40E-09 | 0.080075073 | 33.42536649 | Leg fat percentage (right) | ukb-b-20531 |
| rs2660241 | C | T | 0.364839 | 0.00814122 | 0.0013284 | 8.90E-10 | 0.089096827 | 37.55962502 | Leg fat percentage (right) | ukb-b-20531 |
| rs2712667 | C | G | 0.643989 | -0.0073706 | 0.00133762 | 3.60E-08 | 0.073275626 | 30.3626853 | Leg fat percentage (right) | ukb-b-20531 |
| rs271590 | T | C | 0.312082 | 0.00794822 | 0.00138295 | 9.10E-09 | 0.079205996 | 33.03138635 | Leg fat percentage (right) | ukb-b-20531 |
| rs2725370 | C | T | 0.696116 | -0.0102564 | 0.00139289 | 1.80E-13 | 0.123726996 | 54.21959373 | Leg fat percentage (right) | ukb-b-20531 |
| rs2726036 | C | A | 0.398071 | 0.014 | 0.00130726 | 9.20E-27 | 0.229985235 | 114.6917359 | Leg fat percentage (right) | ukb-b-20531 |
| rs2727340 | A | T | 0.331183 | 0.009965 | 0.00134644 | 1.40E-13 | 0.124835741 | 54.77477418 | Leg fat percentage (right) | ukb-b-20531 |
| rs2731238 | T | G | 0.06753 | -0.0173262 | 0.00279056 | 5.30E-10 | 0.091231718 | 38.54995869 | Leg fat percentage (right) | ukb-b-20531 |
| rs2731238 | T | G | 0.06753 | -0.0173262 | 0.00279056 | 5.30E-10 | 0.091231718 | 38.54995869 | Leg fat percentage (right) | ukb-b-20531 |
| rs2733296 | G | A | 0.478647 | 0.00921008 | 0.001282 | 6.80E-13 | 0.1184816 | 51.61200786 | Leg fat percentage (right) | ukb-b-20531 |
| rs274111 | G | A | 0.649679 | 0.00751327 | 0.00135064 | 2.70E-08 | 0.074574288 | 30.94416565 | Leg fat percentage (right) | ukb-b-20531 |
| rs2794665 | G | A | 0.659437 | 0.00747678 | 0.00135092 | 3.10E-08 | 0.073876706 | 30.63161827 | Leg fat percentage (right) | ukb-b-20531 |
| rs279789 | A | G | 0.496737 | 0.00757306 | 0.00127671 | 3.00E-09 | 0.083936814 | 35.18505832 | Leg fat percentage (right) | ukb-b-20531 |
| rs2798297 | A | G | 0.366714 | 0.00829968 | 0.00133412 | 4.90E-10 | 0.091558496 | 38.70195528 | Leg fat percentage (right) | ukb-b-20531 |
| rs2802295 | G | A | 0.626183 | 0.00875798 | 0.00131886 | 3.10E-11 | 0.103007339 | 44.09714827 | Leg fat percentage (right) | ukb-b-20531 |
| rs28366151 | T | C | 0.118912 | -0.0143164 | 0.00196989 | 3.70E-13 | 0.120915774 | 52.8182122 | Leg fat percentage (right) | ukb-b-20531 |
| rs28431181 | A | G | 0.122957 | 0.0137101 | 0.00194144 | 1.60E-12 | 0.114940842 | 49.86930302 | Leg fat percentage (right) | ukb-b-20531 |
| rs28457808 | G | C | 0.163078 | -0.0126715 | 0.00173149 | 2.50E-13 | 0.122400013 | 53.55698008 | Leg fat percentage (right) | ukb-b-20531 |
| rs28475375 | A | G | 0.143045 | 0.0100231 | 0.00182756 | 4.10E-08 | 0.072640332 | 30.07882303 | Leg fat percentage (right) | ukb-b-20531 |
| rs2861685 | C | T | 0.412117 | -0.00872997 | 0.00129081 | 1.30E-11 | 0.106437479 | 45.74049498 | Leg fat percentage (right) | ukb-b-20531 |
| rs28711392 | C | T | 0.36736 | -0.00925749 | 0.00133502 | 4.10E-12 | 0.111286279 | 48.08514825 | Leg fat percentage (right) | ukb-b-20531 |
| rs28726372 | C | T | 0.307248 | 0.00791234 | 0.00138093 | 0.00000001 | 0.078760402 | 32.82967269 | Leg fat percentage (right) | ukb-b-20531 |
| rs28878886 | G | T | 0.512242 | -0.00735234 | 0.00128211 | 9.80E-09 | 0.078883124 | 32.88520772 | Leg fat percentage (right) | ukb-b-20531 |
| rs2929458 | C | G | 0.188533 | -0.009659 | 0.0016405 | 3.90E-09 | 0.082802509 | 34.66664895 | Leg fat percentage (right) | ukb-b-20531 |
| rs2954033 | G | A | 0.695201 | 0.00853083 | 0.00138593 | 7.50E-10 | 0.089805506 | 37.88785196 | Leg fat percentage (right) | ukb-b-20531 |
| rs302189 | C | A | 0.777518 | -0.00937638 | 0.00153848 | 1.10E-09 | 0.088197494 | 37.1438306 | Leg fat percentage (right) | ukb-b-20531 |
| rs3087523 | A | G | 0.12462 | 0.0110542 | 0.00193926 | 0.000000012 | 0.078014516 | 32.49245727 | Leg fat percentage (right) | ukb-b-20531 |
| rs308911 | G | A | 0.714421 | -0.00906265 | 0.00141277 | 1.40E-10 | 0.096788895 | 41.14977713 | Leg fat percentage (right) | ukb-b-20531 |
| rs3094904 | C | T | 0.198162 | -0.0108783 | 0.00160895 | 1.40E-11 | 0.106379702 | 45.71270984 | Leg fat percentage (right) | ukb-b-20531 |
| rs34045288 | T | C | 0.334423 | 0.0128684 | 0.00135208 | 1.80E-21 | 0.190867771 | 90.58250497 | Leg fat percentage (right) | ukb-b-20531 |
| rs34117444 | A | G | 0.325553 | 0.00874218 | 0.00136588 | 1.50E-10 | 0.096396462 | 40.9651356 | Leg fat percentage (right) | ukb-b-20531 |
| rs34325 | C | T | 0.52397 | -0.00814948 | 0.00128022 | 1.90E-10 | 0.095453181 | 40.52197286 | Leg fat percentage (right) | ukb-b-20531 |
| rs34769775 | T | C | 0.297428 | -0.00951242 | 0.00139843 | 1.00E-11 | 0.107537365 | 46.2701141 | Leg fat percentage (right) | ukb-b-20531 |
| rs34811474 | A | G | 0.230825 | -0.0128538 | 0.00151354 | 2.00E-17 | 0.158122278 | 72.12324697 | Leg fat percentage (right) | ukb-b-20531 |
| rs34930419 | C | T | 0.033187 | 0.0222426 | 0.00360059 | 6.50E-10 | 0.090395183 | 38.16135278 | Leg fat percentage (right) | ukb-b-20531 |
| rs35445661 | G | T | 0.041434 | 0.0201198 | 0.00321833 | 4.10E-10 | 0.092376341 | 39.08284511 | Leg fat percentage (right) | ukb-b-20531 |
| rs35513882 | C | G | 0.153143 | 0.0107394 | 0.00177967 | 1.60E-09 | 0.086616934 | 36.41506401 | Leg fat percentage (right) | ukb-b-20531 |
| rs35792595 | A | T | 0.297366 | 0.00831504 | 0.00140362 | 3.10E-09 | 0.083737197 | 35.09373481 | Leg fat percentage (right) | ukb-b-20531 |
| rs35961581 | G | A | 0.122548 | -0.01067 | 0.00194488 | 4.10E-08 | 0.072684144 | 30.09838674 | Leg fat percentage (right) | ukb-b-20531 |
| rs372519 | A | G | 0.536403 | 0.00998348 | 0.00128701 | 8.70E-15 | 0.135471557 | 60.17277796 | Leg fat percentage (right) | ukb-b-20531 |
| rs3733347 | C | T | 0.791407 | -0.00885319 | 0.00157129 | 1.80E-08 | 0.076358776 | 31.7458439 | Leg fat percentage (right) | ukb-b-20531 |
| rs3737992 | A | G | 0.168986 | -0.0107601 | 0.00169876 | 2.40E-10 | 0.094597375 | 40.12070561 | Leg fat percentage (right) | ukb-b-20531 |
| rs3749473 | C | T | 0.524408 | 0.00853594 | 0.00127681 | 2.30E-11 | 0.104256383 | 44.69409591 | Leg fat percentage (right) | ukb-b-20531 |
| rs3754963 | T | A | 0.255087 | -0.0106736 | 0.00146051 | 2.70E-13 | 0.1221027 | 53.40879466 | Leg fat percentage (right) | ukb-b-20531 |
| rs3764002 | T | C | 0.26151 | -0.0162067 | 0.00145192 | 6.20E-29 | 0.244980176 | 124.595917 | Leg fat percentage (right) | ukb-b-20531 |
| rs3766823 | A | G | 0.172011 | 0.012602 | 0.00168872 | 8.50E-14 | 0.126653964 | 55.68826107 | Leg fat percentage (right) | ukb-b-20531 |
| rs3782812 | G | C | 0.098204 | -0.0184709 | 0.00215219 | 9.30E-18 | 0.160943947 | 73.65714747 | Leg fat percentage (right) | ukb-b-20531 |
| rs3803286 | G | A | 0.666747 | -0.0104202 | 0.00135466 | 1.40E-14 | 0.133512672 | 59.16862743 | Leg fat percentage (right) | ukb-b-20531 |
| rs40071 | C | T | 0.17949 | -0.0142492 | 0.00166743 | 1.30E-17 | 0.159787764 | 73.02738383 | Leg fat percentage (right) | ukb-b-20531 |
| rs429343 | G | A | 0.576563 | -0.00884889 | 0.00129062 | 7.10E-12 | 0.109067283 | 47.00897819 | Leg fat percentage (right) | ukb-b-20531 |
| rs429358 | C | T | 0.154044 | -0.0181307 | 0.00177146 | 1.40E-24 | 0.214327063 | 104.7529937 | Leg fat percentage (right) | ukb-b-20531 |
| rs4307239 | G | A | 0.458848 | 0.0070178 | 0.00128366 | 4.60E-08 | 0.07221356 | 29.88835118 | Leg fat percentage (right) | ukb-b-20531 |
| rs4357530 | A | G | 0.67876 | -0.00876573 | 0.00136843 | 1.50E-10 | 0.096540284 | 41.03278605 | Leg fat percentage (right) | ukb-b-20531 |
| rs4398538 | C | T | 0.642508 | -0.00778797 | 0.00133413 | 5.30E-09 | 0.081507337 | 34.07628483 | Leg fat percentage (right) | ukb-b-20531 |
| rs445077 | A | T | 0.48013 | 0.00886071 | 0.00128045 | 4.50E-12 | 0.110877216 | 47.88635677 | Leg fat percentage (right) | ukb-b-20531 |
| rs4482463 | A | C | 0.923052 | -0.0167413 | 0.00239585 | 2.80E-12 | 0.112809291 | 48.82689523 | Leg fat percentage (right) | ukb-b-20531 |
| rs4528240 | T | C | 0.342005 | -0.00796221 | 0.00136305 | 5.20E-09 | 0.081609349 | 34.12272305 | Leg fat percentage (right) | ukb-b-20531 |
| rs4547574 | T | A | 0.227699 | -0.00852487 | 0.00151989 | 0.00000002 | 0.075722085 | 31.45945645 | Leg fat percentage (right) | ukb-b-20531 |
| rs4561038 | C | T | 0.10825 | -0.0115915 | 0.00206067 | 1.90E-08 | 0.076127764 | 31.6418875 | Leg fat percentage (right) | ukb-b-20531 |
| rs4672338 | T | C | 0.336286 | 0.00789339 | 0.00134845 | 4.80E-09 | 0.081922907 | 34.2655279 | Leg fat percentage (right) | ukb-b-20531 |
| rs4673617 | T | C | 0.271768 | 0.00882539 | 0.00143577 | 7.90E-10 | 0.089579544 | 37.78314137 | Leg fat percentage (right) | ukb-b-20531 |
| rs4759318 | T | C | 0.362188 | 0.00767909 | 0.00133081 | 7.90E-09 | 0.079789098 | 33.29564282 | Leg fat percentage (right) | ukb-b-20531 |
| rs4762951 | G | A | 0.780431 | -0.0104006 | 0.00154275 | 1.60E-11 | 0.105831253 | 45.44914084 | Leg fat percentage (right) | ukb-b-20531 |
| rs479018 | A | G | 0.332506 | -0.0115979 | 0.00137789 | 3.90E-17 | 0.155762539 | 70.84833088 | Leg fat percentage (right) | ukb-b-20531 |
| rs4790292 | A | C | 0.153744 | -0.0155801 | 0.0017783 | 1.90E-18 | 0.166592871 | 76.75919734 | Leg fat percentage (right) | ukb-b-20531 |
| rs4808762 | C | T | 0.290963 | 0.0146424 | 0.0014071 | 2.30E-25 | 0.219966537 | 108.2865724 | Leg fat percentage (right) | ukb-b-20531 |
| rs4820323 | G | C | 0.58097 | -0.010038 | 0.00129854 | 1.10E-14 | 0.134660178 | 59.75630269 | Leg fat percentage (right) | ukb-b-20531 |
| rs4876611 | G | A | 0.72019 | 0.011491 | 0.00142306 | 6.80E-16 | 0.145153108 | 65.20324745 | Leg fat percentage (right) | ukb-b-20531 |
| rs4877285 | A | G | 0.681905 | -0.00805355 | 0.00137885 | 5.20E-09 | 0.081591573 | 34.1146305 | Leg fat percentage (right) | ukb-b-20531 |
| rs4894808 | C | G | 0.400136 | -0.00983161 | 0.00132874 | 1.40E-13 | 0.124782589 | 54.74812714 | Leg fat percentage (right) | ukb-b-20531 |
| rs4897299 | C | G | 0.576774 | 0.00719988 | 0.00129161 | 0.000000025 | 0.074862253 | 31.07332438 | Leg fat percentage (right) | ukb-b-20531 |
| rs494048 | C | T | 0.537584 | 0.00843315 | 0.00130384 | 9.90E-11 | 0.098240497 | 41.83415965 | Leg fat percentage (right) | ukb-b-20531 |
| rs4958702 | C | T | 0.572319 | -0.00852363 | 0.00128863 | 3.70E-11 | 0.102282465 | 43.75147541 | Leg fat percentage (right) | ukb-b-20531 |
| rs4979236 | A | G | 0.219887 | 0.0115946 | 0.00154911 | 7.20E-14 | 0.127313398 | 56.02050587 | Leg fat percentage (right) | ukb-b-20531 |
| rs5021156 | C | G | 0.171754 | 0.00982695 | 0.00169375 | 6.60E-09 | 0.080596094 | 33.66191944 | Leg fat percentage (right) | ukb-b-20531 |
| rs525101 | C | T | 0.370373 | 0.00956991 | 0.00132547 | 5.20E-13 | 0.119525716 | 52.12858084 | Leg fat percentage (right) | ukb-b-20531 |
| rs539515 | C | A | 0.204983 | 0.0223485 | 0.00157805 | 1.60E-45 | 0.343101328 | 200.5650422 | Leg fat percentage (right) | ukb-b-20531 |
| rs55707359 | G | T | 0.015449 | 0.0290641 | 0.00524363 | 3.00E-08 | 0.07407857 | 30.72201375 | Leg fat percentage (right) | ukb-b-20531 |
| rs561136 | T | C | 0.8598 | -0.0109902 | 0.0018347 | 2.10E-09 | 0.08545814 | 35.88236583 | Leg fat percentage (right) | ukb-b-20531 |
| rs56218501 | T | C | 0.211815 | -0.00913883 | 0.00156305 | 0.000000005 | 0.081746095 | 34.18498989 | Leg fat percentage (right) | ukb-b-20531 |
| rs56260582 | G | A | 0.138379 | -0.012631 | 0.00184636 | 7.90E-12 | 0.108634395 | 46.7996607 | Leg fat percentage (right) | ukb-b-20531 |
| rs56328878 | A | C | 0.267181 | -0.00803168 | 0.00143813 | 2.30E-08 | 0.075122414 | 31.19008095 | Leg fat percentage (right) | ukb-b-20531 |
| rs56356382 | C | T | 0.192343 | -0.0159189 | 0.00162795 | 1.40E-22 | 0.199364417 | 95.61895323 | Leg fat percentage (right) | ukb-b-20531 |
| rs56399737 | T | C | 0.449167 | -0.00944722 | 0.00128871 | 2.30E-13 | 0.122766939 | 53.73999986 | Leg fat percentage (right) | ukb-b-20531 |
| rs56803094 | G | A | 0.226675 | -0.00904306 | 0.00153144 | 3.50E-09 | 0.083244079 | 34.86830627 | Leg fat percentage (right) | ukb-b-20531 |
| rs57241669 | G | A | 0.076164 | -0.0131601 | 0.00241033 | 4.80E-08 | 0.072038414 | 29.81023271 | Leg fat percentage (right) | ukb-b-20531 |
| rs57800857 | C | A | 0.364894 | -0.0107864 | 0.0013329 | 5.80E-16 | 0.145693562 | 65.48742395 | Leg fat percentage (right) | ukb-b-20531 |
| rs57803 | A | G | 0.824112 | -0.0104241 | 0.00168003 | 5.50E-10 | 0.091120892 | 38.498434 | Leg fat percentage (right) | ukb-b-20531 |
| rs58120873 | A | G | 0.086867 | -0.0137118 | 0.00227682 | 1.70E-09 | 0.086298756 | 36.26866288 | Leg fat percentage (right) | ukb-b-20531 |
| rs58431213 | A | G | 0.217583 | -0.0100462 | 0.00156891 | 1.50E-10 | 0.096475349 | 41.00223944 | Leg fat percentage (right) | ukb-b-20531 |
| rs58582157 | C | G | 0.296893 | 0.00839741 | 0.00139898 | 1.90E-09 | 0.085780209 | 36.03028557 | Leg fat percentage (right) | ukb-b-20531 |
| rs58862095 | T | C | 0.419274 | -0.0106755 | 0.00129664 | 1.80E-16 | 0.150039362 | 67.78562735 | Leg fat percentage (right) | ukb-b-20531 |
| rs588660 | A | G | 0.584207 | 0.0103856 | 0.00129279 | 9.50E-16 | 0.143882894 | 64.53676848 | Leg fat percentage (right) | ukb-b-20531 |
| rs58948644 | A | G | 0.582747 | 0.00980946 | 0.00129311 | 3.30E-14 | 0.130329512 | 57.54654583 | Leg fat percentage (right) | ukb-b-20531 |
| rs59086897 | A | T | 0.487582 | 0.0183956 | 0.00127276 | 2.40E-47 | 0.352334105 | 208.8982872 | Leg fat percentage (right) | ukb-b-20531 |
| rs59104534 | T | C | 0.298468 | 0.00843876 | 0.00140014 | 1.70E-09 | 0.086422807 | 36.32572956 | Leg fat percentage (right) | ukb-b-20531 |
| rs59346947 | A | G | 0.203206 | 0.0100827 | 0.00158528 | 2.00E-10 | 0.095304406 | 40.45216124 | Leg fat percentage (right) | ukb-b-20531 |
| rs59499656 | T | A | 0.343215 | -0.00976696 | 0.00134956 | 4.60E-13 | 0.120025442 | 52.37625267 | Leg fat percentage (right) | ukb-b-20531 |
| rs6011058 | T | C | 0.67462 | 0.00988618 | 0.00136382 | 4.20E-13 | 0.120368407 | 52.5463939 | Leg fat percentage (right) | ukb-b-20531 |
| rs6021948 | A | T | 0.321778 | -0.0110443 | 0.0013709 | 7.90E-16 | 0.144581365 | 64.9030098 | Leg fat percentage (right) | ukb-b-20531 |
| rs6103254 | C | T | 0.126695 | -0.0134997 | 0.00192814 | 2.50E-12 | 0.113204424 | 49.01975159 | Leg fat percentage (right) | ukb-b-20531 |
| rs6138536 | G | A | 0.462459 | 0.00955026 | 0.00128232 | 9.50E-14 | 0.126214987 | 55.467368 | Leg fat percentage (right) | ukb-b-20531 |
| rs61734410 | T | C | 0.255218 | -0.00864218 | 0.00149905 | 8.20E-09 | 0.079658506 | 33.23643066 | Leg fat percentage (right) | ukb-b-20531 |
| rs61754230 | T | C | 0.01971 | 0.0259598 | 0.00458951 | 0.000000015 | 0.076910009 | 31.99411073 | Leg fat percentage (right) | ukb-b-20531 |
| rs61782665 | G | A | 0.584629 | -0.00896294 | 0.00129394 | 4.30E-12 | 0.111072789 | 47.98137631 | Leg fat percentage (right) | ukb-b-20531 |
| rs61826925 | T | C | 0.110803 | 0.0115926 | 0.00202773 | 1.10E-08 | 0.078439377 | 32.68447013 | Leg fat percentage (right) | ukb-b-20531 |
| rs61985887 | T | C | 0.082364 | 0.0139992 | 0.00232465 | 1.70E-09 | 0.086291447 | 36.26530105 | Leg fat percentage (right) | ukb-b-20531 |
| rs62107261 | C | T | 0.048318 | -0.0350222 | 0.00297806 | 6.30E-32 | 0.264789356 | 138.2992933 | Leg fat percentage (right) | ukb-b-20531 |
| rs62124758 | T | C | 0.143904 | 0.0100799 | 0.00184123 | 4.40E-08 | 0.072398038 | 29.97066383 | Leg fat percentage (right) | ukb-b-20531 |
| rs62183912 | A | G | 0.556083 | 0.00849159 | 0.00128083 | 3.40E-11 | 0.102706589 | 43.95366071 | Leg fat percentage (right) | ukb-b-20531 |
| rs62218301 | G | A | 0.166063 | -0.011275 | 0.00172735 | 6.70E-11 | 0.099872354 | 42.60616181 | Leg fat percentage (right) | ukb-b-20531 |
| rs62261725 | G | A | 0.3262 | -0.0114549 | 0.0013622 | 4.10E-17 | 0.155511759 | 70.71325888 | Leg fat percentage (right) | ukb-b-20531 |
| rs62393181 | A | G | 0.083719 | -0.0128908 | 0.00231448 | 2.60E-08 | 0.074745151 | 31.02079161 | Leg fat percentage (right) | ukb-b-20531 |
| rs62443626 | A | G | 0.46398 | -0.00765616 | 0.00128597 | 2.60E-09 | 0.084505476 | 35.44543637 | Leg fat percentage (right) | ukb-b-20531 |
| rs62508259 | C | T | 0.335205 | -0.00801227 | 0.00135197 | 3.10E-09 | 0.083798601 | 35.12182237 | Leg fat percentage (right) | ukb-b-20531 |
| rs6265 | T | C | 0.188491 | -0.017985 | 0.00163174 | 3.00E-28 | 0.240331988 | 121.4839669 | Leg fat percentage (right) | ukb-b-20531 |
| rs6433672 | A | C | 0.842492 | -0.00977673 | 0.00174651 | 2.20E-08 | 0.075447595 | 31.3361105 | Leg fat percentage (right) | ukb-b-20531 |
| rs6441176 | G | T | 0.560286 | -0.00907954 | 0.001282 | 1.40E-12 | 0.115532059 | 50.15932022 | Leg fat percentage (right) | ukb-b-20531 |
| rs6478538 | G | A | 0.677179 | -0.00919041 | 0.00136622 | 1.70E-11 | 0.105418576 | 45.25103277 | Leg fat percentage (right) | ukb-b-20531 |
| rs6480742 | G | C | 0.155162 | 0.0101217 | 0.00176037 | 8.90E-09 | 0.079268524 | 33.05970732 | Leg fat percentage (right) | ukb-b-20531 |
| rs6495017 | A | T | 0.238293 | -0.0112532 | 0.00150272 | 7.00E-14 | 0.127428288 | 56.07844234 | Leg fat percentage (right) | ukb-b-20531 |
| rs6540497 | C | G | 0.578935 | -0.00748781 | 0.00129219 | 6.80E-09 | 0.080411674 | 33.57815891 | Leg fat percentage (right) | ukb-b-20531 |
| rs6551304 | G | A | 0.831906 | 0.0102356 | 0.00170858 | 2.10E-09 | 0.08547162 | 35.88855517 | Leg fat percentage (right) | ukb-b-20531 |
| rs6561937 | A | T | 0.753693 | -0.0119244 | 0.00148711 | 1.10E-15 | 0.143423924 | 64.29643354 | Leg fat percentage (right) | ukb-b-20531 |
| rs6575340 | A | G | 0.636027 | 0.01045 | 0.00133104 | 4.10E-15 | 0.138314559 | 61.63825951 | Leg fat percentage (right) | ukb-b-20531 |
| rs6602997 | T | C | 0.711837 | 0.0170525 | 0.00141303 | 1.60E-33 | 0.274975871 | 145.6375457 | Leg fat percentage (right) | ukb-b-20531 |
| rs663129 | A | G | 0.233332 | 0.0198016 | 0.00150955 | 2.60E-39 | 0.309439657 | 172.0701597 | Leg fat percentage (right) | ukb-b-20531 |
| rs6674490 | A | C | 0.410268 | -0.00739217 | 0.00129731 | 0.000000012 | 0.077960508 | 32.46806153 | Leg fat percentage (right) | ukb-b-20531 |
| rs672187 | A | C | 0.265547 | -0.0107984 | 0.00148416 | 3.40E-13 | 0.121154288 | 52.93676239 | Leg fat percentage (right) | ukb-b-20531 |
| rs6740162 | C | T | 0.379309 | 0.00902751 | 0.00131585 | 6.90E-12 | 0.109188697 | 47.06772277 | Leg fat percentage (right) | ukb-b-20531 |
| rs6744646 | G | A | 0.828315 | 0.0217719 | 0.00168764 | 4.50E-38 | 0.3023643 | 166.4305472 | Leg fat percentage (right) | ukb-b-20531 |
| rs67609008 | C | T | 0.283592 | 0.0092557 | 0.00142153 | 7.50E-11 | 0.099424906 | 42.39420351 | Leg fat percentage (right) | ukb-b-20531 |
| rs6782581 | G | C | 0.43929 | -0.00718979 | 0.00128493 | 2.20E-08 | 0.075387928 | 31.30930832 | Leg fat percentage (right) | ukb-b-20531 |
| rs6860245 | C | G | 0.247906 | -0.0197355 | 0.00148067 | 1.60E-40 | 0.316307408 | 177.6559319 | Leg fat percentage (right) | ukb-b-20531 |
| rs6862472 | G | C | 0.220173 | -0.0104071 | 0.0015401 | 1.40E-11 | 0.106275696 | 45.66270275 | Leg fat percentage (right) | ukb-b-20531 |
| rs6904567 | G | T | 0.372374 | -0.00731245 | 0.00131744 | 2.80E-08 | 0.074270604 | 30.80804418 | Leg fat percentage (right) | ukb-b-20531 |
| rs6938973 | C | T | 0.601538 | 0.0144538 | 0.00130376 | 1.50E-28 | 0.242461278 | 122.9047809 | Leg fat percentage (right) | ukb-b-20531 |
| rs6945561 | C | T | 0.799715 | 0.00926397 | 0.0015952 | 6.30E-09 | 0.080736991 | 33.72593495 | Leg fat percentage (right) | ukb-b-20531 |
| rs6977416 | A | G | 0.33424 | -0.0100609 | 0.00136477 | 1.70E-13 | 0.123976473 | 54.34439146 | Leg fat percentage (right) | ukb-b-20531 |
| rs698838 | T | C | 0.625175 | 0.00798348 | 0.00131325 | 1.20E-09 | 0.087791516 | 36.95640045 | Leg fat percentage (right) | ukb-b-20531 |
| rs7027096 | T | G | 0.664778 | 0.00746055 | 0.00135662 | 3.80E-08 | 0.073007933 | 30.24302719 | Leg fat percentage (right) | ukb-b-20531 |
| rs7027096 | T | G | 0.664778 | 0.00746055 | 0.00135662 | 3.80E-08 | 0.073007933 | 30.24302719 | Leg fat percentage (right) | ukb-b-20531 |
| rs7027304 | T | C | 0.652645 | 0.00943959 | 0.00134717 | 2.40E-12 | 0.1133641 | 49.09773495 | Leg fat percentage (right) | ukb-b-20531 |
| rs7097348 | T | C | 0.716775 | 0.00823685 | 0.00142024 | 6.60E-09 | 0.080538095 | 33.63557329 | Leg fat percentage (right) | ukb-b-20531 |
| rs7117842 | C | T | 0.369021 | 0.00959042 | 0.00132929 | 5.40E-13 | 0.119370687 | 52.05180314 | Leg fat percentage (right) | ukb-b-20531 |
| rs7124681 | A | C | 0.408396 | 0.0151222 | 0.0012958 | 1.80E-31 | 0.261812094 | 136.1927546 | Leg fat percentage (right) | ukb-b-20531 |
| rs7132908 | A | G | 0.384442 | 0.0139928 | 0.0013129 | 1.60E-26 | 0.228282687 | 113.591532 | Leg fat percentage (right) | ukb-b-20531 |
| rs719802 | C | T | 0.614412 | -0.00956129 | 0.00131051 | 3.00E-13 | 0.12174268 | 53.22948976 | Leg fat percentage (right) | ukb-b-20531 |
| rs7206608 | G | C | 0.321559 | 0.00936746 | 0.00136829 | 7.60E-12 | 0.108778222 | 46.86918359 | Leg fat percentage (right) | ukb-b-20531 |
| rs7213608 | T | C | 0.675271 | -0.0128328 | 0.00137869 | 1.30E-20 | 0.184086692 | 86.63823586 | Leg fat percentage (right) | ukb-b-20531 |
| rs7218014 | C | T | 0.197302 | 0.0145377 | 0.00160828 | 1.60E-19 | 0.17545016 | 81.70865893 | Leg fat percentage (right) | ukb-b-20531 |
| rs72634826 | A | G | 0.25988 | -0.0117763 | 0.00147322 | 1.30E-15 | 0.142660606 | 63.8973003 | Leg fat percentage (right) | ukb-b-20531 |
| rs72651329 | G | C | 0.339005 | 0.00822361 | 0.00134769 | 0.000000001 | 0.088393659 | 37.23445489 | Leg fat percentage (right) | ukb-b-20531 |
| rs72823964 | T | C | 0.132046 | -0.0120233 | 0.00188571 | 1.80E-10 | 0.095733269 | 40.65346418 | Leg fat percentage (right) | ukb-b-20531 |
| rs72892910 | T | G | 0.172202 | 0.0173367 | 0.001692 | 1.20E-24 | 0.214701739 | 104.9861841 | Leg fat percentage (right) | ukb-b-20531 |
| rs72917533 | C | T | 0.185516 | -0.0108024 | 0.00164251 | 4.80E-11 | 0.101236824 | 43.25381961 | Leg fat percentage (right) | ukb-b-20531 |
| rs73026725 | A | C | 0.153578 | -0.011519 | 0.00177518 | 8.60E-11 | 0.098815947 | 42.10607541 | Leg fat percentage (right) | ukb-b-20531 |
| rs7308788 | A | G | 0.697905 | 0.00811147 | 0.00139714 | 6.40E-09 | 0.08069518 | 33.70693622 | Leg fat percentage (right) | ukb-b-20531 |
| rs73091318 | C | T | 0.102621 | 0.0124987 | 0.00212976 | 4.40E-09 | 0.082306614 | 34.44041341 | Leg fat percentage (right) | ukb-b-20531 |
| rs74278876 | T | C | 0.145088 | -0.0127473 | 0.00181604 | 2.20E-12 | 0.113717324 | 49.27034389 | Leg fat percentage (right) | ukb-b-20531 |
| rs7442885 | G | C | 0.214124 | -0.0126955 | 0.00155782 | 3.70E-16 | 0.147452584 | 66.41483059 | Leg fat percentage (right) | ukb-b-20531 |
| rs7463186 | G | A | 0.516034 | 0.00807388 | 0.0012766 | 2.50E-10 | 0.094338621 | 39.99953111 | Leg fat percentage (right) | ukb-b-20531 |
| rs75035127 | G | A | 0.030384 | -0.0251893 | 0.00371402 | 1.20E-11 | 0.10697364 | 45.99850548 | Leg fat percentage (right) | ukb-b-20531 |
| rs7537581 | A | C | 0.531723 | 0.00817203 | 0.00128634 | 2.10E-10 | 0.095107372 | 40.35973958 | Leg fat percentage (right) | ukb-b-20531 |
| rs75435178 | A | G | 0.210055 | -0.00928196 | 0.00156912 | 3.30E-09 | 0.083514422 | 34.99186331 | Leg fat percentage (right) | ukb-b-20531 |
| rs75499503 | T | C | 0.22014 | -0.0157559 | 0.00156165 | 6.20E-24 | 0.209540288 | 101.7932594 | Leg fat percentage (right) | ukb-b-20531 |
| rs75557510 | G | A | 0.060857 | -0.0182279 | 0.00270175 | 1.50E-11 | 0.105974557 | 45.51797725 | Leg fat percentage (right) | ukb-b-20531 |
| rs75695257 | T | C | 0.102038 | 0.0120889 | 0.00215318 | 0.00000002 | 0.075861066 | 31.52193693 | Leg fat percentage (right) | ukb-b-20531 |
| rs7575523 | G | T | 0.603245 | -0.0116467 | 0.0013061 | 4.80E-19 | 0.171549108 | 79.51570593 | Leg fat percentage (right) | ukb-b-20531 |
| rs7636464 | C | T | 0.58293 | 0.0073144 | 0.00129165 | 0.000000015 | 0.077073259 | 32.06769298 | Leg fat percentage (right) | ukb-b-20531 |
| rs764729 | C | A | 0.728017 | 0.00841878 | 0.00143337 | 4.30E-09 | 0.082430763 | 34.49702951 | Leg fat percentage (right) | ukb-b-20531 |
| rs7655064 | C | T | 0.124895 | 0.0108478 | 0.00193072 | 1.90E-08 | 0.075963126 | 31.56783139 | Leg fat percentage (right) | ukb-b-20531 |
| rs7665222 | G | A | 0.527401 | -0.00792018 | 0.00128491 | 7.10E-10 | 0.090036279 | 37.99484573 | Leg fat percentage (right) | ukb-b-20531 |
| rs7667864 | C | A | 0.714547 | 0.00837391 | 0.0014145 | 3.20E-09 | 0.083634979 | 35.04698593 | Leg fat percentage (right) | ukb-b-20531 |
| rs7696754 | T | G | 0.393532 | -0.00760826 | 0.00130501 | 5.50E-09 | 0.081316332 | 33.98936193 | Leg fat percentage (right) | ukb-b-20531 |
| rs770082 | A | G | 0.436966 | 0.00912993 | 0.0012924 | 1.60E-12 | 0.115012937 | 49.90464802 | Leg fat percentage (right) | ukb-b-20531 |
| rs7704382 | G | C | 0.43341 | 0.0083038 | 0.00128923 | 1.20E-10 | 0.097500876 | 41.48517775 | Leg fat percentage (right) | ukb-b-20531 |
| rs7707394 | A | G | 0.357293 | -0.0099691 | 0.00132953 | 6.50E-14 | 0.127715229 | 56.22320801 | Leg fat percentage (right) | ukb-b-20531 |
| rs77221192 | A | G | 0.018802 | -0.0280628 | 0.00470169 | 2.40E-09 | 0.084897077 | 35.62493003 | Leg fat percentage (right) | ukb-b-20531 |
| rs7739335 | T | C | 0.778406 | -0.0162312 | 0.00153581 | 4.20E-26 | 0.225327095 | 111.6930824 | Leg fat percentage (right) | ukb-b-20531 |
| rs7774 | A | C | 0.310475 | 0.00932509 | 0.00138884 | 1.90E-11 | 0.10506583 | 45.08183984 | Leg fat percentage (right) | ukb-b-20531 |
| rs78081111 | C | T | 0.542727 | -0.00731571 | 0.0012818 | 1.10E-08 | 0.078195212 | 32.57410023 | Leg fat percentage (right) | ukb-b-20531 |
| rs785510 | C | T | 0.704564 | 0.00950926 | 0.00139665 | 9.90E-12 | 0.107718199 | 46.35731486 | Leg fat percentage (right) | ukb-b-20531 |
| rs7893571 | T | G | 0.665923 | 0.00785718 | 0.00135658 | 7.00E-09 | 0.080341162 | 33.54614231 | Leg fat percentage (right) | ukb-b-20531 |
| rs7925725 | C | A | 0.411252 | 0.00866977 | 0.00129894 | 2.50E-11 | 0.103952895 | 44.54889856 | Leg fat percentage (right) | ukb-b-20531 |
| rs7927346 | T | C | 0.424346 | 0.00737726 | 0.00129709 | 1.30E-08 | 0.077695037 | 32.34818791 | Leg fat percentage (right) | ukb-b-20531 |
| rs7945659 | T | A | 0.446673 | -0.0108197 | 0.00128398 | 3.60E-17 | 0.156060808 | 71.00908564 | Leg fat percentage (right) | ukb-b-20531 |
| rs7957882 | A | C | 0.665426 | 0.00748288 | 0.00135718 | 3.50E-08 | 0.073357363 | 30.39923532 | Leg fat percentage (right) | ukb-b-20531 |
| rs7975187 | G | A | 0.213878 | 0.00921074 | 0.00155746 | 3.30E-09 | 0.083477098 | 34.9748003 | Leg fat percentage (right) | ukb-b-20531 |
| rs79847714 | C | T | 0.228217 | -0.0112816 | 0.00151875 | 1.10E-13 | 0.125639985 | 55.17836311 | Leg fat percentage (right) | ukb-b-20531 |
| rs7987928 | A | G | 0.799885 | -0.0108055 | 0.0015988 | 1.40E-11 | 0.106306284 | 45.67740849 | Leg fat percentage (right) | ukb-b-20531 |
| rs80135274 | T | A | 0.070311 | 0.0153227 | 0.00250494 | 9.50E-10 | 0.08878984 | 37.41760119 | Leg fat percentage (right) | ukb-b-20531 |
| rs8020365 | A | T | 0.220415 | 0.0151033 | 0.00154649 | 1.60E-22 | 0.198962449 | 95.37827548 | Leg fat percentage (right) | ukb-b-20531 |
| rs8062006 | C | G | 0.287981 | -0.00860433 | 0.00147459 | 5.40E-09 | 0.081445183 | 34.04799544 | Leg fat percentage (right) | ukb-b-20531 |
| rs8089514 | A | T | 0.368664 | 0.00732374 | 0.00134002 | 4.60E-08 | 0.072173663 | 29.87055386 | Leg fat percentage (right) | ukb-b-20531 |
| rs811054 | T | C | 0.537266 | 0.0071048 | 0.00128922 | 3.60E-08 | 0.073292745 | 30.37033942 | Leg fat percentage (right) | ukb-b-20531 |
| rs8119351 | A | G | 0.085035 | -0.0164947 | 0.00229349 | 6.40E-13 | 0.118708905 | 51.72436177 | Leg fat percentage (right) | ukb-b-20531 |
| rs812949 | C | T | 0.729204 | 0.00929018 | 0.00143561 | 9.70E-11 | 0.098331156 | 41.87697548 | Leg fat percentage (right) | ukb-b-20531 |
| rs8132491 | A | G | 0.312987 | -0.00830365 | 0.0014152 | 4.40E-09 | 0.082277761 | 34.42725773 | Leg fat percentage (right) | ukb-b-20531 |
| rs853961 | T | G | 0.505913 | 0.00935209 | 0.0012755 | 2.30E-13 | 0.122806315 | 53.7596496 | Leg fat percentage (right) | ukb-b-20531 |
| rs879620 | T | C | 0.613277 | 0.0107901 | 0.00131536 | 2.30E-16 | 0.149109154 | 67.2917275 | Leg fat percentage (right) | ukb-b-20531 |
| rs906835 | A | C | 0.682423 | -0.00791314 | 0.00138835 | 0.000000012 | 0.078000805 | 32.48626402 | Leg fat percentage (right) | ukb-b-20531 |
| rs910244 | C | A | 0.68791 | -0.00807839 | 0.00137372 | 4.10E-09 | 0.082617564 | 34.58224547 | Leg fat percentage (right) | ukb-b-20531 |
| rs9304665 | A | T | 0.763643 | 0.00939371 | 0.00150764 | 4.60E-10 | 0.091816624 | 38.82209736 | Leg fat percentage (right) | ukb-b-20531 |
| rs935166 | A | G | 0.506867 | -0.00797524 | 0.00127471 | 3.90E-10 | 0.092507425 | 39.143958 | Leg fat percentage (right) | ukb-b-20531 |
| rs9381202 | G | T | 0.312061 | 0.00822885 | 0.00137613 | 2.20E-09 | 0.085184693 | 35.75685936 | Leg fat percentage (right) | ukb-b-20531 |
| rs9389857 | T | C | 0.052432 | 0.0171126 | 0.00286877 | 2.40E-09 | 0.084805267 | 35.58283418 | Leg fat percentage (right) | ukb-b-20531 |
| rs9449999 | G | A | 0.589165 | -0.00747673 | 0.00129944 | 8.70E-09 | 0.079371422 | 33.10632184 | Leg fat percentage (right) | ukb-b-20531 |
| rs9512686 | A | G | 0.331728 | -0.00780344 | 0.00135941 | 9.40E-09 | 0.079028919 | 32.95120267 | Leg fat percentage (right) | ukb-b-20531 |
| rs9515455 | A | G | 0.414831 | 0.0105928 | 0.00130396 | 4.50E-16 | 0.14665199 | 65.99226075 | Leg fat percentage (right) | ukb-b-20531 |
| rs9563641 | G | A | 0.182739 | -0.0097824 | 0.00166266 | 0.000000004 | 0.082692768 | 34.61656229 | Leg fat percentage (right) | ukb-b-20531 |
| rs9568867 | A | G | 0.129187 | 0.0141597 | 0.00191977 | 1.60E-13 | 0.124090295 | 54.40135321 | Leg fat percentage (right) | ukb-b-20531 |
| rs9571687 | A | C | 0.329436 | -0.00832178 | 0.00136169 | 9.90E-10 | 0.088640922 | 37.34874081 | Leg fat percentage (right) | ukb-b-20531 |
| rs962929 | A | T | 0.511883 | -0.00701742 | 0.00127989 | 4.20E-08 | 0.072601379 | 30.0614309 | Leg fat percentage (right) | ukb-b-20531 |
| rs9641499 | A | C | 0.434718 | -0.0097825 | 0.00128722 | 3.00E-14 | 0.130741085 | 57.75560736 | Leg fat percentage (right) | ukb-b-20531 |
| rs972283 | G | A | 0.511937 | -0.010255 | 0.00127534 | 8.90E-16 | 0.144113401 | 64.65756827 | Leg fat percentage (right) | ukb-b-20531 |
| rs9783304 | T | G | 0.689277 | -0.0134784 | 0.00138024 | 1.60E-22 | 0.198932357 | 95.36026802 | Leg fat percentage (right) | ukb-b-20531 |
| rs9788550 | C | G | 0.247498 | -0.0120628 | 0.00148597 | 4.70E-16 | 0.146474334 | 65.89859745 | Leg fat percentage (right) | ukb-b-20531 |
| rs9839081 | A | G | 0.325082 | -0.0100183 | 0.00138302 | 4.40E-13 | 0.120219338 | 52.47242604 | Leg fat percentage (right) | ukb-b-20531 |
| rs9843653 | C | T | 0.511535 | 0.0152838 | 0.0012761 | 4.70E-33 | 0.271965589 | 143.4475961 | Leg fat percentage (right) | ukb-b-20531 |
| rs9852062 | A | T | 0.556841 | -0.0084912 | 0.00128843 | 4.40E-11 | 0.101612883 | 43.43266555 | Leg fat percentage (right) | ukb-b-20531 |
| rs9860326 | G | C | 0.328357 | 0.00967515 | 0.00135893 | 1.10E-12 | 0.116611589 | 50.68987744 | Leg fat percentage (right) | ukb-b-20531 |
| rs9866090 | A | G | 0.228545 | 0.00991853 | 0.00152282 | 7.40E-11 | 0.099484918 | 42.42261919 | Leg fat percentage (right) | ukb-b-20531 |
| rs9867130 | G | A | 0.859295 | 0.0108666 | 0.00186489 | 5.60E-09 | 0.081236883 | 33.95321641 | Leg fat percentage (right) | ukb-b-20531 |
| rs997123 | C | T | 0.517864 | -0.00703204 | 0.00127511 | 3.50E-08 | 0.07338941 | 30.41356721 | Leg fat percentage (right) | ukb-b-20531 |

Supplementary Table 14: Information on all instrumental variables (IVs) ultimately used for Appendicular lean mass (ALM) in our study

| SNPs | effect_allele.exposure | other_allele.exposure | eaf.exposure | beta.exposure | se.exposure | pval.exposure | R^2^ | F | trait | id.exposure |
| --- | --- | --- | --- | --- | --- | --- | --- | --- | --- | --- |
| rs10068807 | G | A | 0.690817 | -0.0241893 | 0.00305435 | 1.90E-15 | 0.152708319 | 62.72042582 | Appendicular lean mass (ALM) | ebi-a-GCST90000026 |
| rs10221927 | C | A | 0.133579 | -0.0304405 | 0.00413637 | 1.30E-12 | 0.13466905 | 54.15827262 | Appendicular lean mass (ALM) | ebi-a-GCST90000026 |
| rs10283100 | G | A | 0.944673 | 0.0548823 | 0.00618178 | 4.00E-20 | 0.184668386 | 78.82019711 | Appendicular lean mass (ALM) | ebi-a-GCST90000026 |
| rs1035583 | A | G | 0.618122 | 0.0183945 | 0.00288925 | 1.10E-10 | 0.104322536 | 40.53271833 | Appendicular lean mass (ALM) | ebi-a-GCST90000026 |
| rs1036821 | A | G | 0.305458 | -0.0255077 | 0.00307579 | 3.30E-17 | 0.165016664 | 68.77478475 | Appendicular lean mass (ALM) | ebi-a-GCST90000026 |
| rs10748128 | T | G | 0.345127 | 0.0226868 | 0.00296445 | 1.70E-14 | 0.144054007 | 58.56770758 | Appendicular lean mass (ALM) | ebi-a-GCST90000026 |
| rs10753139 | C | A | 0.545935 | 0.0286803 | 0.00281686 | 1.30E-23 | 0.229519367 | 103.6661224 | Appendicular lean mass (ALM) | ebi-a-GCST90000026 |
| rs10771406 | T | G | 0.283072 | -0.0227169 | 0.00313294 | 6.90E-14 | 0.131252688 | 52.57677909 | Appendicular lean mass (ALM) | ebi-a-GCST90000026 |
| rs10787526 | A | G | 0.556763 | 0.0158588 | 0.00286167 | 2.60E-08 | 0.0810948 | 30.71153637 | Appendicular lean mass (ALM) | ebi-a-GCST90000026 |
| rs10788872 | G | A | 0.420785 | -0.0228229 | 0.00285052 | 2.70E-15 | 0.15555549 | 64.10523105 | Appendicular lean mass (ALM) | ebi-a-GCST90000026 |
| rs10807137 | T | C | 0.825089 | -0.0466364 | 0.00370859 | 1.10E-35 | 0.312438576 | 158.1365979 | Appendicular lean mass (ALM) | ebi-a-GCST90000026 |
[truncated: 505,929 more chars]
